# SVA retrotransposon insertion-associated deletion represents a novel mutational mechanism underlying large genomic copy number changes with non-recurrent breakpoints

**DOI:** 10.1186/gb-2014-15-6-r80

**Published:** 2014-06-02

**Authors:** Julia Vogt, Kathrin Bengesser, Kathleen BM Claes, Katharina Wimmer, Victor-Felix Mautner, Rick van Minkelen, Eric Legius, Hilde Brems, Meena Upadhyaya, Josef Högel, Conxi Lazaro, Thorsten Rosenbaum, Simone Bammert, Ludwine Messiaen, David N Cooper, Hildegard Kehrer-Sawatzki

**Affiliations:** 1 Institute of Human Genetics, University of Ulm, D-89081 Ulm, Germany; 2 Centre for Medical Genetics, Ghent University Hospital, B-9000 Ghent, Belgium; 3 Division of Human Genetics, Medical University Innsbruck, A-6020 Innsbruck, Austria; 4 Department of Neurology, University Hospital Hamburg Eppendorf, D-20246 Hamburg, Germany; 5 Department of Clinical Genetics, Erasmus MC, NL-3015 Rotterdam, The Netherlands; 6 Department of Human Genetics, KU Leuven, B-3000 Leuven, Belgium; 7 Institute of Medical Genetics, School of Medicine, Cardiff University, Cardiff CF14 4XN, UK; 8 Molecular Diagnostics Unit, Hereditary Cancer Program, Catalan Institute of Oncology (ICO-IDIBELL), L’Hospitalet de Llobregat, E-08908 Barcelona, Spain; 9 Department of Pediatrics, Duisburg General Hospital, D-47055 Duisburg, Germany; 10 Medical Genomics Laboratory, Department of Genetics, University of Alabama at Birmingham, Birmingham, Alabama 35294, USA

## Abstract

**Background:**

Genomic disorders are caused by copy number changes that may exhibit recurrent breakpoints processed by nonallelic homologous recombination. However, region-specific disease-associated copy number changes have also been observed which exhibit non-recurrent breakpoints. The mechanisms underlying these non-recurrent copy number changes have not yet been fully elucidated.

**Results:**

We analyze large *NF1* deletions with non-recurrent breakpoints as a model to investigate the full spectrum of causative mechanisms, and observe that they are mediated by various DNA double strand break repair mechanisms, as well as aberrant replication. Further, two of the 17 *NF1* deletions with non-recurrent breakpoints, identified in unrelated patients, occur in association with the concomitant insertion of SINE/variable number of tandem repeats/*Alu* (SVA) retrotransposons at the deletion breakpoints. The respective breakpoints are refractory to analysis by standard breakpoint-spanning PCRs and are only identified by means of optimized PCR protocols designed to amplify across GC-rich sequences. The SVA elements are integrated within *SUZ12P* intron 8 in both patients, and were mediated by target-primed reverse transcription of SVA mRNA intermediates derived from retrotranspositionally active source elements. Both SVA insertions occurred during early postzygotic development and are uniquely associated with large deletions of 1 Mb and 867 kb, respectively, at the insertion sites.

**Conclusions:**

Since active SVA elements are abundant in the human genome and the retrotranspositional activity of many SVA source elements is high, SVA insertion-associated large genomic deletions encompassing many hundreds of kilobases could constitute a novel and as yet under-appreciated mechanism underlying large-scale copy number changes in the human genome.

## Background

Large deletions encompassing the *NF1* gene region at 17q11.2 are present in 5 to 10% of patients with neurofibromatosis type 1 (NF1; MIM #162200) [1]. The majority of large *NF1* deletions have recurrent breakpoints and are mediated by nonallelic homologous recombination (NAHR) between various highly homologous duplicated sequences located within the *NF1* gene region [2]. Three types of recurrent NAHR-mediated *NF1* deletions have been identified, distinguishable by virtue of the locations of their breakpoints. Most frequent are type-1 *NF1* deletions of 1.4 Mb, with breakpoints located within low-copy repeats termed NF1-REPa and NF1-REPc [3-5]. It is estimated that 70 to 80% of all large *NF1* deletions are type-1 [6,7]. Less frequent are the type-2 *NF1* deletions, which span 1.2 Mb and have their breakpoints located within the *SUZ12* gene and its pseudogene *SUZ12P*. NAHR between *SUZ12* and *SUZ12P* gives rise to type-2 *NF1* deletions, which are observed in 10 to 20% of all large *NF1* deletions [6,8]. By contrast, type-3 *NF1* deletions, which are characterized by breakpoints located within NF1-REPb and NF1-REPc [7,9,10], are rare, accounting for only 1.4 to 4% of all large *NF1* deletions [6,7]. All three types of *NF1* deletion (that is, type-1, 2 and 3) are considered to exhibit recurrent breakpoints that occur preferentially within genomic regions encompassing only a few kilobases. In particular, type-1 *NF1* deletions are characterized by pronounced breakpoint recurrence, with an estimated 80% of all type-1 deletions exhibiting breakpoints located within an NAHR hotspot termed PRS2, which spans 2 kb [11,12]. The existence of a recombination hotspot such as PRS2 is noteworthy since the recombining low-copy repeats, NF1-REPa and NF1-REPc, exhibit high sequence homology over an extended region encompassing approximately 50 kb.

In addition to *NF1* deletions with recurrent breakpoints mediated by NAHR between low-copy repeats, a number of *NF1* deletions have been identified that appear to exhibit non-recurrent breakpoints. These so-called ‘atypical *NF1* deletions’ tend to be heterogeneous in terms of their size and the number of genes located within the deleted region. An estimated 8 to 10% of all large *NF1* deletions are atypical [6,7]. However, of the atypical *NF1* deletions reported to date, only 6 have been characterized at the highest level of resolution so as to reveal the precise locations of the deletion breakpoints (Tables S1 and S2 in Additional file 1). None of these 6 deletions exhibited extended sequence homologies or additional rearrangements at their breakpoints, suggesting that non-homologous end joining (NHEJ) had been responsible for mediating them. Owing to the small number of atypical *NF1* deletions so far characterized, it is unclear whether other mutational mechanisms are also involved. In this study, we identified and characterized the precise breakpoints of 17 atypical *NF1* deletions and found that a variety of different mutational mechanisms, including replication-based mechanisms involving multiple template switching events and a novel SINE/variable number of tandem repeats/*Alu* (SVA) insertion-associated mechanism, are responsible for their occurrence.

## Results

**Figure 1:**
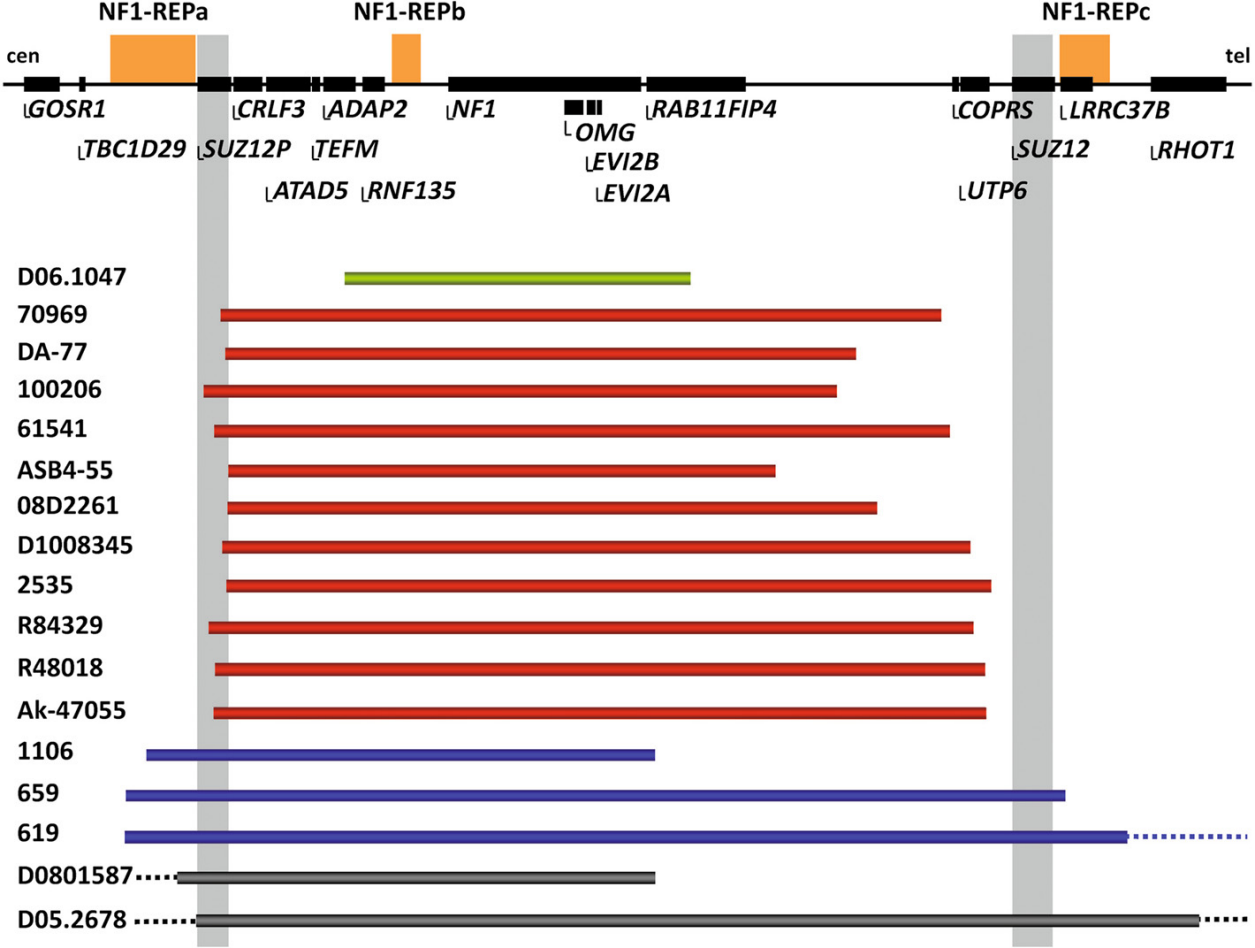
**Location of the breakpoints of the 17 atypical *NF1 *deletions.** At the top is a schematic representation of the *NF1* gene and its flanking regions. The relative positions of the genes located within this region are denoted by horizontal black bars. Below, the extents of the 17 *NF1* deletions analyzed are indicated by horizontal bars. The centromeric breakpoints of the deletions depicted by red bars are located within *SUZ12P*. None of these deletions had telomeric breakpoints located within *SUZ12*. The deletions depicted by blue bars exhibit breakpoints located within NF1-REPa. Two deletions (grey bars) extended beyond the region indicated here in a centromeric direction (indicated by dotted lines). The patient identification numbers are given on the left. cen, centromere; tel, telomere.

Custom-designed multiplex ligation-dependent probe amplification (MLPA) and targeted array comparative genomic hybridization (array CGH) were performed to identify the breakpoint regions of the 17 atypical *NF1* deletions spanning between 519 kb and 5.9 Mb (Figure 1). Breakpoint-spanning PCRs, using the Expand Long Template PCR system under standard conditions with primers designed on the basis of the array CGH results, were successful in the case of 15 of the 17 atypical *NF1* deletions. Sequence analysis of the corresponding PCR products provided the precise breakpoint locations of the 15 deletions that exhibited simple breakpoints with readily interpretable transitions from centromeric to telomeric breakpoint-flanking sequences. Short microinsertions, of 9, 10 and 11 bp, respectively, were observed at the breakpoints of 3 of the 15 deletions (Table 1). These microinsertions were homologous to sequences located in the immediate vicinity of the breakpoint-flanking regions. Hence, the short microinsertions are likely to have been mediated by replication-associated template switching, which will also have caused the large *NF1* deletions identified in these patients (Figures S1 to S3 in Additional file 1). Microhomologies of between 1 bp and 52 bp were observed at the breakpoints of 13 of the 15 deletions, whereas 4 of these 13 deletions exhibited microhomologies ≥ 6 bp (Table 1). Single nucleotide changes (SNCs) in relation to the human genome reference sequence that do not represent known polymorphisms (that is, not present in dbSNP) were identified in the breakpoint-flanking regions of 4 of the 15 deletions (Table 1; Figure S4 in Additional file 1). The breakpoints of 3 of the 15 atypical *NF1* deletions with simple breakpoints were located within *Alu* elements that exhibited pairwise sequence homologies between 79 and 89% (Table 1), suggestive of *Alu*-mediated NAHR being the potential mechanism underlying the corresponding deletions.

**Table 1:** **Breakpoint positions and sequence features of the 17 atypical *NF1 *** **deletions**

**Patient**	**Breakpoint locations**^**a**^	**Deletion size (bp)**	**Microhomology (bp)**^**b**^	**Insertion at breakpoint**	**SNCs**^**c**^	**Mosaic**	**Proximal (distal) breakpoint location**	**SINEs located at proximal and distal breakpoints**^**d **^**[homology]**^**e**^	**Postulated mutational mechanism**^**f**^
08D2261	29,102,848 (30,079,302)	976,455	−	–	–	Yes	*SUZ12P* (between *RAB11FIP4* and *COPRS*)	–	NHEJ
100206	29,065,415 (30,016,354)	950,940	–	–	*+*	Yes	*SUZ12P* (between *RAB11FIP4* and *COPRS*)	–	NHEJ/ RBM
D1008345	29,094,424 (30,218,204)	1,123,781	1	–	–	No	*SUZ12P* (*UTP6*)	–	NHEJ
D05.2678	28,142,439 (34,112,082)	5,969,644	1	–	–	No	*SSH2* (*MMP28*)	–	NHEJ
R84329	29,074,557 (30,223,384)	1,148,828	1	TGTCCCCTCTG	*+*	Yes	*SUZ12P* (*UTP6*)	–	NHEJ/RBM
70969	29,092,903 (30,175,393)	1,082,491	1	GGCCAGGTT	–	No	*SUZ12P* (between *RAB11FIP4* and *COPRS*)	–	NHEJ/RBM
619	28,946,218 (31,954,580)	3,008,363	2	GTAGCAGAAT	–	No	NF1REPa (*ASIC2*)	–	NHEJ/RBM
61541	29,082,032 (30,187,273)	1,105,242	2	–	*+*	Yes	*SUZ12P* (between *COPRS* and *UTP6*)	–	NHEJ/RBM
2535	29,101,686 (30,250,762)	1,149,077	2	–	–	No	*SUZ12P* (between *UTP6* and *SUZ12*)	–	NHEJ
R48018	29,084,006 (30,241,383)	1,157,378	2	–	–	No	*SUZ12P* (between *UTP6* and *SUZ12*)	–	NHEJ
Ak-47055	29,082,023 (30,243,011)	1,160,989	4	–	–	Yes	*SUZ12P* (between *UTP6* and *SUZ12*)	–	NHEJ
D06.1047	29,264,225 (29,783,515)	519,291	6	–	–	Yes	*ADAP2* (*RAB11FIP4*)	–	MMEJ/RBM
659^g^	28,948,946 (30,345,260)	1,396,315	20	–	–	Yes	NF1REPa (NF1REPc)	*Alu*Y (*Alu*Sp) [84% in 135 bp]	*Alu*-mediated NAHR/MMEJ/RBM
1106	29,001,813 (29,765,892)	764,080	24	–	+	No	NF1REPa (*RAB11FIP4*)	*Alu*Sz6 (FLAM_C) [79% in 112 bp]	*Alu*-mediated NAHR/MMEJ/RBM
D0801587	27,726,501 (29,729,864)	2,003,364	52	–	–	Yes	*TAOK1* (*RAB11FIP4*)	*Alu*Y (*Alu*Y) [89% in 301 bp]	*Alu*-mediated NAHR/MMEJ/RBM
DA-77/grandmother	29,100,005 (30,101,550)	1,001,546	2	SVA_F1 element	–	Yes^h^	*SUZ12P* (between *RAB11FIP4* and *COPRS*)	–	SVA insertion
ASB4-55	29,103,071 (29,969,839)	866,769	–	SVA_F element	–	Yes	*SUZ12P* (between *RAB11FIP4* and *COPRS*)	–	SVA insertion

^a^Indicated are the genomic positions of the centromeric breakpoints and, in parentheses, the positions of the telomeric breakpoints (hg19). The indicated genomic positions correspond to the nucleotides immediately before and immediately after the deleted DNA sequence. Where microhomology (100% sequence identity) was present at the deletion breakpoints, the position of the centromeric breakpoint was defined as the last nucleotide adjacent to the region of microhomology whilst the position of the telomeric breakpoint was defined as the first nucleotide adjacent to the region of microhomology.

^b^Indicated are the numbers of nucleotides exhibiting microhomology at the corresponding breakpoints. Microhomology was defined as one or more perfectly matching basepairs.

^c^Plus signs indicate single nucleotide changes (SNCs) identified within the breakpoint-flanking regions (±75 bp) of the patients but absent from the reference sequence of the human genome (hg19). Where an SNC was identified within the breakpoint-flanking sequence, a replication-based mechanism (RBM) was considered to have been responsible for causing the SNC as well as the large *NF1* deletion. Dashes indicate that SNCs were not detected in the respective breakpoint-flanking sequences.

^d^SINEs and LINEs spanning the breakpoints were identified by means of the Repeat Masker track of the UCSC Genome Browser (date: 04.11.2013). Indicated are the *Alu* elements that were located at both breakpoints and in direct orientation with respect to each other. Dashes denote that directly oriented SINEs or LINEs located at both breakpoints and harboring the respective deletion breakpoints at homologous positions were not identified.

^e^Indicated is the homology between the *Alu* elements as well as the length of the *Alu* sequences exhibiting the indicated homology.

^f^NHEJ, non-homologous end joining; MMEJ, microhomology-mediated end joining; *Alu*-mediated NAHR,*Alu*-mediated nonallelic homologous end joining; RBM, replication-based mechanisms such as fork stalling and template-switching (FoSTeS) and microhomology-mediated break-induced replication (MMBIR). SVA insertion, SVA insertion-associated deletion.

The deletions in patients 100206 and 61541 may have been mediated by either NHEJ or RBM. In both patients, SNCs were detected in breakpoint-flanking regions suggestive of the involvement of a low-fidelity DNA polymerase and a RBM. However, the involvement of NHEJ in the occurrence of these deletions cannot be excluded. In addition, the deletions identified in patients R84239, 70969 and 619 may have been caused by either NHEJ or RBM. The microinsertions identified at the breakpoints exhibit homology to sequences closely flanking the breakpoints and hence could have been caused by multiple template switching events indicative of a replication-based mechanism. However, we cannot exclude the possibility that the microinsertions occurred at random and that the deletions were mediated by NHEJ.

The deletion identified in patient D06.1047 may have been mediated by MMEJ since microhomology > 5 bp is indicative of MMEJ [29]. However, microhomology has also been observed at rearrangement breakpoints mediated by a replication-based mechanism and hence the deletion in this patient could also have been caused by an RBM.

In the case of patients 659, 1106 and D0801587, several mechanisms (*Alu*-mediated NAHR, MMEJ or RBM) could have caused the respective deletions. We observed directly oriented *Alu* elements at the deletion breakpoints of all three patients. Hence, the deletions may have been caused by *Alu*-mediated NAHR. Alternatively, the deletions could have been caused by MMEJ or RBM since microhomologies of 20 to 52 bp were observed at the breakpoints.

^g^The centromeric deletion breakpoint in patient 659 is located within intron 7 of the *LRRC37B*pseudogene in NF1-REPa whereas the telomeric breakpoint is located within intron 2 of the functional *LRRC37B* gene located in NF1-REPc. The breakpoints of the deletion in patient 659 do not overlap with the nonallelic homologous recombination (NAHR) hotspots of type-1 *NF1* deletions, termed PRS1 and PRS2. Hence, this atypical *NF1* deletion is not considered to exhibit recurrent breakpoints located within regions of extended sequence homology.

^h^The grandmother of patient DA-77 exhibits somatic mosaicism since she possesses cells harboring the deletion alongside normal cells. Her granddaughter, patient DA-77, harbors the deletion in all of her cells.

### Analysis of deletion breakpoint-flanking regions

We investigated whether non-B DNA-forming sequence motifs as well as direct and inverted repeats were overrepresented in the breakpoint-flanking regions of the 15 atypical *NF1* deletions with simple breakpoints. To this end, we determined their frequency within a 300 bp fragment flanking each deletion breakpoint (that is, 150 bp on both sides). Non-B DNA-forming sequences were identified within 53% of the deletion breakpoint-flanking fragments (Tables S3 to S5 in Additional file 1). Similarly, direct and inverted repeats in the size range of ≥ 6 bp up to 150 bp, as identified by MEME Suite sequence analysis tools, were detected in 77% of the deletion breakpoint-flanking regions (Table S6 in Additional file 1). However, when compared with a control sequence dataset, no overrepresentation of either non-B DNA-forming sequence motifs or direct and inverted repeats was detectable within the deletion breakpoint-flanking regions (Tables S5 and S6 in Additional file 1).

To determine the frequency of repeats > 150 bp capable of forming DNA secondary structures and located within the breakpoint-flanking regions of 15 of the atypical *NF1* deletions with simple breakpoints, we screened 2 kb sequences flanking the deletion breakpoints on either side by means of BLASTN self-alignments. Only 5 of the 15 deletion breakpoint-flanking regions harbored repeats in the size range of 244 to 316 bp (Table S7 in Additional file 1). As compared with a control dataset, the number of repeats was not found to be overrepresented in the deletion breakpoint-flanking regions (*P* = 0.99, two-tailed Fisher’s exact test; Tables S8 and S9 in Additional file 1). We also searched for large direct and inverted repeats (≥ 1 kb) located within 20 kb regions flanking the deletion breakpoints on either side by means of BLASTN alignments. A 5.7 kb inverted repeat exhibiting 99% sequence homology was identified that harbored one of the deletion breakpoints in patient 619. Remarkably, one of the breakpoints of another atypical *NF1* deletion (in patient 659) was located only 2.7 kb telomeric to one of the breakpoints in patient 619 and between both 5.7 kb repeats (Figure S5 in Additional file 1). These inverted repeats were the only direct and inverted repeat sequences ≥ 1 kb identified in the vicinity of the breakpoints of the 15 atypical *NF1* deletions (Table S10 in Additional file 1). We suspect that these 5.7 kb inverted repeats located within NF1-REPa may have contributed to the occurrence of the atypical *NF1* deletions by mediating the formation of a hairpin structure, thereby inducing DNA double strand breaks underlying the deletions (Figure S5 in Additional file 1).

We also investigated whether SINEs and LINEs spanning the breakpoints, or located immediately adjacent to the breakpoints, might be overrepresented at the breakpoints of the 15 atypical *NF1* deletions with simple breakpoints as compared with a control dataset of sequences. When the centromeric and telomeric breakpoints of the 15 atypical *NF1* deletions with simple breakpoints were considered, SINEs or LINEs were located at or immediately adjacent to 22 of the 30 breakpoints (73%). However, the number of SINEs and LINEs located at the *NF1* deletion breakpoints was not significantly elevated as compared with a control sequence dataset (Table S11 in Additional file 1).

### SVA element insertions at the deletion breakpoints in two patients

**Figure 2:**
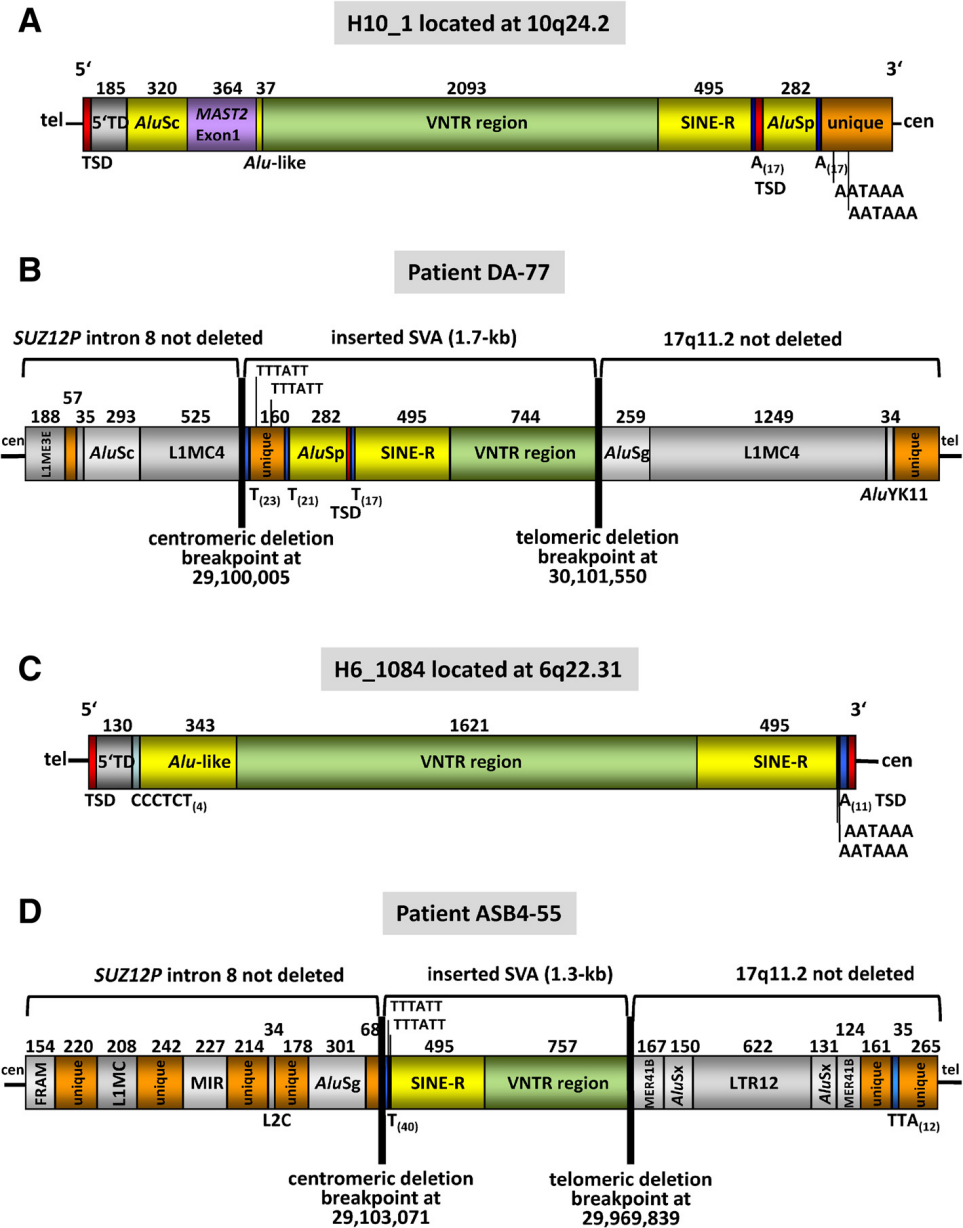
**Structure of the SVA elements inserted at the *NF1 *deletion breakpoints and their source elements. (A)** The SVA_F1 element H10_1 spans 4,039 bp and is the likely source element of the SVA copy that inserted within *SUZ12P* intron 8 in the grandmother of patient DA-77. Starting at its 5′ end, H10_1 comprises a target site duplication (TSD), a transduced sequence (5′TD) and a full-length *Alu*Sc from chromosome 9p13.3, a transduced partial exon 1 of *MAST2*, an *Alu*-like region, a variable number of tandem repeats (VNTR) region, a SINE-R, a polyA_(17)_ tract, the second TSD, an *Alu*Sp element, a second polyA_(17)_ tract and a non-repetitive, unique sequence resulting from a 3′ transduction that harbors two polyadenylation signals (AATAAA). The size of each region is given in basepairs. **(B)** A copy of the source element H10_1 integrated within *SUZ12P* intron 8 in the grandmother of patient DA-77. The SVA insertion was associated with a deletion of approximately 1 Mb. The inserted SVA spans 1.7 kb and is 5′ truncated. **(C)** Structure of the putative source SVA element H6_1084, which spans 2,691 bp and belongs to the SVA_F subfamily. A copy of H6_1084 is presumed to have integrated within *SUZ12P* intron 8 in patient ASB4-55. Full-length H6_1084 has the following structure starting from the 5′ end: a TSD, a 5′TD from chromosome 12p11.21, a CCCTCT_(4)_ repeat, a 343-bp *Alu*-like region, a GC-rich VNTR region, a SINE-R element, two polyadenylation signals, a polyA_(11)_ tract and the second TSD. The length of each region is indicated in basepairs. **(D)** Structure of the 5′ truncated copy of H6_1084 that has integrated within *SUZ12P* intron 8 in patient ASB4-55. The SVA insertion was associated with an atypical *NF1* deletion of 867 kb. The SVA integration sites within *SUZ12P* intron 8 demarcate the centromeric breakpoints of the atypical *NF1* deletions.

In contrast to the aforementioned 15 deletions with simple breakpoints, breakpoint-spanning PCRs were not successful under standard conditions in 2 of the 17 atypical *NF1* deletions investigated. However, a combination of semi-specific PCR, inverse PCR and PCR analysis of somatic cell hybrids made it possible to narrow down the deletion breakpoint regions in patient DA-77 as schematically indicated in Figures S6 and S7 (Additional file 1). GenomeWalker analysis of the breakpoint regions in this patient then revealed the insertion of an SINE/VNTR/*Alu* (SVA) retrotransposon, which is absent from the human genome reference sequence (hg19), at the corresponding genomic position (Figure S6 in Additional file 1). SVA elements are composite retrotransposons that vary in size from 700 bp up to 4 kb [13,14]. In order to amplify across the inserted SVA element and the deletion breakpoints, we performed PCR using primers with a high melting-temperature and PCR conditions optimized to amplify GC-rich sequences (Figure S8 and Table S12 in Additional file 1). The application of an optimized PCR protocol was necessary since SVA elements contain a variable number of tandem repeats (VNTRs) region that is extremely GC-rich and can extend over several hundred basepairs. Sequence analysis of the resulting PCR product indicated that the inserted SVA spans 1.7 kb and exhibits 99% sequence homology to its probable source element H10_1 located on chromosome 10q24.2 (Figure 2; Figure S9 in Additional file 1). H10_1 is known to be one of the most retrotranspositionally active SVA source elements so far identified in the human genome [13,15]. This source element belongs to the evolutionarily youngest subfamily of SVA elements (SVA_F1) and encompasses a VNTR region of 2,093 bp with a GC-content of 79% (Figure 2; Figure S10 in Additional file 1). Compared with its source element H10_1, the SVA element inserted at the deletion breakpoint of patient DA-77 is truncated at its 5′ end (Figure 2).

PCR-based techniques designed to identify unknown DNA sequence inserted at deletion breakpoints also facilitated the analysis of the atypical *NF1* deletion identified in patient ASB4-55 (Figures S11 and S12 in Additional file 1). Inverse and semi-specific PCR indicated that the centromeric deletion breakpoint was located immediately adjacent to a polyT_(40)_ tract that is present in patient ASB4-55 but absent from the reference sequence of human chromosome 17 at the corresponding genomic position. Under the assumption that an SVA insertion could also have occurred in patient ASB4-55 at the breakpoint of the large *NF1* deletion that was also refractory to analysis by breakpoint-spanning PCR under standard conditions, we performed breakpoint-spanning PCRs under conditions optimized to amplify across sequences with a high GC-content (Table S12 in Additional file 1). By these means, we successfully amplified across the deletion breakpoints, and sequence analysis of the PCR product confirmed the insertion of a SVA element in this patient as well (Figure S13 in Additional file 1). BLAT sequence alignments against the human genome reference sequence indicated that the inserted SVA exhibited maximum (99.5%) sequence homology to the SVA_F element H6_1084 located on chromosome 6q22.31, which was therefore deemed most likely to be the source element of the SVA inserted at the deletion breakpoint in patient ASB4-55. Whereas full-length H6_1084 spans 2.69 kb, the copy inserted at the deletion breakpoint in patient ASB4-55 spans only 1.3 kb and is 5′-truncated (Figure 2; Figures S14 and S15 in Additional file 1).

Remarkably, the sites of the two SVA insertions within *SUZ12P* intron 8, identified in patients ASB4-55 and DA-77, are separated by only 3,067 bp. SVA elements are thought to integrate within genomic sequences via target-primed reverse transcription (TPRT) mediated by the LINE 1 protein machinery [16-19]. One of the hallmarks of L1-mediated retrotransposition of SVAs is that they often integrate at DNA sites resembling the L1 endonuclease consensus cleavage sites such as 5′-TTTT/A-3′ or 5′-CTTT/A-3′ [20]. The SVA elements we identified within *SUZ12P* intron 8 most likely integrated via TPRT since L1 endonuclease cleavage sites 5′-CTTT/A-3′ were detected at the corresponding integration sites within *SUZ12P* intron 8 (Figures S16 and S17 in Additional file 1). Furthermore, long polyT tracts were noted at the integration sites; such tracts also represent hallmarks of L1-mediated TPRT (Figures S9 and S15 in Additional file 1). Both SVA elements identified at the deletion breakpoints of patients DA-77 and ASB4-55 were inserted into the plus strands within *SUZ12P* intron 8. The telomeric breakpoints of the deletions in patients DA-77 and ASB4-55 are separated by 132 kb; whereas the deletion in patient DA-77 encompasses 1,001,546 bp, the deletion in patient ASB4-55 spans 866,769 bp.

Patient ASB4-55 exhibited somatic mosaicism, with 93% of her blood cells harboring the deletion and 7% lacking the deletion (Table S13 in Additional file 1). Somatic mosaicism was also detected in the grandmother of patient DA-77 who passed on the atypical *NF1* deletion to her offspring (Figures S18 and S19 in Additional file 1). FISH analysis of blood cells from the grandmother of patient DA-77 indicated that 75% of her blood cells harbored the deletion whereas 25% were normal. The SVA insertion at the deletion breakpoints was confirmed by breakpoint-spanning PCR using DNA from the grandmother and the cousins of patient DA-77 (Figures S8 and S19 in Additional file 1).

Neither of the SVA elements identified at the *NF1* deletion breakpoints appeared to be a frequent insertion/deletion polymorphism within *SUZ12P* intron 8 as determined by PCR analysis of DNA samples derived from 100 healthy control individuals (Figures S20 and S22 and Table S14 in Additional file 1). Further, these SVA elements identified within *SUZ12P* intron 8 are not listed in the database of polymorphic retrotransposons, dbRIP [21], nor were they reported as SVA insertion polymorphisms in the 1000 Genomes Project dataset [22].

In order to confirm that the SVA insertions occurred *de novo* within *SUZ12P* intron 8 in patient ASB4-55 and the grandmother of patient DA-77, and to establish whether or not these insertions occurred prior to the somatic *NF1* deletion, we performed PCR and SNP analysis using blood-derived DNA from the patients. These analyses did not indicate the presence of the SVA insertion in the absence of the large *NF1* deletion (Figures S20 to S23 and Table S15 in Additional file 1). Hence, we conclude that in both patients, the atypical *NF1* deletions occurred concomitantly with the SVA insertions during early postzygotic development.

### Somatic mosaicism

In addition to the mosaicism noted in patient ASB4-55 and in the grandmother of patient DA-77, a further eight of the 17 atypical *NF1* deletions investigated were found to exhibit somatic mosaicism (Table S13 in Additional file 1). Thus, taken together, at least 10 of the 17 atypical *NF1* deletions (59%) were of postzygotic origin.

### Location of the atypical *NF1* deletion breakpoints

**Figure 3:**
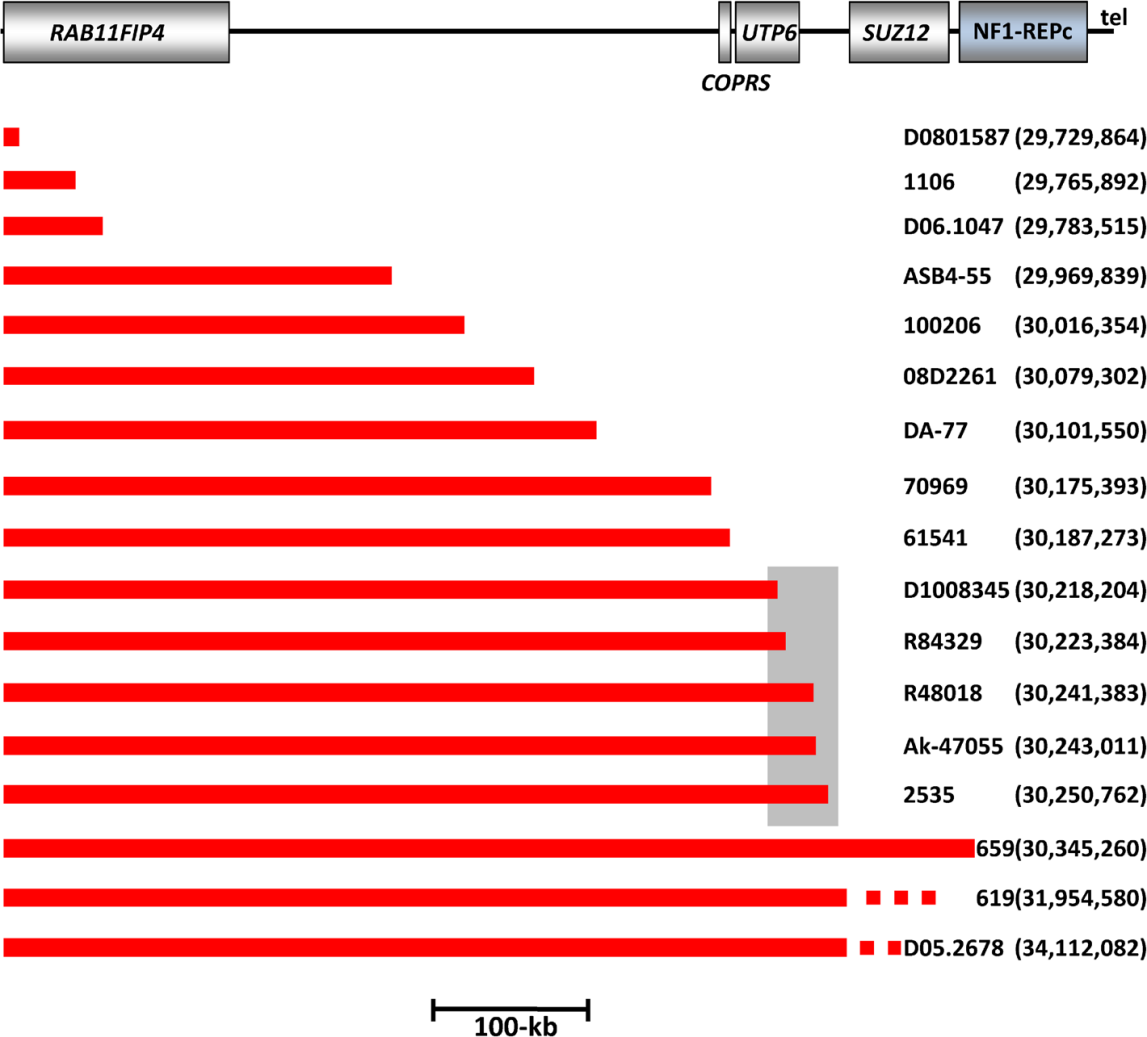
**Locations of the telomeric breakpoints identified in the 17 atypical *NF1 *deletions.** A schematic representation of the genes located within the region is given on top. The extent of each of the 17 atypical *NF1* deletions is indicated by a red bar. The centromeric breakpoints of these deletions differ from each other and are not indicated on this schema. The numbering of the breakpoint locations is according to the human GRCh37/hg19 assembly. Five deletions exhibited breakpoints that were located within a 32.6 kb region (demarcated by a grey box). tel, telomeric direction.

**Figure 4:**
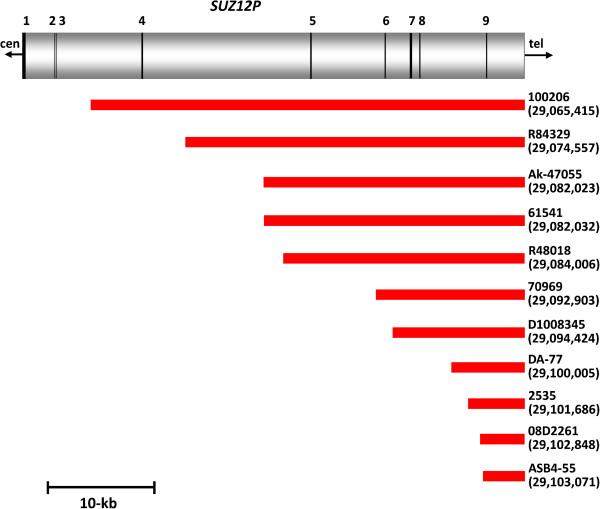
**Centromeric breakpoint positions of the 11 atypical *NF1 *deletions with centromeric breakpoints located within *SUZ12P*.** The exon-intron structure of *SUZ12P* is indicated as well as the numbering of the exons presented as vertical black lines. The extent of each of the 11 atypical *NF1* deletions is shown by red bars. The telomeric breakpoints of these deletions differ from each other and are not indicated on this schema. The numbering of the centromeric breakpoint locations is given according to the human GRCh37/hg19 assembly. tel, telomeric direction.

The 17 atypical *NF1* deletions investigated here exhibited considerable differences in terms of their sizes and breakpoint locations (Figure 1). Nevertheless, the majority of the breakpoints were found to be located within the genomic region flanked by NF1-REPa and NF1-REPc. Only two deletions, those harbored by patients 619 and D05.2678, exhibited breakpoints telomeric to NF1-REPc (located 1.53 Mb and 3.69 Mb distal to NF1-REPc). If these two deletions are excluded from consideration, the telomeric breakpoints of all of the remaining 15 atypical *NF1* deletions were located within a 615-kb region between *RAB11FIP4* and NF1-REPc (Figure 3). Remarkably, 5 of these 15 deletions had telomeric breakpoints located within a 32.6 kb stretch, indicative of an overrepresentation of breakpoints within this genomic region (Table S16 in Additional file 1; *P* < 0.0001). In addition, the centromeric breakpoints of the atypical *NF1* deletions investigated were found to be preferentially located within a specific genomic region: 11 deletions exhibited centromeric breakpoints located within *SUZ12P,* thereby indicating an overrepresentation of such breakpoints within 39 kb of *SUZ12P* sequence (Figure 4; Table S17 in Additional file 1; *P* < 0.0001). However, a clustering of these 11 deletion breakpoints within a specific region of *SUZ12P* encompassing only a few hundred basepairs was not observed (Table S18 in Additional file 1). This notwithstanding, the centromeric breakpoints of the unrelated patients Ak-45077 and 61541, located within *SUZ12P* intron 4, are separated by 10 bp (Table 1; Table S18 in Additional file 1). It should be noted that the breakpoints of the recurrent type-2 *NF1* deletions are also located within *SUZ12P*[23]. Although atypical *NF1* deletions do not appear to be mediated by NAHR between segmental duplications, on the basis that extended sequence homology between the centromeric and telomeric deletion breakpoints was not evident, we noted that two of the atypical *NF1* deletions investigated exhibited breakpoints that overlapped with the breakpoints of type-2 *NF1* deletions (Table S18 in Additional file 1). The reasons why the centromeric breakpoints of the atypical *NF1* deletions accumulate within *SUZ12P* are currently unknown. No overrepresentation of non-B DNA forming sequences, short repeats identified by MEME Suite analysis, or repeats >150 bp was observed in the regions flanking the breakpoints within *SUZ12P* as compared with a control sequence dataset (Tables S19 to 21 in Additional file 1).

## Discussion

This study has provided a body of evidence to support the view that atypical *NF1* deletions are not only heterogeneous in terms of their size but also heterogeneous in relation to their underlying mutational mechanisms. Sequence analysis of the deletion breakpoints suggested that replication- and recombination-based mechanisms as well as non-replicative repair and retrotransposon-associated mutagenesis are involved in the formation of these deletions. Fifteen of the 17 atypical *NF1* deletions investigated exhibited ‘simple’ breakpoints that lacked microinsertions >11 bp at the breakpoint junctions (Table 1). Microhomologies ranging from 1 bp to 52 bp were detected at the breakpoints of 13 of these 15 atypical *NF1* deletions with simple breakpoints. Other non-recurrent disease-causing gross deletions have also been reported to exhibit breakpoint microhomologies at high frequency [24-26]. In our study, the majority of the atypical *NF1* deletions (10/17) exhibited short regions of microhomology (1 to 4 bp). We assume that some of these deletions with simple breakpoints and short regions of microhomology may have been mediated by NHEJ, a repair mechanism that is frequently associated with short microhomologies at deletion breakpoints although not dependent upon them (Table 1). By contrast, microhomology >5 bp is a prerequisite for microhomology-mediated end joining (MMEJ), an alternative pathway of NHEJ [27-30]. Four of the atypical *NF1* deletions investigated here exhibited microhomologies >5 bp at the breakpoints and hence may have been mediated by MMEJ. Microhomologies have also been observed at the breakpoints of non-recurrent genomic rearrangements mediated by replication-based mechanisms such as microhomology-mediated break-induced replication or fork stalling and template switching [26,31-37]. Hence, replication-based mechanisms may have been responsible for the atypical *NF1* deletions exhibiting microhomologies at their breakpoints.

Three of the atypical *NF1* deletions investigated here were characterized by microinsertions at the breakpoints of 9, 10 and 11 bp, respectively (Table 1). Sequence alignment indicated that these microinsertions exhibited homology to sequences closely flanking the deletion breakpoints (Figures S1 to S3 in Additional file 1). We conclude that these microinsertions probably resulted from serial template switching events during DNA replication that also caused the respective atypical large *NF1* deletions. Also indicative of the involvement of replication-based mechanisms in the formation of some of the atypical *NF1* deletions investigated are the SNCs that were observed in the breakpoint-flanking regions of 4 of the 15 atypical *NF1* deletions with simple breakpoints (27%; Table 1). These SNCs represent nucleotides that differ from the reference sequence of the human genome (hg19) but do not appear to represent polymorphisms since they are not present in dbSNP. Recently, Carvalho *et al*. [33] showed that replicative mechanisms underlying the complex copy number gains that characterize the *MECP2* gene region are associated with an increased frequency of SNCs in the breakpoint-flanking regions. SNCs are likely to be caused by the involvement of low-fidelity DNA polymerases or a replisome with reduced fidelity [33]. In similar vein, we postulate that the SNCs observed in the breakpoint-flanking regions of the atypical *NF1* deletions investigated here were introduced concomitantly with the occurrence of the large *NF1* deletions mediated by replication-based mechanisms. At the breakpoints of three of the 15 atypical *NF1* deletions with simple breakpoints, directly orientated *Alu* elements were located and we surmise that these three deletions could have been caused by *Alu*-mediated nonallelic homologous recombination.

The different types of non-recurrent copy number change in the human genome are thought to be induced either by DNA double strand breaks or by secondary structures whose formation is facilitated by specific sequence motifs such as non-B DNA-forming sequences and direct and inverted repeats [38]. Indeed, non-B DNA-forming sequences and/or inverted and direct repeats have been found to be overrepresented at the breakpoints of numerous human inherited disease-associated rearrangements [24,25,32,33,39-47]. This notwithstanding, we did not observe any overrepresentation of non-B DNA-forming sequences or direct/inverted repeats within 300 bp regions flanking the breakpoints of the 15 atypical *NF1* deletions with simple breakpoints as compared with a control dataset of DNA sequences that did not encompass atypical *NF1* deletion breakpoints (Tables S3 to S9 in Additional file 1). Nevertheless, we cannot exclude the possibility that the occurrence of some of the *NF1* deletions investigated here may have been facilitated by DNA secondary structures formed by these repeat sequences. Remarkably, we identified a 5.7 kb inverted repeat at or flanking the breakpoints of two unrelated patients with atypical *NF1* deletions (patients 659 and 619). It would appear likely that this 5.7 kb repeat, which exhibits 99% sequence identity, is causally associated with the occurrence of the corresponding deletions whose breakpoints are separated by only 2.7 kb (Figure S5 and Table S10 in Additional file 1).

The breakpoints of the atypical *NF1* deletions investigated here turned out to be non-randomly distributed; indeed, we observed a preponderance of breakpoints within specific genomic regions (Figures 3 and 4). Most notably, 11 of the 17 deletions (65%) possessed breakpoints that were located within *SUZ12P*. The pseudogene *SUZ12P* also harbors the breakpoints of the recurrent type-2 *NF1* deletions mediated by NAHR. The high frequency of centromeric deletion breakpoints within *SUZ12P* suggests that this genomic region exhibits an unusual degree of genomic instability. At the outset of this study, we expected atypical *NF1* deletions to be quite heterogeneous in terms of their size and breakpoint position. It turns out, however, that although this group of *NF1* deletions has arisen through the action of a number of different mutational mechanisms, the deletion breakpoints exhibit a certain degree of clustering. This is consistent with the view that the nature, size and location of human gene mutations are often determined either by specific characteristics of the local DNA sequence environment or by higher order features of the genomic architecture [38,48].

Intriguingly, SVA element insertions within *SUZ12P* intron 8 were observed at the breakpoints of two unrelated patients with atypical *NF1* deletions (patients DA-77 and ASB4-55). SVA elements are hominid-specific, non-autonomous, non-long terminal repeat retrotransposons that originated approximately 25 million years ago [49,50]. SVAs expand in their host genomes through an RNA intermediate and may integrate via Target-Primed Reverse Transcription (TPRT), which is mediated in *trans* by the L1 protein machinery [16,19]. Several active SVA source loci in the human genome are known to give rise to new insertions of SVA elements that may vary in size due to truncations or transductions at the 5′ or 3′ ends [13,15,51]. In the current reference sequence of the human genome (hg19), 2,676 SVA elements have been identified, encompassing 0.13% of the genome [52]. The SVA retrotransposition rate in humans has been estimated to be 1 in every 916 births [53,54]. SVAs are divided into subfamilies A to F1 based on diagnostic mutations, sequence divergence and evolutionary age [13,15,55]. One of the most active SVA elements is H10_1, located on chromosome 10q24.2, which has been identified as the source element of at least 13 further SVA_F1 elements in the human genome [13,15]. In our study, a 5′-truncated copy of H10_1 was detected at the deletion breakpoints of patient DA-77. The SVA insertion and the associated atypical *NF1* deletion had occurred *de novo* in the grandmother of patient DA-77 who exhibited somatic mosaicism with normal cells (Figures S18 and S19 in Additional file 1). The inserted SVA copy spans 1.7 kb, and hence had been reduced in size compared with the 4 kb H10_1 source element (Figure 2). Remarkably, patient ASB4-55 also possessed a *de novo* insertion of a 5′ truncated SVA element at the breakpoints of the atypical *NF1* deletion identified in this individual. The source element of this inserted SVA is likely to be H6_1084, which also belongs to the SVA_F subfamily but is located on chromosome 6q22.31 (Figure 2). The insertion of both SVA elements within *SUZ12P* intron 8 is likely to have been mediated by TPRT since long polyT tracts and sequences with homology to L1 endonuclease cleavage sites were both observed at the integration sites.

**Figure 5:**
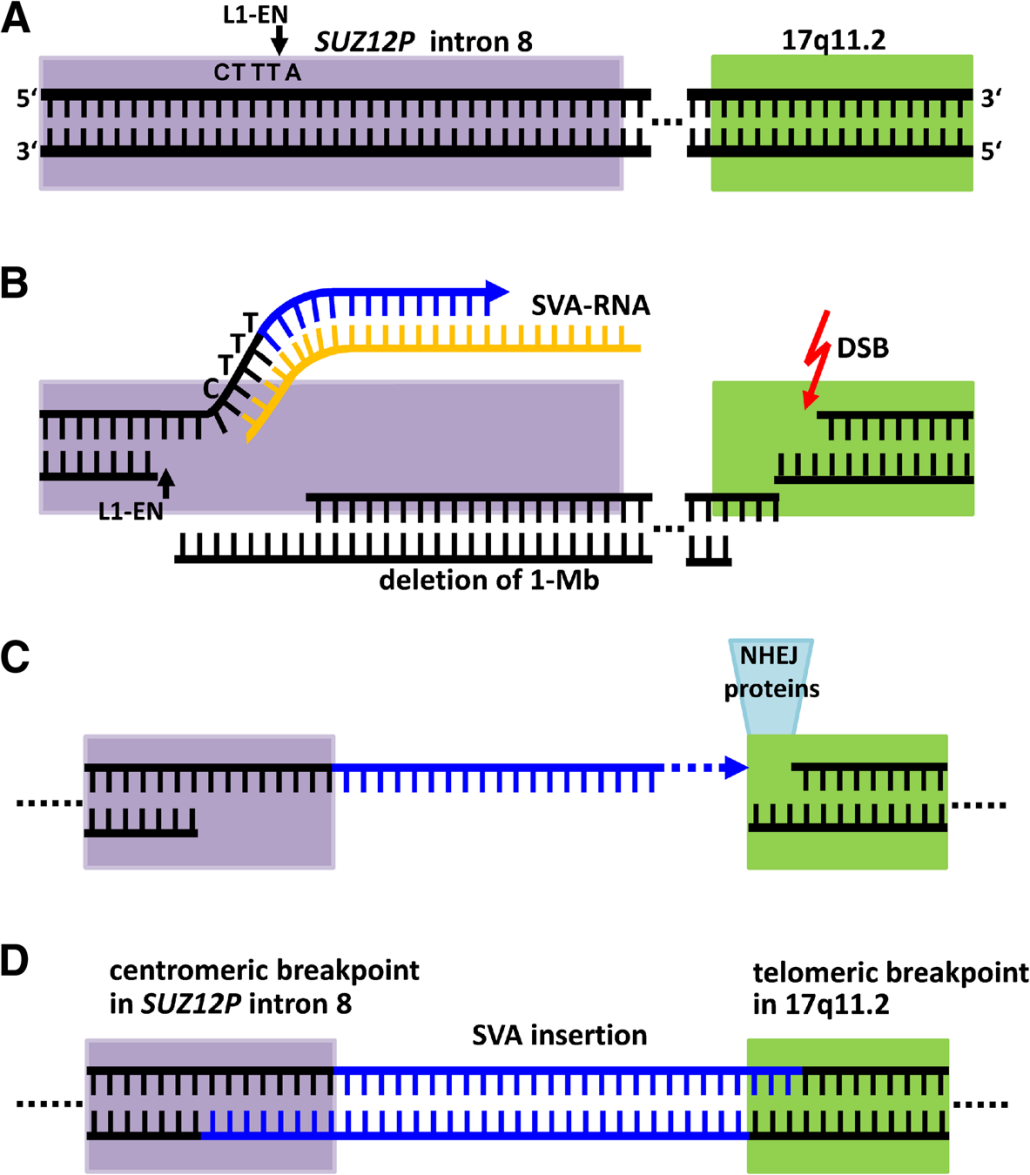
**Putative mechanism underlying the large atypical *NF1 *deletions identified in patient ASB4-55 and the grandmother of patient DA-77.** The deletions were associated with the insertion of an SVA element mediated by the LINE 1 protein machinery via target-primed reverse transcription. **(A) ***SUZ12P* intron 8 is indicated in lilac whereas the telomeric part of the *NF1* region is shown in green. The dotted lines indicate the approximately 1-Mb distance between these two regions. The SVA insertion within *SUZ12P* intron 8 is likely to have been initiated by the L1 endonuclease (L1-EN), which will have introduced a nick at the consensus cleavage site 5′-CTTT/A-3′. **(B)** Next, the SVA mRNA annealed to the T-overhang by means of its polyA-tail. Then, the L1 reverse transcriptase used the SVA mRNA as a template for reverse transcription to synthesize the SVA cDNA (blue). Second strand cleavage by the L1-EN occurred upstream of the first-strand cleavage site. Independently, a double strand break (DSB) occurred in the telomeric region of 17q11.2. **(C,D)** After dissociation of the SVA mRNA, the integration process was not finalized by recombinational repair using the downstream *SUZ12P* intron 8 sequence. Instead, the DNA ends were ligated by NHEJ to the open-ended DNA sequence located within the telomeric 17q11.2 region, between the *RAB11FIP4* and *COPRS* genes, which resulted in the deletion of the intervening sequence and hence the occurrence of the atypical *NF1* deletion **(D)**.

It is noteworthy that at least 10 of the 17 atypical *NF1* deletions investigated (59%) exhibited somatic mosaicism with normal cells. Hence, it is not only type-2 *NF1* deletions that are frequently of postzygotic origin [23,56,57] but also atypical *NF1* deletions. The atypical *NF1* deletions detected in patient ASB4-55 and the grandmother of patient DA-77 must have occurred during postzygotic development since both individuals exhibited somatic mosaicism with normal cells. In neither individual did PCR experiments detect the presence of cells harboring the SVA insertion in the absence of the atypical *NF1* deletion. Therefore, we may conclude that the SVA insertions are likely to have occurred concomitantly in association with the large *NF1* deletions during postzygotic cell division. This is consistent with previous reports demonstrating that whilst retrotransposition can occur in the germline, it is also frequent in the soma in various tissues and developmental stages [58-60], in the brain [61-65] and during cancer progression [66-69]. According to the model presented in Figure 5, the SVA insertions within *SUZ12P* intron 8 were mediated by TPRT and were associated with ligation to sequences located in the telomeric *NF1* gene region, thereby giving rise to the large *NF1* deletions. We postulate that a loop-like chromatin conformation may have brought *SUZ12P* and the region of the telomeric deletion breakpoint into close proximity. The physical interaction of these chromosomal regions may have then potentiated the ligation of the *SUZ12P* sequence, with the inserted SVA element at its end, to sequences located within the telomeric *NF1* gene region. The ligations were probably potentiated by NHEJ since proteins of the NHEJ pathway have been shown to mediate L1 retrotransposition [70]. Importantly, we did not observe extended sequence homology between the inserted SVA elements and the telomeric deletion breakpoint regions in patients DA-77 and ASB4-55. This strongly suggests that the deletions arose concomitantly with the retrotransposition events as opposed to being mediated by secondary nonallelic homologous recombination events. We speculate that the insertion of the SVA elements triggered the occurrence of the large *NF1* deletions according to the model proposed in Figure 5.

Previous studies have suggested the existence of retrotransposon insertion hotspots in the human genome [22,71-73]. This conceptual view is also supported by our observation of two *de novo* SVA insertions within *SUZ12P* intron 8 separated by only 3 kb. Moreover, our findings suggest that the increased genomic instability manifested by *SUZ12P* not only causes recurrent type-2 and atypical *NF1* deletions, but may also facilitate the integration of SVA mRNA intermediates.

The SVA insertion-associated *NF1* deletions identified in the family of patient DA-77 and in patient ASB4-55 encompassed 1 Mb and 867 kb, respectively. These deletions are unprecedentedly large since, until now, only much smaller deletions (up to 46 kb) have been reported in association with pathogenic non-long terminal repeat (LTR) retrotransposon insertion-associated deletions in the human genome (Table 2). Thus, in a comparison of the human and chimpanzee genomes, Lee *et al*. [74] identified 13 human-specific SVA insertion-associated deletions ranging from 14 bp up to 8.7 kb in length (Table S22 in Additional file 1). HeLa cell culture studies using an *in vitro* L1 recovery system have documented the occurrence of a genomic deletion >71 kb associated with the insertion of an L1 element [75]. Thus, although large genomic deletions can in principle be mediated by a retrotransposon insertion-associated mechanism, this has until now not been reported in association with SVA insertion. Our findings imply that SVA insertion-associated large genomic deletions encompassing several hundred kilobases could represent a novel type of pathogenic (and conceivably also non-pathogenic) copy number change. At present, however, it is not possible to estimate the frequency of non-LTR retrotransposon insertion-associated large deletions causing human disease because this type of rearrangement is likely to have gone undetected in many studies.

**Table 2:** **Known *de novo *** **pathogenic retrotransposon insertions associated with deletions ≥ 100 bp in the human genome**

**Gene**	**Chromosomal location**	**Disease**	**Retrotransposon (length)**	**Length of the deletion**	**Reference**
*HLA-A*	6p22.1	Leukemia^a^	SVA_F1 (2 kb)	~14 kb	[76]
*ABCD1*	Xq28	Adrenoleukodystrophy	*Alu*Yb9 (98 bp)	4,726 bp	[77]
*SERPINC1*	1q25.1	Antithrombin deficiency type 1	*Alu* (6 bp)^b^	1,444 bp	[78]
*LPL*	8p21.3	Lipoprotein lipase deficiency	*Alu*Yb9 (150 bp)	2.2 kb	[79]
*CHD7*	8q12.2	CHARGE syndrome	*Alu*Ya5/8 (75 bp)	10 kb	[80]
*PMM2*	16p13.2	Congenital disorders of glycosylation type-Ia	*Alu*Yb8 (263 bp)	28 kb	[81]
*APC*	5q22.2	Familial adenomatous polyposis	*Alu*Yb9 (93 bp)	1,599 bp	[82]
*EYA1*	8q13.3	Branchio-oto-renal syndrome	L1 Hs (3,756 bp)	17 kb	[83]
*PDHX*	11p13	Pyruvate dehydrogenase complex deficiency	L1 Hs (6,086 bp)	46 kb	[84]
*BRCA1*	17q21.31	Hereditary breast/ovarian cancer	*Alu*Y (~190 bp)	23,363 bp	[85]

^a^The germline SVA insertion-associated deletion was identified in three unrelated Japanese families. Of the individuals harboring the SVA insertion-associated deletion, one individual in each family presented with leukemia.

^b^The affected family members harbored an intragenic 1,444 bp deletion and an insertion of a polyT tract of 40 nucleotides followed by a 6 bp sequence (5′-GAGACG-3′). This 6 bp sequence, located at the 3′end of the insertion, was homologous to the consensus sequence of the free right *Alu* monomer (FRAM).

Owing to their repetitive nature, SVA and other retrotransposon insertions at deletion breakpoints are undetectable by array CGH. Furthermore, specially optimized PCR assays are necessary to detect the GC-rich SVA elements at deletion breakpoints because long GC-rich sequences are refractory to analysis by breakpoint-spanning PCRs performed under standard conditions. It follows that the incidence of SVA insertion-mediated deletions may well have been seriously underestimated in human genome pathology. Since the retrotransposition of non-LTR retro-elements occurs in the germline, early embryonic development, and in somatic cells [60-62], retrotransposon insertion-associated deletions are likely to occur in all three contexts.

## Conclusion

It has been known for some time that retrotransposons can cause human disease by inactivating genes through insertional mutagenesis [66,71,86,87]. Our findings indicate that retrotransposon insertions may also exert their influence in a pathogenic context aggravated by accompanying large genomic deletions that encompass many hundreds of kilobases leading to the loss of multiple dosage-sensitive genes.

## Materials and methods

### Patients

The deletions analyzed in this study were identified by MLPA analysis (P122-C1 NF1-area kit, MRC-Holland, The Netherlands) in 17 unrelated NF1 patients who were referred for molecular diagnostics to the participating institutions (Tables S23 and S24 in Additional file 1). This study was approved by the Institutional Review Boards of Ulm University (application number 129/09) and the Hamburg Medical Association (PV3291) and we adhered to their rules. Either the patients or their parents gave their informed consent to the molecular characterization of the deletions. High-molecular-weight DNA samples extracted from blood of the patients were analyzed in order to identify the breakpoint locations.

### Custom-designed MLPA and array CGH

To determine the positions of the deletion breakpoints more precisely, we performed custom-designed MLPA analysis with probes established previously (Table S25 in Additional file 1) [23]. By means of this analysis, the breakpoint regions could be assigned to intervals of a few tens of kilobases (Table S26 in Additional file 1). Next, we performed targeted array CGH using 8 × 15 K arrays (Agilent Technologies, Santa Clara, CA, USA) with probes located within the deletion breakpoint regions as predicted by the MLPA analyses. This array CGH probe-set included 4,891 control probes as well as 10,853 test probes. The details of probe design, as well as the analysis of the array, are described in Figure S24 (Additional file 1).

### Breakpoint identification by PCR

Breakpoint-spanning PCRs were performed using the Expand Long Template PCR system (Roche, Mannheim, Germany). The locations of the PCR primers were selected on the basis of the array CGH results (Table S27 in Additional file 1). The resulting PCR products were gel-purified (Zymoclean™ Gel DNA Recovery Kit, Zymo Research, Irvine, CA, USA) and sequenced with the BigDye Terminator v3.1 Cycle Sequencing Kit (Life Technologies, Darmstadt, Germany). Sequence alignments of the breakpoint-spanning PCR products against the reference sequence of the human genome (hg19) indicated the precise locations of the respective deletion breakpoints.

### Somatic cell hybrids and PCR analysis

Hybrid cell lines harboring only the chromosome 17 with the deletion were established for patients 659, 619, 1106, DA-77 and ASB4-55 using Epstein-Barr virus-transformed cell lines according to the procedure described previously [8]. Using DNA isolated from the hybrid cell lines, we performed PCR and sequence analysis of the respective products in order to narrow down the locations of the deletion breakpoints.

### Identification of large insertions at the deletion breakpoints in two patients

Inverse PCR, semi-specific PCR, as well as GenomeWalker™ analysis were employed to investigate the insertions at the deletion breakpoints of patients DA-77 and ASB4-55. Inverse PCR, as schematically described in Figure S25 (Additional file 1), was performed with the restriction enzymes and PCR primers listed in Tables S28 and S29 (Additional file 1). Semi-specific PCRs, employed according to the principle described in Figure S26 (Additional file 1), were performed with primers summarized in Tables S30 and S31 (Additional file 1). PCR products obtained from these assays were then investigated by sequence analysis. To perform GenomeWalker™ analysis (Clontech, Saint-Germain-en-Laye, France), genomic DNA (2.5 μg per experiment) was restriction digested and adaptors were ligated to the DNA fragments. Subsequently, PCR was performed with an adaptor-specific primer in combination with a primer located close to the breakpoint regions (Figure S27 in Additional file 1). PCRs were performed with the Advantage® 2 PCR Kit (Clontech). The corresponding PCR products were gel-purified (S.N.A.P.™ UV-Free Gel Purification Kit, Invitrogen, USA) and cloned for sequence analyses. The restriction enzymes used for each experiment, together with the region-specific primers, are listed in Tables S32 and S33 (Additional file 1).

### Analysis of the breakpoint-flanking sequences

Two datasets of sequences were analyzed in order to investigate whether non-B DNA-forming sequences, direct and inverted repeats ≥ 6 bp, or retrotransposons were overrepresented in the regions flanking the atypical *NF1* deletion breakpoints. The dataset of deletion breakpoint-flanking sequences included 150 bp located centromeric and 150 bp located telomeric to each deletion breakpoint (Table S3 in Additional file 1). The control dataset included sequences that did not flank any known atypical *NF1* deletion breakpoints; these control sequences were located within 17q11.2, telomeric to *SUZ12P* (genomic region: 29,118,000-29,148,000; hg19) and between *RAB11FIP4* and *COPRS* (30,020,000-30,050,000; hg19). In total, the control dataset encompassed 60 kb of genomic DNA subdivided into 200 fragments of 300 bp each. Within each 300 bp fragment, a hypothetical breakpoint was assigned a location between nucleotides 150 and 151 (Table S4 in Additional file 1). This control dataset, as well as the breakpoint-flanking sequences of the *NF1* deletions, were screened for the presence of non-B DNA sequence motifs (Tables S3 and S4 in Additional file 1). To this end, we used the non-B DNA database and the non-B DNA Motif Search Tool under preset search criteria for direct repeats (length: 10 to 150 bp, separated by < 10 bp) and inverted repeats (length: 6 to 150 bp, separated by <100 bp) [88]. In addition, we screened both datasets for the presence of direct and inverted repeats using the MEME suite software [89], which detects repeats irrespective of whether or not they are capable of forming non-B DNA structures. The repeats considered had a minimum length of 6 bp with no restriction being placed upon the number of nucleotides between the repeats.

Both datasets were also investigated for the presence of retrotransposons by means of the UCSC Repeat Masker track [90] (Tables S3 and S4 in Additional file 1).

In order to search for direct and inverted repeats >150 bp exhibiting ≥ 87% sequence homology within 2 kb regions flanking the deletion breakpoint regions, we performed BLASTN [91] self-alignments of the respective regions. The number of such repeats was also determined in a control dataset of sequences derived from two genomic regions: one located telomeric to *SUZ12P* (genomic position: 29,118,000-29,210,000; 92 kb), the other between *RAB11FIP4* and *COPRS* (genomic position: 30,020,000-30,048,000; 28 kb). In total, these two regions comprised 120 kb of genomic DNA, which were subdivided into 30 fragments of 4 kb each. Hypothetical breakpoints were assigned locations between nucleotides at positions 2,000 and 2,001 of each of these 4 kb fragments. BLASTN alignments of 20 kb regions flanking the deletion breakpoints were performed to determine the occurrence of direct and inverted repeats ≥ 1 kb exhibiting ≥ 87% sequence homology.

### Somatic mosaicism

Mosaicism of normal cells and cells harboring the *NF1* deletion was sought in eight NF1 patients by FISH analysis of blood lymphocytes cultivated for 72 h in the presence of phytohaemagglutinin. In each case, at least 200 interphase nuclei were analyzed. When buccal cells were available for FISH analysis, approximately 100 interphase nuclei were evaluated. Mosaicism was also investigated by microsatellite marker analysis of DNA isolated from peripheral blood samples as described previously [8] and by PCR and sequence analysis of the insertion/deletion polymorphism rs17884042.

### Availability of data

The microarray datasets are available from the Gene Expression Omnibus [92], accession number GSE57859.

## Abbreviations

bp: base pair; CGH: comparative genomic hybridization; LINE: long interspersed nuclear element; LTR: long terminal repeat; MLPA: multiplex ligation-dependent probe amplification; MMEJ: microhomology-mediated end joining; NAHR: nonallelic homologous recombination; NF1: neurofibromatosis type 1; NHEJ: non-homologous end joining; PCR: polymerase chain reaction; SINE: short interspersed nuclear element; SNC: single nucleotide change; SVA: SINE/variable number of tandem repeats/*Alu*; TPRT: target-primed reverse transcription; VNTR: variable number of tandem repeats.

## Competing interests

The authors declare that they have no competing interests.

## Authors’ contributions

JV, KB, DNC and HKS conceived the study, performed data analysis and wrote the manuscript. JV, SB and KB performed the experiments. KBMC, KW, V-FM, RM, EL, HB, MU, CL, TR and LM identified the deletions and JH performed the statistical analysis. All authors read and approved the final manuscript.

## Supplementary Material

Additional file 1 Tables S1 to S33 and Figures S1 to S27. Click here for file
