# Supplementary material for: SVA retrotransposon insertion-associated deletion represents a novel mutational mechanism underlying large genomic copy number changes with non-recurrent breakpoints
Source: Genome Biol. 2014 Jun 2;15(6):R80. doi: 10.1186/gb-2014-15-6-r80 (PMC4229983; doi:10.1186/gb-2014-15-6-r80)
Supplement: Additional file 1 — Tables S1 to S33 and Figures S1 to S27. [file gb-2014-15-6-r80-S1.pdf]

Additional file 1

Table S1: Summary of the atypical *NF1* deletions analysed in previously published studies.

Patient ID	Deletion size (Mb)	Reference
UWA 106-3	3.2 – 3.7	Dorschner et al. (2000); Kayes et al. (1992); Kayes et al. (1994); Kehrer-Sawatzki et al. (2003)
UWA 155-1	2.1 – 2.7	Dorschner et al. (2000); Kehrer-Sawatzki et al. (2003)
ID806	~7	Upadhyaya et al. (1996)
3724A	2.0 – 3.1	Cnossen et al. (1997); Kehrer-Sawatzki et al. (2003)
UWA 113-1	~1	Dorschner et al. (2000)
BUD	4.7	Jenne et al. (2000); Kehrer-Sawatzki et al. (2003)
BL	3	Riva et al. (2000)
6	3	Venturin et al. (2004a)
118	1	Venturin et al. (2004b)
442	2	Kehrer-Sawatzki et al. (2005)
806	5.5	Mantripragada et al. (2006); Pasmant et al. (2010)
T165	>2.2	Mantripragada et al. (2006)
282775	>1.33	Mantripragada et al. (2006)
T145	1.61 – 1.75	Mantripragada et al. (2006)
ASB4	1.07	Mantripragada et al. (2006)
SNF1-2	~1.3	Maertens et al. (2007)
SNF1-3	1.84 – 2.8	Maertens et al. (2007)
552	2.7	Kehrer-Sawatzki et al. (2008)
DUB	7.6	Pasmant et al. (2008)
NF00028	0.84	Pasmant et al. (2009)
NF00358	1.2	Pasmant et al. (2009)
NF00234	0.87	Pasmant et al. (2010)
NF00398	1.0	Pasmant et al. (2010)
DIE	1.2	Pasmant et al. (2010)

Table S2: Locations of the atypical *NF1* deletion breakpoints identified in previous studies. The suggested mechanism underlying the deletions was non-homologous end joining in all cases.

Patient ID	Deletion size	Centromeric deletion breakpoint			Telomeric deletion breakpoint			Reference
		located in	position	repeat	located in	position	repeat	
6	3-Mb	*TMIGD1*	28,651,627	–	*ASIC2*	31,819,849	–	Venturin et al. (2004a)
442	2-Mb	*EFCAB5*	28,284,055	L1M1	*SUZ12*	30,324,251	–	Kehrer-Sawatzki et al. (2005)
552	2.7-Mb	*NF1*	29,560,750	–	*ASIC2*	32,270,556	MIRc	Kehrer-Sawatzki et al. (2008)
DUB	7.6-Mb	*PIPOX*	27,381,233	MIRb	*GGNBP2*	34,918,232	*Alu*Sx	Pasmant et al. (2008)
NF00028	0.84-Mb	*LRRC37B2*	28,919,249	LINE	*RAB11FIP4*	29,756,041	*Alu*Sq	Pasmant et al. (2009)
NF00358	1.2-Mb	*BLMH*	28,585,504	–	*RAB11FIP4*	29,770,680	*Alu*Sx	Pasmant et al. (2009)

Table S3: Analysis of retrotransposons and non-B DNA sequence motifs located within the breakpoint-flanking sequences of the 15 atypical *NF1* deletions with simple breakpoints. The breakpoint-flanking sequences encompass 300-bp each and were downloaded from the UCSC Genome browser (hg19). Each 300-bp fragment comprises 150-bp proximal (centromeric) and 150-bp distal (telomeric) to the breakpoint which is located between the nucleotides highlighted in black. This deletion breakpoint dataset was screened for the presence of non-B DNA forming sequences using the non-B DNA database (http://nonb.abcc.ncifcrf.gov; Cer et al. 2013). Retrotransposons were identified by means of the UCSC Repeat Masker Track (http://genome.ucsc.edu/).

Patient ID	Breakpoint-flanking sequences (5'→3')	Retrotransposon at breakpoint	Non-B DNA sequence motif (length of the motif, number of nucleotides between the motifs)
61541 proximal	TTTTGCATACTTACTCTTGAAGGTGCCTCATCAGTTGCCATTTGAGCATATCTGATTCTTAAAAATTTGAATATTCAAGAACAAATTGTAAAAATATTCC<br>TTGTATAATTTTTTTGGTTTTGTAGTAATTCCTTTGTTTTCTTCACAGTACTTCTGCAGAGTGATCCATTTGTAATTAATAGACACACACACATCATCCA<br>CCATTCAGCTTTATCTTCTCATTAGAAAGTGCGTCTAACCCCTATATTTTGAGATCCTGGATTTCAGAATATTGGACTTGATGTAGACACGAGTTTCTCA	–	Cruciform (6-bp, 0-bp);<br>STR/Z-DNA (11-bp)
61541 distal	GCAATGTCCAATAGAACGTTCTTTAATGGTGGAAGTGGAAATGAAAGTTCTACTATCTGTACTGTCCAATATAGTAACCATATACCTACTGAGCACTTGA<br>AATGTGGCCAATGTGAATAAATTGAATAATTCTAATAAATTTAAATTGCTACATGTGCCTTGGAGCTACTGTTTCTAACAGTGCAACTCTAGGTGTACTA<br>GCTAGATGTGTTTTCATAACCTGCAATATTGAGAATCAGATACAGGGATGTGATGACCCCAGGTGACCACCCTTATAGCCTTGTGTTAAGGAGACTACCA	–	Cruciform (6-bp, 3-bp)
70969 proximal	AGGAATTAGGAAGTATATATATATATAAATGGAAATGTGTAGATGTGTTTTAGAGACAGGATCTCACTCTTTCGCCCAGACTAGAGGGCAGTGACAGGAT<br>CATAGCTCACTGCAGCCTTGAACTCCTTGGCTCAAGAGATCCTTCCACCTAGCCTCTGAGGTAGCTAGGACTACAGGCACATGACACCCACCACACCCAG<br>CTAATTTTTTGACATTTTGTATAGACGAGGTCTCTGTGTTGTGCAGGCTGGTCTTGAACTCTTGGCCTTAAGTGATATTCTTGCCTTGGCCTCCCAAAGT	*Alu*Jr	STR (14-bp)/<br>Cruciform (7-bp, 0-bp)
70969 distal	TAAGTATTGTGATATTTGTCAGCGAGAACAGGGAAGCCTAGACAAAGGACACTTACGACTAAGGTCTTGGCCGGGCGCGGTGGCTCACTCCTGTAATATC<br>AGCACTTTGGGAGGTCGAGGTGGGCAGATCATATGAGGTCAGGAGTTCGAGACCAACCTGGCCAACATGGTGAAACCCCGTCTCTACTAAAAATACAAAA<br>ATAAGCCGGGTGTGGTGGCACATGCCTGTAATCCCAGCTACTTGGGAGGCTGAGGCAGGAGAATTGCTTGAACACGAGAGGCGGAGGTTGCAGTGAGCTG	*Alu*Sx	–
100206 proximal	ACCCACCTTGGCCTCCTGAAGTGCTGGGATTACAGACCTGAGCCACAGCACCCGGCCTTGGTGGTGGTCTTATTGAAGGGGAAACGCTTTTTGAAATCAA<br>TAGGATGAAGGTTTTTTAAAAACAATGAAGTCTTAAATCATTAGATTTTAAAGTGGGGTCTGGAGGTGGAAGACAGCTTATTTCTGCTGTGTAACATTTA<br>GTACTTAACCATACATAATTATCAATGCAAGTTTTTTGTCCTAGGTGCAAGATTATATTTATTTGAGAGTATCAGGTGAACAAGTTTACATTTAAAGGCG	–	STR (10-bp);<br>Cruciform (6-bp, 1-bp)
100206 distal	AACATGATGAAACCCTGTCTCTACAAAAAATACAAAATTTGGCTGGGCATGGTGGCGCACGCCTGTATTTCCAGCTACTTGGGAGGCAGAGGTGGGAGGA<br>CTGCTTGAGCTGAGGGGGTGAGGGTTGCAGTGAGCTGTGATCATGCCACTGTACTCCAACCTGGGTCACAGAGCAAGACTGTGTCTCTAAATAAAGAAGG<br>CAAGAAAACAAATTGTATGAAGAAGAAAAATAATTAGTGATTTATGTATGCTTTTGTTTTATTGCTCATCCCAATATTACTGGCCCATCAACAGAGTTTA	*Alu*Jb	–
D1008345 proximal	CCATGGAGGCAGCAGTGGAACTTTATTTTTGAAAGTTTATGTAAAGGCCGGTTGCCGTAGCTCACACCTATAATCCCAGCACTTTGGGAGGCAGAAGTGG<br>GCAGATTGCTTGAACCCAGGAGTTAAGCATCCTGGGCAACATGGCGAAACCCTGTCTCTACCAAAAACACGAAAATGAGCTAGGCATGATGGCCTGTGCC<br>TGTAGTCCCAGCTACTTGGGAGGATTGCTTGAGCCCAGTAAGTCGAGGCTGCAGTGAGCCATGATTGCACCACTGAGCTCCAGCCTGTGCGACAGAGTGA	*Alu*Jb	–
D1008345 distal	ACCCTTTGTCCTTCTACATGGGAAATTAACCCTGAGTTAACATTCTAGCTATTTCCCTAGACATTTAATAAAGGTCACCTATCTTATCAAGATTGCTTTT<br>GTTAAAAATGACGCCTGATTGGTAACCACACCTGGAATAGAAGGGTCAGCAATGGTGGCAAGCAATTGCTTTGGGCTTCCTTGAACATGTGACTCCTAAA<br>ATTGAGCATGACCTTTCTAGGATCTGTAGAAGCCATGCCCATCCATGGGGCTTCTAAACCAGACTGGATCCCCTACCTACTGATATGATCTCATCTCCTC	LTR87	Cruciform (6-bp, 0-bp)
D0801587 proximal	GAGACGGAGTCTCGCTCTGTTGCCCAGGATGGAGTGCAGTGGCACAATCTCGGCTCGCTGCAACCTCTGCCTGCCCTGTTCATGCCATTCTCCTGCCTCA<br>GCCTCCCAAGTAGCTGGGACTACAGGCGCCCGCCACTATGCCTGGCTAAATTTTTGTATTTTTATTAGAGACGGGGTTTCACCGTGTTAGCCAGGATGGT<br>CTTGATCTCCTGACCTCGTGATCTGCCCGCCTTGGCCTCCCAAAGTGCTGGGATTACAGGCGTGAGCCACTGCGCCTGGTGATATTGGATTTATATATCT	*Alu*Y	–
D0801587 distal	ACTGCAAGCTCTGCCTCCCAGGTTCACGCCATTCTCCTGCCTCAGCCTCCCAAGTAGCTGGGACTACAGGCGCCCACCATCACTCCTGGCTAATTTTTTT<br>TTTGTATTTTTAGTAGAGACGGGGTTTCACCGTGTTAGCCAGGATGGTCTCGATCTCCTGACCTTGTGATCCACCCGCCTTGGCCTCCCAAAGTGCTGGG<br>ATTACAGGCGTGAGCCACCGCGCCTGGCCAGATTATTTTTAAAGGTGCTTATTATGATAGGTGGACCTCAGTTTCCTTATCTGTAAAATAGAGGTCATAC	*Alu*Y	IR (10-bp, 41-bp);<br>Polypyrimidine tract (10-bp)
1106 proximal	TGAGGAAGAAGTGCAGGAGGCCGGATGCAGTGGCTCACGCCTGTAATCCCAGCACTTTGGGAGATGGAGGTGGGAGGATCGCTTGAGGCTAGGGGTTCGA<br>GACCACATCTTTACAAAAAATTTAAAATTAGGCCAGGCACAGTTGCTGACGCCTGTAATCCCAGCACTTTGGGAAGCCAAGGCAGCAGATCACTTGAGGC<br>CAGGAGTTCGAGACCAGCCTGGCCAACATGGCAAAACCCCATCTCTACTAAAAATACAAAAATTAGCCACGAGGGGTGGTGCACACCTGTAATCCCAGCT	*Alu*Sz6	–

1106 distal	AAAGCCATGTTCTCTCTTGCCTCTGAGTGTTATCTTTCTCCTAGGATGTCCTCCCTCCCGCTTCCTTGTCAGGAGCTAACGTCTACTCACCTTTTTAAGA<br>CTTACTTGCCGGGAGTGGTGGCTCATGCCTGTAATCCCAGCACTTTGGGAGGCTGAGGTGGGAGGATTACTTGAGCCCAGGAGTTTGAGACCAGCCTGGA<br>CAATATAGTGAGACTGCTTCTCTTAAAAAAAAAATTTTTACTTAGGTACCGCCTCCTCCAGGAAGCCTCCCTGACTCCTTCTGCCCCAGCCCAAAGGAGA	FLAM_C	Polypurine tract (10-bp)
619 proximal	AAATCCAGATTGTGGGACAACTTACAAGACAACTATCTTTGACTCTTAAAAAATGCCAATGTCATGAAAGATCAAAAAAAGTAGAGGCATGTTTTAGATT<br>AAAGGAAATGAAGACATGACATGCAGTGCCTGATCTTTGACTGGATTCTGTAGTATTCTTTCATCTTTCTGCATGTTTGAATTTTTTTCAAAATATAAAT<br>TTGGGCAAAAGAGATAACCAAGATAATTGATTAATTTATTGTTATGGCTTCTTGGGGGCAGTTTCAGAGAAATAAAAACAATCTCTGTAACTGGAATAAA	L1MC4	–
619 distal	TTCTCAAATAAATATGTGGCCTGCAGGCGTACAGAGGCAAGTCTATGGTTTGGCTAGAAGGGGAACTAAATTCCAGCTCCCTCCTGCTGCTGCTCCTGCC<br>ACTGATGAAAAGTGTCTTCCCAACAGGAAGAATGAATGAGCCTTGGGGCGGTGGTAGCAGGTGTGCTGAACTCTGGCTAGGTCTCCTCAACCCCTGAAGG<br>TGAGGCCCTGCTCAAAGCTTCCTGATTCCTGTTAAGTTCAATGTTTTCTGACAGACACAAGCCAATTAGCTGTCTGCTGCTGGCGGCTGGTTTATTCAAC	–	STR (11-bp)
659 proximal	AGCGTCCCCACCCACCCCGATAAGCCTCCCACCCAAGTGTGGCTCCCTGCTGAGGGAGTACCTCAAGGGCCCGGGCCTGGGTGGCGCCAGAAGGTTTGGA<br>CACACAATTAGCCGGGGGTAGTGGCGGGCACCTTTAGTCCCAGCTACTCGGGAGGCTGAGGCAGGAGAATGGCGTTATCCCAGGGGGTGGAGCTTGCAGT<br>GAGCAGAGATCACGCCACTGCACTCCAGCCTGGGCGACACAGTGAGACTCCATCTCAAACAAAAAAAAAAAAGAGTGCTTTATAGATTCTAGATACAAGT	*Alu*Y	Cruciform (6-bp, 4-bp);<br>Polypurine tract (12-bp)
659 distal	GGCGCAGTGGCTCACACCTGTAATGCCAGTGCTTTGGGAGGCCAAGGCGGGTGGATCACCTAAGGTTGGGAGTTCAAGACCAGCCTGACCAACATGGGGG<br>AAACGGTGGTGCATGCATCCCAGCTACTCTGGAGGCTGAGGCAGGAGAATCGCTTGAACCAAGGAGGTGGAGGTTGTGGTGAGCCAAGATCATGTCATTG<br>CACTCCAGCCTGGGCGACAAGAGCGAAACTCCATCTCAAAAAACAAACAAAACAAAACTCAAGGGTCAGATAACATAGCCAGTGTAACCATAATTCAAAA	*Alu*Sp	–
R84329 proximal	GTGATCCACCCGCCTTGGCCTCCCAAAGTGCTGGGATTACAGGTGTGAGCCACCGCGCCCAGCCTTTTTTTTTTTTTTTTTTTTGAGACAGAGTCTCACAT<br>TGTCGCCCAGGCTGGAGTGTAGTGGTGCGATCTTGGCTCACTGCAACCTCCATCCCCCAGGTTCAAGAGATTGTCCTGCCGCAGCCTCCCTAGTAGCTGG<br>GATTATATGCATGCCCTACCACGTCCAACTAATTTTTATATTTTTAGTAGAGACAGGGTTTCACCATGTTGGCCAGGCTGGTGTCAAACTCCTGACCTCA	*Alu*Sz	Polypyrimidine tract (19-bp);<br>Cruciform (6-bp, 3-bp)
R84329 distal	TTACAGGTACGTGCTACCGTGCCTGGCTAATTTTTGTATTTTTAGTAGAGATGGGGTTCCACCATGTTGACCAGGCTGATCTCCAACTCCCAACCTCAAG<br>TAATCCACCCGCCTCAGCCTCCCAAAGTGCTAGGATTACAGGCATGAGCTACCGCGCTCGGCCCCTACACCTTGTTTTAATCTTTATTTAACTAATTACT<br>GATAAGGCTGATGTCTTTTCATAAGTTTAACTACAAGTGATGTTTCCCCTTTGTGACTTTCACGTTCACATGATTTGCCCATTTTTCTTTTTAGTTTTCA	*Alu*Sz	–
2535 proximal	ACTTGTCTTTCAGGTAGATGTCTCATACTTGAATGTAAACAGGTCATACTAATTTTAAAGTTGCCCAATAGAAGAATGGGCAAAGGAAACAATAAGAAGT<br>TTACCAAAATAGATATATATGGTTCTTAAACAATTAAAACGATAAAACATCTGAATTAAAAGTACACTGAGATGCGTTTTTTCACTTATCAGATTGACAG<br>AGACCAAAAAACTTGATAAAGCACTTGATCAGGCTGTGGGGGAAAGGTCTCCACATACATTACAGATAGGAGGGTGCATTGGATTGTAGGTATCAAATGT	L1Mc	–
2535 distal	CTGAATGATCAGAGAGCAGTATGGCCAGGAATGGAAGGAGTTAAGGTAGGAAGCACTCATTCTGCAATGCCTTTATCTGAGAAATTAGGGATATTTATTT<br>ACTTGCAAAAGGCTCCATCTTAGTGTTCTTTGAAGGAGGAGTCAGTAGCTTAGGCTCTTGAAAAGGTCTTTAAGACTCAGATTTCCAAACTATTTATTAT<br>AAGCCATGCTTCTCTCTGAAAAACTCTTGATGTAACAAGAGTTTCAGAATATAACAAGTTCCCCAATTCTGTTTGGGAGTTTTATGGTGAACTCAATGTC	–	–
08D2261 proximal	GGGCTGTGCGCGGTGGTTCATGCCTGTAATCCCAGCACTTTAGGAGGCTGAGGTGGGTGGATCACGAGGGAAGGGGTTTAAGACCAGCCTGGCCAAGATG<br>CTGAAACCCGTCTCTACTAAAAATACAAAAATTAGCCGGGCATGGTGGCACGCGCCTGTAATCCCAGCTACTCGGGAGGCTGAGGCAGGAGAATCGCTTG<br>AACCTGGGCAGCAGAGATTATGGTGAGTGGAGATTGCACCACTGCACTCCAGCCTGGGTTGCAACAGAGCAAGACTCCATCTCAAATAAATAAATAAATA	*Alu*Sg	STR (17-bp)
08D2261 distal	CAGATGTCTGCCACCACACCCAGCCAATTTTTTTATTTTTTGTAGAGATGGGGTCTCACTCTGTTGCCCAGGTTGGTCTCCAACTCTTGTACAACTCAAG<br>CGATCTTCCCACTTTGGCCTCCCAAAGTGCTGGGATTACAGGCATAAGCCACCACACCAGGCCTATTTTGTGCATTTTAACACAATTGTTAAAGTGGTAA<br>ATTTTATGTTATGTATATTTTATCACAATTTAAGGAAAAAAAAAAGCAGGAGAGAAGGGAAAAGAAAGAGAGACTCTTAGGCCCTGGGAAGAGGAAACAG	*Alu*Jr	Cruciform (8-bp, 4-bp);<br>Cruciform (7-bp, 4-bp);<br>Polypurine tract (10-bp)
D06.1047 proximal	GCTGGGATTACAGGCGCGCACCACCATGCCTGGCTAGTTTTTGTATTTTTAGTAGACAGGGTTCCACCATCTTGGCCAGGCTGGTCTTGAATTTCCGACC<br>TCGTGATCCACCCACCTCACCCTCCCAAAGTGCTGGGATTACAGGCGTGAGCCACCGCGCCCGGCCTATTTATTTATTTTTGAGATGGGATTTCACTCT<br>ATTGCTCAGGTTGAAGTGCAATGGTGTGATCTCGGCTCACTGCAACCTCCACCTCCCGGGTTCAAGTGATTCCCCTGCCTCAGCCTCCCCAGTAGCTGGG	*Alu*Sg7	STR (13-bp)
D06.1047 distal	CACACATGCGGCACCACTCTCAGTTAACGTTTGTATTTTTTGTAGAGACGGGCTTTCACCATGTTGCCCAGGCTTGTCTCGAACTCCTGAGCTCAAGCGA<br>TCCGCCCCCCTCAGCCTCCCAAAGTGCTGGAATTACAGGCGTGGGCCACCCTGCTGGCCCCTAGTACATTCTTTTTAGGATTTTGTACCAGAAAAGAAGT<br>CCTTCCTGATGAAATGATACCTTTTTACATCAGATAACAAGAGTTACAGCATAACTATCCCCTTGCTGTCCTTTTTGGTTTTGGTAAGTCAGAAAACTTA	*Alu*Jb	–
D05.2678 proximal	GGCCACCACACCCAGCTAACTTTTGTATTTTTAGCAGAGATGGGGTTTCACCATGTTGGCCAGGATGGTCTCAACCTCCTGACCTCGTGATCCGCCCGCC<br>TTGGCCTCCCAAAATGCTGGGATTACAGGTGTTAGCCACCGTGCCTGGCCCCTATTTTTTGTATTTTTAGTAGAGACGGGGTTTTGCCATGTTGGCCAG<br>GCTGGTCTTGAACTCCTGACCTCAGCTGATCTGCCTGCCTCGGCCTCCCGAAGTGCTGAGATTACAGGCATGAGCCACTGTGCCCAGCCTGTTCCTTTTC	*Alu*Sx	–
D05.2678 distal	GAGCTGAATGGAGAGGAAGCTGGAAGGCTGTGCCCCTTAGGTGACTCACCCTACTCTGGCCTAAAAGCTTGTGCCAACATGCCTTACCCCCTTGGTTAGG<br>GCTGGGACCACTTTCCTGGGGAGTCACTTCTGAAAGTCCCTGGGAGTGGCTGTGGAGTCTGGGTAAAATGGAACTAGTTCTCCTGGGACCCAGCCAGCTT<br>GGTGGAATCACACAGTGCTAGGGATTCTAGGCCTGCATCAGTCCTGACTTGTCCTGGGAGGGTGCATATAGCCTAGCCTATTTAGTGCCTGAGGGAAGGG	–	–

R48018 proximal	GGGTGACAGAGCGAGACTCCGTCTCAAAAAAAAAAATTCTTTCTCACAAAGAAACACTAGTATAATAATGGCTGGTTGGCTGGGCCCATTACTCATACCT<br>GTAATCTCAGCACTGGGAGGCCAGGGCGGGTGGATCACCTGAGGTCAGGAGTTCTAGACCAGCCTGGTCAATGTGGTGAAACCTCATCTTTACTAAAAAT<br>AGGAAAATTAACCTGGCATGGTGGCGGGCACCTGTAATCCCTGCTACTCAGAGACTGAGGCAGGAGAATCACTTGAACCCAGGTGGGAGGTTGCAGTGAG	*Alu*Sx1	Polypurine tract (11-bp);<br>Cruciform (6-bp, 1-bp)
R48018 distal	TAGTATACTATGCAACTATTAAAGAAAGTAAGGAGTGTGTGTATCTAATGGGATGGAAATAGGCTTAATTTACATTGCTTAATTTTAGAAAGGCAAGTTG<br>CAGAACAGTATATTGAATAATCCATTTTAAATATGTGTATGTGTATGTGTCATAAAAAAAAAAAGAAAGAAAAAGCAGCCAGGCACTGTGGCTCAAGCCT<br>GTAATCCTAGCACTTTGGGAGGCTTAGTGGGGTGGATCACCAGGTCAGGAGTTCAAGACCAGCCTAGCCAAGATGGTGAAACCCCTCTCTACTAAAAATA	L1MC4A	STR (19-bp);<br>Polypurine tract (11-bp)
Ak-47055 proximal	TACATTTCTTTTTGCATACTTACTCTTGAAGGTGCCTCATCAGTTGCCATTTGAGCATATCTGATTCTTAAAAATTTGAATATTCAAGAACAAATTGTAA<br>AAATATTCCTTGTATAATTTTTTTGGTTTTGTAGTAATTCCTTTGTTTTCTTCACAGTACTTCTGCAGAGTGATCCATTTGTAATTAATAGACACACACA<br>CATCATCCACCATTCAGCTTTATCTTCTCATTAGAAAGTGCGTCTAACCCCTATATTTTGAGATCCTGGATTTCAGAATATTGGACTTGATGTAGACACG	–	Cruciform (6-bp, 0-bp);<br>STR/Z-DNA (11-bp)
Ak-47055 distal	TGTTCACACAAATAGTTCTATTATTGCTTTCCTTTACAGCCTTGGTCTGCTCAAAAACCCACATTCAAATTGGATCACATAAACTCCAGCCCTTAAGCAC<br>ACAGGTATTGTAGGCTAGAGGCCGCTTTTGGCTACAAACCCCCTTATTCATTTAAAATAACTTCATTCACAAGGTTCATTGAACAGCTGGTTTGTGCCCA<br>TCACAGGCCCTGACAAAACACTTCGCCTAACTTGTCACTAATCCTTACAGCAACTCTGGTTGAGTGTTACTAGTATCATTTAACAGATAAGCAAACCAAG	–	–

STR (yellow): short tandem repeats of 2–6-bp that are repeated several times. A subtype of STR may also represent Z-DNA. Z-DNA is characterized by five or more tandem repeats, each comprising an alternating pyrimidine–purine dinucleotide motif (Wang et al., 1981; Cer et al., 2011).
Polypurine tract (yellow): poly-A or poly-G tracts.
Polypyrimidine tract (yellow): poly-C or poly-T tracts.
IR (green): inverted repeat of ≥6-bp separated by ≤100-bp.
Cruciform (green): A subtype of IR of ≥6-bp in length separated by 0–4-bp.

Table S4: Retrotransposons and non-B DNA sequence motifs identified within the breakpoint-flanking sequences of the control dataset which includes sequences that do not contain breakpoints of known atypical *NF1* deletions. The control sequence dataset comprises two 30-kb regions, one located distal to *SUZ12P* (29,118,000-29,148,000; hg19) and the other located between *RAB11FIP4* and *COPRS* (30,020,000-30,050,000; hg19). These 30-kb regions were subdivided into 200 fragments of 300-bp each which are listed in the second column. Hypothetical breakpoints were assigned locations between nucleotide positions 150 and 151 highlighted in black. This control dataset was screened for the presence of non-B DNA forming sequences using the non-B DNA database (http://nonb.abcc.ncifcrf.gov; Cer et al. 2013). Retrotransposons were identified by means of the UCSC Repeat Masker Track (http://genome.ucsc.edu/).

ID	Breakpoint-flanking sequences (5'→3')	Retrotrans-poson at breakpoint	Non-B DNA sequence motif (length of the motif, number of nucleotides between the motifs)
1	GATCTCAGCTCACTGTAACCTCTGCCTCCCAGGCTCAAGCAATTCTCATGCCTCAGCCTCCTGAGTGGCTGGGATTATAGGTGTGTGCCACCACACCCTG<br>CTAATTTTTTTTTTCTTTTTGAGACGGGTCTTGCCCTGTTGCCAGGCTGAAGTGCAATGGCTCACTGCAACATTTGCCTCCCGGGTTCAAGTGATTCTCC<br>TGCCTCACTCTCCCTAGTTGCTGGGATTACAGGTGTGCACTACCACATCCAGCTAATTTTTGTATTTTTAGTAAAGATGGGGTTTCACCATGTTGGCCAG	*Alu*Sc	Polypyrimidine tract (10-bp)
2	GGGTGGTGTCTTGATCTCCTAACCTTGTGATCCACCCACCTCAGCCTCCCGAAGTGCTGGGATTACAGGCGGGAGCCATTGCGCCTGTCCTAATATTTGT<br>ATTTTTAGTAAAGATGGGGTTTTACCAGGTTGACCAGGCTGGTCTCGAACTCCTGACCACAAGTGATCCAACCGCCTCGGCCTCCCAAAGTGCTGGGATT<br>ACAAGTGTGAGCCACTGCGTCCAGTCTCTTGGCCTCTAAAATTTTTTTTTTTTTTGAAACGGAGTCTTGCTGTGTCGCCCAGGCTGGAGTGCAGTGGCGT	*Alu*Sx	Cruciform (6-bp, 1-bp);<br>Polypyrimidine tract (14-bp)
3	GATCTCGGCTCACTGCAAGCTCCGCCTCCCGGGTTCACGCCATTCTCCTGCCTCAGCCTCCCGAGTAGCTGGGACCACAGACACCCACCACCATGTCCGG<br>CTAATTTTTTTATTTATAGTAGAGATGGGGTTTCACCATGTTAGCCAGGATGTTCTCAATCTCCTGACCTCGTGATCCGCCCGCCTCAGCCTCCCAAAGT<br>GCTGGGATTACAAGTGTGAGCCACCGTGCCTGGCCAGGCCTCTAAATTTTTAAGATTTACTTTTTAATTTTGTGTGTGTGTTTGAGACACAGTTTCGCTC	*Alu*Sy	Cruciform (6-bp, 0-bp);<br>STR (11-bp)/Z-DNA (11-bp)
4	TTGTTGCCCAGGCTGGAGTGCAATGGCATGATCTCAGCTCACTGCAACCTCTGCCTCTTGGGTTCAAGCAATTCTCCTGCCTCAGCCTCCCAAGTAGCTG<br>GGATTGCAGGCATATGTGCCACCATGCCCGGCTGATTTTGTATTTTTAGTAGAGATGGGGTTTCTCCATGTTGCTCAGGTTGGTCTCGAACTCCCGACCT<br>CAGGTGATCCACCTGCCTCGGCCTACCAAAGTGCTGGGGTTACAGGTGTGAACCACTATGCCCAGCCTAAGATTGACTTTTGAGAGATACTCTACTTACC	*Alu*Sp	Cruciform (6-bp, 3-bp)
5	TCTACTTACCAAGAGGAAAAGAGATATAAACATATTAAATAACAAGCATTTTATATACATTTCCCAGTTAATCTACGTAATAACCCTGTGAATACAGATG<br>AGAAAACTACAGCTACAGGAAATAAAGAAGCCTTCCCACAGTAACACTGCCTATAAATGACGGAATAAACTCAGGATGTTTTGAATTCAAAGCTTATGTA<br>TGCTCTATGCACTTCACTCTGCTTCCTGAGGCAGGTGAGATGTTTTAATATCTACTTGCCTGAATGTTAATGTCTGCCCACAGAAATAAGTCGGAGCTCT	MirB	Cruciform (6-bp, 0-bp)
6	GGAGTAGAGAACACCCGATGATTCAGAATCGTTCCGAAGTGCTATATTTCTTCGACTGCTCAGACTGTACCCCTCAAAACCAGCTGTCCACTCTTTTTCA<br>AAGCAAAGATATTGAAAACTGTTAGAATCTCTTGAGGGATATCTTCAATACAGACACGTTAAATAAAACATGGAAATGGGAGACTACTCTTAGGGGCCAT<br>GGTCAAGTTCAATAAAATAAAAGATTCCATTAACAGGGTATCATGATCAGTCCCACAAATTACAAAAGATTTTAGTATATTCCTGCATAATTTTGACATA	–	A-phased repeat (24-bp)
7	ATAAATCACACAAGTGTTATCTCTTATCTAATAAAATCATTGTACAAGCATAATTGCTTGAACTTATTCCAACCTGATAAAAATCTAATTAATAGGCCAG<br>GCGCGGTGGCTCACGCCTGTAATCCCAGCACTTTGGGAGGCCGAGGCAAGAGGATTGCTTGAGCCCAGGAATTTGAGACCAGCCTGGGCAACATAGCAAG<br>ACCCAGTCTCTAGAAAAAAATAAAAAATTAGCCAGGTGTGGTGTGCATGCAGGTGGTCCCAGCTACTTGGGAGACTGAGGCAGAAGGATCTAGAGGTCAG	*Alu*Jo	Cruciform (6-bp, 4-bp)
8	GCTGCATGATTGCACCACCACACACCAGCCTGGGCGACAGAGTGAGACCTTGTCTCAAAAAAACTAACTAAATAACATTCAAATATAAAAAAAAGAATAA<br>AAAGGAATATCAGTGCCCTACATAATATTTTCAATCTTTGGTTTTCAATGAGGCTAAAAATCTTGTCCAGCATTTTTTTGGACTCTGAAGAACTGATGA<br>AAACAAAAATCACATGAGGTAATCTGAGTAAAAGACATGGTGCATGTTGTTCTTATCAATTCAGGATCTATGCTTAGAATAGCTGGTTGTCAGTGTGCTG	–	Cruciform (6-bp, 3-bp)
9	CCCTTAGCACAGAATACAGGTATTCTAATATATAGTTAGGCTTCAGGAGAGAAAAGGTTAGCAGCATACCATGAGGCACCAATGTGGAATGACCTATCTG<br>GGGGACACTCCAAGGACTCCACTCCTGTCGGCCATCTCCTCGGGCGCAGACTCTGAACTGGTAATCAACGTTGGGGTCTATGTGCAATACTATGAATTCA<br>GTTTCAGAACCTACATATACATCCTCAAAATGATTTGAAGTACATTTACGAAACTGGAGCCTGTAATCTTGGGCTGTAAAGTCATCATCCACCTAGGGAG	–	Cruciform (7-bp, 2-bp)
10	AAAAGCTTTATGTTAGGTAAAAAACAGAAAATGACTTCTCTCAGCATCACTGCTTAAAAATTTTGAACGGAGCCATTTTGGACCAAGCAGACAGGCCCAC<br>ATGCTGAATATGGGGTATTCTTGGGAAGGCCATAGAAAATATGCCATCACATCATTATCTCCCTTCTTTTTTTTTTGAGAAAGGGTCTCACTTTGTCACC<br>CAGGCTGTAGTGCAGTGGCGTGATCTCGGCTTATTGCAGCCTCCACACCCCGGGTTCAAGCCATCCTCTTCCTTCCACCCCACAAGTAGCTGGGATTTCA	–	Polypyrimidine tract (10-bp)

11	GGTGCACACCACCATGTCCGGCTAATTTTTTGTATTTTTTGTAGAGACTGGGCTTCACCATATCTCAGATCTTTAACTCCTGAGCTCAAGCAATCTTCTC<br>GTCTCAGCCTCTCAAAGTGCTAGGACTATAGGCGTGAGACACTGCGCCTGGGCTCCCTTCTTTTCTTTATTGAACCATGTGTTCTGTATTTCCCAGTCTG<br>ATATAAAAAAAGTCTGCCTCCTTTGAAGAAATGGCATGTTTTTTAATCAATGCCAAAGATAAAACATTCAAGATTCAGTAAGTCTCCCACAACCATACATC	*Alu*Jb	–
12	ATTCATAATAACCAAGAGATAATTAACATACAGTATACAGAACCCCCATTAATTTTTAGGGGTCCTGCATATATTCTGTATGTTAATTAGCCACCCAATA<br>GAAAAATGGGTAAAAGACATAAATAAGCGTTCTACAGGAGAAGAAACATAAATAGCCAAAAATGTATATTATGAATATTCAACCTAATTTGTACCTAATT<br>TGTAATATGGCAAATACGATCTAAAACCACAACAAGGCCAGACATGATGGCTCATGCCTATAATCCTAACACTTTGAGAGGCCGAAGCGGGTGGAGCACT	L1M5	IR (14-bp, 42-bp);<br>Slipped motif (11-bp, 0-bp)
13	TGAGCCCAGGAATTTGAGGCCAGCCTAGGCAACACAGCAAAACCCCATCTCTACAAAAAATTACAAAACTTAGCTGGCTGTGGAAGCACATGCCTGAAGT<br>CCCAATTACTTGGGGGCTGAGGCGGGAGGATCACTTGAGCCCAGGAGATTGAGACTGGAGACATGATTGTGCCACTGTACTCCAGCCTGGGTGACAGAGT<br>GAGACCCTGTCTCAAAAACAAACAAATAAATAAAACCACAACAAGGTACATTTAGGCAAAAATATAAAGTCTCACAATGCCAATTATTGGTCAAGATATG	*Alu*Jo	Cruciform (6-bp, 3-bp)
14	GAATAACAGAAACTCATATACTCCTTGGAAGGCTAAATTGATAGAATCATCTTGTGGAAAGTTTGTTATTACCTATCAGCGTATGGATCCAATACTCTAA<br>GAAGACCTCATAATTTTACACCTAGGTAAATATTAATATTTTACAAACTTCACATATGTCCATAAAGAAACACAAGTAAAAATACTTATAGCAAAAAACT<br>GGAAATAACTAGGTCCACTGATAGGAAAAATAATAAAAATGGAATGACAGTCCTGTTAGAAAATAACAACAGCATGTTTATAAAAAAAGAAAAAAAAATT	L1M5	Cruciform (7-bp, 0-bp)
15	TCCTTTTAATAAAATCTCAGCTGGGCACAGTGGCTCACACCTGTAATCCCAGCACTTTGGGAGGCCGAGGTGGGCAGATCACCTGAGGTCGGGAGTTTGA<br>GACCAACCTGACCAACATGGAGAAACTCTGTCTCTACTAAAAATACAAAAATACTAAAAATACAAACTTAGCCAGGTGTGGTGGCGCATGCCTTAATCCC<br>AGCTACTTGGGAGGCTGAGGCAGGAGGATTGCTTGAACCCGGGAGACGGAGGTTGTGGTGAGCCAAGATCGTGCCATTGTATTCCAGCCTGAACAACAAC	*Alu*Sp	DR (15-bp, 2-bp)
16	AACGAAACTCTGTCTCAAAAAAAAAAAAAAAGAAAATGTCAGCCAGGCGGAGTGGCTCACACCTGTAATCCCAGCACTTTGGGAGGCCAAGGTGGGCAGA<br>TCACCTGAGGTCGGAGGTTGCATCGCTGCACTCCAGCCTGGGCGACACAGCAAGGCTTCATTTAAAAAAAAAAGGCCAGGCACGGCGGCTCACACCTATA<br>ATCCCAGCACTTTGGGTGGCCGAGGCGAGTGGGTCACCTGAGGTCAGGAGTTCAAGAACAGCCTGGCCAACATGGTGAAACCTCGTCTCTACAAAAAATA	FLAM_C	Polypurine tract (15-bp);<br>Polypurine tract (10-bp)
17	CAAAAATTAGGCAGGTGTGATGGCAGGTGCCTATAATCCCAGCTACTCAGGAGGCCGAAGCAGGAGAATGGCTTGAACCCGGGGGAAGCGGCAGGCGGGG<br>GAGGGCATGGAGGTTGCAGTAAGCCGAGATCACGCCACTTCACTCCAGCCTCGGTGAAAGAGCAAAACTCTGTCTCAAAAAATAAATAAATAAAATAAAA<br>ATAAAACTGGAAGAGCTACCTCAGTGTTCCATGTGACTTCATTTTAACTGTAAAGTAAAAGAGATTTTTGTTTGAGAAGAAAAGAATGTATTTAAAAGTA	*Alu*Sx1	STR (15-bp)
18	GTCAACGAAAAAATCTGAAAGGCCTCCAAATCGCAAACCCATAATGTGGAAGTCATTTCTAGAATTCTGAATTACATACCTTACACCATCGTACAATGAT<br>GCCTCCAGGTTTCTCTATTAGTTCTTCTATCTGTACTGGTGGGCGAGATGCTACTGTTCCATGCTTAAAAATGTGGTCTTTCACTATGTTAAGAATTGAG<br>TCATCCAACTGAGCAGATAAACAAGGCACATCAACCAGTAAAGGTACTTCTGGTAAGCTGAGGAAACAAAGCTCATGTGTTATGAGACCTCTAACTGAGA	–	–
19	GTTAAAAAATACAAGAAATATTTTAGAGCAGTCACATGCTTGAAAGATCCTGAAGCCCCCAGTAATTAATTCTCATGGCAGTAGGGGATTTGCAAGATGT<br>TACACAAAGATTCTCTTTTCAAATAAATATAAGTAAGACAGAAAATTGGTAGGATCATAACTGTCCAGTTTAAGCCAAAAGATCTGGTTTACTGGAATAT<br>GACTCCATTTCAACAATACATTGATAATTTCTAAAATTTAACAATTCTTTTATATATTGGATTGAAAAAATGTGTAGTAATACTATACCAAGGATTATAA	–	–
20	TCACAACTATTTCATTAGTAACAGTGGTAATGGGCAGTGAAACGCTTTTTTTTTTTTTTTTCAGACAGAGTCTCACTCTGTCCCCTAGGCTGGAGTGCTG<br>TGGTGCGATCTCAGCTCACTGCAACCTCCACCTCCCCGATTCAAGCGACTCTCTTGCCTCAGCCTCTCGAGTAGCTGGGACTTACAGGCGCACGCCACCA<br>TGCCCGGATAATTTTTTATTTTATTTTATACTTATTTTGAGATGGAGTCTTGCTGTTGCCCAGGCTGGAGTGTAGTGGTACAAACTTGGCTCACTGCAAC	*Alu*Sz6	Polypyrimidine tract (16-bp);<br>Cruciform (8-bp, 3-bp);<br>STR (16-bp)
21	ATCTGCCTCCTGGGTTCAAGCAATTCTCCTGTCTCAGCCTCCCCAAGTAGCTGGATTACAGGTCCATGCCCGTCTAATTTTTATATTTTTAGTAGAGACA<br>GGGTTTCAACATGTTGGCCAGGCTGGTCTCAAACTCCTGGCCTCAAGCAATCCACCCGCCTTGGCCTCCCAACGTGCTGGGATTACAGGTGTAAACCACC<br>ACGCATGGTCTGAAAACCTTGAAATATATATTCTCTATCTCCCTTAATGAGAATCTTGAACAGAGGGAGGAAAAAAGCAGACATAGTTTAAATGCTTAAA	*Alu*Sz	–
22	AGTAAGTCAAAAAGGCACAAACTCTTTTACCTGTCCAACTGAATGTGCGAGGCCTTTTTGGTAAAGCTCCACAGTTTCTCATTCTCTTCTCCCACACCAC<br>CAAGCATGGCGATTTCACCTACAATCAAGAATAAGAGAACTAGAACAATGAAAATGGTCACATATAAAGTAATCAACACAACTCATGTCTGGGTGGGTCT<br>CTAGCGTTCTTGCAAATAAGTTCTTAAGTGATGCCACAGTCAAATTCCCAAAAAGTTAAAGTAGAAACTATTTTAGACGTGACTCTAAGATCAAATCCAA	–	–
23	GAGGAAAAGGGATATACAGTGGCAACATTTTGCCAGAGATGAAAAATCTATCTACTCTATACAAGGAAATCAACCAATTTTAGGCAAAGCAGAAAGCCCA<br>GGGACTTCTTTCCATCTCTCCTACAGAATCATTATGAACTTCAATAGGAGCCAGTACAAAATATTGCCCTGAGAGGTGCAACAGGGGATAGGGTGTTTTT<br>TTTTTTTTTTTTGAGACTGGGTCTTGTTCTGTCACTCATGCTGGAGCACAGTGGTAGGATCATAACTCACTGCAGCCTTGAACTTCCAGGTTCAAGCAAT	–	Cruciform (6-bp, 4-bp);<br>Polypyrimidine tract (17-bp)
24	CCTCCCATCTCAGCCTCCCAAGTAGCTGTGACCACACGTGCACGCCACCATGCCCAGCTATTTTTTTTTTTTTTTTTTTGGTAGAGATGAGGTCTCACTA<br>TGTTGCCCAGGCTGGGCATAAGGCTTGAAAAGCCAAATGATGCATCTATGTTTTCCTAAAAGAGTATCATATATCAGATTACAAATTATTCAACAGAACA<br>ATAAAAAGAAAATGTCAGGCTGGGTGCGGTAGCTCACGCCTGTAATCCCAGCACTTTGGGAGGCGGAGGCAGGTGGATCACGAGGTCAGGAGATCAAGAC	–	Z-DNA (13-bp);<br>Polypyrimidine tract (19-bp);<br>Cruciform (7-bp, 1-bp)
25	CATCCTGGCCAACATGGTGAAACCCTGTCTCTACTAAAAATACAAAAATTAGTTGGGTGTGGTGGCAGGTGCCTGTATTCCCAGCTACTAGGGAGGCTGA<br>GGCAGGAGAATCACTTGAATCCGGGACATGGAGGTTGCAGTGAGCCGAGATCACGCCACTGCACTCCAGCCTGGGCAAAAGAGCGAGACTCCGTCCCCCC<br>CGCCAAAAAAAAAAGTTACATAGAGGCATATGATATGAAAATGACCAACTTCTATGTCCTTCCACAGCCCTGAAGAACACTGCCAGAACTCTTTTCCATC	*Alu*Sc8	Polypurine tract (10-bp)

26	TAAATGTGCCCTGTAGTTACTCAAGTTTTTTGAGACCATATGACCTTCTCCTTCACCAAAAGAAAAACAGAGACATGGCCGGGTACTGTGGCTCATGCCT<br>ATAATCTCAGCACTTTGGCAGGCTGAGGCTGGTGGGTCACCTGGGGTTGGGAGTTCAAGACTAGCTTGGCCAACATGATGAAACCCCGTCTCTACTAAAA<br>ATACAAAAAGTAGCTGGGCATAGTGGCACGCTCCTGTAGTTCTAGCTACTAGGGAGGCTGAGGCACTAGAATCGCTTGAACCCGAGAGGTGGAGGTTGCA	*Alu*Sx	–
27	GTGAGCCAAGATCGTGCCACTGCACTCCAGCCTGGGCAACAGTGTGAGACTCTGTCTCAAAAATAAGTAAATAAATAAATAAAATCCTCAAAATTCCATA<br>AGTAAGTTTCTATCCCTCAGGAGAATAGGAAAGAGTTTGACATTAAAAATATTCCTAAAGTTTCCTTCTGCTGCTGAGATGTATAATAATTTTTTTTAAT<br>TAAAAAACATAGGCCAGGCACAGTGGCTCACGCCTGTAATCCCAGCACTTTGGGAGGCCGAGGTGGGCAAATCACGAGGTCAGGAGTGCTAGACCAGCCT	–	Cruciform (6-bp, 3-bp);<br>STR (16-bp);<br>Cruciform (8-bp, 0-bp)
28	GGCCAACATGGTGAAACTCTGTCTCTATTAAAAATACAAAAAATTTGCTGGGCGTGGTGGCGGGCGCCTATAATCCCAGCTACTCGGGAGGCTGAGGCAG<br>GAAAATCGCTTGAACCTGCGAGGCGTAGGCTGCAGCAAGCTGAGATCGTGCCACTGCACTCCAGCCCAGGCAACAGTGCAACACGCCATCTCAAAAATAA<br>ATAAGTTAATTAATTAAATTAAATTTTGGACATACAAAAATATTCAAGTATCAAATGCTGTAACACTCACACTGCCTCAAAGGGATTTTTTCTTTTTGAG	*Alu*Sx3	Cruciform (6-bp, 0-bp)/STR (12-bp);<br>Cruciform (6-bp, 1-bp) (underlined)
29	ACAGAGTCTCACTCTGTCACCCAGGCTGGAGTGTGATGTATAATCACAGCTCACTGCAACCTCAAACTCTGGGGCTCAGGGGATCCTCCCGCCTCAGCCT<br>CCCAAGTAGCTAGGACTACAGGAATGTGCCACTATGCCCAGCCAGGATTTATCTTTTTTTTTTTTTGAGATGGAGTCTTGCTCTTGTCGCCCAGACTGGA<br>GTGCAATGGTGCAATCTCAGCTCACTGCAACCTCTTGCCTCAGCCTCCCGAGTAGCTAGGATTACAGGTGCCCACCACCATGCCCAGCTAATTTTTGTAT	*Alu*Sq2	Cruciform (7-bp, 3-bp);<br>Polypyrimidine tract (13-bp)
30	TTTTAGTAGAGACGGGGTTTCACCATGTTGGCCAGGCTGGTCTTGAACTCCTGACCTCAGATGATCCGCCCCCCTCGGCCTCCCAAAGTGTTGTGATTAC<br>AGGCACGAGCCACCGCGCCCGGCCAGGATTTATCTTCTAAAATAAAAACAAATGATCAGTCCTCAGGGTGATGAGTGTACACTCCTGGCCCGTGGTGCAC<br>TGCTTCTGGGTTTGTTTCCAAATGCAAAGCCACCAAGGAAAGACATTTCATGGTACTGCTAGAATTTTCTCAAAAGTACTGTTATGGTCAGTGGCAAATA	–	Cruciform (6-bp, 0-bp)
31	AACACTTCTCATTGAAGACAAGACAAAAATGTAACCAGCAATGTCAAGATAACACCCATACTATATTCTAAGTGGGACCAAAAGAAGATTAACTTCCAGG<br>TGGAGTGACAGCCATGTGAATCATCCACGCCTTTTTCCCACACCACTCCCAGCACTCATTCTGCAGAAGAGATGGAATTCAGTCTCCTTCCCTAGCTCCT<br>TTTTCCTCAAATGACTTTTAAATGTTGGTGACCCTTAGCGTTCTAATCTCTTTCATCTGCTCTTCTCACATTACACGGTGTCCCTTGATGATTGTACTTA	–	–
32	TTCCAATAGATTCAGATTTGATATATATGCCATTAACTGCCATACTGACATCTCCTGGTATGATCTCTCTCCTAAGAATCATACCCGTATTTCCATCTTA<br>AGCACCTCAAACTCACCATGCCCAAGCCAAATGCCTAACTACGTACTAAGCACCCGCCTTCTCCTTCAATTCCTTTGTCAAAAACAGCACCACTATTTAT<br>TCAGAATCCAAAAGTAGGCTGATTTTTGTTATCACAAATCCTTTCCTTTCCCCTCCATCACATATGTAGGTAATCATCTACAGATAACAGTCACTGCTCA	–	–
33	GTAAAATCATCAATAATTCTACTTCTTAAAAAAAAAAAAAACTCTGTCTTGTTTTTTTGTTTTTATTTTTAAGAGACAGGGTCTTGCTCTGTTGCCCAGG<br>CTGGAGTGCAGTGCTGCAATCATAGCTCAAGCTCTCCCACCTCAGCCTCCTGAGTAGTTGGGACAACTTGTGTGTGCCACCATGCCTGGCTAATTTTTTT<br>TTTTTTTTTTTTGAGACGGAGTCTCGCTCTGTCGCCCAGGCTGGAGTGCCATAGCACGATCTAGGCTCACTGCAAGCTCCGCCTCCTGGGTTCACGCCAT	*Alu*Jb	Polypurine tract (14-bp);<br>Polypyrimidine tract (19-bp)
34	TATCGTGCCTCAGCCTCCCGAGTAGCTGGGACTACAGGCGCCTGCCACCATGCCTGGCTAACTTTTTGTATTTTAAGTAGAGATGGGGTTTCACTGTGTT<br>AGCCAGGATGGTCTTGATCTCTTGACCTTGTGATATGCCTGCCTCGGCCTCCCAAAGTGCTGGGATTACAGGTGTGAGCCACTGTGCTCAGCACGCCTAG<br>CTATTTTTTTTTAAAAAAATTAGAGACAATCTCACTACATTGCCCAGACTGGTCTCGAACTCCTTACCTCAAATGATCCTCCTCTCCTGTCTAGGCTCCC	*Alu*Y	IR (10-bp, 35-bp);<br>Cruciform (8-bp, 2-bp)
35	AAAGTGCTGGGATTACAGGTGTGAGCCATTGTGCCTGGCTCAAAATCTCTTAAAATCAGCCCTGCCTCATCTTTACCAGCCACCGCCTTTACCATTCCTA<br>CCTGGATTACTTTCTAATAACTTTTTTTTTGAGATGGGGTCTCATTATCTTGCCCAGGCTGGTCTTGAACTCCTGGGCTCAAGTGATCCACCCGCCTCAG<br>CCTCCCAAAGTGCTGGGATTACAAGCATGAACCACCGTGCCCAGTTTCTAATAATTTTTTTTTATTTTGAGACGGAGTCTCACTCTGTTGCCCAGGCTGG	FLAM_C	Cruciform (6-bp, 3-bp)
36	AGTGTAGTGTTGTGATCTTGGCTGACTGCAACCTTTGTCTCCCAGGTTCAAGTGATTCTCCTGCCTCAGCCTCCCGAGTAACTGGAATTACAGGTGCCCA<br>CCACCACGTCTGGCTAAATTTTGTATTTTTAGTAGAGATGGGGTTTTACCATGTTGGCCAGGTTAGTCTCAAACTCCTGACCTCAAGTGATCCTCCCATC<br>TTGACATCCCAAAGTGCTGGGGTTATAGGTGTTAGCCTGGCCTCGGCCTCTAATAACTTTTTAATCAGTCTCTTTTCCCTCTAGTCTTACAATCTCTAAC	*Alu*Sx1	STR (10-bp)
37	ATAGTCTCCAAATAGGCAGGGTCATTTTTTTTTTAAATGCACATCTGATCCTGTCACTCTGCTGCTTAAAATGCTTCAACTAATCCTTGAATATGCCATT<br>CACTCCACTTATGATAATGTTACTTCTGCTTGGAATCATGCTGTTACCTCCTCTTTTTCCTATGTCTGCTGGATGAATTGCTTCAAGCCTCTGTGGAACT<br>CTTCCTACAGAAAGCTATCCCACACTATTCCAGACAAAGTTAAGAACTTCCTCCTCTATGCTCCAATAAGGTCTTGATCTCTTAGCATGTATCTCTCTCT	L2c	Polypyrimidine tract (10-bp)
38	CACTGGGCTCTGAATCCCAAAGGAAAGAACTCTCACCTATTTTATTCTTTTCTTTTTTTTGAGAGGGAGTCTCACTCTGTCACCCAGGCTGGAGTGCAGT<br>GGTGCAATCTCGGCTAACTGCAACCTCCGCCTCCCGGGTTCAAGCAATTCTCCTGCCTCAGCCTCCCGAGTAGCTAGGACTACAGGCATGCGCTGCCACG<br>CCCGGCTATTTTTGTATTTTTAGTAGGGACGGGGTTTCACCATGTTGGCCAGGATGGTCTTGATCTCCTGACCTGATGATCCGCCCACCTCCACCTCCCA	*Alu*Sc8	–
39	AAGTGCTGGGATTACAGGCGTGAGCCACCGTGCCCGGCCTTTATTCTTAGTCTTATTTTATATTTTTTGAGACAGGGTCTTACTCTGTCACCCAGGCTGG<br>AGTGCAGTGGTGCAATCACGGCTCACTGCAGTGTCAATCTCCTGGCCTCAAGGGATCCTCCCGCCTCGGCCTCTTAAGTAGTTGGGACTCCAGGTGCATG<br>CCACCATGCCCAGCTAAGTTTTTTTTTTTTTGTAGCGACAGGCTGTTGCCCAGGCTGGTCTTAAACTCCTGACCTCAAGTGATCCACTTGCTTCAGCCTC	*Alu*Jb	Polypyrimidine tract (13-bp);<br>Cruciform (6-bp, 3-bp)
40	CCAAAGTGCTGGGATTACAGGCGTGAGCCACCACACCTGGCCCCCTTGTTTATGTTATGTGCCTGACAAAGATCTCTGTTGAAGGAAGGTGGTATAATGA<br>AGAAAGGAAGGCAGCAGGGCATGACTCAATGGAAATAATTGGCAAAGATAGGAGTAAACACCAATCACATTTTTTTAAACTTTCTAAATAAAACAATGGA<br>GACTGTAATTGTGGTGGCTTTTGCCTGTAATCCCAGCACTTTGGGAGGCCAAGATGAGAAGATTGCTTGAGACCAGGAATTCGAGACCAGCCTGGGCAAC	–	–
41	ATGGTGAAACCCTGTCTCTACTAAAAATACTAAAAAATTACAGGCATGGTGGACGCGCCTGTAATCCCAGCTATTTGGGAGGCTGAGGTGGGAGGATCA<br>CCTGAGCCAGGGAGGTCAAGACTGCAGTGAGCTCTGACAGCACCACTGCACTCCAGCCTGGGCAACAGAGCAAAACCCCTGTGTCAAAAAAAAAATATAT<br>ATATATATATAATTTGATTGACTGTAACACAGAAGATAAATGCTTGAGAGAATGGATACCCCATTTTCCACAATGTGATTATTACACACCGCATGCCTGT	*Alu*Jb	Polypurine tract (10-bp);<br>Cruciform (8-bp, 0-bp);<br>STR (17-bp) (underlined)

42	ATCAAAACATCTCAATGTGCCCCATATCATTTATTCTTTCTTGTTTTATAACTAATTATAACTAACACTTCAACTGCACTTGCCATGCGATACCTAACGG<br>TATATCTGTCATCCTATATTACAAACTGTCTCTAGGTAAGAACATTCTAAGAACCCCACAGCTGGCAAGAAGCCTGCTGCTATCCTGAGAAATCAGCAAA<br>ATGTCAATCTGAACGAACCACATTTAACCTGTGATATAATTTGGCTCTGTGTCCCCACCCAAATCTCATCTCGAATTGTACTCCCATAATTCCCATGTGT	–	Slipped motif (10-bp, 0-bp);<br>Cruciform (6-bp, 4-bp) (underlined)
43	TGCAGGAGGGACCCGGTGGGAGATAATTGAATCATGGGGGCAGTTTCTCCCACACTGTTCTTGGTAGTGAATAACTCTCATGAGATCTAATGGTATTTTA<br>AGGGGAAACCCCTTTCGCTTGGCTCTCATTCTCTTCTCTTGTCTGCCGCCATGTGAGATGTGCCTTTCACCTGCCATGATTGTGAGGCCTCCCCAGCCAC<br>ATGGAACTGTAAGTTCATTAAACCTCTTTCTTTTGTAAATTGCCCAGTTTCAGGTATGTCTTTATCAGCAGTGTGAAAATGGACGAATGCACTAATACAA	THE1B	Cruciform (6-bp, 3-bp)
44	CAGGCTCCCTGGTCCCCCACCTTCTCGGACTAAGTCCTCTGCAGTGTTGACTCCGTGTTCTATGAGCTTCTGGCAGTCATCTAGTGGTTTAATGGTCTCC<br>TGTTCAATGGTGTCCACCTCTTGCAAAAGGGTCACCAATCGCTCATCCAGGAGCTTTCCAAGGGTTCCCTTTAAATCATTAAAATGCTGTTTGAGAACAT<br>CCCTTGTCTGTGATGCACTTTCTTTGATCTAAAAAGAAATAACAAAATACTCAAAATCAGAATCTTTAAAAAAGGCTAATCCAAAGGTATAATTCACATG	–	–
45	AATCTAAAAGCACTGTATAACCATCTTCTTCAATTTAGTAAAAAAAAGATTAACTGAAATTATATTTAAGGTGTGATATTTTGATACACATATACATAGT<br>GAACTGTTAACTATAGTTAAGCTAATTAACATGTCCATCTCTTCACATGGTTACCTCTGTGTGTGGTGAGAACACTTATGATCTCTCCTAACACATTCCA<br>AGTATACATTACACTATTATTAACTATTGTCCCTATCCTGTACTTGAGCTCTCTAGAATTTACACATATTAAATAACTGAAACTCTGTACCCTTTGACCA	L1MA9	Cruciform (7-bp, 0-bp)
46	ACATCTCCCCATCCCCAACTCCATCCCTGGCAAGCACCATTCTACTTTCTGCTTCTATGACTTCGACCTTTTTAGATTCTACATATGAGATCGTGCAGTA<br>TGTTTATTTCTGTATCTGGCTTATTTCACTTAGCATAGTGTCCTTCAGGTTCATCCATGCTGTCACAAATGACAGAATTTCCTTCTCTTTTAAGGTTGAA<br>TGGCATTCCATTGTATATATACATCTTCCATTCATTGATAAACCAAATGTGATTCATTTGCACAAACAGACAGAAGTACACAGAACTGCCCTTCAAGTCA	L1MA9	Cruciform (6-bp, 4-bp);<br>Cruciform (6-bp, 0-bp)
47	GATAGAATAAGCCAAATCTTGGTTTCATCATTCACAAACTATGTCAATTTGGTTAAGTTAATTAAGTATTCTATGCCTCAATTTTCTTATTTGTCAAATG<br>AGCATAATAATAGTTATTTCAGCCGGGCGCAGTGCCTCATGCCTGTAATCCCAGCACTTTGGGAGGCCGAGGAGGGCAGATCATTTGAAGTCAGGAGTTC<br>AAGACCAGCCTGGCCAACGTAGTGAAACCCTGTATCTACTAAAAATACAAAAATTAGCTGGACGTGGTAGTGCGGGCCTGTAATCCCAGCTACTTGCGAG	*Alu*Sz	–
48	GCTGAGGCAGGAGAATTGCTTGAACCTGGGAGATGGAGGTTGCAGGGATCCAAGATTGCGCCACTGCACTCCAGCTGGGCAACAGAGTGAGACGCCATTT<br>CAAAAAAGAGAAATAAATAATAGTTACTTCATGGAGTTGCTGTGAGGATAAATTAAAATAATCTAGGTACTACAGTGGGTAAAATAAATGGCACATTGTT<br>ATACTCGCTAAAAAAAGTTATAAATTATTATCTATACAAAGTTGTTCTTGAAAAGTGTACGTTTCAATACTGAAAATTGTTTAATTATGAGGGGATTAAA	MIRc	–
49	ATTCATTTTACTGAAAAGCAGCAAAGAAGTGGTAGATTAAAAAGCTATGGTCAGCTGGGCATGGAGGCTCATGCCTATAATCCCAGCACTTGGGAGGCCG<br>AGGTGGGTGGATCATGAGGTCAGGAGTTCAAGATCAGCCTGACCAACATGGTGAAACTCCATCTCTACTAAAAACACAAAAAAATTAGCTGAGTTTGGTG<br>GTGCATGCCTGTAATCCCAGCTACTTAGGATGCTGAGGCAGGAGAATCGGTTGAACCCGGGAGGCGAAGGTTCCAGTGAACTGAGATCGCGCCATTGCAC	*Alu*Sx3	–
50	TCCAGCCTGGGCAACAGAGTGAGACTCCGTCTCAAAAAAAAAAAAAAAAAAGCAAGCCATGGTCAGGTGCAGTGGCTCACACCTATGATCCTTGCACTTT<br>GAGAGGCTGAGGCAGGAGGATCACTTGAGCCCAAGAGTTTGAAACCAGCTTGGATAACATGGAGAAATCCTGTCTTTACAAATAATACAAAAATTAGCCG<br>GGCATGGTGGCACATGCCTGTAGTCCCAGCTACTTAGGAGGCTGAGGTGGGAGGATCACCTGAGCCCAGGGGGTCGAGACTGCAGTAAGCTGTGACTGCA	*Alu*Jb	Cruciform (6-bp, 3-bp);<br>Polypurine tract (18-bp)
51	CCCAAATTCCTTTCATTAATTCCCCACCCCCACCCTACCAGTTCTCCCAGAGATGATTAGGTTCTGGGAACAGGTTGCCACAAAACATTAATTTGTTTAA<br>GCTATTTATTATGTAAGATAGTGGTTGAATATCTGGCCCAAGACAGTTAATGGTGTTCCTGGGAGGCTGGCTCATTCAAAACCAAATCATGGGTCAGGTG<br>GCAGTTGGTGAATGTAGGGACCAAACAGCAGTGTCCCGTAGCAGAAGGAGCCCAGCTCTGGGACACAGCCTGGTTCCCACCACTTGTGTGGGCCTTGGAC	–	–
52	ACATTGCTTAACTTCTCGGGCCTTTAGTTTTCTCACTCATAAAATGGGCATGATCATACTGTCACCAGGATTGCTGGGAAAAGCAAATGGGGTCATATAT<br>GTAAAGGGCCCCCAACATTGTGGAACTGACCGAATGGTGTCCCCACAAGTCAGGAGATCCAGTTGGGATTTCCAGCTTAGCTGGAACTTCAGGCAAGTCA<br>TTTCTTTGTCAGGATCTGACTGGATCATATGCCTGAAAGTGTTTTAATGATGACAAAAATTTGCATCTTGTTTACTCAGCACTCTTACATACTGGGCCTC	–	Cruciform (8-bp, 1-bp)
53	ACTGATATCTCTGAGGCTCAGAGAGGTAAAGTGATTTACCCAGGGTCACACAGCAAATGAGTGGCAAAGCAGGAGATAAGACACTAGTTTTGCAACTGTA<br>AATCACCAGCCCTTTCTCTCTATGCCTGTTTGCCCCAGAGCTTGGGGCTGTATAGGCTGGTGGGCAGGCTCACCCCCACATCAGCCTAGGCCCTGGCATT<br>GCTTACCAGCTCCTTCCCCACTGGTGAGGGGACCCAGGGAATATCTGCTGCTGTCCCCAGGGACTGACGTGATGGGGCTAGGTTGTGAGCCCTGGTCGGT	–	Cruciform (8-bp, 1-bp);<br>Cruciform (6-bp, 4-bp)
54	CTGCAAGTGTCAGATAGGATGATACATTAAAAGCACAAAGCACAGCACTTGGCTTCTCTCAAATCCTCCATAGATGTCAGTTAGCTTGAGTACTGCCAGA<br>TTAGAAGCTCTCGTGGGCAGGGATTGTGTGTTTTGTGCCTATAAACTCTGGTAACTACTAACTGAGAAAAAACACACAGTACCCTGGGGAAGAGCAGGAG<br>TTAGGACTGTGGCCAAGAAAGAATGCATGAAAGAGACAGAGAAGAACGGTCAGGGAAGTAGGAGAACCAAGGCGGTATTTTAGCCTAGAAACTAAGGGAG	–	IR (10-bp, 34-bp)
55	GAGAGAATGTTCAGGAAGGAAAAGGTCATGGTGTCAAATGCTGCCAAGAGGCTACAGAGGGTAAGAATGAAGCTTTTTTCCTTTGGATTTTGCACTTAAG<br>AGCATGAGAGGCCTTTGTAAAGGTGTCAGTGGGGTGATCAGATGGCAGAGGACTTCATGGCCAAGGGAGAAGAGACAGAGAATTAGGGGGCTCCTTCCAG<br>CACAACCAGAATAACTAGCACTTATGGAGCACTTGACATGTGCCCACCCACTGTGAAGCATATTTTGTACAATATTTCAGTAATTCCTGTCAACATCCTG	–	–
56	CAAGGTGTGCTATTGTCCCCATCCTAACATGATAAGATCAGATGGCTTAAATAATTTCCCCAAAGTCACACAGCTAAGAAGAAGGAGATCCAGGAGTTAA<br>GCATGGTTCTCTTTGATTTCTTAACTTGAGTCTTAATCAATGTAGGGAAGAGAGGAAGAATAGCAGAAAGGGGCCCCAAGGTGGCAAGGACCCAGGATCT<br>GGGACTCAGGAGGTTGAGGATGCCCCCTTCTTCTGGGTGCTGTCTATCTGAGCCACTTAGTCTAAACCAAGAGTTACTTTGATACCATACATAGTTTTAC	–	MR (10-bp, 62-bp)
57	AGGCTGAAGGAACTTCTCTGCACCCCTGATTCCATAATAAGCCCTTTTGGCAGTGTGAGCCAATGAGGAAGCTTCTGGAAGAGAGTTGAGCCCCGAATCC<br>TTTGAAGATTAAAACAGGCAGGGTATGGTGGCTCACACCTGTAATCTCAGCACTTTGGGAGGCCGAGGTGGGAGGATTGTTTGAGGCCAGAAGTTTGAGA<br>TCAGCCTCGGCAACACAGGGAGACCCTGCCTCTGTGAAAAATAAAAAAATTAGCCAGGTATGGTAGCATGCACCTGCAGTCCCAGCTACTCAGGAGGCTG	*Alu*Jb	IR (10-bp, 66-bp)

58	AGGCTGGAGGATCACTTGAGGCTGGGAGGTGGAGGCTGCAGTGAGCCGATATCATGCCACTACACTCCAGCCTGGGCAACAGAGGGAGACTCCCTCTCAA<br>AACAAACAAACAAACAAACAAACAAAAAACAACCAAACCAACAACACACACACACACACACACACACACACATATACACACAGCATTCACATGAGCATAATG<br>TTCAAATTTCCATTATCTCAACTGGTCCTCCAACCAGCCCATGGTGGACACACGGAATCATCCCATTTGACATATGTGAAAACTGAAGCTGAGAGATTTT	Hal1-3A_ME	Cruciform (8-bp, 1-bp) (green); Slipped motif (12-bp, 0-bp)/STR (27-bp) (pink); Triplex motif (14-bp, 1-bp) (underlined); STR (27-bp)/DR (13-bp, 1-bp)/ Triplex motif (13-bp, 1-bp)/Z-DNA (27-bp) (yellow)
59	GTCAGGAACTGAGGCATCTGAGTGCTACAGGGTTCTGCTGAGAGTAAACTGAAATCTTGTTCTCTGACCAATCCTGCCAAGAGACCAAGGAGAAGGAAAT<br>CACAGAAGACAGTCCAGGACGAGTCATTCATTCATTCATTCTGCATTTATGTGTTGACCACCTACTATGAGCAGACCCTGTTCAAGGCAGAGATTTAGAG<br>GCAGAAAGACAACAATCCCTGGCTTCAAGCTGGGGAGACAGACGAGAAAACAGATGGTGACTCAATACTTCTGTAAGTGCCAGGGAAGCCCAGAGGCCTT	L2a	STR (18-bp)
60	GGTGCAAGGATGCTGGGATGGGAGGGGCTGCACTGCTAGGAAAGGAAGCTTGAGGTGCAGCGCTACACCCCTGAACATGAGCAATATCTTCCCCATTCTG<br>GTGCTCTTATCAGGACAGGGCAGCCAGACACCAAGACACCTGTGGGTGTCCTGCCGACCCAGACAGCCCCGTCAACAGACCTTGTGCTGTCCACTTTCTC<br>TGCAACCTCAATAGCAAGCCCCGGCCAGACACTGCCAATGGGCTGAGGTCTGGGGACAGAACCTGGACCCAGCATGAGTCAGGCTCAAGACAGTCTCATC	–	Cruciform (6-bp, 4-bp)
61	CTTGCTAGCAGCTCTGCGTGACCCGGGCCACTCAACTCACACCCCTGAGCCTTGATTTCTTCATCTGTCCAGTGGGGTCAGGCCTACCCCTATAAGCCAC<br>CAGGAGAATGAAATGGGGTCATGTCCATGGCAGGGCTTTTTCAGCCGTGAATCTCTTTACGAAGAGCAGAGGCTTTTTGTCCAAGGCTCCTGGTGCATTC<br>CCAGCACTGAAGTTCGGGACAGTCCTCGCCTCCCGACAAAGGCAGTGGATGAGTTAATTCTTAAGCTCTTCAGCCCGTGGAGAGCAGAGGCAGAGCAAAG	MIRc	–
62	GGCAGGCCTAGAGGCCTCTTTTGGAAATGACAAGATCCATTTAGCAATCAATTAGCACAGTCCCCAGGCTCCGGGCCTGCACACTCGGCAGAAAGAATGC<br>GTCCTGCTGCACTGATTAGAAACAGATGCGCTGGCAAACAGAGCTGGCCGAGCAGGGCCGGAATTCCTGGGCACCCAGCTCCTGGCTGGGAGACGGGATT<br>TGTTATTACATTCTGGGCACCGACACAGGGAGGGAGGCCAAGGAAACCAAGCTAAGAGAGCAGCCTGCCTGGAGAATGTCTACCTTTGAGAAGATTCAGA	–	Cruciform (6-bp, 2-bp)
63	GGCAGGAGGGGGAAGAATGTTGACTCTGTTAGAAATAATCTGGGATAAAATTAGTTCAGCCAAATACAGAATATGACTTTTGACCAACAGATGCTAAAAT<br>TCCAAATGTGACAAGGCCGACAGATGAGGAAATGAGTGACTTGGGAGCTTGGCAAGTGGCAGGAGGCCTGAAATCTCTGGTGTCTTGGAAAATGACGCCC<br>CCATTGCCAACCTCATTTACAGAGCTATTGTGTACAGGGCCCCGCAGCAAGCCCTTCGTGTGCTATCTCATTTATTGATCACAGCCACCCTCTTATTTCA	–	–
64	CCCATTTTGCAGATGAGAAAACTGAGTCACACATAGGTCCAGCAACTTGCCTAAAATTGCACGGCTAGTAAGTAGCAGATCCAGCGCTTAAAGCCAGCTG<br>TCTGACTTGAGAGGCCCCTCTCTTGGCCATGATGGTATTAATTCCTCTTCTTTCAATTCATTCCTTCTCCCTTAACAAATGAAAGGGCAGCTTTGAGCTC<br>CTCTACCACAGTCTCGAAGCACTGCAGGAAAGGTCCACTGCAGTGTCCCTAACAGTGGTCAGCAACCTCGGTTATACTCTCCCAGTGACGAGGGGCTCAC	–	Cruciform (6-bp, 2-bp)
65	TGTCTCTTAAGGCTATGCTGCCTCTAATCTTGGACAGTTTGCAAGGTTGGCCAGTCTTTCTTCTATTAAGCCAAAAGTTGGGTCCCTGCAAGCTTGAGGC<br>GTCCCCCAGCAGTGCTGCTTCTGCCCTCCAGATTCCCTTAGAAGGCAACTAATTCTTTTTCCCTAGGACCATACCAGGCCATAAGGATGGCCCTATCTTT<br>ATTTTTTTAAGCAGAATAGCCTCTGTTTGAGCAGCTGCTGCCAGCACGGGCAGCTGTGGTGCTCTCATCTCATCACCGACACATTCCCCTGACACCTTCC	L3	Cruciform (6-bp, 4-bp)
66	AACCCATCGATGTCCCTGGTGGGTCCGGGCATGGACGTGAGGGTCAACCAGGTCTCCCATCTCCCAGCAGCACAAAATGGAACAGCCTTTAAAATATTAT<br>TTTAATATGGTTTGTTTGCCATTACAAGAAAGATACATTCTCACTAAAGGAAACGTAGAAAATGCAGTGAGCAGAGCAAAAGTGCCTCCCCAGAGAAAGA<br>AAAATCACCCATGATTCCACCTCCAGCCTTTTTTGCTATGCTTCATTTTTAACCTTGCTTCGTTTATTCTATAGCTACACATCGCATCCTACCCCAAAAC	L1ME4a	Cruciform (8-bp, 1-bp); Cruciform (6-bp, 3-bp) (underlined)
67	TGGAGAGACAGACAGGAGTACACAGAAAATCACAACTTAGCAGGACAGAAAACCTGAGCAGAAACCACTGTGGGAACCAGTATTGGGGCAGGAAAACCTG<br>AACCGTAACTGGTAATTGCTGGAGTCTCAGCGTGGACGAGTCTGAGAGTAAAACCCCGAGGGAGCCCAGTTGCAGGCGAGCCCTCACTCTTGTGTGAGTC<br>TTATCTACAGGAGCTCTAACAGCTTCTCACAGTGATGATCAGAGAAAAATCCAGTGTTCCCAGCAGGAGGAGGTGAAAAGTAGCCATTCTGAAATGCACC	L1MCa	–
68	AGAGCATTCTGTTCTCTGTAAGAAGGATGGCCGGGCGTGGTGGCTCACGCCTGTCATGCCAGCACTTTGGGAGGCTGAGGCGGGTGGATCATGAGGTCAG<br>GAGTTTGAGACCAGCCTGGCCAATATAGTGAAACCCCATCTCTACTGAAAATACAAAAAAATTAGCCGGGCCTGGTGGTGGGCCCCTGCAATCCCAGCTA<br>CTCGGGAGGCTGAGGCAGGAGAATCGCTTGAACCCAAGAGGCGGGGGTTGCAGTGAGCCAAGATCACGCCATTGCACTCCAGCAGGGGTGACAGTGCAAG	*Alu*Sg4	–
69	ACTCCATCTCA**AAATAAATAAATAAATAAATAA**GGACTGCGCTCAATACAAACTGTTTTATCATAGCCTAACCTACTGGGGCTTTATGAGAGCCCAATAA<br>CCCGGGGGAAGAGAAATAGCCAACTCTAGCCCCCTCTAGCCACCCTTTTCCACCTAAGGAGGGGGTACTGAGAATCACTTGTGAAGATCACAGCCCAAGG<br>GCACAGGCTCACCAACAGACTGAGGCCTAATCACAGGACTATCGAACACTTCCTTTCCCCGCAGCATCTGACCACCAAATCACTAAAGGCCTGTTGACTA	L1MCa	DR (10-bp, 2-bp); Triplex motif (10-bp, 1-bp) (underlined); STR (22-bp) (bold)
70	CAGTTCCATTTACCCAGTACATCATGTCTGGCCATCAAGAAAAAAATTACAAGGCATACCAAAACACAAAAAAACACAGTTTGAAGAGACTGAGCAAGCA<br>TCAGAACCAGACTCAGACATGGGAGAAATGTTGGAATTAAATCAGACCAGAAATGTTTAAAAAACTATAATTAAGATGCTAAGGGCGACCAGGCATGGTG<br>GCTCATACCTGTAATTCCAGCACTTTGGGAGGCCAAGGTGCTAGGATCACTTGAGACCAGGAGTTTGAAACTCGCTCTGGCAACACAGCAAGACCCCTGA	L1MCa	Cruciform (6-bp, 1-bp)
71	TTCTACAAAATATTTTAAAATTAGCTGGGCATGGTGGTACGTGCCTGTAGTCCCAACTATGTGTGGGAGGCTGAGGTGGAGGATCGCTCGAGCCTGGGAG<br>GTCGAGGCTGCTGTGAGCCCTGATCATGCGCCACTGCATTCCATTCTGGGCAACAGAGCAAGACTCTGTCTCAAAAAAAAAAAAAAAAAAGCACTCTTCA<br>TTTCTGTTACCACGCTTCTGATCTCTAGCAGAGAATCATACAAACAAAAACTGAGGGAATTTGTTGTTAGTAGACCTGCCTTGTGAGAAATGTTAAAAGA	*Alu*Jr	Polypurine tract (18-bp)

72	AGCTTTTCAGAGAGAAGGAAAATTATATGTCAGAAACCTGGATCTCCATGAAGAAAGGAGAAGCACTAGAAAAGGAATAAGTAATGGTAAAATAGAACCA<br>CAGGATGTCACAACTGGAAACACTCTTAAAGATCATCCTCTCAAGTTGTGGATGGTAGAGCTGAGTCCAGAGAGGGCAAGTGACTTGCCCATGGTCACAC<br>AGTGGCGGTGCCTGGCCAGGAGCCAAGGGCTTCCGGCTCCCAGCTCAGGGCTCTCTCCACTGCCACGCCCACATGGCTCAAAGTGAGTATACTCCCAGAC	MIR3	Cruciform (8-bp, 1-bp)
73	AGCCAGGGAGGGCTCAAGTTAGGACAAGGTTTGTTATTCTTTGACACTAAGAGGAATAAATCAATCAAGTCATTCTTAGCTAAAACAAGTGACCTCTTAC<br>CAAGAGTTCAAATTTAATCATAATAAATAAAAAGAGGGAATGGAAATCAGCAATGGTGCAGCAGACCACAGATGAGGTGATCTGGAAGCACACAGAGCAC<br>TTCCATTCTCCTCGATGGCCACTCTCTCTCTCGCTTTCCTTCTTCCTCTCTCCTACGCTCTCAGTTGCCACCACCAACAACCCCTTTGGGGTCATTAGAC	–	STR (11-bp)
74	TTGCAGCTCTTGGGTATGAGGTACTGACATCTTGACACCTGGAGAAGAGAGTGGGCGCACAGGACCACACTCTCCAATCAAATCAGATCCAGAGGGCATG<br>ACTAGAGGGGGAAAGTCTTCCTATGACTGCTGGGACGGGTTAATTTGTGGAAAACAGAAATGTAAGTGGACAACAACATCTAGAAATCCCCTTTACCAGC<br>ACGGAACTTCCAGAGACTTAAGTTGCTTCACATCTCTGGTATCAGAGTCCTCATCTGTCCAATTTATTCTTTCAATGTATTCCACAAAAATTTCTTGAGT	–	–
75	GCTTGCTGTGGGCCATGATCTGCTGGGCACTGGGGGACAGCCAAGAGGTTTAAAGATCTCGGTTACCAAGAGAAACTAAGAACAAAAGAAGAGAGGCTGG<br>GCACAGTTGCTCGCACCTGTAATCCCAGCACTTTGGGAGGCTGAGGCTGGCGGATCACTTGAGGTCAGGAGTTCGAGACCAGCCTGGCCAACATGGCAAA<br>ACCTTGTCTCTACTAAAAATATAAAAATTAGCCAGACAAGGTGGTGTATGCCTGTAATCCCAGCTACTCAGGAGGCTGAAGGTAGGAGAATCACTTGAAC	*Alu*Sz6	–
76	TCAGGAGGCAGAGGTTGCAGTGAGCTGATATTGCACCACTGCACTCCAGCCTGGGTGACAGAGTGAGACTCTGTCTCAAAAAAAAAGAGAATATTAATTA<br>AATACTTTTTTTCTTTTCAGGCATTATAATAGGCAAAAGGGACACAATGATTAACAAAAACAAATAGTAAGCCTCATGGTCTTGGAAATAGGATTCACTT<br>AGATAACCTGAAAACTCTCCCACTAATAGGCACCTAGAAATTATGGATAAAATACAATAGCTGGCCTCTGAAAAGCAGAGCTAAGAGCTCAAGTGAGTAA	–	Cruciform (8-bp, 3-bp);<br>Cruciform (6-bp, 3-bp) (underlined);<br>A-phased repeat (26-bp)
77	GGAAATTCCCAGGGACCAAAAATTGAGAGAGGAGTAAATATCAAAGGAATAAGCTTTTGAGTTAATGCTGTAGTTGTATGGGGTGGGGGTAGGAGGTAGA<br>GGGGAGTGAAGAGGTTATAAGGAAGCTTTTCTGCTCTATCCTAGTCTCTGAGTGGGAAAAAATCTCCCCCAAGGGCTGGAACTGTGGGGCTTCACCTCAT<br>GTCAGGTGAGAAAGTTTGATTGTACTCCTGCAGTCCAGGAACTCCCAAACTGGTCATGTAACCTAATGACTATCTTCAAGACAGTGACACCCCTAGGATT	L1MEd	–
78	CCTGACAGTAACAAACACAAAACTACTCTGGAGGGCATGCTCTCAATGAAGGCTGCGTATGATTCCCACAGAGAGAGGGCTACCATAGATGAGCTCATAG<br>TCCAAAATTACAAGTTATGTATGTGAGCAAGAATCAGACAGAGTAAGCAATAGATTTGATCAACCTTGCCAAATGTTCAACCATTAGAACAAACATAGAA<br>ACTTCAAAATAAGTAAGCCTTAAATAATTAACAACATAAAGGAAGGGGCTCAAAACAAGAAAAAGAAAGATGTCATTACAAAGAACAAGCATATTTGGAA	L1MEd	–
79	AATGCCCAACTTGAACTGTAGAACTGTATTTTTCTGTAGAAAAATACAGAAAGCCACTGAAATGTAAAACCCAATAGATGAATTAAATAGCAGATTAAAT<br>GCAACTGAAGAGATAACTGGTGAGCTGGAAGATAGCTCTGTTGATAATGCATTTTATTGCTTCTTACGCAACAGAATGTTTTTTAAATTTAATTTTCTTC<br>ATTTCTTTTATTTTTAAAAATTAATATTTTTAATTGAAAAATCATAGTTGTATATATTTGTGGGGTACAATGTGATGTTTTGATATATGTATACAATGTG	–	Cruciform (12-bp, 2-bp);<br>Cruciform (6-bp, 0-bp);<br>Cruciform (8-bp, 4-bp) (underlined)
80	GAATGATTAAATCAAGATAATTAACATACCCATCATATCACCTCACTTGTTTTTGGTGGTAAGAAATTTGAAATTTACTCTTAGTTTTTGTTTTTCATTT<br>TTTGAGACAGTCTCACTTTGGCCCCCAGGCTGGAGTTCAGTGGCGCAATCTTGGCTCACTGCAGCCTCAACTTCCTGGGTTCAAGAGATCCTCCTGCCTC<br>AGCCCCCCACAAAGTAGCTGGGACTACAGGCATCCACCACCACACCTGGCTAATTTTTTGTATTTTTTGTACAGATGGGGTTTCGCCATGTTGCCCAGGC	*Alu*Jb	Cruciform (6-bp, 1-bp);<br>Cruciform (6-bp, 1-bp);<br>STR (10-bp)
81	TGGTCTCGAACTCCTGAGCTCAAGCAATCCACCCACCTCAGCCTCCCAAAGTGCTAGAATTACAGGTGTGAGCCACCACGGCCAGCCCACTCTTCGTTAT<br>TTTGAAATACACAATACATTTACATTATAATTGACTTTAGTCATCCTGCTATGCAATAGACCTCCAAACTTATTCCTCCTACCTGAGGAATTCCAGCTCA<br>CCAGTTATTGCTTCAGTTGCATTTAATCTGCTATTTAACTCGGACTGTACAATTTGACCAATAACTTCCCCTTTCCTGCCCCCACTCCCAGCCTCTGATA	L1MA8	–
82	ACCATCATTCTACTCTCTACTTCTATGAGTTCAGCTTTTTAAAATTTCACAAACAGGCATATCTCAGAGATATTGTAGGCTTGATTCTAAACTGTCACAA<br>AAAAGCAAGTATCACAATAAAGCAAATCACAAATTTGGGGGTTTCCCAGTACATATAAGTTTTGTTTACACTGTACTGTAGTCTATTAAGTGTGTAATAA<br>CATTATGTCTAAAAATGTGCATACCTTAATTTTGTTTTGTTTTGTTTTGTTTGAGACAAAGTCTCGCTCTGGTTGCCCAGGCTGCAGTGCAATGGCACCA		Cruciform (7-bp, 1-bp);<br>Slipped motif (10-bp, 0-bp)/STR (23-bp) (pink); Triplex motif (11-bp, 0-bp) (underlined)
83	TCTCGGCTCACTGCAACCTCCACCTCCCGGGGTTCAAGTGATTCTCCTGCCTCAGCCTCCTGAGTAGCTGGGATTACAAGCGCCTGCCACCATGTCCAGC<br>TAATTTTTGTATTTTTAGTAGAGACGGGGTTTCACCATGTTGGCCAGGCTGGTCTCGGACTCCTGACTTCAGGCGATCCACCCGCCTCAGCCTCCCAAAG<br>TGCTGGGATTACAGGCATGAGCCACCGTGCCTGGCCACATACCTTAATTTCAAAATACTTTACTGCTAACATGATAACACAGAAATACAAAGTGAGATTA	*Alu*Sq2	–
84	TGCTGTTGCGAAAATGGTGGTGATAGACTTGCTCAACAAAGGCTTGCCACTAACCTTCAATTTATTTTTTAAAAAAAGCACTATCTGCAAAGCACACTGA<br>AGCAAGGTACGCTTGTATAAGTGAAATTATTTGGTATTTGTCTGTGTCGGCCTTATTTCACTTAGCATAAGGTCCTCCAGGTTCATCCACATTGTCACAA<br>ATGACAGGCTTTCCTTTTTCAAGGCTGAATAGTATTCCATCATGTATATGTACCACATTTTCTTTATCCATTCATTTGTTGATGGACACTTAGGTTCATT	L1MA8	Cruciform (6-bp, 0-bp);<br>IR (10-bp, 27-bp)
85	CCATATCTTGGCTATTGTGAATAATGCTACAATAAACATGAGAGTGTAGATGTCTCTCCAATATACTGATTTCATTTCCTTTGAATATATACTCAGTAGT<br>AGGACTGCTGGATAATATGGTAGTTCTATTTTTAGTTTTTTGAGGAACATCCTTTCTGTTTTCTGTAGTAGCTGTACTAATTTACATGCCCCCCTACAGT<br>GTACAAGGGTTCCCTTTTCTCTACATTCTCACCAACACTTGTAATCTTTTATCTTTCTGATAATAGTCACCCTAACAGGTGGGAAGTGATAGCTCATTGT	L1MA8	–
86	GGCTTTAATTTGTATTTCTCTAATGATCAGTGACGGTGAGCATTTTTTCATGAACCTATTGGCCATTGGCATGTCTTCTTTTGAGAAATGGCTGTTCAGG<br>TCTTTTGTCCATTTTAAAATCAGGTTATTTATCTTATTGCTATTGAGTTGAGTTCCTTATATATTTTTGATATTAACCGCTTATCAGATGTATGGTTTGC<br>AAACATTTTCTCCCACTTTGTGGGTTGTCTCTTCATTTTGTTAATTGTTTTCTTTGCTGTGCAGAAGCTTTTGGGTTTGATGCAATCCCATTCATCTACT	L1MA8	–

87	TTTGCTTTTGGTGCCTGTGCTTTTGGGATCATATCCAAAATATCATCACCCAGACCAATGCCATGGAGGTTTTTCCTTATGTTTTCTTCTAGTAGCTTTA<br>TAGTTTTAGGTCTTTTAATAATCTTTAATCCATTTTGTGTTGATTTTTGTAATGATGTGAGATAAGGATCCAATTTCATTCTTCTGCATGTGCGTAATT<br>GATTATTTCCAGTTTTCCAAATGCCATTTATTGAAGAGACCGTTATTTCCCCATTGTGTATTCTTTTGTGCCTTTGTCATAAATCAATTGACCATAAATG	L1MA8	–
88	CCTGGGTTTATTCCTAGGCTTTCTATCCTGTTTCACTGCTGGGTCTGTTTTTATGCCAGTACCATGTTCTTTGGATTACAGTTGATTTATAATGTACTTT<br>GAAATTAGTAGTGTGATGCTTCCAGTTTTGTTCTTTTTGCCCAAGATTGTTTTGGCTATTCAGGGTCTCCTGTGGTTCCATATGAATTTATGAATCTTTT<br>TTTTCTATTTTTGTGAAAAACTGACATTCAAATTTTGGTAGAGAGTGCACTGAATCTGTAAATCACTTTGGGTAGCATAGACAGTCTAATAATATTAATT	L1MA8	A-phased repeat (27-bp)
89	CTTCCAACCCACCAATGCAGGATATTTTTCCATTTATTTGTGTTTTCTTCAATTTATTTCATCAGTGTTTTATTTTATTTTATTTTATTTTATTTTATTT<br>TATTTTATTTTATTTTATTTTGAGATGGAGTTTCACGCTTGTCGTCCAGGCTGGAGTGCAGTGGCATGATTGTGGCTCACTGCAACCTCTATCTCCCAGA<br>TTCAGGTGACTCTCCTACCTCAGCCTCCTGAGTAGCTGGCTCTACAGGTGCCTGCCACCATGCCCGGCTAATTTTTCCTGTTTTTAGTAGAGACGGGGTT	*Alu*Sq2	Slipped motif (25-bp, 0-bp)/STR (54-bp) (pink); Triplex motif (28-bp, 0-bp) (underlined)
90	TCGCCATGCTGGCCAACTCCTGACCTCAGGCGATCCGCCTGCCTTGGCCTCCCAAAGTGCTCCGATTACAGGCATGAGCCACCGCACCCAGCCCATCAAT<br>GTTTTATGTAAGTTTTCAGTCTTTAGGTCTTTCACCTCCTTTGATAAAGTTACACATAATTATTTTATTTTGTGTGTTTTCCTACTGTAAACGGGATTGT<br>TTTCTTAATTTTCTTTCCAGATAGTTTGCTATTAGTGTGTAAAAATGCTGCTGATTTTTATATGTTGATTTTGTCTGCTGCAACTTTACTGAATTTATTT	L1MA8	Cruciform (6-bp, 3-bp)
91	ATCAGCTCTAACAGGCTTTTTGGCAGAGTTTTTAAGGTTTTCTATATATAGATTGTGTCACCATAAAACAGAGAAAATTTCACTTGTTCCTTTCCTATTA<br>GGATGCCTTTTATTTCTTTCTCTTGCCTGATTGCCTACAATAGAACATTTTAAGAATAGTATTACGTTGTTATAAGTTGCAGTGTTATTTTGAGACCAAT<br>CATTCAAAGGATAAAGAAAGAGAGATTGATTATTTTAAACATAGATATAACAAGCTTTTAAACATTAACTTCAAAACATATCTGTATCTCACATTTGAAC	–	Cruciform (6-bp, 0-bp)
92	AGACTAGCTTTTGCCATCATACATCTCAAACCTTACGTCTCCTTTAACAACCATATGCATCTAATGCTGAACATCATAGGCCTGTTATCCTAGTACAACA<br>TCTGATCTCTGTACCTTATGCCACAATCTCAGGTGAACTGACAGGGTTAGTGGGGAGTAGTATTATTCCTGGAAGCCATTCCCAAACCAGGTGGCTAGCA<br>ATGCCTTACCTACACACGGAAATAACTGAGATCAATATAAATATATAGCACTGGGCTAGATAGTCCATTGTCCACTTGGTACCACCATCACTTCTTTTAA	–	–
93	GTCAGATAATTACTTTTACTACTATGACTCCAAGGTAGAGTTAGCCAATAAGAAAACTTGAACAAGATTGGAAGGTGGGTAGAAGTAAGGTAAAATAATA<br>GCCATTTTTTTCAGGTTGTGACAGTCAGACATGGATTTGCAATTTTTCCGCAAGCTCCCACTCAGAGGTTGTACAGAAACTGCATTTCATAGCCTTTCCA<br>CAAACTTACTTCACAGTCATAATGGCAAGATATGGTAGTTTCTTAGACTGTCTCATAATCAGTGGCTTCTCCCAGCCCCACCCCCAGGCTGGAGGTATGG	–	–
94	ATGTATGGATATTACCTGGATTCCTAGACAGAGGCTAATGGTTTAGCTGGAAGGGCAGGGACTTTGAAAGAATAAGATTACCAGGCATGGTGGCTTACGC<br>CTGCAATGCCAGCACTTTAGGAGGCCGAGGCGGGCACATCACATGAGGCCAGGAGTTTGAGACCAGCCTGGCCAAGATGGCAAAACCCCATCTCTATTAA<br>AAATACAAAAATGGGCCAGGTGTGGTGGTGCATGTCTGTAATTCCAGCTACTCCTGGGGCTGAGGCACAAGAATTGCTTGAACCTGGGAGGTGGGGTTGT	*Alu*Sz6	–
95	AGTGAGCTGAGATCGCACCACTGCACTCCAGCCTGGGCAACAGAGAATCCATCTCAATAAATAAATAAATAAAATATGTAGGAAAGATGACAGGGCCATA<br>AAAACCTAAGCCATTCTTGAAGAAGAACAAGAGGGGAAGATTTGCCCTAGTAGATATGAAAATAAAGGCTGCCCTACCAGCTATCAAGACAAAGCTACAG<br>TAATAAAAAAAATAGTATGGTATTGGTGCAAGAGTAGACTGATGAAATAGAAGAAAAAGCCCAGAAACAAATGTAGAGAGATATGGTGCTATGGTCTAAA	L1ME3D	STR (18-bp)
96	TGTTTATATCCCCCCGAAATGCATACGTTGACATCCTCCTCCCCACGGTGATGGGAAGTGGGGCCTTTGGGAAGTGATGAGGTCAAGGAGGTGGCACCCT<br>CATGAACAGGATTAGTGCTCTCATAAGAAGAGGCTTGCAAGACAACCCTCACCCCTTCCACCATGTGAGGACACAGTAAGAAGTTGCCATCTAAGAGCCA<br>GAAAGCAGGCCCTCACCAGACACCAAATTCGCCTTGATTTTGGACTTCCCAGCCTCCAGAACTACGGGAAATAAATTTCTGTTGTTTTTAAGCTACCAGT	MLT1A0	–
97	TTATGGTATTTTGTGTTAGTAGCCCAAGTGAACTAAAACATATGGAAGCTGGATTTATGACAGAACAGATGTTATAGTTTAATAGGAAAAGGATGGACCA<br>CAACAAATGATACTGGCACAATTGGTTGTCCATATGTAAAAAAGTTAAAATTAATTCCTCCTACATGCCCTACATAAAAAGCCATTTAAAGTAGCTTAAA<br>CACAAATGTGAAAGGTGAAACTATAACAAAAATTAGGACATGATATAGGATAATATAGAACAATAATTTCATAATTTAGGTCCTCAGGGTAGAGAGAGAC	L1ME3D	–
98	TTCTTAAATAAAGGACAAAACGGCACCTACCTTAAAAAAAAAAAAAAGGTCAGGTGAGCACAGTGGCTCACACATAACCTCAGCTACCCACGAGGCTGAG<br>GTAAAAGGATCGCGTAAGCCTAAAAGTTCGAGGCTGCAGTGAGCTATGATCACACCACTATACTCCATCCAGCCTGGGTGATGGAGTGAGACCTCAACTG<br>TAAAAAAAAAAAAAAAAAAAAAAAAAAGAAAAGAGAAAAGGAAAAAGAAAGAAAGAAAGTGCATGAAGTAATTAAGAGCTTCTGGTTTTCAAGAGACTTC	FRAM	Polypurine tract (14-bp); Polypurine tract (26-bp)/Triplex motif (13-bp, 0-bp)/Slipped motif (13-bp, 0-bp); STR (16-bp)
99	ATAAAGAATGAGAAAAAATAAGACACAAACTTGAAGATGACAATTGCAACACATATTACAAAGGATTAATTTCCAGAACGTAGAAAGAATGCTTATAAAT<br>CAATAAGAAAGAGAAAAATAGCCCCAAAGGAAATAGATGAAAACCCGGAATTCCACTGAAGATCAAAGGCAAATGTGTAATAAATATGAGAATATGCTTA<br>ACACAATTGAGAATTAAGGAAATGCAAATTAAAATCACAAAGCCAAAATTAAAAGTTTAAACAAACAGACTCAAAGCCCTACCATTCCATGCTTACCAAC	L1ME3D	–
100	TGGGCAACAATTAAAGCACTGACAGTCCCAAGTGCTAGTGAGCATGTGAAGCAAAAGAAATTCAAATATTGCAAATGGGAATGGAAACTGATATCACCAC<br>TGGTTGTTAGGTACTAATTAATAATGTTGAACATGTGTACACTCTATGACCCCCAGTCCCAGTGATAGGTATATAATTACTAAAAAAGTGGAAACATTCT<br>CTGTGTCCATAATAAGTAGAGTGGATTTATGTATTGTGATATATTCAAACACTGGGCTACTATACCACAGCTGAAATAAATGAACAACAGTGATATTCAT	L1ME3D	–
101	CACTGCACTCTAGCTTGGGTGACAGAGTGAGACCCTGTCTCTAAATAAAAATGAAAAGCCACAATACAAACAAACAAACAAACAAAAAAACTCATGGATA<br>AAATGCCCTCATTATGCTAAAAGAAAACCAAAAATTATTTAAAAACCAAAGAGGCCTGTAGGAGGCAGAGCTGGGACAATGACTTAATCCTTTTTGAATT<br>ATAGTGCTCTTTGTCAATCTAAATGAGAGGTTTATAAAGCCTCTTCCTTTCAAAGTTATTGAAAGAGAAGAAAAAGCCTCTTCCCCAAAAAATACATAAT	–	STR (21-bp); Triplex motif (12-bp, 2-bp)

102	CTATACCCTAAAAAATTATTCAATTGCAGAAGTTTACAAATTCCCTCAGAGCCAAACCATGGCTTGCCTGGAGCAGTAATTATGAAACTTCGTTTGCAGA<br>AGAATCATGTAGGCTACTTGTTATAATTCAGACTGCAGAGTTCCATCCCAGACCTACTGAACCAGAAAATGACAGGAGGGGCTAAAGGAGTGTATGGATG<br>GAGTTCTCATTTTTTCCAAGCACACCTGTGATTCTGTAGCCCACAATTTGAGAAACATTGCCCTAAATCTACTGATCTCAGATTAAGAACCCATATTCTA	–	–
103	GGGGATATGACAGGAAAATCTTCATGTCAAATATATTATGAAGATAAATAGGTTGTTTATACAGACACCAGATTTTTCTTCTTTTATTTCAGAGACAGGG<br>TCTCACTCTGTTGCACATCCTAGCTCACTGCAGCCTTGAACTCCTGGGCTCAGGTGATCCTCCTGCCTCAGTCTCCTGAGTAGCTGAGCTTATAGGTGCG<br>GGCCACCACACTTGGCTAATTTTATTTTTCACTTTTTGTAGAGACGAGATCTTGCTATGTTGCCCAAGCTGGTCTTGAACCAGCTTCAAGCAATCCTCCC	*Alu*Jr	–
104	ACCTTGGCCTCCCAAAATGCTGAGATTACAGGAGTGATCCACTGCACCTGGTCCAGATTTTTATTTTAAAGACTTCAGATAACAGGCCAGGCATGGTGGC<br>TCATGCCTGTAATCCCAGCACTTTGGGAGGCCAAGGTGGGAGGATCACCACTGCAACCTGACCTGGGCGACAGAGCAAGGCCCTCTTCTCAAAAAACAAT<br>TCAGGTAACATATAATTCATTTTCTCTCGCTTAATGGGAAAATTCTAACTATAAGCCACTCCGATAAGTTAAAGCAAAAACATACTTTGCTTTCTAGATA	FLAM_C	–
105	TTATTATCTTAGTTTCATATGACATATTGTTTACCACCATAACCCTTTTGTTAACTTTCTCTTATCAAGAAAGTTTAAAGTATAAGAAAAATCCACCAAG<br>AAAGAACATATCAAAATATGAATAAAGGACAATATTTCAACAGTTAATATAGAGATTTAAAAACTTTCTATTTATTCTCTAAATTCTCTCTAAATTTAAA<br>AAGCAGAATACAATATTGTTTCCCAGCAATGTTCACGACCATGTAAAAACAAAAAATGCCCATGCCCAAGGACTAAAACAGAAAAAGAGCAGTATCATTT	–	DR (10-bp, 1-bp)
106	ACTGGGTGGGCAGACCATAGAAACAATTTTCTTTCCTTTTTTTTTTTTTTTTTTTTTTTTTTTTTTTTGACACAGAGTTTCACTCTTGTGGCCCAGACTG<br>GAGTGCAGTGGCGCAATCTCGGCTCACTGCAACCTCTGCCTTCTGGGTTCAAGAGATTCTCCTGCCTTAGTCTCCTGAGTAGCTGAGATTACAGGCGCAT<br>GCCACCACGCCTGGCTAATTTTTGTATTTTTAGTAGAGACAAAGTTTCGCCAAGCTGGTCTTGAACTCCTGATCTCCGGTGATCCACCCACCTTAGCCTC	*Alu*Sq	Polypyrimidine tract (32-bp)/<br>Triplex motif (16-bp, 0 bp)/<br>Slipped motif (16-bp, 0-bp)
107	CCAAAGTGCTGGGATAAGGCGTGAGCCACCTCATCCAGCCCTTTTCTCTCCATTTGACATTAACGCAGTTATAATAATATTTATGCAGTTAGTAAAATAA<br>TTCTTTAAATCACAAAGAGAAATCCTAAATTAATTGTTAACTAATTTCTGTATTTACCAAAGAATTTCTGTGAAATAAATCCAAGTAAAAATTTTAAAAA<br>GAAAGAAAGGCCATAAACCCAAATTGCACTTGATTCAGCTCACCATTCAACTATGAGATCTTTGCAAACTGGGCCCTCATCTACATAGCATCTGGTAAAC	–	STR (12-bp)
108	AAATGCTGTTGAGTGAAAACGCAGTTAATCATGTGTGTTTCAAAGAACTTTCTAAAAGAACCAAAGAGAGAATACCTTATGATTTTAACACAACCCCTGG<br>GATACTGTTAGAAATTCAGAATCTCAGGCCCCACCCAGAACTGCTAAATCAGAACATGAATTTTTTTTCTTTCCTCCTCCTCCCCTAGAACCTATATTTT<br>TTTTTTTTTTTTTTTTTTTTTTGGAGATGGAGTTTGCTCTTGTTACCCAGGCTGCAGTGCAATGGAGCAATCTTGGCTCACTGCAACCTCCACCTCCCG	–	STR (12-bp);<br>Polypyrimidine tract (26-bp)/<br>Slipped motif (13-bp, 0-bp)/<br>Triplex motif (13-bp, 0-bp)
109	GGTTCAAGTGATTCTCCTGTCTCAGCCTCCTGAGTAGCTGGGATTACAGGCGCATGCCACCATGCCTGGCTAATTTTTGTATTTTTAGTAGAGATGGGGT<br>TTCATCATATTGGTCAGGCTGGTCTCGAACTCCTGACCTCAGGTGATCCTTCTGCCTCAGCCTCCCAAAGTGCTGGAATTACAGGCGTGAGCCAACGCGC<br>CCAGCCAAACCTG**TATATATATATAT**TTTTTTTTTGAGTCTTGCTCTGTCGCCCAGGCTGGAGTGCAGTGGCGCCAACTCGGCTCACTGCAACCTCCGCC	*Alu*Sq2	Cruciform (6-bp, 0-bp) (green)/<br>Cruciform (6-bp, 0-bp) (bold);<br>STR (13-bp) (underlined);<br>Polypyrimidine tract (10-bp)
110	TCCCAGGTTCAAGCAGTTCTCCTGCCTCAGCCCCCCTAGTAGCTGGGATTACAGGCATGCGCCACCAAGCCCAGCTAATTTTTTTGTATTTTTAGTAGAG<br>ACGGGGTTTCACCATTTTGGCCAGGCTGGTCGCGAACTCCTGACCTTGTGATCTGCCCGCCTCAGCCTCCCAAGGTGCTGGGATTACAGGCATGAGCCAC<br>CGCACCCGGCCAAATCTGTATTTTTAACAGGACCCTCAGTTGCTTTGTATACATATTACAGATACCAGAATAAGTGAAGACCTTGTGTCAGAACGCTGTA	*Alu*Sx3	–
111	ATACATTTCGAATGGCCAAGAGGAAATTCTGCAGTTGAGCACTGTGCCTCTGAGGTCCTGTCTTGCTCTTTAGCGGATATAGCATACACAGGAAGTGCAT<br>TGGTTAGGTAAAGTCAATTTCCTCTTCAGAAAGAATTCAAAAGCTCCTTCAGCTGTTGCAGAGCCTACGTTTTGTTTTTGTTTCAGAACCTCAGAAAAAA<br>GGAAGTAAGTGAACACAAAACAGACAGAGGGCTAAAAAAATGAGACTCAATTTCCAAATTCACCTCCTGTACTCTCATTGTAAATTTGGTCCCTTTTGCT	–	IR (11-bp, 87-bp)
112	CATAGAATCCCAACCACTGCCATTTGAAACTTGATACCACCATGGGATCATTTTCTTTTCCTTGAAATATTTCATGATTTAAAGCCTAGTCACCTTTAAT<br>CCAATCATGTAACTAATCAAGTTCATTTGCTTGACTTTATGAAACTAATTTAAAAATGCAGCTCCACTGCTGAGTGAATTTCTTCATATTTGATGTGGTA<br>CCAATGGTTCCATGCGTTCACTGTTAACTCAATAAATCTTCACAATGCACTTTCAGAAAGTGAGGACCAAAGAAGAGAAAGAAGACAACTTACCCAAGGC	–	Cruciform (6-bp, 0-bp);<br>Cruciform (7-bp, 1-bp)
113	TTCGCAGTGCCAGAAACAGTCTAACTCCAAAGACTATGCTCTTTCATTACACCATGCTGTTTTGTACTTACAGGACACTTTTTTTTTGAGACAGGTTCTC<br>ACTTGGTCGTCCAGGCTGGAGAGCAGTGGTACAATCACAACTCACTGCGGCCTCGAACTCCTGGGCTCAGGTGATTCTCCCACCTTAGCCTCCCAGGTAG<br>CTGGGACCACAGGCATGTGTCACTACACCCAGCTATATTTTGTATTTTTTGTCGAGACGGATTTTCATCATGTGGCCCAGGCTGGTCTCGAACTCCTGGA	*Alu*Jo	Cruciform (6-bp, 4-bp)
114	CTCAAGCAATCCTCCTGCCTCGGCCTCTCAAAGTGCTGAGATTACAAGTGTGAGCCACCGCGCTCGGCCATAGAACACTATTTTAAAAGAGCTCCCAGAT<br>AAATCTGTAAATTATGTATTGGCTAAAGACACTTCTTGGTATAACACGTAGTATTTCCTGTCCCTGTGTAAAGAATTGTGTGGTGGACAACCAATCAGCT<br>TCAAATGCTACCTAGTCATTCAAGGCAAAAACAACTGTGGTGATTTGTAACAAAATAGATTTAAGAATACAAATGACAGACAAGATTTCAAACAGGAAAC	–	–
115	TGTAATATACATTGTTTATGTTGATCAATAATATTTTGAAATAATCTGCCACTAAAACCTTTAATGTAACAGACCATTAAAACTTACAAAGTTTTGGCCT<br>GAGCACATTGGCTCATGCCTGTAATCCCAGCACTTTGGGAGGCCCAGGCAGGTGGATCACAAGATCAGGTGGTCAGGAGTTCGAGACCAGCCTGGCCAAC<br>ATGGTGAAACCCCGTCTCTACTAAAAATGCAAAAATTACCCAAGCGTAGTGGTGCACGCCTGTAGTCCCAGCTACTGGGCTGAGGCAGGAGAATCACTTG	*Alu*Sg	Cruciform (7-bp, 3-bp)

116	AACCCGGGAAGCGGAGGTTGCAGTGAGCTAAGATAGTGCCATCGCACTCCAGCCTGGGCAATAGAGCAAGACTCCACCTCAAAAAAAAT**AATAAAT**AAA<br>**TAA**TAAAAAACAAAACTTACAAAGTTTCCACTACAGCTGAATATAACCCCAAAGCAACTGTTTTAAAATTTTAACATGATTAAGAGCTCACAAAATGCCA<br>AAAATTCATCAAGTTTGTATTTCAATACTAAGCCACCCAAAAGCTTAGGGAGTATTTCCTTCACAACCCTCCAAAACAACAGTGACTGATATATTTTCCT	–	DR (10-bp, 1-bp) (underlined);<br>Triplex motif (13-bp, 1-bp) (grey);<br>STR (13-bp) (bold);<br>Cruciform (6-bp, 3-bp);<br>Cruciform (6-bp, 0-bp)
117	CCAGAGTTTAGTTACTAAAAAGACATATGTAGAGGCCAGGTGCGGTGGCTCATGCCTATAATCCCGGCACTTTTGAAGGACGGATCACTTGTGTCCAGGA<br>GTTCAAGACCAGCCTGGGCAACATGGCAAAACTCTGTCTCTAGTAAAAAATACAAAAAATCAGCTGTGTGTGGTGGCATGGCTTGTAGTCCCAGCTGCTT<br>GGGAGGCTAAAGTGACAGGAACACTTGAGCCTGAGAGGTTGAGGCTTCAGTGGGTGGTTTTTGCACCACTGCCCTCCAGCCTGGGTGACAAAGTGAGACC	*Alu*Jb	Triplex motif (6-bp, 1-bp)
118	CTGCCTCAAAAATAACCAAACAAACAAAAAAGAAAACCACATATATAAAACACAAAACTATTTGCTAACAAGAACATACTGATTAAAAACTGGCCAGGTA<br>CAGTGGCTTACGCCTGTAATCCTAGGATTTTGGGAGGCTGAGGCAGGTGGATTGCCTGAGCTCAGGAGTTTGAGACCAGCCTGGGCAACATGGTGAAACC<br>CTGTCGCTGCTAAAAACACAAAAATTAGCTGGGTTTGGTGGTGCCTGCCCGTAATCCCAGCTACTCGGAGGCTGAGGCAGGAGAATCATTTGAGCTCAGG	*Alu*Sx	STR (12-bp);<br>Cruciform (6-bp, 0-bp);<br>Cruciform (6-bp, 0-bp)
119	AGGTGGAGGTTGCAGTGAGCCGAGATCACGCCACTGTACTCCAGCCCAGGTGAGAGAGCAATACTCTGTCTCGAACAAACAAACAAACAAAAAACACCAC<br>AAAAAAAAACAGAAAAACATATTTATCAAAAATAAAAGACAGCCGGGTACAGCGGCTCATGCCTGTAATCCCAGCACTTTGGGAGGCTGAGGCAGTTGAA<br>TCACGGAGTCAGGAGTTCAAGACCAGCCTGGCCAACATGGTGAAACCCCGTCTCTACTAAAAATACAAAAAAATTAGCTGGTCATGGTGGCGGGCACCTG	*Alu*Sg7	STR (18-bp)
120	TAATCCCAGCTACTCAGGAGGCTGAGGCAGGAAAATTGCTTGAACCTGGGAGGTGGAGGTTGCAGTGAGCCGAGGCCGTGCCACTGCACTCCAGCCCAGG<br>GGACAGCACGAGACTCTGTCTCAAAAAAAAAAAAAAAAAAAAAAAAAGGCCAGGTGCGGTGGCTCACGCCTGTAACCCTTGCACTTTGGGAGGCCGAGGC<br>GGGCGGGTCATGAGGTCAGGAGATCGAGACCATCCTGGCTAACATGGTGAAACCCCGTCTCTACTAAAAATACAAAAAATTAGTTGGGCTTGGTGGCGGG	*Alu*Y	Polypurine tract (25-bp)/<br>Triplex motif (12-bp, 0-bp)/Slipped motif (12-bp, 0-bp)
121	CACTTGTAGTCCCAGCTACTCGGGAGGCTGAGGCAGGAGAATGGCGTGAGCGGGGGAGGCGGAGCTTGCAGCAAGCTGAGATTGCGCCAGTGCACTCCAG<br>CCTGGGCGACAGAGCGAGACTCCGTCTAAAAAAA**AAATAAAAATAAAAATAAAATAAA**GACAAAGATAAAAAATAGTCAACTGAAATTGAAAACGCTAA<br>TCAACATATTTTTTCTCAGTTTCTGTGAAATGTATATTACATTAGCTATTTGTCTTTGTACTGACACCAAGACATCAGTCATTAGTAATAATGGAAACAG	*Alu*Y	Cruciform (7-bp, 1-bp) (green);<br>Polypurine tract (10-bp);<br>Slipped motif (12-bp, 0-bp)/<br>STR (27-bp) (pink);<br>Triplex motif (11-bp, 1-bp) (underlined);<br>Triplex motif (12-bp, 1-bp) (bold);<br>Cruciform (6-bp, 3-bp) (green)
122	CAACAATACAGCAGCAGCTATTATCTACTGAGTACCTGTATGCCAGGTAAATCTGTACCTATGGTCTCATTTAAACTCTACCATAACCCTGTGAAGAGAA<br>CCTGTGTAGATGAGTAACATGGGAGTCAGAGAGATGAAACTGGCCATATCACAAAGTTAGTGAAGTGGCAGAGATTTTGAATTCAGTCTGACTCAAGAAT<br>GGACTTGTAAGCACTGCTATGTTTCTCCTTGTTTCTCTTACACTTAAGACTTTTTTAGAAAAAAAAGTGCTTAATTGAGAACTTTCAAACAGAAAACGTA	MIRb	STR (10-bp);<br>Cruciform (8-bp, 4-bp)
123	TATTATATGATTTGGTAATCTGTTACAGTAGTGGCCCCCAATGAACCAGGAATTCACATTTCCAACTACTCGTGGCCTTGTGTGGTCCTCTCCCCTAGAA<br>TCTGGGCTGGGCCTGTGACTAGCAATGCAGTCACACGTGCACAGCAATAGAGTGAAACTGATCCTGTGCCAGTTCCAGGCCTCATCCTTCAGAAGACCTG<br>TCAACATCTGCTTTTTGGAGGCCACCATGAAAGAAGTATGTCTATCTTTCTGGAGAGGACAAGGGGAAAGAGACAGGAAGGTGAAAAGGCAGGAAGGGAG	MLT1H2	Z-DNA (12-bp)
124	GAAGACAGGGAGAGAGACTGGCACCAAAGCCCAGGTATGCCAGTGTTCCAACTGAGACTAGCTAATACTAAGTCTCCAAATATAAATGAATGTTCTCTGT<br>CAGACATTCCAGCCACAACTGCAACCACAAGAGTGTTGCCAAGCAAAACCAGGAGAACTACCTATCTGAGCCCCAGTCAACTTCCAGAATTGTAAGCAAA<br>TAAAATGGTTGCTTTTTAAGTCATTAAGCTTTGGCACAGTTTTATTACGTAGCAGTAAACAAATCATATAAAAAAATACACGCTTAATTCTTGATTATCT	MLT1H2	–
125	ATACTAATAGAGTGCACATCATCAAGACCAAGACAATCGAAAAAAGAATGTCTGCCAAATTACACCCACAACATTCATAAAGGCATTCATTCAACATTTA<br>TTGAGGCCTAGTATGTGTCAGAAATTAGGGTTATAAAGATGAATAAAGCATGGTCTTCACCTCAAAAACTCTCATTCTGGTAAGGGGGATGGCAAGCAAA<br>AGTAACTATAATGAAATGAATTATGTTCTTACATATCTGGGGCAGCCACAAACTGCCATCAGAGCATAAAAGAGCATTATGATGGGAAAGAGAGTAGGAG	L2c	–
126	ACATTTAAAGAATAAGTTGTCAGTTGGAGGAGCAGGGGATGTCACCTTGAATAACAATAATATTTAGTATTATTATGGCTGACATTAATTAAGAATCTCC<br>TATGTCCTAGGCACTGTGCTAAGCGTTTTTTTTTTAATAACTATAAGTTTTAGGGTACATGTGCACAACGTGCAGGTTTGTTACATATGTATACATCTGC<br>CATGTTGGTATGCTGCCCCTATTAACTCGTCATTTAACATTAGGTATATCTATCTCCTAATGCTAT**CCC**TCC**CCC**CTC**CCC**ACA**CCC**AGTGCTAAGCATT	L1PA4	Polypyrimidine tract (10-bp);<br>QC (21-bp)
127	TTAAATAAGTTTCTCATTTTAATTCCCACAATGGCCAGGTAAAGCAGATACTACCCTTATCCTCATTTTACACAGAAGGAAACTGAAGATTAGGAAAATT<br>TAGTTTCTCAAGCTTTCACAGCTAATACGAACAACTAAAATAGCTGCAAATATTAGTGACTCACTGATAGCCCTCAATAATAAAAAAAGGCTGGGTTGGG<br>TGTGGTGGCTCTCACTTGTAATCCCAGCACTTTGGGAGGCTAAGGCGGGCAGATCACTTGAGGCCAGGAGTTCAAAACCAGCCTGGCCAACACAGTGAAA	–	–

128	CCTCGTCTCTATTAAAAATACTAAAATTAGCTGGCCTTGGTGGCGCATGCCTGTAATCCCAGATACTCGGGAGGATGAGACAGAAGAATTGCTTGAATCC<br>AGGAGGCAGAGGTTGCAGTGAGCCGAGACTGAGACACTGCACTCCAGCCTGGGTGACAGAGCAAGACTCCGTCTCGAAAAAATAATAATAATAGTAATAA<br>CAATAAAAGGCTGTCTTATATTTGTTTGGCTTTTTAGATGACAAATTAGTCATATTCTTTACCAGATTTATAGCAATAAGAATAGTTATCGGCCAGGAGC	*Alu*Sz	STR (13-bp)
129	AGTGTCTCATGCCTGTAATTCCAGCACTTTGTGAGGCCGAGGCAGGCGGATCACCTAAGGTCAGGAGTTGGGAGACCAGCCTGGCCAGTATGGCGAAACC<br>CTGTCTCTACTAAAAATACAAAAAAATTAGCCGAGCGTGGTGGTGGGAGCCTGTAATCCCAGCTACTCAGGAGGCTGAGGCAGGAGAATCGCTTGAACCC<br>GGGAGGCAGAGGTTACGCTGAGCTGAGATCGTGCCACTGCACTCCAGCCTGGGTGACAGAGCAAGACTCCACCTCAAAAAAAAAAAAAATAGTTCTCTAT	*Alu*Sx1	STR (10-bp);<br>Polypurine tract (14-bp)
130	CAAAATTATATACATTACAATCTACACTAAAACAAAATTGCCGGGCATGGTGGCTCATGCCTGTAATCCCAGCACTTTGGGAGCTCGAGGCAGGTGGATC<br>ACCTGAGGTCGGGAGTTTGAGACCAGCCTGACCAACATGGTGAAACCCCATCTCTACTAAAAATACAAAATTAGCCGGGTGTGGTGGCACATGCTTGTAA<br>TGCCAGCTACTCGGAAGGCTGAAGGAGGAGAATCACTTGAACCCCCGGGAGGCGGAAGTTGCCGTGAGCCAATATTGCGCCATTGCACTCCAGCCTGGGC	*Alu*Sp	Cruciform (6-bp, 3-bp)
131	AACAAGTGCGAAACTCCGTCTTTAAAAAACAAAAAAATCTACACACAAAAACATACCTCTATAACATTCACACACATTTTTTTCTTTCTTTTTTCTTTCT<br>TTCTTTTTTTTTTTTTTTTTTGAGATGAAGTCTTGCACTATTGCCCAAGCTGGAATGCAGTGGCGCAATCTTGGCTCTGTGCAACCTCTGCCTCCCAGG<br>TTCAAGCAATTCTCCTGCCTCAGCCTCCCGGGTAGCTGGGATTACAAGCACCCACCACCAAGCCTGGCTAATTTTTGAATTTTTAGTAGAGATGGGGTTT	*Alu*Sx1	Slipped motif (11-bp, 0-bp) (pink);<br>DR (14-bp, 1-bp) (bold);<br>Triplex motif (15-bp, 4-bp) (blue letters); STR (15-bp) (underlined);<br>Polypyrimidine tract (19-bp)
132	CAGCATGTTGGCCAGGCTGGTCTCGAACTCCTGACCTCAGGTGATCCACCCGCCTTGGCCTCCCAAAGTGCTGGGATTACAGGTGTGAGCCATTGCGCCT<br>GGCCAACTTTTTGTATTTTTAGTAGAGATGGGGTTTCACCATGTTGGCCAGGCTGGTCTTGAAACCCTGACCTCAGGTAATCCACCCAACTTGCCATCCC<br>AAAGTGCTGGTATTACAGGAGTAAACCACCCTGCCCGACCAGAAAATGGCTAGTTTCTTCTTTTTTTTTTTTTTTTTTTTGAGATGCAGTCCCACTCTGT	*Alu*Sq10	IR (11-bp, 79-bp);<br>IR (11-bp, 35-bp);<br>Polypyrimidine tract (20-bp)/<br>Slipped motif (10-bp, 0-bp)/<br>Triplex motif (10-bp, 0-bp)
133	TGCCCAGGCTGAAGTGCAGTGGCGCAATCTCGGCTCACTGCAACCTCTAACCCCCAGGTTCAAGTAATTCTCTTGCCTCAGCCTCCTGAGCAGCTGGCAT<br>TACAGGCGCACCACCACACCCACCTAACTTTTTTGTATTTTTAGTAGAGACGGGGTTTCACCATGTTGGCCAGGCTGGTCTCGAACTCCTGACCTCAGGT<br>GATCCGCCCGCCTTGGCCTCCCTAAATCCTGGGATTACAGGTGTGAGCCACTGAGCCCGGCCAGAATGGCTTGTTTTAATTCTCCTTGACCTCCTTCTAT	*Alu*Sx	–
134	ATTTCTGGGGAGTTTTAAACTCAACCAGCAGCTTTCAGAGTACCATATTTAAAGATGATTTCTGTACATTGGTGTTGTTCAGGAAATAAAAAGTTAAAAA<br>AAAAAAAAAGACAATTTGTAGGCCGGGCATGGTGGCTCACACCTGTAATCCCAACACTCTGGGAGGCCAAGGCGGGCAGATCACGAGGTCAGGAGTTCGA<br>GACCAGCCTGGCAAATATGGTGAAACTCCGTCTGTACTAAAAATAGAAAAACTAGCCGGGCATGGTGGCATGCACCTGTAGTCCCAGCTACTCGGGAGGC	*Alu*Sg	Cruciform (6-bp, 1-bp);<br>Polypurine tract (14-bp)
135	TGAGGTGGGAGAATCACTCGAACCCGGAAAGCAGAGGTTGCAGTGAGCCGAGATCGCACCACTGCACTCTAGCGTGGGTGACAGAGCAAGACTCCATCTC<br>AAAAAAAAAAAAAAAAGACAACTTGTAAAAAGATTTATTGGTGAAAAGACTGACTCAAAAACCAATGTAAATATGTGGTCTTTTGCAAACAAATACAGTC<br>AAGCATCACATAATGATGTTTTGGACAATGACAATCACATACATAATGGTAGTCCCATAACATTATATTACTATATTTTTATGTCTTTACTGTATCTTCT	–	Polypurine tract (16-bp)
136	TTCTTTCTTTTTTTTTTTTTTTTTTTTAGACAGGGTCTAGTTCTGTTGTCGAGGCTGGAGTACAGTGGTGTGATCATGGCTTACTGCACCCTCAGCCTCC<br>TGGGCTCAATAAATCCTCTCACCTTTGCCTCCCGAGTAGCTGGGACCACAGGCATGCACCACCACACCTGGCTAATTTTTAATTTCTTTGTGGAGATGGG<br>GGTCTCCCTATATTGCCCAGACTGGTCTCAAACTTCTGGGCTCGAGATCTTCCCGTCTTGGCCTCTCAAAGTGCTGGGACTACAAGTGTGAGCCAGCATG	*Alu*Jr	Slipped motif (10-bp, 0-bp)/<br>Triplex motif (10-bp, 0-bp)/<br>Polypyrimidine tract (20-bp)
137	CCTACTGCACCTTTTCTATGTTAGATGCACACTTACAGTTATGTTACAACTATCTACAGTATTCAGTATAGTAACATGCTGTACAGACTTGTACCCTAGG<br>AGCAATAGAATATACCATATAGCCTAGGTGTGTAGCAGATGATACCATCTAGGTTTGTACAATGACAAAATCGCCCAATGACACATTTCTGAGAATATAT<br>CCCTGTTGTTTTGACTGTATATGTGTGGCTTTTATTTTTACCCCCCTTATTTGCAATTAGTTTTGCTTCCAGTATTTTGGGGTACCAGAATCAATACATT	–	–
138	GCAACCTACCACCAACAAATGTTCCTTTAAATATCTACAATAGACTCCACCTCTGTATTAAAAATGAGGCTGTGGGACAGGCACAGTGGCTCACGCTTGT<br>AATCCTAGCACTTTTTGAGGCCGAGGTGGGTGGACAGCCTGAGCTCAGGAGTTCAAGACCAGCATGGGCAATATGGTGAAACCTCCTTCTACTAAAAATA<br>CAAAAAATGAGCCGGGTGTGGTGGCATGCGCCTATAATCCCAGCTACTCAGGAGGCTGAGGCAGGAGAATTGCTTGAACCCGGGAGGTGGAGGTTGCAGT	*Alu*Sx	Cruciform (6-bp, 0-bp)
139	GAACCAAGGTGGTGCCACTGCACTCCAGTCTGGGTGACAAAGTGAGACCGTCTTCAAAAAAAAAAGCTTTGCATGGGAAAAATATTCTTGTAAATGCTTA<br>TGATGTTACCAAAATTCTTATAATGCTAACAAAATGCTTATAATGTTAATAAAAATTAAAAATTGATACAAAGTTGCATATACAGTAGTCTCTCAACTTT<br>GCAGAATAATATGTATAGAAAGATACATCAAAATGTTAACTGCAGGCCAGGCAGGGTGGCTCAAGCCTGTAATCCCAGCACTTTGGGAGGCCGAGGAAGG	–	Polypurine tract (10-bp)
140	TGGATCATGAGGTCAACAGATGGAGACCATCCTGGCCAACGTGGTGAAACCCCATCTCTACTAAAAATACAAAAATTAGCTGGGTGTGGTGGCAGGCGCC<br>TGTAGTCCCAGCTACTCAGGAGGCTGAGGCAGGAGAATGGCTTGAACCCAGGAGGCAGAGGTTGCAATGAGCCGAGATGGCGCCACTGCACTCCAGCATG<br>GCGACAGAGTGAGACTCCATCCCAAAAAAAAAAAAAAAAAAAAAAAGTTAACTGCAGTTGTCTGTAAGTAGTAGGTTATTTTAAAACTATTTCATCTGGG	*Alu*Sc	Polypurine tract (23-bp)/<br>Triplex motif (11-bp, 0-bp)/<br>Slipped motif (11-bp, 0-bp)
141	CTGAGTGTGGTGGCTCATGCCTGTAATCCCAGCACTTTGGGAGGCCGAGATGCGCGGATCACCTGAGGTCAGGAGTTTGAGACCAGCCTTCACCATGGTG<br>AAACCCCGTCTCTACTAAAAATACAAAAATTAGCTGGGCGTGGTGGCGGGCACCTGTAATCCCAAGTACTCAGGAGGCTGAGGCAAGAGAATTGCTTGAA<br>CCTGGGAGGTGGAGGTTGCAGTGAGCCAAGATTGAGCCATTGCACTCCAGCCTGGGCGACAAGAGCGAAACTTCATTAAAAAAAAAAAATTATTTCATCT	*Alu*Sq2	Cruciform (7-bp, 0-bp);<br>Polypurine tract (12-bp)

142	GTTCTTGGTTTTCCCTGTATCTGGATTTAACCTTTTCTTAGAATACTATAATAGGGACACTTTAAATACTACTACTGTAACAAATTATACACAGATTTTT<br>GTAGTGTTTTCAAAATGGGTAACAGAAGGGGAAGTGGGAGATAAAAGGAGCAATAATGGCTTTCTTTTCTTTTCCTTTTTTTTTTTTTTTTGAGACAGTT<br>TCACTCCTGTTGCCCAGGCTGGAGTGCAGTGGCATGATCTCGGCTCACCGCAACCTCCGTCTCCTGGGTTCAAGCAATTCTCTTGCCTCAGCCTCCCGAG	–	STR (10bp);<br>Polypyrimidine tract (16-bp)
143	TAGCTAAGATTACAGGCGCCCACCACCACACCCGGCTAATTTTTGTATTTTTAGTAAAGATAGGTTTTCATCGTATGGGTCAGGCTTGTCTCAAACTCCT<br>GACCTCAGGTGATCCGCCCGCCTCAGCCTCCCAAAATGCTGGGATTACAGGTGTGAGCCACCACGCCTGGCCAACAGCTTTCTTTTCTTTGCTAAGCTAC<br>TGAGACTTCCTGTCTTGGTTTTTTGAAATAACTTTTGAGTGTTTTTTTTTTTTTGAGACAGTCTTCTTCTGTTGCTGAGGCTGGAGTGCAGTGGTGCCAT	*Alu*Sq	STR (10-bp);<br>Polypyrimidine tract (13-bp)
144	CTTGGCTCACTGCAACCTCCATCTCCCAGGTATAAGCAATTCTCCTGCCTCAGCCTCCAGAGTAGCTGGAATTACAGGTGCATTCCACCACACCTGGCTA<br>ATTTTTTTTACATTTTTAGTAGAGACAGAGTTTTGCCATGTTGAAGGCTGGTCTCAAACTCCTGATCTCATGTGATCCACCCGCCTCGGCCTCCCAAAGT<br>GCTGGGATTACAGGCGTGAGCCACTGAGCCCAGCCACCAACTAGTATCTTAATTAGGTACCAGGATCTTACCATTTGCTCTTGAAGGAGCAGAGACTTGC	*Alu*Sx	–
145	TTTTGTCAGTGTCCTGAGAAATACTGAATCAGAATACCAAAAGGTACATGAAAGCCAACATAGTCATTTTATAGAGTGAACTACAACACACAGACTAAAA<br>TACTGGCATTTCTCACTGTAGACACCTCCAGTACCTCAACTAATTCCCCTTTGATAATGTACCAAGATGAAGTTTTTGGACTTACAAAGATTAAAGACCA<br>GGCCCTGACTTTGGCATTGTCATCAAGGTGGTGCCACTCTGTGAGGCCTGAGTGGCCAGCTGACATCACTACGTGACATTTTGATCAGTTATCTCTACTT	–	–
146	ACGTTTTTGTTTTTGAGATGGAGTCTCGCTCTGTCGCCCAGGCTGGAGTGCAGTGGTGTGATCTTGGCTCACTGCAACCTCCGCCTGCCCGGCTCAAGCA<br>ATTCTCCTGGCTCAGCCTCCTGAGTAGCTGGGACTACAGGTGCACGCTACCACACCTGGCTAATTTGTATTTTTTTAGTAGAGACGGGGTTTCACCATAT<br>TGGCCAGGCTGGATCTCTACTTATAACAAACAGAAAGGCCAATGCTGTAGAAATAGGACAAGTGATGAAAGATATAATAGAAGTGAAATATAAAAATGAA	*Alu*Sx4	–
147	TACACAAATAGAACTTATATTTTTCTTACTTGTTTTGTTTTTAGGGGTAGTAACCTAAGTATCAGCCACCAAAGGAAAGAGGAAAGCATAAATCCATGAG<br>GGCAGTGATCTATTTCCTTGAACACAGAATCTAGGACCTAGAAGTTTCTCAGCCCTACAGTAGGCACCCAATAAATATTTATTGAGTGAATGAACAAATG<br>AATATTTAAGGACAGTGGGAGTTAATTTCTTTGGTATTTACATCTTAGTACCAAGGATTCCAGTGAGCTATAGAGAACTATTCTTAATGATTTCAACATC	L2a	Cruciform (6-bp, 1-bp);<br>Cruciform (8-bp, 0-bp)
148	AGCTAGGCCAAGTTGGCTGTAAACATTATCTGATTTCCTGAAGACAATGAACAGCTTTCTAGCCTTTAACTTATTCAAACATTAAGAGTTAACAGACAAT<br>CCTTGCATTCCTACACAGAACTTCCTTTTCTCTGAAAACAGAGGGTAGAGCCCAAACTTAACCACAAGAGGAGAAACTGCTACATGTTGGCAGTTTTGAC<br>AGAATAACTTAAAAACATTTTTATTCTTCTTCCTTCTAAATGTTCATACAACATGATTTCTCAATACGAGACAAATTTCATGCTAGAGTTTAAAGGATAA	–	Cruciform (6-bp, 2-bp)
149	GAAACTAAAGCAAAGACAAAAGGTAGGCCAGATGCAGTGGCTCACACCTGTAATACCAGTACTTTGGGAGGCCGAGGCAAGAGGATTGCTTGAGCTCAGG<br>AGTTAGAAATCGGCCTGGCAGCTGGGCACGGTGGCTCACCCAGGCACGGTGGTAATCCCAGCATTTTGGGAGGCTGAGGTGGGCAGGATCACCTGAGGTG<br>AGGACTTCGAGGCCAGCCTGGCCAACATGGTGAAACCCTGTCTCTAGTAAAAATACAAAAAGTAGATGGGCATGGTGGCAGACGCCTGTAATCCTGGCTA	*Alu*Sx1	DR (10-bp, 8-bp);<br>Cruciform (6-bp, 1-bp);<br>Cruciform (6-bp, 1-bp)
150	TTCGGGAGGCTGAGGCAGGAGAATCTCTTGAACCCAAGAGGCGGAGGTTGCAGTGAGCCTAAGGTTGCAGTGAGCTGAAGGTTGCAATGAGCCAAGATTG<br>TGCCACTTCACTCCAGCCTGGGCAAAAGAGCGAGACTCTCTCAAAAAAAAAAAAAAAAAAAAAGGAAGAAACTGTCTGGGAAACACAGGAAGACATCATC<br>TCTACTAAAAAAAAATTAAACAAAATTAGGCAGGTGTGGTGGTGTGTACCTATAGCCCCAGCTACTTGGGGATCTGAGACAGGAGGATGGCTTGAGCCTAG	*Alu*Sx1	Cruciform (6-bp, 4-bp);<br>DR (14-bp, 3-bp);<br>Polypurine tract (21-bp)/<br>Triplex motif (10-bp, 0-bp)/<br>Slipped motif (10-bp, 0-bp)
151	CAACATTAATTAACTTCAAAAATATAAATTTGAAGGGGAAAAAATCAGGTCACTGAAGCATATATATAATACAATATGATTCCATTTGTGTACATTCTAA<br>AAGCAGAAAAAATAATAATAAATTGTTTTAAAATACATGTGGTAAAAACTATACGGAAGAGTAATCGAAAAATCAACATAAAATTCAGAATAGCGGTTAC<br>ATGGAGACTGCAACAGAATACACTTAAATAGCAGCCAAAGGGAGCATCCCAGGCATTAAATAATGTTCAAACTGTTCACCGAAGTGCTGGGCACCATATT	L1ME3D	STR (11-bp);<br>MR (10-bp, 38-bp)
152	CTTTAACATATGTTTTGTTTTGTTTTGTTTTGTTTTTAGACAAGGTTTGCATTGTTGCCAGGCTGGAGTGCAGTGGCATGATCAGGACTCACTGCAGCCT<br>CGACCTCCCCGGCTCAAGTGATTCTCCCACCTCAGCACCCCCCAAGTAGCTGCAACTACAGGTGCACACCACCACACCCGGCTAATTTTTTTTTTTAATTT<br>TAG**TAAATTAAATTAAATT**TTTAAAAAAATTTTTAGATTTTAGTTGCCCAGGCTAGTCTCAAATTCCTGAGCTCAAGCAATCCTCCTGCCTTGGCCTCCC	*Alu*Jb	DR (11-bp, 4-bp) (pink); Triplex motif (11-bp, 1-bp) (underlined);<br>Triplex motif (12-bp, 0-bp) (bold);<br>STR (26-bp) (green letters);<br>Polypyrimidine tract (10-bp) (blue letters); STR (16-bp) (bold);<br>Cruciform (8-bp, 4-bp);<br>Cruciform (8-bp, 2-bp);<br>Cruciform (6-bp, 2-bp) (underlined)
153	AAAGTGCTAGGATTACAGGCATGAGTGGTAGCTAACATATGTTTTATGTATTATTTATGTATGACACACAATAAAAGCAAATACACAAAGGGTTGAAGCA<br>AGCGTTGGAGACTGAGGGCCCAAAAAAGTTCCATTTACATAATAAAACTGGTGTGTTTTAACAGTAACTATGCCAGCTCTAAAGGGTATTGGCAGGCCAG<br>ACAATTTGAGGAGAAAAAGGATATATTAATCTAATCATATGTTTATTAACTTATTTTCTTAATATACAATTTTAAATCAGAGGTTTTTATTTGTAGACTT	–	–

154	ATTAAAAAGAGAACATGGCTTAAAATATCCAGAATTGATGAATCATCAGATACAGAAAGCACAATTAATCCTTAGAAAGGGAAGTAAAAATGAAATTCCA<br>GGCCGGTCGCAGTGGCTCACGCCTGTAATCCTAGCACTTTGGGAGGCTGAGGCGGGCGGATCACGAGGTCAGGAGATTGAGACCATCCTGGCTAATACGG<br>TGAAACCCCGTCTCTACTAAAAAAAAAATACAAAAAATTAGTCGGGCTTGGTGGCAGGTGCCTGTAGTTCCAGCTACTCGGGAGGCTGAGGCAGGAGAAA	*Alu*Y	Cruciform (6-bp, 1-bp); Polypurine tract (10-bp)
155	GGCGTGAACCCGGGGAGTGGAGCTTGCAGTGAGCCGAGATCGCGCCACTGCACTCCAGCATGGGCGACAGAGCGAGACTCCGTCTCAAAAAAAAAAAAAA<br>AAAATTGAAATTCCACCTAGGATGGCCACAAAATTTATCATTCTAACTAGGATATTTTTTAGAGTAAATAGAGCACTATTAATAATTCTGAAAGGGTGCC<br>AGGTGTAAACCAGGACTGTCCAGGGAAAATCAGTAACATATCATCCTCTGGAGAAATAGCAGTCACAGAACGTGTTCCTGCGCATTCAAGGTAAACGGAG	–	Polypurine tract (18-bp)
156	ATGGACTTCCCAGAGGACGCACGCTCAGGGCAGTGGCCTTGAGGACTCCAAGCCGGGATGGTGCTAGGCCTTTCAAGTCGCCCGAGAGCAAAGTGGTATC<br>AGGATAGCACAGAGCAAAGAAGATAAAGAAACTCAAGAGCCCATCAGGTGTCCCGCCCCCAGGTGCGACGGCCAGGGCCACATCACCGGGTAGTACGCGT<br>CCCACTGTAGCGCCTCCGGGTGCCCCTTGGCAGCCAAGCGGACCTGAATGGCTCCCAGTTCTCCTGGAAGTCGGTCAGGACGGAAGGCATGTCCTGCTCC	–	Cruciform (6-bp, 1-bp)
157	ACGGCGGGGTGTAACGGCTAGGGCCACGTCAGCAGCAGCTTCCTCACACACCGCAGGCAGTTCCTGTCTGAAAAGCACCCCTATTTATACATATGGATCC<br>CAGTGTCTCCCAGAACCCTGCATGAGGACAGGGGCCTTGGCTGCTCACCAGGACCCCTCAGGGCCCAGCAGGCCGGGGCAGGTGCACGGAACCCCGTAGC<br>GTCAGGGAGCCGCCTCATCCTTCCCAGTGAGTGTGCTCCTGCGCGGGCCGCCCCATGTCCTGTCCAGGTTTGATGATTACAAAGAACTACATGTGAACTT	–	STR (10-bp)
158	CCTTCTGCACATAGCTCCATGCCCTTGTGCAATCAGATCTTCAGGATCTATTCCTGGAAACAAGAATGCTAGCTTTAAAATTTTGATAGATCGTGCCAAA<br>AAGGCCTCCAAAAAGTTTCTACTTACCCTAACCTTTCGCCATTAACAAAATATTTTAAAACAAAACAAAACAAAACAAAAAA**AAAGAAAGAA**AGAAAAAG<br>AAGAAGA**AAAGAAAGAA**ACAGCAGTCACAGAGAAAATACTGATTGCCAGTACTGGCTGACAGCAGACTTCTCAACGGCAACAATAGATGTCAGGATAAGA	–	STR (24-bp)/Slipped motif (10-bp, 0-bp)/Triplex motif (12-bp, 0-bp) (yellow); Triplex motif (12-bp, 8-bp) (bold); DR (11-bp, 10-bp) (pink); STR (15-bp) (underlined); STR (11-bp) (yellow); Cruciform (6-bp, 0-bp)
159	TGAGAAAATATTGGCCAGGTGCGGTGGTTCCCTGAGCGTGCATCCTCTGCGCCTGTAATCCCAACTACTTGGGAGGCTGAGGCAGGAGAATGGCGTGAAC<br>CCGGGAGGCGGAGCTTGCAGTGAGCTGAGATCGCGCCACTGCACTCCAGCCTGGACGACAGAGGGAGACTCCATCTCAAAA**AAATAAATAAAT**AAAAAAT<br>AAAAAAGATGGGAAAATATCTTCAAGTTACTCAAAGAAAATAATGGTCACCTAGAATTCTATCCCCAGATAGATGTTATATGAAAGAAAATGACTTTTGC	*Alu*Yc	Cruciform (6-bp, 4-bp); DR (10-bp, 5-bp); Triplex motif (10-bp,1-bp) (underlined); STR (15-bp) (bold); Cruciform (6-bp, 4-bp)
160	TCAAATGAAGAAAGAGTGTTTTTTACCATTACGTACCAATGTGCAAAGAACTAAAGGATATACTTCAGGGAGAAGAAAATTGAATATAGAAAAAGGAACA<br>AGAAGCAATAAACAACGATGAGTGAACAGACTGGTAGAGTTAAAGATAAAACTGAATAAGCCATGCCATCATAAAACAATATGAATTTTTATAATAAATA<br>ATTTAAGGATATTGAAATAAGATACATTAAAAAACTGAAAAACAATATGTAAGCTAGGAAGAAAATGATCATACTTCAAGTATCAAATAGTTCCTACATT	L1ME2	Cruciform (6-bp, 1-bp)
161	ATCTGGGAAAAATATAAAGATGTTGAATAATTGCTACCCTGTCAAGTAAAGAATACG**TATATATATATAT**GTTAAAGATTTAAGAATAACACTAAAAGGA<br>TAGAAATAGTATTCACAGGTTTCAAATTAGTAGAGGTGAAAAAAAGAAACAAAAACAACTAGATCAACCCAATAAGAGCCAAGAAATGGGGGGAAAAAAG<br>AGGAAAAGCTGGCTGGACACAGTGGCTCATGCCTGTAATCCTAGGACTTTGGGAGGCCAAGGCGGGCGGATCACCTGAGGTCAGGAGTTCAAGACCAGCC	L1ME2	STR (13-bp); Cruciform (6-bp, 0-bp) (bold); Cruciform (6-bp, 0-bp) (underlined)
162	TGGCCAACATGGTGAAACCCCATCTCTACTAAAAATACAAAATTAGCCAGGCATGGTGGCATGTGCCTGTGGTCCCAGCTGCTTGGGAGGCTGAGGCAGG<br>AGAATCGCTTGAACCTAGGAAGTGGAGGTTGCAATGAGCCAAGATGGCACCACTGCACTCCAGCCTGGGTGACAAGAGCAAAACTCCATCTCAAAAAAAA<br>AAAAGGAAAAGCAGAGCACTAGAAAGAACAAAAGTAATATAAATTAGTTCTATATGTCATGCATATTAACCAAAATGAGATAATCAATACAACATATCAG	*Alu*Sq2	Polypurine tract (12-bp)
163	TAATCAGAGTACATTTTCAAATGTTAAAGGATTTACTCGGGAGATAGAGATTCTCTGATTAGATTAAAAAAATAAAAGTTGCATATATGCTGTTTATAAA<br>AGACACACAACTAAAACACAAGGACTCAAAAAAGCTGAAACTTTTATTTAATGATAAAAGATAAACTAGGCAAATATTAATCAAAAGAAAGCTGTTGTAA<br>CAAATCTCTGGCAAAATATATTTTAAGTCCAAAGCATTATTAGAAATAAAGAGTCATGGCTGGGTGTGGCGACTCACACCCTGTAATCCCAGCACTTTGG	L1ME2	Cruciform (6-bp, 2-bp); IR (11-bp, 58-bp); Cruciform (6-bp, 0-bp)
164	GAGGCCAAGGCAGGTGGATCACTTGAGGTCAGGAGTTCAAGACCATCCTGGCCAATATGGTGAAACCCTGTCTCTACTAAAAATACAAAAACATTAGCCG<br>GGTCTAGTGGTTCATGCCTGTAGTCCCAGCTACTCAGGAGGCTGAGGCAGGAGAATCGCTTGAACTCAGGAGGCAGAGGTTGCAGTGAATCGAGATCACG<br>CCATTGCACTCCAGCCTGGGCTATCTCAAAAAAAAAAAAAAATTAAAAAAATTTTAAAAAAAGGAATAAAGAGTCACTATATAATAATTAAAGGAGTTAA	*Alu*Sz	Polypurine tract (15-bp); Cruciform (6-bp, 3-bp)
165	TCAAAAAGGTTGAACATCTCGGAACTTTTATGCTTCCAGTTAACACAACCTCAAAATATGTTAAGCAAAAATTAAATATAAAAAGCAAAAACTGATAGAA<br>TTACAAGATGAAATTTAAAATCTACAAGCATAGTGGGAGAGGTTAACATCTCTTAGAAACTGGAATTTGAAATTGAATTAGAAATAGAAATATCCAAGAG<br>ACAAAAAAACCAAGTTAGGCTGTAGATTAAACACAGTAAGTTTAATCTAATGAACATATGGAGAACTCCACAACCAACAGCTACAGAATATACATTTTTAA	L1ME2	IR (10-bp, 7-bp)

166	GCTTCATGAACATTTTGAAAATTGATTTTGTACTACAATACAAAGCAAATCAAGAACCAATATTATGTAGACTACATTCTCTCATCACAATTCAATTAAA<br>TTCAAAATCAATAACAGCACAATTTTAAAAATTTAATGTGTTTGGGAAATGTAAAAACACACTTCTGAACAACAAATGGATCAAAGAAAAAATCATAATG<br>AAGATTAGAAAATATTTACAACTGAATGATATAATTCTTTGTGTCAAAATTTGTGGAATGTAGCTAAAAGCAGTAATTAGAGGAAAAGCCATATAAGCGT	L1ME2	IR (10-bp, 72-bp);<br>Cruciform (6-bp, 1-bp);<br>Cruciform (6-bp, 1-bp)
167	ATATTAAAAGAAATTGGAAAATTAATTGAATAAATATCAGATTCAAGATTTTCTATAAACTATGTCAGAGTAAATCCAAAGAAAATAGAAGGAAATAATA<br>AAGAAAGGAACAGCTATTAATAAAATAGAAAACAAAGAAACAATAGAAGGGGTAATCAAGTCTAGTTATTTGAAAATACCAGTAGACAGAAATTTGATCT<br>GATTGATCAAAATAAGAAAAGGTACAAATAAATAATATTAGCAATTAAATATCTACAGATATAATAGATACTTTAAAAATAATAAGTAAATACTATGAAC	L1ME2	Cruciform (6-bp, 2-bp)
168	AAGATTATGCCAATAAACTGGGTGAAATGAAAATTTTCACAGAACAAAAACACCTACCAAAACTGACTAAGGGCTGGGTGTGGTGGCTCATACCTGTAAT<br>CCCAGCACTTTGGGAGGCTGAGGCAGGAGGATTGCTTGAATCCAGGAGTTTGAAACCAACCTGGGCAACAGAGTGAGACCTCGTCTCTACAAATACATAA<br>ATTTTTTGAAATAAGCAGGGCATGGTAGTGCATGCCTGTAGTCCCAGCTACTTGGGAGGCTGAGGCAGGAGGACCACCACACTGCAGCCTGGGCAACAGA	*AluJo*	Cruciform (6-bp, 0-bp)
169	GCAAGACCCTGTCTCAAGGAAAAACAAAAAAAAACTGACTAAGAAATAGAGGATCTGAATAAACAAATAATCATTGAAGGAATTAAATTCCTGTTTTAGA<br>AATTTATCCCCACATACACAAACACACAATATGTGGTCCAGGTGATTTTACAAGTTAGCTCTACTAAACATTCAAAAAAGAGATAACCAACTTCATGCAA<br>ACGATTCCAAAGAACGGAAAAGAAGGAAAACCTTCAAACTATTTTATGAGGTAAATATAACCTTGATATCAAAAAGAAACAAGAAAAATATAAGAAAGG	L1ME2	Cruciform (7-bp, 1-bp)
170	AAAATTACAGCACAATCTCACTTTTGAACAATTTGAGATGCAATAATCTTAAATAAAATGTTTTGAAACCAAGCGCTCACTACACATGTACTAAAATTAG<br>AATGACACAGAGTAGATTATCATGGTCCCTGTGCAGGGATGATACACACATTCATGAAGGATTCCATATTTTTTCATCTCGCCCCAGTTAAAATGGCTTC<br>TACGCAAAAGTCAAGCAATAACAAATGCTGGTGAGAATGTGGAGAAAAGGGGAACCCTCGAACACTGTTGGTGGGAATGTAAATTAGTACAACCACTACG	–	Cruciform (6-bp, 2-bp)
171	GAAAACAGTTTGGAGATTCCTCAAAAAACTAAAAACAGAGCTACCATATGATCCAGCAATCCCACTGCTGGGTGTATTCCCAAAAGAAAGGAAAATAGTT<br>CATTGAAGAGATATCTGCACTCCTATGTTTGTTGCAGCACTATTCACAATAGCTAGGATTCAGAGTGTCAATTAACGGATGAATGTACAAAGAAAATGTG<br>GTACATATACACAATGGAGTACTATTCAGTCATAAAAAAGACTGAGATCCAGTCATTTGCAACAACATGGATGGAACTGGAGGTCATTATGTTAAGTGAA	L1MA2	–
172	ATAAGCCAGGCATGGAAAGGCAAACATCGCATGTTCTCATTTATTTGTGGGATCTAAAAATCAAAACAACTGAACTCATGAATGTAAAGGATGGTTACCA<br>GAGGCTAAATGGTAGTCGGGGGCTGATGGGGAGGTGGTGATGGTTAATGCATACAAAAAAATAGAAAGAATGAATAAGGGCTGGGCATGGAGGCTCATAC<br>CTGTAATCCTCATCTCTACTAAAAATACAAAAATTAGCTGGGCGTGGTGCCAGGTGCCTGTAGTCCCAGCTACTTGCAGGCTGAGGCAGGAGAATTGCTT	L1MA2	–
173	GAACCCAGGAGGTGGAGGCTGCAGTGAGCCGAGATCATGCACTGCACTCCAGCCTGGGTGACAGAGCCACTATTTTGATAGCACAACAGGGTGACTATAG<br>TCAATAATAACTTAATTGTACATTTTAATTAAAGAGTGTAATTGAATTGCTTGTAACTCAAAGGAGGAATGCTTGAGGGGATGGATACCCCATTCTCCAT<br>GACATGCTTATTTTACATTGCATGCCTGTATCAAAACATCTCATGTACCCCATAAATATATACCTACTCTGTACCCACAACAATTAAAAATTAAAAAAAA	L1MA1	Cruciform (6-bp, 0-bp)
174	TTTCTTTTAAGTTCACAAACCAATACAGCATTATGTACTATAATAACAACAACCAACATAAACCAGCAAAGTTTATTTCAGGAATAAAAGGATGGCTTAA<br>CATTAGAAAATCTGTTAATATAATTAACAGGAATAGATCAAAAAGGGAAATTATATATAGAAAAATTATTTGAAAAAACTAAGGGCTGGGTGTATATCCA<br>TTTGTGATCTATGCTATGGTTTGACTATCTCTGCCAAAACTCATGTTGGAACCTTAATTCTCAGTGTATCAATATTGGGAGGTGGTACCTTTAAGAGGTT	L1ME2	Cruciform (8-bp, 3-bp);<br>MR (10-bp, 88-bp)
175	TATTAGGTTATTACTGTAGATTAATATCTTTCTCTAGAGACTCAATTAGTTCTTGGGAATGGATTCATTTCCCCAAGCAGGCTTTGTTTTTGTTTTTGTT<br>TCTGAGACAGGTCTCATTGTGTCACCCAGGCTGGAGTACAATCATAGCTCACTGCAGCCTCAAATTTCCGGGCTCAAGTGATCTTCCTGTCTTAGCCTGT<br>AGAGTAACTGGGACTACAGATGCACACCACCACACCTGACTAGTTTTTTTTTTAATTTTGTGTAGAGATGAGGTTTTGCCATGTTACCTAGGCTGGTCTC	*AluJo*	Triplex motif (10-bp, 1-bp);<br>STR (19-bp) (underlined);<br>Cruciform (6-bp, 2-bp);<br>Polypyrimidine tract (10-bp)
176	AAACTCCTGAACTCAAGCAATCCACCTGCCTCAGCTTCCCAAAGTGCTCCCAAAGGGATTACAGGTGTAAGCCACTACGCCCGGCCTAGCAGGTAGTTAT<br>AAAGCAAGGTTGCCTCTCATGTTTGGTCTCTCAGCACATACCCACTTCCTCTTCCGCTTTCCCACCATGCTATGACACAGCACAAGGCCCTCACCAGAAG<br>CCACCCAGATACTGGTGCCATGCCTCTTAGACTTCCCAGCTTCCGGAACCATGAGTCAAGTAAACCTCTTTTCTTTACAAATTACCCATTCTCAGGGATT	MSTB2	–
177	CTATTATAACAACACGAAATGGACTAAGACTATATATATAAATATATATTTTTTTAATTCTTAGCAAACTAAGACTTGAAAAAAACTTTATAACTTTAAA<br>AGGGTGTATCTATAAAAACTTAAAGCAAACATCATACTTCATGGGCAAAATACTCCTCAAAGCATTCTCTTCAAAGTTAGGATCAAGAGCTGCCTATTCT<br>TACCTCCTCTATTGACCATTATTCTGGAGGTCATAGCCAGTGTGTAAAGACCAGAAAGAAAATCAAAGCTGAAAAGTCTTGAAAGGAAAAAATGAAACTG	L1ME2	STR (10-bp);<br>Cruciform (7-bp, 4-bp); Cruciform (8-bp, 2-bp) (underlined);<br>Cruciform (6-bp, 0-bp) (bold);<br>Cruciform (6-bp, 4-bp)
178	TCATTGTCTACAGTTTATAGCATTGTCCATAAAGAAAATCCAAGAGGAGGCCAGCATGCCCATAGTCCAGCTACTCAGGAGGCTAAGGTGGGAGGCTTGC<br>TTAGGCTCAGGAGTCCAAACTTGCAGTGAACTATAATCATGCCACTGCACTCCAGCCTGGGAGATAGAGTGAGACCCTGTTTCTTAAAAAAAAAAAAAAA<br>AATCCAAGAACAGCGCTTTAGAAAAGTGAGAAAAGTCTGGGTGCTGTTGCTTACGCCTGTAATCCCAGCACTTTGGGAGGCTGAGGTGGGCAGTTCCCTT	*AluJr*	Polypurine tract (17-bp)
179	GAGGCCAAAAGTCTGAAACCAACCTGGGCAACATGGCAAAACCCCATCTTTACCAAAAATACAAAAATTAGCCAGGTGTAGTGGCATGCACCTGTAGTCC<br>CAGCTACTCAGGAGGCTGAGGCAAGAGAATCGCTTGAACCTGGGAGGCAGAGGTTGCAGTGAGCCGAGATTGTGCCATTGCAGACAGAATGAGACTCTGT<br>CTCCAAAAAAAAAAAAAAAAAAGTGAGAAAAGGGCTGGATAAACTATTCGTGTGCAAAAATCAACTGTGCTCCTATAATAAGTACGAATGATTTTAAAAT	*AluSz6*	Polypurine tract (18-bp)

180	GTAATTTTTTAAAAATTTCATTTACTATAGCCAAAAACCTGTAAGATACCTAAAAATGAATTCAACAAAAGTTATGCAAAATATTTCCAGAGAAAAAAAT<br>GTATAACTTTATTAAGACTTTTAAAAAACTAAAAAAAAAAAAAGAGAGAAATTATGTTCATGAATGGGAAGTCCCAGGTTCATAAAGATGGCAAACCTCT<br>CTCCAAACAGTGGATCAATATAATGCAAATGCAAACAAAATTTCAACTCTGTGTGTGTGTGCACACAATCTGACAAACTGCTCTTACAATTTATATGGAG	L1ME2	Cruciform (7-bp, 1-bp); MR (11-bp, 37-bp) (grey); Polypurine tract (13-bp) (blue letters);<br>Z-DNA (18-bp) (underlined);<br>STR (12-bp) (bold);<br>Cruciform (6-bp, 4-bp)
181	GAGCTCAGGCCCTAAATAGACAAGTCAATTTTGGAGAACAAAGTGGGATGACTTACCCTATGAGATAGCAGAACATTTTATAAATTATAGTAATTAGCAC<br>AGTGTGCTATTGGCATAGGAATAGAAAAATAAACCAATGGCACAGAACTGAGCCCAGAAACAGGTCTTAGTCTTTGAGGAAACCTCGTCTATGGCAGAGA<br>TGGTATTACAATTGAGGCTGGGAAGGGGTGATAGTCTAGTCAATAATAATGTTGGAAATATTGGTTCCTCAAATGAGGAATAGAGGGAAAAAATAGATTC	L1ME2	Cruciform (7-bp, 1-bp);<br>Cruciform (7-bp, 2-bp)
182	TTACATCACACAATAGACAGAAAGCAATTTCAGGTGAATTAAAGACCCAAATGTAAAATGCAAAGTTTAAAACTTCTAGAAGAAATCATAGAAAAAATAT<br>CAGAGAAAGATTTTTTTTTTTTTTTTAACAGAGTCTAACTCTGTCACCCAGGCTGGAGTGCAGTGATGCAATATCGGCTCACTGCAACTTCCGCCTCCTG<br>GGCTCAAGCAATTCTCTTGCCTCAGCCTCCTAAGTAGGTGGGATTACAGGCGCACGCCACCACGCCCAGCTAATTTTTGTATTTTTTGTAGAGGTGGGAT	*Alu*Sz	Cruciform (6-bp, 1-bp);<br>Polypyrimidine tract (16-bp);<br>Cruciform (7-bp, 3-bp)
183	TTCACAATGTTGGCCAGGCTGGTCTCGAACTCTTGACCTCAAATGATTTACCTGCCTTGGCCTCCCAAAATGCTGGAATCACAGGTGTGAGCCACCGCAC<br>CCAGCCAGAAAAAAGATTTTTTTGTTTTTGTTTTTTTTTGAGACGGAGTCTCGCTCTGTCACCCAGGCTGGAGTGCAGTGGTGCTATCTCGGCTCACTGC<br>AATCTCCGCCTCCCGGGTTCACACCATTCTCTCTCTTCAGCCTCCCGAGTAGCTGGGACTACAAGAGCCTGCCACCATGCCTAGCTGATTTTTTGTATTT	*Alu*Y	Cruciform (6-bp, 2-bp);<br>Triplex motif (10-bp, 1-bp)
184	TTACTAGAGACAGGATTTCACCATGTTAGCCAGGATGGTCTCGATCTCCTGACCTCGTGATCCGCCTGCCTCGGCCTCCCAAAGTGCTGGGATTACAGGC<br>CCGGCCCAGAAAAAGATTTTTTAAGTAATCATAAAGCAGAAAGGAAAAGATTGTTAAATCTGATGACAATGTCCTTAACAGAGTGAAAAGATGAAGCATG<br>TTGTTTCTTATAAAGTTAAATATGCACCATCCCTATGATCCAGCAGTTCCACTTTGAGGTATTTACCCAAGAGAAATGAAAACACATGTCCACAGAAAGA	–	–
185	CAGGTACAAGGATGTTTATAGCCATGTTCCTCACAGTGGCCAAAACCTGAAACAATCTACATGTCCAGCAACAGAAGAATGGATAAACATATTGTGGTAT<br>ATTTATACAATGGAAATCTTCTTAACAATAAAAATGAAATCTACATAACAACATAGATGAATCTTAAAAACATTATGCTGAGCACAAGAAGCCAGACAGA<br>CTTACTGTATTCTTTCTATTACATTAAGTTCTAAATCAGGCAAAATTACTACATATTAATAGTAAAAGAAGTCAAACCAGTAGTTGTCAGGGAAAGGGCA	L1ME2	–
186	CGAAAGGCCTTGCTAGGATGGTGGAAATGTTCTATAACATGACAGAGGAGTGGGTTCATAGCTGCATGTATTTGTCAAAAGCCATCAAACTAGGCAGGGC<br>GCAGTGGCTCATGACTGTAATCCCAGCACTTTGGGAGGCCGAGGTGGGTGGATCACCTGAGGTCAGGAGTTCGAGACCAGCCTGACCAACATAGTGAAAC<br>CCCCATCTCTACTAAAAATACAAAATTAGCTGGGCGTGGTGGCGCATGCCTGCAATCCCAGCTACTTGGGAGGCTGAGGCGGAGAATCGCTTGAACCTGG	*Alu*Sq2	–
187	GAGGCAGAGGTTGCAATGAGTGAAGATCGTGCCCTTGCACTGCAGCCTGGGCAATAAGAATGGAACTCTGTCTCAAAACAAAACGAAACAAAAACACAAA<br>AACAAAAACAAAAAGCCATCAAACTGTAGCAATTAAGATTTGTGTAATTAACTGTATTTGAAAAATCAGCCGGGCATGGTGGCTCACGCCTGTAATCCCA<br>GCACTTTGGGAGGCCGAGGTGGGCAGATCACGAGGTCAGGAGATTGAGACCATCCTGGCTAACACGATGAAACCCCGTCTCTACTAAAAATAGAAAAAAT	L1ME2	DR (11-bp, 3-bp) (pink);<br>Triplex motif (11-bp, 5-bp) (underlined); STR (19-bp) (bold);<br>A-phased repeat (27-bp) (turquoise)
188	TAGCCGGGTGTGATGGCGGGCACCTGTAGTCCCAGTTACTCAGGAGGCTGAGGCAGGAGAATGGCGTGAACCCGGGAGGCAGAGCTTGTAGTGAGCCGAG<br>ATCGCGACACTGCACTCCAGCCTGGGCGACAGAGCAAGACTCTGTCTCAAAAAAAGAAAAAAATATCTCTCAAATCCACTTCCCCAACCCACTGCAAAA<br>AGACAAACTATAGTCTGGGAAAAGGCATTCGCCTGATAAGAGATTCATGTGCAAAATCCACAAAGAACTGCCATAAATCAATTCTGAGGAAAAAAATATC	*Alu*Y	–
189	AAATAGAATAGTCACAAGAATACAAACAGGCAATTTACAGAAGAGAAAACATTTGGAAAGATGCTTAACCTCACCACTAATCAGGAAAATAACACAATAG<br>CACTTCATACCCATTGGATTCACAAAGTTGTTGAAATCTAAGGATACTAAACACTGGTGAGACTGTGGAGAAACTGGAATTCAGACAGTGTACTCTGCTT<br>GTGGGAATGTGGACAGGCAGTGCCTAGGAAAAGTGAAGAGGTCCCTCGCTTGTGATCCAGCCATTCCTGGCAGGTGTGTCTTTAGCTGGGGGGCATTTGT	L1ME3E	–
190	ATCTCTCATATGTGCACAAGGGGAACCCTCACTGCACCACTGTTTGTAACCAAAAAAAAAAAAAAAAAATGGAGGATGGGCAGAAATACAAAAAAGAAAA<br>CACAGAAGCCACGTGTGGTGGCTCACCCCTGTAATCCCAGCACTCTGGGAGGCCAAGGAGGGCAGATCACAAGGTCAGGAGTTCAAGACCAGCCTGGCCA<br>ACATGATGAAACCCCGTTTCTACTAAAAATACAAAAATTAGCCAGGCATAGTGGTGCATGCCTGTAATTCCTGCTACTCGGGAGGCTGAGGCAGGAGAAT	*Alu*Sg	Polypurine tract (18-bp);<br>Cruciform (6-bp, 4-bp)
191	TGCCTAAATCTGGGAGGCGGAGGTTGCAGTGAGCCAAGATCACGCCATTGCACTCTAGCCCTGGGTGACAGGGGAAGACTCCATCTTGGCGGAAAAAAAA<br>AAAAATTAGCCAGGCATGGTGGCAGATCCCTGTAATCTCAGCTACTTGGAGGCTGAGGCAGGAAAATCGCTTGAATCCCGGAGGCGGAGGTTGCAGTAAG<br>CGAAGATAATACCACTGCACTCCAGCCTGGGCGACAGAGACTCCGTCTAAAAACACACACACACACACACACACACATATGGGTCTAGGGGAAAGGCAAA	*Alu*Sx	Polypurine tract (13-bp);<br>Slipped motif (12-bp, 0-bp)/<br>Triplex motif (12-bp, 1-bp)/<br>Z-DNA (25-bp)/STR (25-bp)
192	CTAAATGTCTATCATGTCTATCAACAGGGGAATGGATACAGTATGGTATGTCCACGTAACAGAATAAAAATCATCAGGGAAATGAATTACAGGGTACACA<br>CAATGTCATAACAAAATGTGTTAAAAAACAGGTTACAGTAGACTACAAATTGTATGTTTCCATTTATATAAAGTTTGAAAACACAGCAAAACTAAACAAT<br>CTATTGTTTAGGGATACAAATATATTTGGTATAAGTATAAAGAAAGCATGAAAATCACACACACAATTTGGGATAATGGTTACTGAGGAAAGGGGAGGGG	L1ME3E	MR (10-bp, 45-bp) (underlined);<br>Cruciform (9-bp, 2-bp);<br>Cruciform (6-bp, 0-bp);<br>Z-DNA (10-bp)/STR (10-bp)

193	CTGAGGAGGGCCTCAGAGGGGACTTTCAAACGAATGGTTATATTCTTTTTCCTAAATGGAGTGGTGGGTACGCAGGTATTTGCTGTTGTCGTTAATCCTT<br>AGACACTGCACACATTTTATAAATATATCTAAACATTTATACACGTAATGACAGCAAGTTCTATAGATGTGTATTGAAACTTAGAAGCTTTCAATCATCT<br>TTATATCTTCAAAAAAACATCTGAAGCAAATATGGCAAGTGGTAAGATTGGACAAAGTTGGGTCATGGATAACAGGTGTTCATTAGTCTTTTAAAATATA	–	–
194	TAATAGGGGCTGGGCACGGTGGCTCACACCTGTAATCCCAGCACTTTGGGAGACTAAGGCAGGTGGATCACCTGAGGTCAGGAGTTAGAGAGCAGCCTGG<br>CTAACATGGTGAAACCCCATCCCTACTAAAAATACAAAATTAGCTGGGCATGGTGGTACATGCCTGTAATCCCAGCTACTTGGGAGGCTGAGACAGAAGA<br>ATTACTTGAACCCGGGAAGTGGAGGTTGCATGAGCCAAGATTGCGCCATTGCACTCCAGCCTGGGCGACAAGAGTGAAACTCCATCTCAAAATAAATAAA	*Alu*Sq2	Cruciform (6-bp, 3-bp)
195	TAAATAAATAAATAAAAATAGAATTTTTAATAGTAAATGTTCTTTAAAAAGAAAAACAATATCCCTATTTGAAGATAAAGATTTATCTGTTCTCGTTAGT<br>TTCCAGCCTGGTGGGGGAGATTAAGACAATAATCAAATAATGGTTTTTATGAAATAAAACTACTAACTAAAGCAGTGTTCTAAAGACAATGGAATCACTC<br>CATGGGAGCATAAAATAAAGGAATTTGATCCGGGCTGGAGTGTCAGGTCGGTAGGTAGAGTGGGAATCGGGGCCAGTGAGGGAAGCACACAGATGGTTCA	–	STR (16-bp); Cruciform (7-bp, 3-bp); Cruciform (6-bp, 2-bp); Cruciform (7-bp, 2-bp); Cruciform (6-bp, 3-bp)
196	GCAGTGGGCAGTGAGGAAGACGCCAGGGGAGGAGAGGAAGGGCTGCTCCCTGAAGCCTTCTGCCAAAGGGGATGACCTAGCAGAATTCCAAATTATAGGG<br>CTCAGACAAAGAAGAAGGCAATAAGAGTGTTCTGTGCAGTGAGAGCATCATGTGCTAAGGCAAAGAGATGTGAGAGAAGAAAGTCAATCTGGAGGCATCT<br>GAGTAGTCCAGGAGGCTGGAAAATGGGGTTGGGGTGGAGTGTGAATAAGGGGCAGAATACGAGGCCAAGAGCCAGCCAGGACTGGTCATTAGGGCCTGTA	–	–
197	TTCCATGCAAGGTAGGGACTTAAGCAGGGGTGTGACATCATCTGATGACAGAGAGTGGATTGAGAGGGAGAGCCCGAAGGCAGGGCATCCAGCTGGTGGC<br>TGTGTAGTGATGTAAGGGAGAAAAGATGGAGGCTGAAGTGGAGGCATAAATGAGAAGGTTATGGAGCCTAGGGCTGTTCCTGAACCAGGAGAGGGTGGCG<br>TGTCAGAAGCCAGGGAGGACCGAGCTCAACCATGTCAAAAGCTGCAGAGAGACTGCTGAAACTTGGGACTTAAAGGATCACCTTGATGTGTCAACTAGGA	–	–
198	GATGAGAACAGTTGCTGTGGAATGAGGTGGGGTGGATGCAGAAGTCAGCCTGCAGTGCTGTGGGTGTTTCATGTCAAAGGCTGAGAGACAGGGTTGGAGA<br>AGGCAGCCTCAGGCCAAGGATGGGGGCTCAGAGCTCTCTTCCAGAGTCTTCCACTCCCTGCCCCAGTCCCAACCTGAGCTCACTCCAAGCAATCAACATG<br>AAAACAAAGGGAGAGTGTCTAGCAGAGGCCTTCCCATCTGCAGAGCAAGGAAGTCTCGGTCCCAAGGTCAATGCTGAAGAGGAACTTGCTTGGTGGTGCA	–	–
199	GGTCATGGAGCCAGCAAGATTCTTGATAAACAGGAGAGGATGGTGGCACCGGAGGTGGATGTAGGGGGTTTCGTGAAGTGATTTGGCACTAGGGTCAAGA<br>GGAGGCCAAGGATAAACAATGTTGTGGAAATGAAATGGTGATAATTTACAAGGGCAAATTGTTTTACATTCCAGCGGAGTTGGCTCCCAAGCCGAACTCT<br>TCCTGCAGAAGTTGAGGTGGTGTGTGAGTCATTGTTATAAATAGACCGGCCAACTGAATGTCATCGGAAAGGACACAGTCCCATGAGTATAACCTTGGAG	–	–
200	GGCGCAAATGGCACTGCATGGATTGAGAGCTTGTTCCAAGAAGAGGGCAGAGGGAGAGCTTGGGGCACGGGGGCTGTCCAGGAGAGGAAGCAGATGCTG<br>AAGAAGGGGGTTCCAGCTCAAGCAGCAGGCTGATGCAGAGGACTGGCCAGGAGCAAGCTTCCTGCATGGCTCAGTGGAGCCCAGCTCTCAGCTGCCTAGC<br>TGCTAGCTCAGGCTGGGTCGTCTCACTTTCTCCATCTACTTGAGCTGCCGTAACAGAATACCACAGACCTAGGGGGCTTACACAGCAGGAATCGATTTCT	–	QC (30-bp)

STR (yellow): short tandem repeats of 2–6-bp that are repeated several times. A subtype of STR may also represent Z-DNA.
Z-DNA (red): five or more tandem repeats, each comprising an alternating pyrimidine–purine dinucleotide motif (Wang et al., 1981; Cer et al., 2011).
Polypurine tract (yellow): poly-A or poly-G tracts
Polypyrimidine tract (yellow): poly-C or poly-T tracts
IR (green): inverted repeat of ≥6-bp separated by ≤100-bp.
Cruciform (green): A subtype of inverted repeat of ≥6-bp separated by 0–4-bp.
DR (pink): direct repeat of 10–300-bp with a spacer sequence of 1–10-bp. Slipped motifs represent direct repeats that are not separated by any spacer sequences.
MR (grey): mirror repeat which encompasses at least 10-bp and is separated by 9–100-bp. A subset of mirror repeats, termed "triplex motif", is characterized by a spacer sequence of 0–8-bp between the repeats.
A-phased repeat (turquoise): three or more tracts of $A_{3\text{-}7}$, $T_{3\text{-}7}$, AAATTT, AAATTTT and AAAATTT in any combination located on the plus or minus strand. The centre of the tracts are separated by 11–12-bp (Cer et al., 2011; Cer et al., 2013).
QC (olive): G-quadruplex-forming repeat encompassing four repeat units, each containing the same number of 'Gs' (on the plus or minus strand). Their number can vary from 3 to 7 and the repeat units can be separated by 1–7-bp (Cer et al., 2011).

Table S5: Number of non-B DNA-forming sequence motifs located within the breakpoint-flanking regions of the 15 atypical *NF1* deletions with simple breakpoints as compared with the number of such motifs identified within the control sequence dataset.

Number of sequences exhibiting	*NF1* deletion breakpoint-flanking sequences (N=30)[a]	Control sequences (N=200)[b]	P-value[c]
at least one non-B DNA-forming motif	16 (53%)	137 (68.5%)	0.14
specific subtypes of non-B DNA-forming motifs: [d]			
inverted repeat	1 (3%)	10 (5%)	0.99
cruciform	10 (33%)	84 (42%)	0.43
short tandem repeat	8 (27%)	43 (21.5%)	0.49
polypurine tract	5 (17%)	25 (12.5%)	0.56
polypyrimidine tract	2 (7%)	22 (11%)	0.75
Z-DNA	2 (7%)	7 (3.5%)	0.33

a: Thirty *NF1* deletion breakpoint-flanking sequence fragments were analysed, each encompassing 300-bp. In parentheses are the proportions of the 30 deletion breakpoint-spanning sequences that exhibited non-B DNA-forming sequence motifs.
b: The control sequences comprised 200 fragments of 300-bp each. The control sequences do not flank any known atypical *NF1* deletion breakpoints. The corresponding sequences are located within 17q11.2 telomeric to *SUZ12P* (genomic position: 29,118,000-29,148,000; hg19) and between *RAB11FIP4* and *COPRS* (30,020,000-30,050,000; hg19). In total, the control dataset encompassed 60-kb of genomic DNA. In parentheses are indicated the proportions of the 200 sequence fragments that exhibited specific non-B DNA-forming sequence motifs.
c: The two-tailed Fisher's Exact test was applied to calculate the statistical significance of the differences in the number of non-B DNA motifs observed in the breakpoint-flanking sequences of deletion breakpoints and in the control dataset.
d: Some sequences from the investigated datasets fulfill the criteria for more than one non B-DNA motif subtype, i.e. 'TTAATTAATTAA' represents a short tandem repeat (2−6-bp sequence repeated several times) as well as a cruciform repeat which is a subtype of an inverted repeat of ≥6-bp separated by 0–4-bp. Therefore, the number of sequences exhibiting non-B DNA subtypes exceeds the number of sequences with at least one non-B DNA-forming motif.

Table S6: Numbers of direct and inverted repeats identified within 150-bp flanking the breakpoints of the 15 atypical *NF1* deletions with simple breakpoints on both sides as compared with the numbers of such repeats identified within a control dataset of sequences not harbouring *NF1* deletion breakpoints. MEME suite (http://meme.nbcr.net/meme/) was used to analyse repeats ≥6-bp up to 150-bp. The number of base-pairs between the repeats was not restricted to a specific length.

Number of repeats	Number of deletion breakpoint-flanking sequences exhibiting the indicated number of repeats[a]	Number of control sequences that exhibit the indicated number of repeats[b]	P-value[c]
1–6 DR	23 (77%)	161 (80.5%)	
0 DR	7	39	0.627
1 DR	12	51	0.123
2 DR	3	50	0.101
3 DR	4	37	0.615
4 DR	2	16	0.999
5 DR	1	7	0.999
6 DR	2	0	
1–6 IR	23 (77%)	161 (80.5%)	
0 IR	8	39	0.342
1 IR	14	68	0.220
2 IR	6	53	0.510
3 IR	3	30	0.586
4 IR	0	8	
5 IR	0	1	
6 IR	0	1	

DR: direct repeat(s); IR: inverted repeat(s).

a: In total, we investigated 30 breakpoint-flanking sequences of 300-bp each. These 300-bp regions comprise 150-bp centromeric and 150-bp telomeric to each *NF1* deletion breakpoint.

b: The control sequences were located within 17q11.2 telomeric to *SUZ12P* (genomic position: 29,118,000-29,148,000; hg19) and between *RAB11FIP4* and *COPRS* (30,020,000-30,050,000; hg19). In total, the control dataset comprised 60-kb of genomic DNA including 200 fragments of 300-bp each.

c: The two-tailed Fisher's Exact test was applied to calculate the statistical significance of the differences in the number of direct and inverted repeats observed in the breakpoint-flanking sequences of the deletion breakpoints and in the control dataset.

Table S7: Direct and inverted repeats (>150-bp, sequence homology of ≥87%) located within 2-kb regions flanking the 15 atypical *NF1* deletion breakpoints on both sides. The repeats were identified by means of BLASTN self-alignments of the 4-kb regions (http://blast.ncbi.nlm.nih.gov/Blast.cgi) (bl2seq[a]).

Patient	Breakpoint location	Length of repeat (retrotransposon)	Genomic position of the region of homology between the repeats	Sequence homology between the repeats	Distance between the repeats	Orientation of the repeats
D05.2678	28,142,439	292-bp (*Alu*Sx1[b])<br>288-bp (*Alu*Y)	28,140,555-28,140,846<br>28,141,173-28,141,460	88%	327-bp	direct
D0801587	29,729,878	316-bp (*Alu*Y)<br>311-bp (*Alu*Sc8[b])	29,729,627-29,729,942<br>29,730,329-29,730,639	88%	388-bp	direct
		285-bp (*Alu*Y)<br>285-bp (*Alu*Y)	29,728,004-29,728,288<br>29,729,659-29,729,943	88%	1,372-bp	inverted
D0801587	27,726,516	279-bp (*Alu*Sp)<br>281-bp (*Alu*Sq2)	27,725,223-27,725,501<br>27,725,547-27,725,827	89%	47-bp	direct
R48018	29,084,006	245-bp (*Alu*Sg)<br>244-bp (*Alu*Sg[b])	29,082,933-29,083,177<br>29,084,829-29,085,072	87%	1,653-bp	direct
R84329	29,074,557	292-bp (*Alu*Yf4)<br>285-bp (*Alu*Y)	29,073,481-29,073,772<br>29,074,182-29,074,466	89%	411-bp	direct

a: The sequence alignments were performed using default settings for the algorithm parameters: Expect threshold: 10, word size: 28 and match/mismatch scores: 1, -2. The following parameters were however not run under default settings but instead adjusted to the requirements of our analysis: the parameter 'number of maximum target sequences' was increased from 100 (default) to 20,000 so that all sequence alignments were displayed. The parameter 'maximum matches in a query' was changed from 0 (default) to 100 in order to identify all possible matches to the query sequence. The parameter 'gap costs' was changed from the default setting 'linear' to 'existence: 5 and extension: 2'. These settings imply that the cost to open a gap scores -5 whereas the cost to extend the gap scores -2. By adopting these settings, we reduced the number of gaps that would extend the length of the alignment at the expense of the sequence identity.
b: Full-length retrotransposon.

Table S8: Direct and inverted repeats (>150-bp, sequence homology of ≥87%) within the control dataset of 120-kb[a]. The repeats were identified by means of BLASTN self-alignments of 4-kb regions (http://blast.ncbi.nlm.nih.gov/Blast.cgi) (bl2seq[a]).

**Control regions[b]**	**Position of the hypothetical breakpoint**	**Length of repeat (retrotransposon)**	**Genomic position of repeat**	**Homology between repeats**	**Distance between repeats**	**Orientation of the repeat**
K14	30,046,000	276-bp (*Alu*Y)<br>276-bp (*Alu*Y)	30,044,724-30,044,999<br>30,045,987-30,046,262	87%	989-bp	inverted
K16	29,152,000	284-bp (*Alu*Sg)<br>282-bp (*Alu*Sx1)	29,150,105-29,150,388<br>29,152,511-29,152,792	87%	2,124-bp	direct
K21	29,172,000	298-bp (*Alu*Sx)<br>293-bp (*Alu*Sc)	29,171,140-29,171,437<br>29,173,415-29,173,707	87%	1,979-bp	direct
K23	29,180,000	194-bp (*Alu*Sx)<br>191-bp (*Alu*Sx1)	29,178,002-29,178,195<br>29,180,006-29,180,196	88%	1,812-bp	direct
K26	29,192,000	305-bp (*Alu*Y[c])<br>305-bp (*Alu*Y[c])	29,190,379-29,190,683<br>29,192,185-29,192,489	89%	1,503-bp	direct
K28	29,200,000	261-bp (*Alu*Sx1)<br>257-bp (*Alu*Sc5)	29,198,407-29,198,667<br>29,201,279-29,201,535	87%	2,619-bp	inverted
		256-bp (*Alu*Sx1)<br>252-bp (*Alu*Sg)	29,198,401-29,198,656<br>29,201,684-29,201,935	87%	3,028-bp	direct
K30	29,208,000	295-bp (*Alu*Sp)<br>292-bp (*Alu*Sp[c])	29,207,345-29,207,639<br>29,208,996-29,209,287	88%	1,358-bp	direct

a: The sequence alignments were performed using default settings for the algorithm parameters: Expect threshold: 10, word size: 28 and match/mismatch scores: 1, -2. The following parameters were however not run under default settings but instead adjusted to the requirements of the analysis; the parameter 'number of maximum target sequences' was increased from 100 (default) to 20,000 so that all sequence alignments were displayed. The parameter 'maximum matches in a query' was changed from 0 (default) to 100 in order to identify all possible matches to the query sequence. The parameter 'gap costs' was changed from the default setting 'linear' to 'existence: 5 and extension: 2'. These settings imply that the cost to open a gap scores -5 whereas the cost to extend the gap scores -2. By adopting these settings, we reduced the number of gaps that would extend the length of the alignment at the expense of the sequence identity.
b: The control dataset comprises two genomic regions: one is located telomeric to *SUZ12P* (genomic position: 29,118,000-29,210,000; 92-kb), the other between *RAB11FIP4* and *COPRS* (genomic position: 30,020,000-30,048,000; 28-kb). The total 120-kb of genomic DNA were subdivided into 30 regions of 4-kb each and hypothetical breakpoints were assigned locations between nucleotides at positions 2,000 and 2,001 of each of these 4-kb fragments. Only seven of these 30 control regions (K1-K30) contained direct or inverted repeats as indicated in the first column.
c: Full-length retrotransposon.

Table S9: Number of direct and inverted sequence repeats >150-bp within the breakpoint-flanking regions of the 15 atypical *NF1* deletions with simple breakpoints as compared with the number of such repeats in a control dataset of sequences.

Sequence feature investigated	Number of *NF1* deletion breakpoint-flanking sequences with repeats[a]	Number of control sequences with repeats[a]	P-value[b]
Direct repeat (>150-bp)	5/30 (17%)	6/30 (20%)	0.99
Inverted repeat (>150-bp)	1/30 (3%)	2/30 (7%)	0.99

a: The number of direct and inverted repeats >150-bp exhibiting ≥87% sequence homology was determined by BLASTN self-alignments of 2-kb regions flanking the deletion breakpoint regions on both sides. The number of such repeats was also determined in a control dataset of sequences derived from two genomic regions: one is located telomeric to *SUZ12P* (genomic position: 29,118,000-29,210,000; 92-kb), the other between *RAB11FIP4* and *COPRS* (genomic position: 30,020,000-30,048,000; 28-kb). In total, these two regions comprise 120-kb of genomic DNA which were subdivided into 30 fragments of 4-kb each. Hypothetical breakpoints were assigned locations between nucleotides at positions 2,000 and 2,001 of each of these 4-kb fragments.

b: The two-tailed Fisher's Exact test was applied to assess the statistical significance of the differences in the number of repeats observed.

Table S10: Identification of inverted repeats ≥1-kb located within 40-kb flanking the atypical *NF1* deletion breakpoints. The 40-kb sequence fragments analysed spanned 20-kb centromeric and 20-kb telomeric to each deletion breakpoint. To identify repeats ≥1-kb, we performed BLASTN self-alignments of the 40-kb fragments under the search conditions "highly similar sequences". Each of these 40-kb sequence fragments was downloaded from the reference sequence of the human genome (hg19). Inverted repeats ≥1-kb were only identified in breakpoint-flanking regions of patients 619 and 659 as indicated. The respective repeats exhibited 99% sequence homology.

**Patient**	**Proximal deletion breakpoint**		
	Position of breakpoint	Length of the inverted repeats	Genomic position of the inverted repeats (hg19)
619	28,946,218	5735-bp<br>5762-bp	28,943,147-28,948,899<br>28,952,608-28,958,368
659	28,948,946[a]	5735-bp<br>5762-bp	28,943,147-28,948,899<br>28,952,608-28,958,368

a: Breakpoint located between inverted repeats.

Table S11: Comparison of the number of retrotransposons located at or directly adjacent to the breakpoints of 15 atypical *NF1* deletions with the number of such elements identified in the control dataset.

	Number of retrotransposons in the		P-value[c]
	NF1 deletion breakpoint-flanking sequences[a]	Control dataset[b]	
**All retrotransposons[d]**	22	136	0.67
**LTR elements**	1	5	0.57
**Non-LTR retrotransposons:**			
LINE elements	3	47	0.15
SINE elements	18	84	0.08
Alu[e]	17	75	0.07
FLAM_C[f]	1	3	0.43
FRAM[f]	0	1	
MIR	0	5	

a: The *NF1* deletion breakpoint dataset included 30 sequence fragments of 300-bp flanking the breakpoints of the 15 atypical *NF1* deletions. The breakpoints were located between nucleotides 150 and 151 of each of these 300-bp fragments. In our analysis, we considered those retrotransposons that overlapped the breakpoints or which were located immediately adjacent to the breakpoints.
b: The control dataset included 200 sequence fragments of 300-bp that are located in 17q11.2 within genomic regions not harbouring known atypical *NF1* deletion breakpoints. The control sequence dataset comprised two 30-kb regions, one located telomeric to *SUZ12P* (genomic position: 29,118,000-29,148,000; hg19) and the other located between *RAB11FIP4* and *COPRS* (30,020,000-30,050,000; hg19). The 60-kb of genomic DNA sequences were subdivided into two hundred 300-bp regions and hypothetical breakpoints were assigned locations between nucleotides 150 and 151 of each of these 300-bp fragments.
c: The two-tailed Fisher's Exact test was applied to assess the statistical significance of the differences between the number of elements observed in the two datasets.
d: LTR and non-LTR retrotransposons (LINE and SINE)
e: Full-length and partial *Alu* elements
f: FLAM_C: free left *Alu* monomer; FRAM: free right *Alu* monomer

Table S12: The SVA elements inserted at the deletion breakpoints in patient DA-77 and ASB4-55 were PCR amplified with primers located within non-deleted regions flanking the SVA elements on both sides. For this purpose, we used long PCR primers with a high melting temperature and high GC-content as summarized below.

**Patient**	**Primer**				
	**designation**	**sequence (5'→3')**	**length (bp)**	**melting temperature (°C)**[a]	**GC-content (%)**[a]
ASB4-55	as117for	CCCAGAATTCCATAGTTACCAGATTCA	27	68.2	41
	as146Brev	TGATCTACTGACAGGTTACCCTTGGA	26	68.2	46
DA-77	GSP1_for	TTTTAGGCAGCATGGGGTATGTTCTG	26	70.1	46
	GSP1_rev	TCTGATCATCCATACGTGACACACTGA	27	69.7	44

a: melting temperature and GC-content were determined with the RaW-program (http://www.mrc-holland.com/).

The Expand Long Template PCR system (Roche, Mannheim, Germany) was used to perform the PCR with the addition of 10% DMSO and an annealing temperature of 58°C. The genomic DNA used as PCR template was diluted with water to 40 ng/µl and added to the PCR to a final amount of 400 ng. The initial denaturation of the genomic DNA was performed for 10 minutes.

Table S13: Somatic mosaicism of cells harbouring the atypical *NF1* deletion and normal cells was detected in 10 of the 17 patients analysed (indicated in bold and marked in grey). Eight of these patients were investigated by FISH and six exhibited somatic mosaicism. The analysis of microsatellite markers and markers detecting a polymorphic insertion/deletion (indel) located within the deletion region revealed somatic mosaicism in four additional patients who were not investigated by FISH. In these four patients, mosaicism was detected by marker heterozygosity.

**Patient ID**	**Mosaicism investigated by**		
	FISH % cells with the deletion	microsatellite marker analysis	indel marker rs17884042[a]
R48018	100%	ni	ni
619	100%	ni	ni
**R84329**	98.5%	nd	nd
**Grandmother of DA-77[b]**	75%	nd	nd
**659**	96%[c]	ni	nd
**Ak-47055**	80%	+ [d]	ni
**100206**	71%	+ [e]	nd
**ASB4-55**	93%	+ [f]	nd
**D0801587**	nd	+ [e]	nd
**08D2261**	nd	ni	+
**61541**	nd	ni	+
**D06.1047**	nd	ni	+
2535	nd	ni	nd
1106	nd	ni	ni
D1008345	nd	ni	ni
D05.2678	nd	ni	ni
70969	nd	ni	ni

ni: not informative, heterozygosity was not detected.
nd: not determined
a: Somatic mosaicism was investigated by PCR and sequence analysis of the insertion/deletion (indel) polymorphism rs17884042 (−/ACAAAAATATTTGA, 29,533,692-29,533,706) located within the *NF1* gene. PCR was performed to amplify the allele lacking the 15-bp by means of primers InDel1f (5'-CACCCAGCAATACGAATG-3', 29,533,366-29,533,383) and InDel2r (5'-AAACGTGAGAGGCTAATCAAAAGT-3', 29,533,701-29,533,722). The allele harbouring the 15-bp insertion was amplified with primer InDel1f and primer InDel3r (5'-CCCAAAACGTGAGAGGCTAATCAAAATA-3', 29,533,699-29,533,726).
b: Peripheral blood of the grandmother of patient DA-77 was investigated by FISH analysis.
c: In patient 659, FISH detected the deletion in 96% of blood cells and in 52% of buccal epithelial cells.
d: Heterozygosity was detected for markers NF1.PCR3, D17S2237, IVs27AC28.4, D17S1166 and D17S1800.
e: Heterozygosity was detected for marker 3'NF1 (29,919,355-29,919,599) amplified with primers 5'-CTTCCATGGCTGCTAACATC-3' and 5'-CCCTGTGGTGTAGTTCAACA-3'.
f: Heterozygosity was detected for markers D17S1849, IVs27TG24.8, IVs27AC28.4, D17S1166 and D17S1800.
All genomic positions are according to hg19.

Table S14: Primers used for the PCR experiments performed to assess the putative SVA element insertion/deletion polymorphism in *SUZ12P* intron 8 in 50 Africans and 50 white Europeans. The genomic DNAs used for these experiments were derived from the human variation panels MGP00008 and MGP00013 (Coriell Cell Repositories, Camden, NJ, USA) and white Europeans from Germany.

Primer designation	Sequence (5'→3')	Genomic position (hg19)
AD91for	CTGGTGACAGCGAGACTCTG	29,099,439-29,099,458 (chromosome 17)
SVA4rev	GAAAATGTATTTAAATGTCTGCACCAA	101,596,855-101,596,881 (chromosome 10)
ASB4SVA1for	CCCCAGAATTCCATAGTTACCAGA	29,102,523-29,102,546 (chromosome 17)
ASB4SVA1rev	TGCTCGTTAAGAATCATCACCAAT	123,169,120-123,169,143 (chromosome 6)

Table S15: Primers used for the PCR experiments performed to investigate whether chromosomes 17 harbouring the SVA insertions within *SUZ12P* intron 8 but not the large *NF1* deletions would be detectable in the grandmother of patient DA-77 and in patient ASB4-55. Both patients exhibit somatic mosaicism with normal cells and those with the atypical *NF1* deletions.

Patient analysed	Primer designation	Primer sequence (5'→3')	Chromosomal position (hg19) of the primer
Grandmother of patient DA-77	SVA4rev	GAAAATGTATTTAAATGTCTGCACCAA	101,596,855-101,596,881 (chromosome 10)
	SVA11for	ACATTTTCTGGTATAACCACCATACA	29,099,681-29,099,706 (chromosome 17)
	SVA11rev	TTCAAGCAAAAATAATCAATGC	29,100,193-29,100,214 (chromosome 17)
	SVA12for	GCGTTTGTCTTATCACTCAGGA	30,101,302-30,101,323 (chromosome 17)
	SVA12rev	GGGAAACTTGGGAAACAGAA	30,101,871-30,101,890 (chromosome 17)
	SVA13for	TGGAATTATTGGTGCAGACATT	101,596,847-101,596,868 (chromosome 10)
	SVA13rev	TGCCCATTCTTCTATTGGGCAAC	29,101,596-29,101,618 (chromosome 17)
	SVA14for	ACACATGCTGGAATTATTGGTGCAGA	101,596,839-101,596,864 (chromosome 10)
	GSP1for	TTTTAGGCAGCATGGGGTATGTTCTG	29,099,021-29,099,046 (chromosome 17)
	AD75for	TCCAGACCCATCTGAACATCT	30,099,005-30,099,025 (chromosome 17)
	AD21rev	GTGCTCTGCTTGGCCTATTC	30,103,669-30,103,688 (chromosome 17)
ASB4-55	ASB4SVA1rev	TGCTCGTTAAGAATCATCACCAAT	123,169,120-123,169,143 (chromosome 6)
	ASB4SVA2for	AAAGACCCTGCTGACAAAACA	29,969,639-29,969,659 (chromosome 17)
	ASB4SVA2rev	TGAGATGGTGCCATTATACTCC	29,970,067-29,970,088 (chromosome 17)
	ASB4SVA3for	GTTTCCCTAAGACAAGCATTGTAAC	29,102,642-29,102,666 (chromosome 17)
	ASB4SVA3rev	CATTTGGGGAAGAAAACATCAA	29,103,475-29,103,496 (chromosome 17)
	ASB4SVA4for	TGAGATTAGGGATTGGTGATGA	123,169,109-123,169,130 (chromosome 6)
	ASB4SVA4rev	TTAGCATTTGGGGAAGAAAACATCAA	29,103,475-29,103,500 (chromosome 17)
	ASB4SVA5for	CTTTCTACACAGACACGGCAACCATC	123,169,331-123,169,356 (chromosome 6)

The Expand Long Template PCR system (Roche) was used to perform the PCRs with the addition of 10% DMSO. The genomic DNA used as PCR template was diluted at 40ng/µl with water and added to the PCR to a final amount of 400ng. The initial denaturation of the genomic DNA was performed for 10 minutes. Different elongation times were tested in order to amplify not only 5' truncated (and hence shorter) SVA element insertions but also full-length SVA elements putatively inserted into *SUZ12P* intron 8 or within the intergenic region between *RAB11FIP4* and *COPRS*.

Table S16: Preponderance of the telomeric atypical *NF1* deletion breakpoints in the genomic region extending from position 30,218,204-30,250,762 (region 1).

Genomic regions analysed[a] No.	Location (extent)	Observed number of breakpoints located within the region	Expected number of breakpoints within the region[b]	P-value[c]
1	30,218,204-30,250,762 (32,559-bp)	5	1	<0.0001
2	29,729,878-30,218,203 (488,326-bp) and 30,250,763-30,345,260 (94,498-bp)	10	14	

a: The telomeric breakpoints of all 15 atypical *NF1* deletions considered are located within a region spanning 615,383-bp. The most centromeric breakpoint is located at position 29,729,878 (patient D0801587) and the most telomeric breakpoint is located at position 30,345,260 (patient 659). Patients 619 and D05.2678 were not included in the analysis because their telomeric deletion breakpoints were not located within the region between NF1-REPa and NF1-REPc but instead were located 1.53-Mb and 3.69-Mb telomeric to NF1-REPc, respectively. The 615,383-bp region harbouring all 15 telomeric breakpoints was subdivided into two regions: region 1 (32,559-bp) and region 2 (582,824-bp).
b: The expected number of breakpoints within region 1 was determined as follows: A total of 15 breakpoints were observed to be located within 615,383-bp. The proportion of the entire 615,383-bp corresponding to region 1 is 0.05 and the proportion corresponding to region 2 is 0.95. Under the assumption of an equal number of breakpoints in both regions 1 and 2, the expected number of breakpoints in region 1 is N=1 (0.05 x 15) and in region 2: N=14 (0.95 x 15).
c: The chi-squared test (one degree of freedom) was used to calculate the significance of the difference between the observed versus the expected number of breakpoints.

Table S17: Preponderance of the centromeric atypical *NF1* deletion breakpoints in *SUZ12P* (region 1, extending from position 29,065,415 to position 29,104,496).

No.	Genomic regions analysed[a] Location (extent)	Observed number of breakpoints located within the region	Expected number of breakpoints within the region[b]	P-value[c]
1	29,065,415-29,104,496 (39,082-bp)	11	2	<0.0001
2	28,946,218-29,065,414 (119,197-bp) and 29,104,497-29,264,225 (159,729-bp)	4	13	

a: The centromeric breakpoints of all 15 atypical *NF1*deletions considered are located in a region spanning 318,008-bp. The most centromeric breakpoint is located at position 28,946,218 (patient 619) and the most telomeric breakpoint is located at position 29,264,225 (patient D06.1047). Patients D05.2678 and D0801587 were not included in the analysis because their centromeric deletion breakpoints were not located within the region between NF1-REPa and NF1-REPc but instead were located 785-kb and 1.2-Mb centromeric to NF1-REPa, respectively. The 318,008-bp region harbouring all 15 centromeric breakpoints was subdivided into two regions: region 1 (39,082-bp) and region 2 (278,926-bp).
b: The expected number of breakpoints within region 1 was determined as follows: A total of 15 breakpoints were observed to be located within 318,008-bp. The proportion of the entire 318,008-bp corresponding to region 1 is 0.12 and the proportion corresponding to region 2 is 0.88. Under the assumption of an equal number of breakpoints in both regions 1 and 2, the expected number of breakpoints in region 1 is N=2 (0.12 x 15) and in region 2, the expected number of breakpoints is N=13 (0.88 x 15).
c: The chi-squared test (one degree of freedom) was used to calculate the significance of the difference between the observed versus the expected numbers of breakpoints.

Table S18: Locations of the breakpoints of the 11 atypical *NF1* deletions within *SUZ12P* and in relation to the location of the breakpoint regions of type-2 *NF1* deletions according to Vogt et al., (2012).

**Patients with type-2 *NF1* deletions (genomic locations of the type-2 breakpoint regions)**[a]		**Patients with atypical deletions (position of the breakpoints)**		**Distance between the atypical *NF1* deletion breakpoint and the breakpoint region of the type-2 *NF1* deletion given in in the same line**
811-M	(29,069,025-29,069,071)	100206	(29,065,415)	3,611-bp
KCD	(29,071,185-29,071,293)			
R605111	(29,073,072-29,073,315)	R84329	(29,074,557)	1,243-bp
697	(29,076,187-29,076,255)			
R323001	(29,077,548-29,077,693)			
2442	(29,078,929-29,078,979)			
736	(29,080,465-29,080,552)	Ak-45077	(29,082,023)	1,472-bp
736	(29,080,465-29,080,552)	61541	(29,082,032)	1,481-bp
1630	(29,084,697-29,084,825)	R48018	(29,084,006)	692-bp
R45407	(29,084,697-29,084,825)			
R164101	(29,085,517-29,085,686)			
2358	(29,085,789-29,086,051)			
R368101	(29,085,789-29,086,051)			
585	(29,085,789-29,086,051)			
488	(29,087,423-29,087,479)			
R636011	(29,087,423-29,087,479)			
1502	(29,087,632-29,087,779)			
1956	(29,088,369-29,088,400)			
R690001	(29,090,564-29,090,621)			
D0703976#4	(29,091,112-29,091,232)			
D0900751#7	(29,091,112-29,091,232)			
IL39	(29,091,380-29,091,426)			
1104	(29,091,476-29,091,551)			
R39407	(29,091,628-29,091,737)			
R55816	(29,092,785-29,092,963)	70969	(29,092,903)	overlapping[b]
R55816	(29,092,785-29,092,963)	D1008345	(29,094,424)	1,462-bp
2429	(29,098,821-29,099,182)			
R286011	(29,099,490-29,099,591)			
R53327	(29,099,719-29,099,793)	DA-77	(29,100,005)	213-bp
R716111	(29,100,541-29,100,765)			
R320021	(29,100,541-29,100,765)			
WB	(29,101,104-29,101,356)			
UC172	(29,101,104-29,101,356)			
R24026	(29,101,104-29,101,356)			
R20807	(29,101,104-29,101,356)	2535	(29,101,686)	331-bp
R93418	(29,102,982-29,103,195)	08D2261	(29,102,848)	135-bp
R93418	(29,102,982-29,103,195)	ASB4-55	(29,103,071)	overlapping[b]
938	(29,103,520-29,103,590)			
R37716	(29,104,990-29,105,048)			
R928201	(29,105,197-29,105,339)			
R491021	(29,105,417-29,105,549)			
R465111	(29,105,417-29,105,549)			

a: Type-2 *NF1* deletions are mediated by nonallelic homologous recombination between *SUZ12* and *SUZ12P*.The breakpoints of the respective deletions cannot be assigned to single base-pair resolution since the breakpoints occurred in short regions of 100% sequence identity (so called breakpoint regions).

b: The breakpoint of the atypical *NF1* deletion is located within the breakpoint region of the type-2 *NF1* deletion.

Table S19: Number of non-B DNA-forming sequence motifs located within the breakpoint-flanking regions of the 11 atypical *NF1* deletions with centromeric breakpoints located within *SUZ12P* as compared with the number of such motifs identified within the control sequence dataset.

**Number of sequences exhibiting**	***NF1* deletion breakpoint-flanking sequences (N=11)[a]**	**Control sequences (N=200)[b]**	**P-value[c]**
at least one non-B DNA-forming motif	7 (64%)	137 (68.5%)	0.75
specific subtypes of non-B DNA-forming motifs: [d]			
inverted repeat	0	10 (5%)	
cruciform	6 (55%)	84 (42%)	0.53
short tandem repeat	5 (45%)	43 (21.5%)	0.13
polypurine tract	1 (9%)	25 (12.5%)	0.99
polypyrimidine tract	1 (9%)	22 (11%)	0.99
Z-DNA	2 (18%)	7 (3.5%)	0.07

a: Eleven *NF1* deletion breakpoint-flanking sequence fragments were analysed, each encompassing 300-bp. In parentheses are the proportions of the 11 deletion breakpoint-spanning sequences that exhibited non-B DNA-forming sequence motifs.
b: The control sequences comprised 200 fragments of 300-bp each. The control sequences do not flank any known atypical *NF1* deletion breakpoints. The corresponding sequences are located within 17q11.2 telomeric to *SUZ12P* (genomic position: 29,118,000-29,148,000; hg19) and between *RAB11FIP4* and *COPRS* (30,020,000-30,050,000; hg19). In total, the control dataset encompassed 60-kb of genomic DNA. In parentheses are indicated the proportions of the 200 sequence fragments that exhibited specific non-B DNA-forming sequence motifs.
c: The two-tailed Fisher's Exact test was applied to calculate the statistical significance of the differences in the number of non-B DNA motifs observed in the breakpoint-flanking sequences of deletion breakpoints and in the control dataset.
d: Some sequences from the investigated datasets fulfill the criteria for more than one non B-DNA motif subtype, i.e. 'TTAATTAATTAA' represents a short tandem repeat (2−6-bp sequence repeated several times) as well as a cruciform repeat which is a subtype of an inverted repeat of ≥6-bp separated by 0–4-bp. Therefore, the number of sequences exhibiting non-B DNA subtypes exceeds the number of sequences with at least one non-B DNA-forming motif.

Table S20: Numbers of direct and inverted repeats identified within 150-bp flanking the breakpoints of the 11 atypical *NF1* deletions with centromeric breakpoints located in *SUZ12P* as compared with the numbers of such repeats identified within a control dataset of sequences not harbouring *NF1* deletion breakpoints. MEME suite (http://meme.nbcr.net/meme/) was used to analyse repeats ≥ 6-bp up to 150-bp. The number of base-pairs between the repeats was not restricted to a specific length.

Number of repeats	Number of deletion breakpoint-flanking sequences exhibiting the indicated number of repeats[a]	Number of control sequences that exhibit the indicated number of repeats[b]	P-value[c]
1–6 DR	8 (73%)	161 (80.5%)	
0 DR	3	39	0.461
1 DR	4	51	0.482
2 DR	0	50	
3 DR	3	37	0.440
4 DR	0	16	
5 DR	0	7	
6 DR	1	0	
1–6 IR	9 (82%)	161 (80.5%)	
0 IR	2	39	0.999
1 IR	4	68	0.999
2 IR	4	53	0.493
3 IR	1	30	0.999
4 IR	0	8	
5 IR	0	1	
6 IR	0	1	

DR: direct repeat(s); IR: inverted repeat(s).
a: In total, we investigated 11 centromeric breakpoint-flanking sequences of 300-bp each. These 300-bp regions comprise 150-bp centromeric and 150-bp telomeric to each *NF1* deletion breakpoint.
b: The control sequences were located within 17q11.2 telomeric to *SUZ12P* (genomic position: 29,118,000-29,148,000; hg19) and between *RAB11FIP4* and *COPRS* (30,020,000-30,050,000; hg19). In total, the control dataset comprised 60-kb of genomic DNA including 200 fragments of 300-bp each.
c: The two-tailed Fisher's Exact test was applied to calculate the statistical significance of the differences in the number of direct and inverted repeats observed in the breakpoint-flanking sequences of the deletion breakpoints and in the control dataset.

Table S21: Number of direct sequence repeats >150-bp within the breakpoint-flanking regions of the 11 atypical *NF1* deletions with centromeric breakpoints located in *SUZ12P* as compared with the number of such repeats in a control dataset of sequences.

**Sequence feature investigated**	**Number of *NF1* deletion breakpoint-flanking sequences with repeats**[a]	**Number of control sequences with repeats**[a]	**P-value**[b]
Direct repeat (>150-bp)	2/11 (18%)	6/30 (20%)	0.99

a: The number of direct repeats >150-bp exhibiting ≥87% sequence homology was determined by BLASTN self-alignments of 2-kb regions flanking the deletion breakpoint regions on both sides. The number of such repeats was also determined in a control dataset of sequences derived from two genomic regions: one is located telomeric to *SUZ12P* (genomic position: 29,118,000-29,210,000; 92-kb), the other between *RAB11FIP4* and *COPRS* (genomic position: 30,020,000-30,048,000; 28-kb). In total, these two regions comprise 120-kb of genomic DNA which were subdivided into 30 fragments of 4-kb each. Hypothetical breakpoints were assigned locations between nucleotides at positions 2,000 and 2,001 of each of these 4-kb fragments.

b: The two-tailed Fisher's Exact test was applied to assess the statistical significance of the differences in the number of repeats observed.

Table S22: SVA insertion-associated deletions in the human genome as compared with the chimpanzee genome according to (Lee et al., 2012).

**Genomic position of the inserted SVA element (hg19)**		**Deletion size (bp)**
Chr 1	160,905,975–160,906,748	1,929
Chr 3	149,097,867–149,099,196	2,581
Chr 3	61,656,812–61,658,447	19
Chr 7	75,581,297–75,582,629	653
Chr 8	122,668,707–122,669,486	1,191
Chr 8	57,983,370–57,984,835	14
Chr 8	145,092,008–145,092,734	4,859
Chr 8	34,951,832–34,952,650	2,791
Chr 10	101,851,975–101,854,321	5,997
Chr 12	31,333,177–31,333,788	347
Chr 14	84,565,214–84,566,566	8,741
Chr 16	67,746,159–67,746,860	30
Chr 22	35,021,272–35,022,965	1,633

Table S23: The 17 unrelated NF1 patients harbouring atypical *NF1* deletions investigated in the present study were initially identified in different centres as listed below. Blood-derived genomic DNA was used to analyse the deletion breakpoints. For some of the patients (marked in bold), EBV-transformed lymphoblastoid cell lines were also available for analysis.

Patient ID	Centres
D06.1047<br>08D2261<br>D05.2678	Department of Clinical Genetics, Erasmus Medical Centre, Rotterdam, The Netherlands
70969<br>100206<br>61541<br>R84329<br>R48018	Medical Genomics Laboratory, Department of Genetics, University of Alabama at Birmingham, USA
**DA-77**	Division of Clinical Genetics, Medical University Innsbruck, Austria
**ASB4-55**	Molecular Diagnostics Unit, Hereditary Cancer Program, Catalan Institute of Oncology (ICO-IDIBELL), L'Hospitalet de Llobregat, Barcelona, Spain; Department of Human Genetics, Catholic University Leuven, Belgium
**1106**<br>D1008345<br>D0801587	Centre for Medical Genetics, Ghent University Hospital, Belgium
2535	Institute of Medical Genetics, Cardiff University School of Medicine, UK
Ak-47055	Department of Pediatrics, Duisburg General Hospital, Duisburg, Germany
**659**<br>**619**	Department of Neurology, University Medical Centre Hamburg Eppendorf, Germany

Table S24: Extent of the 17 atypical *NF1* deletions according to MLPA analysis using the P122-C1 kit (MRC Holland, The Netherlands). The genomic positions of the respective probes and their complete designation are given in Table S25.

**MLPA-probe**	**Patients**																
	D06.1047	70969	DA-77	ASB4-55	08D2261	100206	61541	1106	D1008345	2535	R84329	R48018	Ak-47055	659	D0801587	619	D05.2678
TRAF4	+	+	+	+	+	+	+	+	+	+	+	+	+	+	+	+	+
TRAF4	+	+	+	+	+	+	+	+	+	+	+	+	+	+	+	+	+
SSH2	+	+	+	+	+	+	+	+	+	+	+	+	+	+	−	+	+
SSH2	+	+	+	+	+	+	+	+	+	+	+	+	+	+	−	+	+
BLMH	+	+	+	+	+	+	+	+	+	+	+	+	+	+	−	+	−
BLMH	+	+	+	+	+	+	+	+	+	+	+	+	+	+	−	+	−
CPD	+	+	+	+	+	+	+	+	+	+	+	+	+	+	−	+	−
CPD	+	+	+	+	+	+	+	+	+	+	+	+	+	+	−	+	−
SUZ12P	+	+	+	+	+	+	+	−	+	+	+	+	+	−	−	−	−
SUZ12P	+	+	+	+	+	+	+	−	+	+	+	+	+	−	−	−	−
SUZ12P	+	+	+	+	+	−	−	−	+	+	−	−	−	−	−	−	−
CRLF3	+	−	−	−	−	−	−	−	−	−	−	−	−	−	−	−	−
ATAD5	+	−	−	−	−	−	−	−	−	−	−	−	−	−	−	−	−
CENTA2	+	−	−	−	−	−	−	−	−	−	−	−	−	−	−	−	−
RNF135	−	−	−	−	−	−	−	−	−	−	−	−	−	−	−	−	−
NF1 Ex. 1	−	−	−	−	−	−	−	−	−	−	−	−	−	−	−	−	−
NF1 Ex. 12B	−	−	−	−	−	−	−	−	−	−	−	−	−	−	−	−	−
NF1 Ex. 23	−	−	−	−	−	−	−	−	−	−	−	−	−	−	−	−	−
NF1 Ex. 40	−	−	−	−	−	−	−	−	−	−	−	−	−	−	−	−	−
NF1 Ex. 48	−	−	−	−	−	−	−	−	−	−	−	−	−	−	−	−	−
UTP6	+	+	+	+	+	+	+	+	−	−	−	−	−	−	+	−	−
JJAZ1 Ex. 10	+	+	+	+	+	+	+	+	+	+	+	+	+	−	+	−	−
LRRC37B	+	+	+	+	+	+	+	+	+	+	+	+	+	+	+	−	−
ZNF207	+	+	+	+	+	+	+	+	+	+	+	+	+	+	+	−	−
PSMD11	+	+	+	+	+	+	+	+	+	+	+	+	+	+	+	−	−
PSMD11	+	+	+	+	+	+	+	+	+	+	+	+	+	+	+	−	−
MYO1D	+	+	+	+	+	+	+	+	+	+	+	+	+	+	+	−	−
MYO1D	+	+	+	+	+	+	+	+	+	+	+	+	+	+	+	−	−

+: not deleted; −:deleted

Table S25: Genomic positions of the MLPA probes included in the P122-C1 kit (MRC Holland, The Netherlands) as well as the custom-designed MLPA probes (marked in bold) used to narrow down the breakpoint regions of the atypical *NF1* deletions investigated in this study. The custom-designed MLPA probes were established in our previous study (Vogt et al. 2012) in order to distinguish type-2 deletions with breakpoints located in *SUZ12* and *SUZ12P* from deletions that do not harbour proximal and distal deletion breakpoints within these paralogs.

**Probe designation**	**Probe position on chromosome 17 (hg19)**
TRAF4 9176-L9350	27,074,291-27,074,314
TRAF4 8620-L8632	27,075,052-27,075,075
SSH2 9635-L9920	27,963,580-27,963,603
SSH2 9634-L9919	28,022,495-28,022,518
BLMH 9627-L9912	28,599,612-28,599,635
BLMH 9626-L9911	28,618,478-28,618,501
CPD 9628-L9913	28,770,910-28,770,933
CPD 9629-L9914	28,789,420-28,789,443
**Atyp_9**	28,880,465-28,880,552
SUZ12p 11798-L12590	29,058,391-29,058,414
SUZ12p 11800-L12591	29,058,839-29,058,862
***SUZ12P* Int. 3**	29,068,410-29,068,495
SUZ12p 11801-L12592	29,085,145-29,085,168
***SUZ12P* Int. 5**	29,091,509-29,091,598
***SUZ12P* Int. 6**	29,094,856-29,094,921
***SUZ12P* Int. 8**	29,098,304-29,098,365
***SUZ12P* Int. 9**	29,107,598-29,107,655
CRLF3	29,111,573-29,111,654
CRLF3 3780-L3289	29,124,380-29,124,403
ATAD5 3781-L3290	29,162,044-29,162,067
CENTA2 3782-L3291	29,253,873-29,253,896
RNF135 3783-L3292	29,311,688-29,311,711
NF1 Ex. 1 2491-L1922	29,421,598-29,421,621
NF1 Ex. 12B 2507-L1938	29,552,202-29,552,225
NF1 Ex. 23-2 2512-L1943	29,576,023-29,576,046
NF1 Ex. 40 2525-L1956	29,676,152-29,676,175
NF1 Ex. 48 5220-L3309	29,687,576-29,687,599
**Atyp_8**	29,860,542-29,860,599
**Atyp_7**	29,888,256-29,888,317
**Atyp_6**	29,929,659-29,929,724
**Atyp_5**	29,982,488-29,982,557
**Atyp_3**	30,078,526-30,078,603
**Atyp_2**	30,129,572-30,129,653
**Atyp_1**	30,184,072-30,184,157
UTP6 3785-L3294	30,202,348-30,202,371
**Atyp_10**	30,250,614-30,250,699
***SUZ12* before Ex. 1**	30,262,903-30,262,984
***SUZ12* Int. 4**	30,276,270-30,276,335
***SUZ12* Int. 4_3**	30,289,757-30,289,826
***SUZ12* Int. 6**	30,301,500-30,301,575
***SUZ12* Int. 8**	30,304,954-30,305,031
JJAZ1 Ex. 10 3786-L3295	30,315,410-30,315,433
***SUZ12* Ex. 13**	30,321,630-30,321,683
**Atyp_11**	30,336,103-30,336,192
LRRC37B 3787-L3296	30,348,569-30,348,592
ZNF207 9637-L9949	30,693,753-30,693,776
PSMD11 9632-L9917	30,773,979-30,774,002
PSMD11 9633-L9918	30,796,071-30,796,094
MYO1D 9631-L9916	31,094,710-31,094,733
MYO1D 9630-L9915	31,107,652-31,107,675

Table S26: Results of the MLPA investigation of the 17 atypical *NF1* deletions by means of the MLPA probes included in the P122-C1 kit (MRC Holland, The Netherlands) as well as the custom-designed MLPA probes (marked in bold) used to narrow down the deletion breakpoint regions. The genomic positions of the respective probes and their complete designation are given in Table S25.

**MLPA-probe**	**Patients**																
	D06.1047	70969	DA-77	ASB4-55	08D2261	100206	61541	1106	D1008345	2535	R84329	R48018	Ak-47005	659	D0801587	619	D05.2678
TRAF4	+	+	+	+	+	+	+	+	+	+	+	+	+	+	+	+	+
TRAF4	+	+	+	+	+	+	+	+	+	+	+	+	+	+	+	+	+
SSH2	+	+	+	+	+	+	+	+	+	+	+	+	+	+	−	+	+
SSH2	+	+	+	+	+	+	+	+	+	+	+	+	+	+	−	+	+
BLMH	+	+	+	+	+	+	+	+	+	+	+	+	+	+	−	+	−
BLMH	+	+	+	+	+	+	+	+	+	+	+	+	+	+	−	+	−
CPD	+	+	+	+	+	+	+	+	+	+	+	+	+	+	−	+	−
CPD	+	+	+	+	+	+	+	+	+	+	+	+	+	+	−	+	−
**Atyp_9**	+	+	+	+	+	+	+	+	+	+	+	+	+	+	−	+	−
SUZ12P	+	+	+	+	+	+	+	−	+	+	+	+	+	−	−	−	−
SUZ12P	+	+	+	+	+	+	+	−	+	+	+	+	+	−	−	−	−
***SUZ12P* Int. 3**	+	+	+	+	+	−	+	−	+	+	+	+	+	−	−	−	−
SUZ12P	+	+	+	+	+	−	−	−	+	+	−	−	−	−	−	−	−
***SUZ12P* Int. 5**	+	+	+	+	+	−	−	−	+	+	−	−	−	−	−	−	−
***SUZ12P* Int. 6**	+	−	+	+	+	−	−	−	−	+	−	−	−	−	−	−	−
***SUZ12P* Int. 8**	+	−	+	+	+	−	−	−	−	+	−	−	−	−	−	−	−
***SUZ12P* Int. 9**	+	−	−	−	−	−	−	−	−	−	−	−	−	−	−	−	−
CRLF3	+	−	−	−	−	−	−	−	−	−	−	−	−	−	−	−	−
CRLF3	+	−	−	−	−	−	−	−	−	−	−	−	−	−	−	−	−
ATAD5	+	−	−	−	−	−	−	−	−	−	−	−	−	−	−	−	−
CENTA2	+	−	−	−	−	−	−	−	−	−	−	−	−	−	−	−	−
RNF135	−	−	−	−	−	−	−	−	−	−	−	−	−	−	−	−	−
NF1 Ex. 1	−	−	−	−	−	−	−	−	−	−	−	−	−	−	−	−	−
NF1 Ex. 12B	−	−	−	−	−	−	−	−	−	−	−	−	−	−	−	−	−
NF1 Ex. 23	−	−	−	−	−	−	−	−	−	−	−	−	−	−	−	−	−
NF1 Ex. 40	−	−	−	−	−	−	−	−	−	−	−	−	−	−	−	−	−
NF1 Ex. 48	−	−	−	−	−	−	−	−	−	−	−	−	−	−	−	−	−
**Atyp_8**	+	−	−	−	−	−	−	+	−	−	−	−	−	−	+	−	−
**Atyp_7**	+	−	−	−	−	−	−	+	−	−	−	−	−	−	+	−	−
**Atyp_6**	+	−	−	−	−	−	−	+	−	−	−	−	−	−	+	−	−
**Atyp_5**	+	−	−	+	−	−	−	+	−	−	−	−	−	−	+	−	−
**Atyp_3**	+	−	−	+	−	+	−	+	−	−	−	−	−	−	+	−	−
**Atyp_2**	+	−	+	+	+	+	−	+	−	−	−	−	−	−	+	−	−
**Atyp_1**	+	+	+	+	+	+	−	+	−	−	−	−	−	−	+	−	−
UTP6	+	+	+	+	+	+	+	+	−	−	−	−	−	−	+	−	−
**Atyp_10**	+	+	+	+	+	+	+	+	+	−	+	+	+	−	+	−	−
***SUZ12* b Ex. 1**	+	+	+	+	+	+	+	+	+	+	+	+	+	−	+	−	−
***SUZ12* Int. 4**	+	+	+	+	+	+	+	+	+	+	+	+	+	−	+	−	−
***SUZ12* Int. 4_3**	+	+	+	+	+	+	+	+	+	+	+	+	+	−	+	−	−
***SUZ12* Int. 6**	+	+	+	+	+	+	+	+	+	+	+	+	+	−	+	−	−
***SUZ12* Int. 8**	+	+	+	+	+	+	+	+	+	+	+	+	+	−	+	−	−
JJAZ1 Ex. 10	+	+	+	+	+	+	+	+	+	+	+	+	+	−	+	−	−
***SUZ12* Ex. 13**	+	+	+	+	+	+	+	+	+	+	+	+	+	−	+	−	−
**Atyp_11**	+	+	+	+	+	+	+	+	+	+	+	+	+	−	+	−	−
LRRC37B	+	+	+	+	+	+	+	+	+	+	+	+	+	+	+	−	−
ZNF207	+	+	+	+	+	+	+	+	+	+	+	+	+	+	+	−	−
PSMD11	+	+	+	+	+	+	+	+	+	+	+	+	+	+	+	−	−
PSMD11	+	+	+	+	+	+	+	+	+	+	+	+	+	+	+	−	−
MYO1D	+	+	+	+	+	+	+	+	+	+	+	+	+	+	+	−	−
MYO1D	+	+	+	+	+	+	+	+	+	+	+	+	+	+	+	−	−

+: not deleted; −: deleted

Table S27: List of PCR primers and their sequences used for the breakpoint-spanning PCRs.

Patient	Forward primer			Reverse primer		
	**Designation**	**Sequence 5'→ 3'**	**Position (hg19)**	**Designation**	**Sequence 5'→ 3'**	**Position (hg19)**
D1008345	Jun4for	TTTTTGTAAGCCAAGATATTCTTCTA	29,091,530-29,091,555	D100_1rev	GGAGGCAGTCAAATCTGAGG	30,218,675-30,218,694
659	BK35for	GGTACTGTGGCCCTGGAGT	28,948,463-28,948,481	BK35rev	TAACCCCTCTCCTGTGTCCA	30,345,583-30,345,602
1106	1106_23for	TGCAAAAATGGTAGTGATTCAAA	28,999,815-28,999,837	1106_23rev	CTGGGGCAGAAGGAGTCAG	29,766,012-29,766,030
100206	Ala1_2for	GCCATTGATTGAATACCTTTTGA	29,061,720-29,061,742	Ala1_2rev	CTAGCCCCATCACGTCAGTC	30,020,861-30,020,880
61541	Ala6_1for	CTGCTCTTTGGTCACTTCAGAAC	29,079,305-29,079,327	Ala6_1rev	CTCTTTTTCCCCAAGCAGTTAAT	30,188,006-30,188,028
70969	Ala7_3for	TGGTATTCAAAATTAACACCCCTAA	29,091,503-29,091,527	Ala7_1rev	CATGAAAAGGTGGACTCTCAAAC	30,175,954-30,175,976
619	CK_121for	GGGACTATAGGCTTGCACCA	28,945,738-28,945,757	CK_122rev	CCCAGTGGAACCAAATCAAA	31,954,773-31,954,792
D0801587	D080_2for	ATATTTGGGCCCATGTTACGAT	27,721,045-27,721,066	D080_2rev	TACAAAGGCAGCCAGCAAGTT	29,730,310-29,730,330
D05.2678	49_D05_2for	TCCAACGAAAAGATTTTCACC	28,140,018-28,140,038	49_D05_1rev	GAGAAAGAAGGAGCAGGGATT	34,114,857-34,114,877
D06.1047	57_1for	TGATGACCACTTGGTTTTGC	29,261,541-29,261,560	57_1rev	TGTGACCTGCTAGTTCTTGGAA	29,784,378-29,784,399
R84329	R84_1for	TGTTGTCTGCATGGGTAGAGA	29,067,202-29,067,222	R84_1rev	CTGTCCACTTGGAAAGAGGTG	30,224,933-30,224,953
2535	Ala7_2for	TTTGTTTTGTGAGTGGTTTATTTACA	29,093,307-29,093,332	2535_1rev	ACGTGTCTGAGCGGAAGAAG	30,250,936-30,250,955
08D2261	49_1for	GATGGCAAGAAACACAGACG	29,098,312-29,098,331	49_1rev	TGCTTTTGTGCAATGTCTGG	30,080,001-30,080,020
R48018	R48_2for	TCTATTTTGTACATTTAGCTGCATTTT	29,081,049-29,081,075	R48_3rev	GGGGAAAAGTATTTTCAAACTCA	30,244,915-30,244,937
DA-77	GSP1_for	TTTTAGGCAGCATGGGGTATGTTCTG	29,099,021-29,099,046	GSP1_rev	TCTGATCATCCATACGTGACACACTGA	30,103,175-30,103,201
ASB4-55	as117for	CCCAGAATTCCATAGTTACCAGATTCA	29,102,524-29,102,550	as146Brev	TGATCTACTGACAGGTTACCCTTGGA	29,971,139-29,971,164
Ak-47055	AK_4for	TTTCTATTTTGTACATTTAGCTGCATT	29,081,047-29,081,073	2535_1rev	ACGTGTCTGAGCGGAAGAAG	30,250,936-30,250,955

Table S28: List of restriction enzymes used for the inverse PCR experiments performed in order to identify the deletion breakpoints in patients DA-77 and ASB4-55.

**Patient**	**Breakpoint region analysed**	**Restriction enzyme**	**Genomic position (hg19)**
DA-77	telomeric	*Eco*53KI[a]	30,103,521
		*Pvu*II	30,103,539
		*Dra*I	30,103,996
		*Ava*II	30,105,425
		*Avr*II	30,105,830
ASB4-55	centromeric	*Pci*I[a]	29,100,547
		*Hinc*II[a]	29,101,984
ASB4-55	telomeric	*Pci*I	29,972,011
		*Hinc*II	29,972,017
		*Eco*53KI	29,972,654
		*Sac*I	29,972,656
		*Bae*GI	29,973,013

a: Restriction enzyme used successfully to characterize the deletion breakpoint.

Table S29: Primers used for the inverse PCR assays performed to identify the deletion breakpoints of patients DA-77 and ASB4-55.

**Patient analysed**	**Primer**	**Sequence (5'→3')**	**Genomic position (hg19)**
DA-77	ADinv_3rev	CATATCTTGCCTCTTGCTGCTA	30,103,219-30,103,240
	ADinv_3for	CACTAGGTTCCAGTCCCTTGTT	30,103,433-30,103,454
ASB4-55	as_inv2rev	CCACTACTAAACACCATTCTTCAA	29,102,350-29,102,373
	as_inv1for	GACACATATGTACACACACCTTAAAA	29,102,413-29,102,438
ASB4-55	as47rev	TAATTGTCATTTCAACAATTGATCTAC	29,971,157-29,971,183
	as48for	AATGTTGTCTGTAGGCATTCAGGAG	29,971,227-29,971,251
ASB4-55	as54for	TGTAGGCATTCAGGAGATAGCACA	29,971,236-29,971,259
	As54rev	TGATCTACTGACAGGTTACCCTTGGA	29,971,139-29,971,164
ASB4-55	as63rev	GAGAAGCAAAAGAAAACTCAATCA	29,971,813-29,971,836
	as64for	AACCAAAAATGAACAAATTAACAGAAT	29,971,898-29,971,924

Table S30: Forward and reverse primers used for the semi-specific PCRs performed to identify unknown inserted sequences at the deletion breakpoints of patient DA-77. The region-specific forward and reverse primers are located within non-deleted regions close to the deletion breakpoints as determined by array CGH.

**Primer designation**	**Breakpoint analysed**	**Sequence (5'→3')**	**Genomic position (hg19)**
as79for	centromeric	GGCAAGAAACACAGACGTACAATAAT	29,098,315-29,098,340
AD67for	centromeric	TGCAATTTCTTTTTGGAAACG	29,098,344-29,098,364
AD38for	centromeric	GGCTGCGTGCTGGTAGTTA	29,098,474-29,098,492
as96for	centromeric	TCAGAAATTTTATTGTGGATCGAA	29,098,882-29,098,905
AD70for	centromeric	GATTTAAATGGAAAACAATAGAACCA	29,098,906-29,098,931
AD71for	centromeric	GAAAATAGTGGTCATGTCTGTGG	29,098,943-29,098,965
AD72for	centromeric	AATTAGAATGAGGCGCATTGG	29,098,973-29,098,993
AD88for	centromeric	CAGCATGGGGTATGTTCTG	29,099,028-29,099,046
AD89for	centromeric	TTTTTAATCACTGTACCTGACACATA	29,099,053-29,099,078
AD90for	centromeric	AAGTGCCAAGAAGAAACTGG	29,099,129-29,099,148
AD91for	centromeric	CTGGTGACAGCGAGACTCTG	29,099,439-29,099,458
AD92for	centromeric	TTCAAAACTATAGGAAAGTTGAAAGAA	29,099,481-29,099,507
AD93for	centromeric	GGGATTCTAATGAGTGATGTGTTC	29,099,979-29,100,001
AD68rev	telomeric	GTTGGGGGAGGATTAGGGTA	30,103,336-30,103,355
AD69rev	telomeric	CAGGACTCCTCTGGCTGTTT	30,103,314-30,103,333
AD23rev	telomeric	TTCAAGCTTCCCAGCAAAGT	30,103,153-30,103,172

Primer egalAAL (5'-TGAATTCGATCAAAAAAAAAAAAAA-3') was used as the non-specific primer for each PCR. The same set of experiments was also performed with primer egalTTL (5'- TGAATTCGATCTTTTTTTTTTTTTT-3') as the non-specific primer.

Table S31: Forward and reverse primers used for the semi-specific PCRs performed to identify unknown inserted sequences at the deletion breakpoints of patient ASB4-55. The region-specific forward and reverse primers are located within non-deleted regions close to the deletion breakpoints as determined by array CGH.

**Primer designation**	**Breakpoint analysed**	**Sequence (5'→3')**	**Genomic position (hg19)**
as101for	centromeric	GAAGAGAAGCCAGTTGCTTGA	29,102,303-29,102,323
AD32for	centromeric	CTGAGTATGGTTGAAGAATGGTG	29,102,340-29,102,362
as115for	centromeric	TCCCTAAGACAAGCATTGTAACAC	29,102,645-29,102,668
as16rev	telomeric	TGCTGGTAGAGAGTGAAGAATGA	29,971,391-29,971,413
as37rev	telomeric	AAAAACCATCATAATAAAATGCAAA	29,971,324-29,971,348
as116rev	telomeric	TCTGGATTATTGGGCAGGAC	29,971,263-29,971,282

Primer egalAAL (5'-TGAATTCGATCAAAAAAAAAAAAAA-3') was used as the non-specific primer for each PCR.

Table S32: List of all restriction enzymes used for the GenomeWalker™ analysis.

Patient	Breakpoint region	Restriction enzyme	Position (hg19)
DA-77	centromeric	*Pvu*II[a]	29,092,597
		*Eco*RV	29,097,395
		*Nla*IV	29,098,714
		*Eco*53KI	29,098,791
		*Swa*I[a]	29,098,910
		*Msl*I[a]	29,098,959
DA-77	telomeric	*Nla*IV	30,103,440
		*Eco*53KI[a]	30,101,577
		*Pvu*II	30,103,539
		*Dra*I	30,103,996
		*Swa*I	30,103,996
		*Stu*I	30,105,979
		*Eco*RV	30,106,421
ASB4-55	centromeric	*Bmg*BI	29,089,196
		*Nae*I	29,092,295
		*Bsa*BI	29,095,322
		*Eco*RV	29,097,395
		*Eco*53KI	29,098,791
		*Swa*I	29,098,910
		*Hpa*I	29,099,560
		*Stu*I	29,099,895
		*Pvu*II	29,102,267
		*Msl*I	29,102,507
		*Nla*IV	29,102,520
		*Bst*Z17I	29,102,581
ASB4-55	telomeric	*Hinc*II	29,972,017
		*Bsa*BI	29,972,145
		*Eco*53KI	29,972,654
		*Stu*I	29,973,908
		*Hpa*I	29,977,639
		*Bmg*BI	29,981,530
		*Bst*Z17I	29,983,906
		*Eco*RV	29,995,481
		*Nae*I	30,003,122
		*Swa*I	30,012,166
		*Fsp*I	30,015,466

a: The indicated restriction enzyme was successfully used to characterize the deletion breakpoint and to identify the inserted SVA element.

Table S33: Gene-specific primers (GSPs) used to perform the GenomeWalker™ analysis. The given GSP was combined either with primer AP1 (5'-GTAATACGACTCACTATAGGGC-3') or primer AP2 (5'-ACTATAGGGCACGCGTGGT-3').

**Patient analysed**	**Breakpoint region**	**Primer**	**Sequence (5'→3')**	**Genomic position (hg19)**
DA-77	centromeric	GSP1_for	TTTTAGGCAGCATGGGGTATGTTCTG	29,099,021-29,099,046
DA-77	centromeric	GSP2_for	AAAAGTGCCAAGAAGAAACTGGGAGAA	29,099,127-29,099,153
DA-77	telomeric	GSP1_rev	TCTGATCATCCATACGTGACACACTGA	30,103,175-30,103,201
DA-77	telomeric	GSP2_rev	AGGCTTGTGTGTCTCTTATGCTTGCTC	30,103,108-30,103,134
ASB4-55	centromeric	GSP3_for	AGTGTTTCCCTAAGACAAGCATTGTAACAC	29,102,639-29,102,668
ASB4-55	centromeric	GSP4_for	ACCAGGTTTTGAGACCTCAGGCATATTA	29,103,038-29,103,065
ASB4-55	telomeric	GSP3_rev	TTGATCTACTGACAGGTTACCCTTGGAA	29,971,138-29,971,165
ASB4-55	telomeric	GSP4_rev	CAATATTTTAGAACCTGTTCTTTTCACTTG	29,971,063-29,971,092

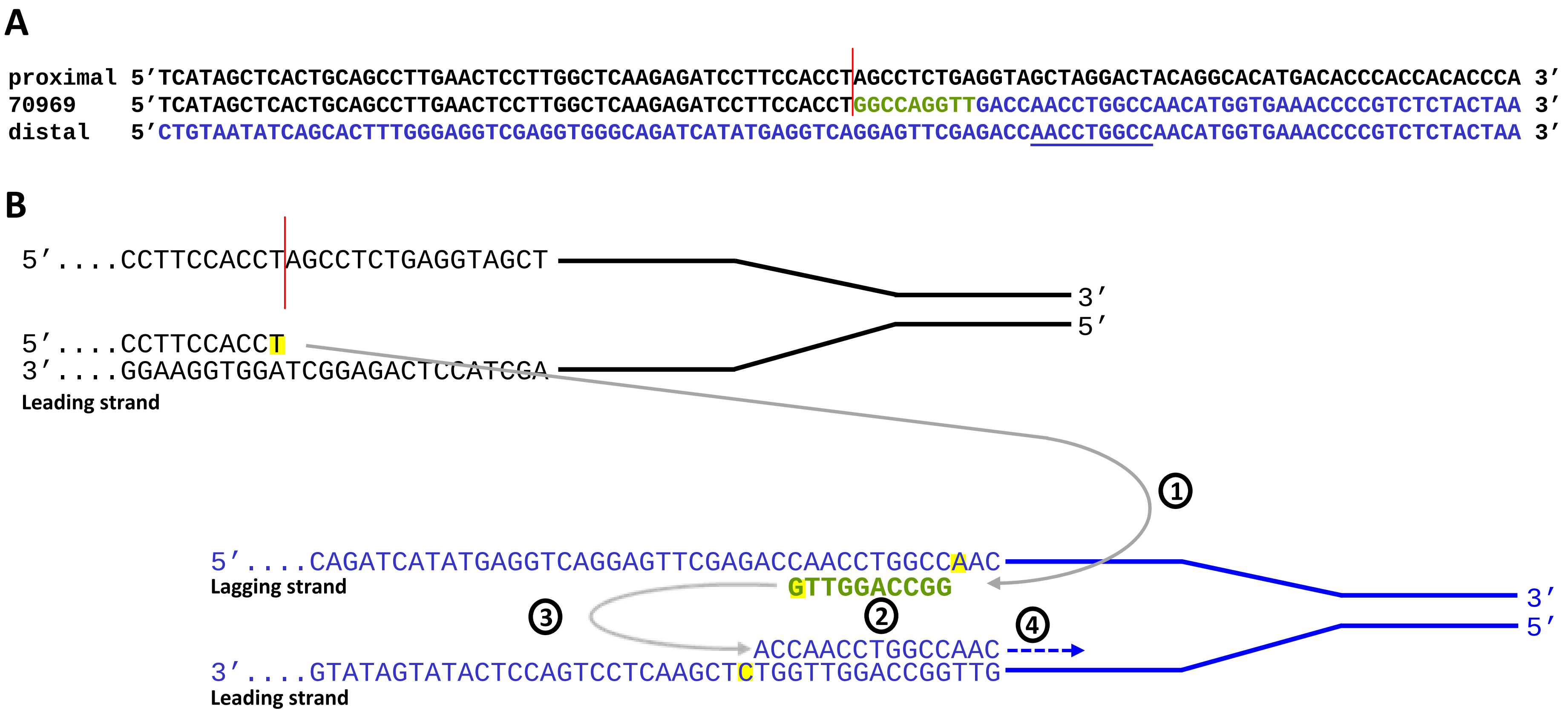

Figure S1: The insertion of 9-bp (green) at the deletion breakpoints of patient 70969 may have occurred in association with the occurrence of the large *NF1* deletion mediated by replication-associated template switching. **(A)** Alignment of the deletion breakpoint-flanking sequences of patient 70969 against the reference sequence of the human genome (hg19). Sequences located at the proximal (centromeric) deletion breakpoint are indicated in black, whilst sequences at the distal (telomeric) breakpoint are given in blue. The vertical red line highlights the position of the proximal deletion breakpoint. The 9-bp insertion (green) represents a duplication of 9-bp from the distal breakpoint region (underlined). **(B)** In the proximal breakpoint-flanking region, DNA synthesis at the leading strand is interrupted but appears to have resumed, after an interstrand template switch, at sequences located within the distal breakpoint-flanking region (blue) (**step 1**). Subsequently, the 9-bp indicated in green are newly synthesized and included in the nascent DNA strand at the replication fork located in the distal breakpoint region (**step 2**). This is then followed by another template switch occurring onto the leading strand (**step 3**) upon which replication is continued (**step 4**). The nucleotides exhibiting microhomology at sites of template switching are marked in yellow.

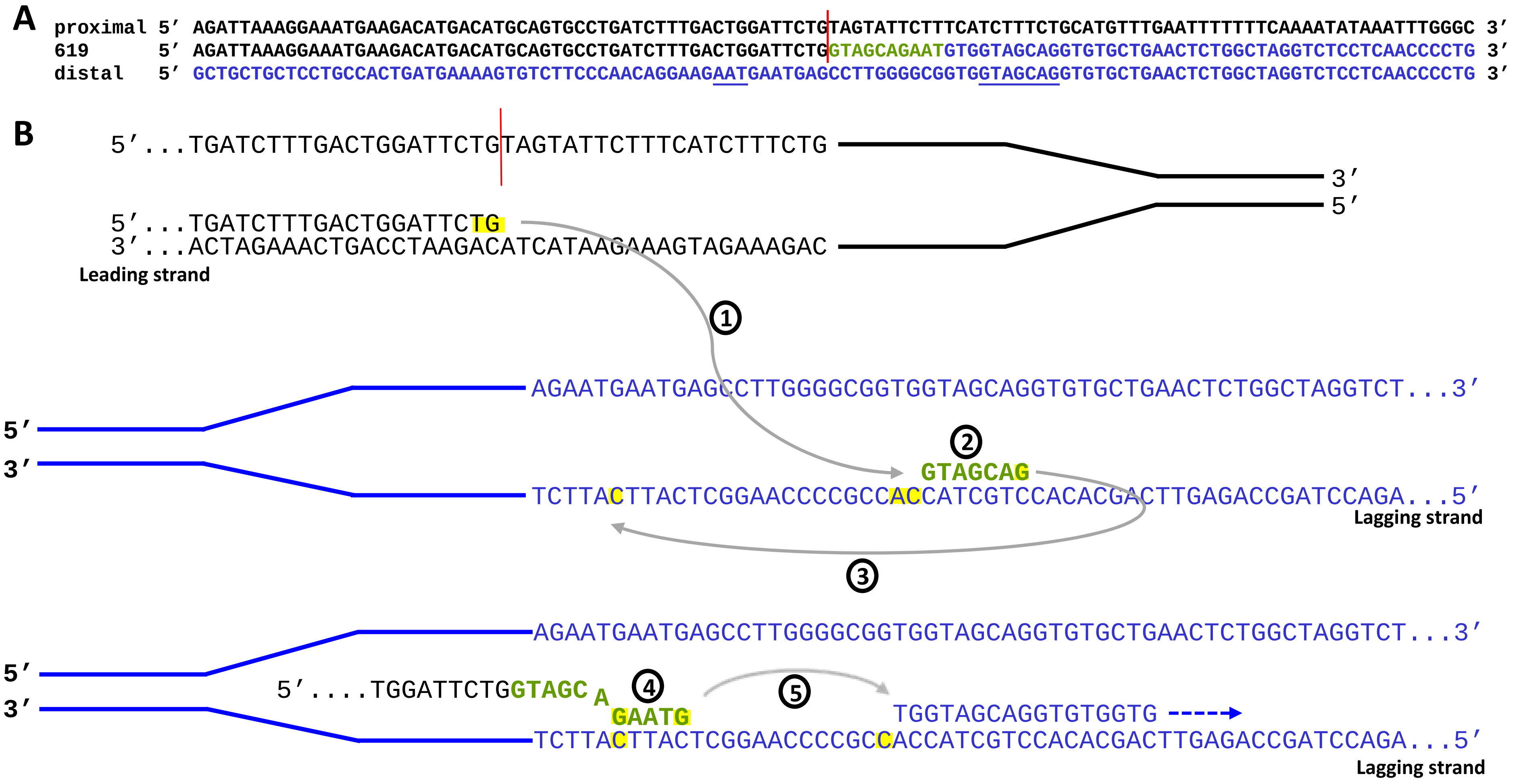

Figure S2: The insertion of 10-bp at the deletion breakpoint of patient 619 is likely to have occurred concomitantly with the large *NF1* deletion mediated by template switching during replication. **(A)** Alignment of the deletion junction sequences against the reference sequence of the human genome (hg19). Sequences at the proximal (centromeric) deletion breakpoint are indicated in black, whilst sequences at the distal (telomeric) breakpoint are given in blue. The vertical red line represents the proximal deletion breakpoint. The insertion of 10-bp (green) appears to represent a duplication of the underlined sequences. **(B)** Within the proximal breakpoint-flanking region, DNA synthesis at the leading strand is interrupted but continues after an interstrand template switch into a replication fork located within the distal breakpoint-flanking region (blue) (**step 1**). The 7-bp sequence indicated in green is newly synthesized and included within the nascent DNA strand (**step 2**). Subsequently, a further template switch occurs which involves sequences located in 3′ direction, also within the lagging strand template, causing the insertion of the trinucleotide 'AAT' (**step 4**). Finally, another template switch occurs (**step 5**) and replication is continued within the distal breakpoint-flanking region. The nucleotides exhibiting microhomology at sites of template switching are marked in yellow.

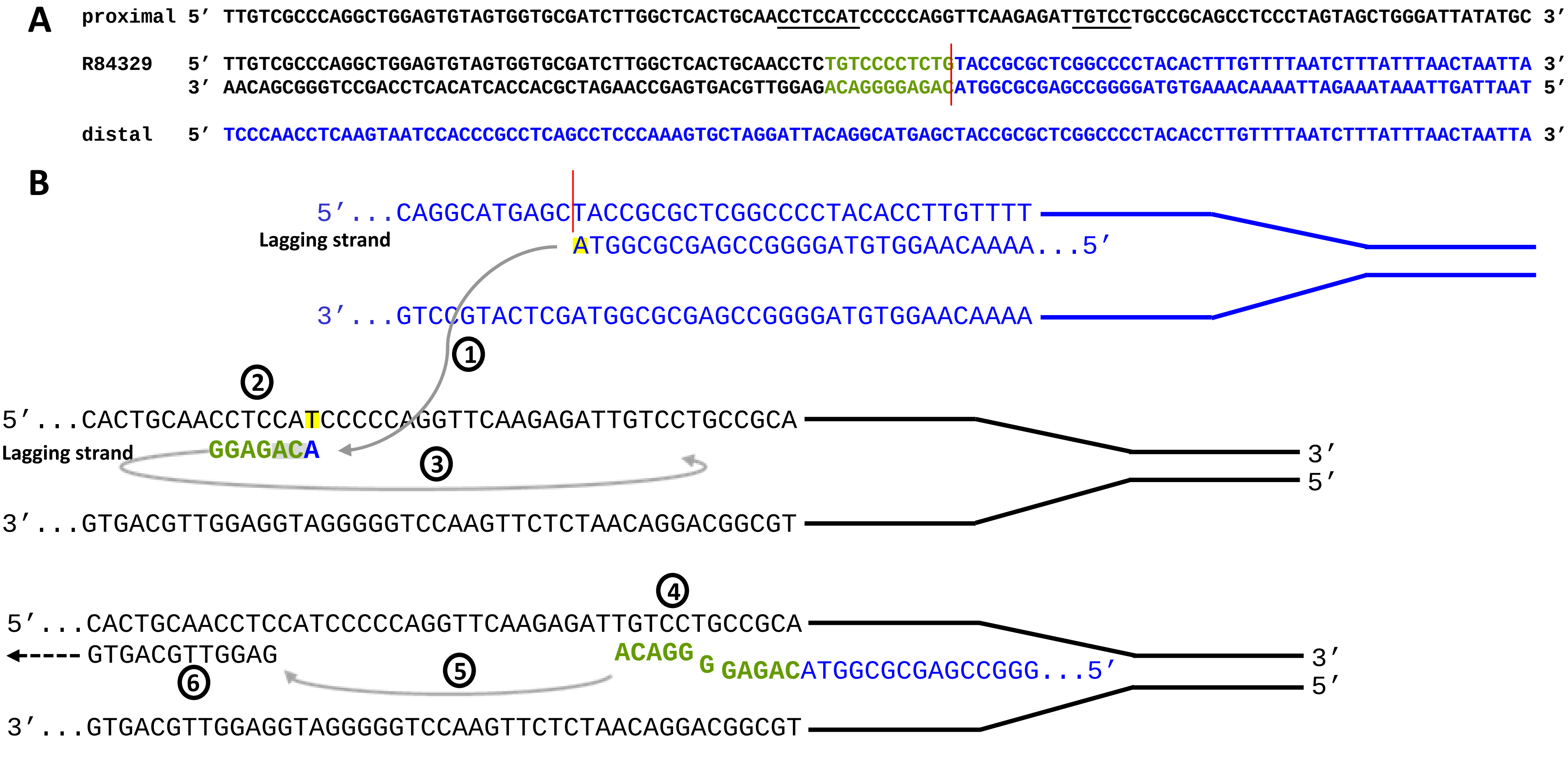

Figure S3: The insertion of 11-bp at the deletion breakpoint junction of patient R84329 is likely to have occurred concomitantly with the large *NF1* deletion mediated by replication-associated template switching. **(A)** Alignment of the deletion breakpoint-flanking sequences against the reference sequence of the human genome (hg19). Sequences at the proximal (centromeric) deletion breakpoint are indicated in black, whilst sequences at the distal (telomeric) breakpoint are given in blue. The vertical red line highlights the position of the distal deletion breakpoint. The insertion of 11-bp (green) appears to represent a duplication of pre-existing sequences (underlined). **(B)** In the distal breakpoint-flanking region, DNA synthesis at the lagging strand stops and an interstrand template switch occurs into a replication fork located within the proximal breakpoint-flanking region (black) (**step 1**). The hexanucleotide 'GGAGAC' (green) is included within the nascent DNA strand (**step 2**). Single nucleotide changes due to DNA polymerase errors are highlighted in grey. Subsequently, another template switch occurs (**step 3**) causing the insertion of the five additional nucleotides indicated in green (**step 4**). Finally, a further template switch occurs (**step 5**) followed by continued replication within the proximal breakpoint-flanking region (**step 6**). The nucleotide exhibiting microhomology at the site of template switching is marked in yellow.

Patient 08D2261
```
proximal    GCCAAGATGCTGAAACCCGTCTCTACTAAAAATACAAAAATTAGCCGGGCATGGTGGCACGCGCCTGTAATCCCAGCTACTCGGGAGGCTGAGGCAGGAGAATCGCTTGAACCTGG
deletion    GCCAAGATGCTGAAACCCGTCTCTACTAAAAATACAAAAATTAGCCGGGCATGGTGGCAACCACACCAGGCCTATTTTGTGCATTTTAACACAATTGTTAAAGTGGTAAATTTTAT
distal      CAACTCAAGCGATCTTCCCACTTTGGCCTCCCAAAGTGCTGGGATTACAGGCATAAGCCACCACACCAGGCCTATTTTGTGCATTTTAACACAATTGTTAAAGTGGTAAATTTTAT
```

Patient 100206
```
proximal    TGAAATCAATAGGATGAAGGTTTTTTAAAAACAATGAAGTCTTAAATCATTAGATTTTAAAGTGGGGTCTGGAGGTGGAAGACAGCTTATTTCTGCTGTGTAACATTTAGTACTTA
deletion    TGAAATCAATAGGATGAAGGTTTTTTAAAAACAATGAAGTCTTAAATCGTTAGATTTAAGTACTCCAACCTGGGTCACAGAGCAAGACTGTGTCTCTAAATAAAGAAGGCAAGAAA
distal      GTGGGAGGACTGCTTGAGCTGAGGGGGTGAGGGTTGCAGTGAGCTGTGATCATGCCACTGTACTCCAACCTGGGTCACAGAGCAAGACTGTGTCTCTAAATAAAGAAGGCAAGAAA
```

Patient D1008345
```
proximal    AGAAGTGGGCAGATTGCTTGAACCCAGGAGTTAAGCATCCTGGGCAACATGGCGAAACCCTGTCTCTACCAAAAACACGAAAATGAGCTAGGCATGATGGCCTGTGCCTGTAGTCC
deletion    AGAAGTGGGCAGATTGCTTGAACCCAGGAGTTAAGCATCCTGGGCAACATGGCGAAACCAATGGTGGCAAGCAATTGCTTTGGGCTTCCTTGAACATGTGACTCCTAAAATTGAGC
distal      ATTGCTTTTGTTAAAAATGACGCCTGATTGGTAACCACACCTGGAATAGAAGGGTCAGCAATGGTGGCAAGCAATTGCTTTGGGCTTCCTTGAACATGTGACTCCTAAAATTGAGC
```

Patient D05.2678
```
proximal    CCGCCCGCCTTGGCCTCCCAAAATGCTGGGATTACAGGTGTTAGCCACCGTGCCTGGCCCCTATTTTTTGTATTTTTTAGTAGAGACGGGGTTTTGCCATGTTGGCCAGGCTGGTC
deletion    CCGCCCGCCTTGGCCTCCCAAAATGCTGGGATTACAGGTGTTAGCCACCGTGCCTGGCCCTGTGGAGTCTGGGTAAAATGGAACTAGTTCTCCTGGGACCCAGCCAGCTTGGTGGA
distal      CTTGGTTAGGGCTGGGACCACTTTCCTGGGGAGTCACTTCTGAAAGTCCCTGGGAGTGGCTGTGGAGTCTGGGTAAAATGGAACTAGTTCTCCTGGGACCCAGCCAGCTTGGTGGA
```

Patient 70969
```
proximal    TGACAGGATCATAGCTCACTGCAGCCTTGAACTCCTTGGCTCAAGAGATCCTTCCACCTAGCCTCTGAGGTAGCTAGGACTACAGGCACATGACACCCACCACACCCAGCTAATTT
deletion    TGACAGGATCATAGCTCACTGCAGCCTTGAACTCCTTGGCTCAAGAGATCCTTCCACCTGGCCAGGTTGACCAACCTGGCCAACATGGTGAAACCCCGTCTCTACTAAAAATACAA
distal      GCTCACTCCTGTAATATCAGCACTTTGGGAGGTCGAGGTGGGCAGATCATATGAGGTCAGGAGTTCGAGACCAACCTGGCCAACATGGTGAAACCCCGTCTCTACTAAAAATACAA
```

Patient 619
```
Proximal    TTTTAGATTAAAGGAAATGAAGACATGACATGCAGTGCCTGATCTTTGACTGGATTCTGTAGTATTCTTTCATCTTTCTGCATGTTTGAATTTTTTTCAAAATATAAATTTGGGCA
deletion    TTTTAGATTAAAGGAAATGAAGACATGACATGCAGTGCCTGATCTTTGACTGGATTCTGGTAGCAGAATGTGGTAGCAGGTGTGCTGAACTCTGGCTAGGTCTCCTCAACCCCTGA
distal      TCCTGCTGCTGCTCCTGCCACTGATGAAAAGTGTCTTCCCAACAGGAAGAATGAATGAGCCTTGGGGCGGTGGTAGCAGGTGTGCTGAACTCTGGCTAGGTCTCCTCAACCCCTGA
```

Patient R84329
```
proximal    GTCTCACATTGTCGCCCAGGCTGGAGTGTAGTGGTGCGATCTTGGCTCACTGCAACCTCCATCCCCCAGGTTCAAGAGATTGTCCTGCCGCAGCCTCCCTAGTAGCTGGGATTATA
deletion    GTCTCACATTGTCGCCCAGGCTGGAGTGTAGTGGTGCGATCTTGGCTCACTGCAACCTCTGTCCCCTCTGTACCGCGCTCGGCCCCTACACTTTGTTTTAATCTTTATTTAACTAA
distal      TCTCCAACTCCCAACCTCAAGTAATCCACCCGCCTCAGCCTCCCAAAGTGCTAGGATTACAGGCATGAGCTACCGCGCTCGGCCCCTACACCTTGTTTTAATCTTTATTTAACTAA
```

Patient 61541
```
proximal    ATATTCCTTGTATAATTTTTTTGGTTTTGTAGTAATTCCTTTGTTTTCTTCACAGTACTTCTGCAGAGTGATCCATTTGTAATTAATAGACACACACACATCATCCACCATTCAGC
deletion    ATATTCCTTGTATAATTTTTTTGGTTTTGTAGTAATTCCTTTGTTTGCTTCACAGTACTACATGTGCCTTGGAGCTACTGTTTCTAACAGTGCAACTCTAGGTGTACTAGCTAGAT
distal      AGCACTTGAAATGTGGCCAATGTGAATAAATTGAATAATTCTAATAAATTTAAATTGCTACATGTGCCTTGGAGCTACTGTTTCTAACAGTGCAACTCTAGGTGTACTAGCTAGAT
```

Figure S4

```
Patient 2535
proximal      AAGAAGTTTACCAAAATAGATATATATGGTTCTTAAACAATTAAAACGATAAAACATCTGAATTAAAAGTACACTGAGATGCGTTTTTTCACTTATCAGATTGACAGAGACCAAAA
deletion      AAGAAGTTTACCAAAATAGATATATATGGTTCTTAAACAATTAAAACGATAAAACATCTTAGGCTCTTGAAAAGGTCTTTAAGACTCAGATTTCCAAACTATTTATTATAAGCCAT
distal        TATTTATTTACTTGCAAAAGGCTCCATCTTAGTGTTCTTTGAAGGAGGAGTCAGTAGCTTAGGCTCTTGAAAAGGTCTTTAAGACTCAGATTTCCAAACTATTTATTATAAGCCAT

Patient R48018
proximal      TCATACCTGTAATCTCAGCACTGGGAGGCCAGGGCGGGTGGATCACCTGAGGTCAGGAGTTCTAGACCAGCCTGGTCAATGTGGTGAAACCTCATCTTTACTAAAAATTAGGAAAA
deletion      TCATACCTGTAATCTCAGCACTGGGAGGCCAAGGCGGGTGGATCACCTGAGGTCAGGAGTCATAAAAAAAAAAAGAAAGAAAAAGCAGCCAGGCACTGTGGCTCAAGCCTGTAATC
distal        AGGCAAGTTGCAGAACAGTATATTGAATAATCCATTTTAAATATGTGTATGTGTATGTGTCATAAAAAAAAAAAGAAAGAAAAAGCAGCCAGGCACTGTGGCTCAAGCCTGTAATC

Patient Ak-47055
proximal      GTAAAAATATTCCTTGTATAATTTTTTTGGTTTTGTAGTAATTCCTTTGTTTTCTTCACAGTACTTCTGCAGAGTGATCCATTTGTAATTAATAGACACACACACATCATCCACCA
deletion      GTAAAAATATTCCTTGTATAATTTTTTTGGTTTTGTAGTAATTCCTTTGTTTTCTTCATTTAAAATAACTTCATTCACAAGGTTCATTGAACAGCTGGTTTGTGCCCATCACAGGC
distal        TTAAGCACACAGGTATTGTAGGCTAGAGGCCGCTTTTGGCTACAAACCCCCTTATTCATTTAAAATAACTTCATTCACAAGGTTCATTGAACAGCTGGTTTGTGCCCATCACAGGC

Patient D06.1047
proximal      GACCTCGTGATCCACCCACCTCACCCTCCCAAAGTGCTGGGATTACAGGCGTGAGCCACCGCGCCCGGCCTATTTATTTATTTTTTGAGATGGGATTTCACTCTATTGCTCAGGTT
deletion      GACCTCGTGATCCACCCACCTCACCCTCCCAAAGTGCTGGGATTACAGGCGTGAGCCACCCTGCTGGCCCCTAGTACATTCTTTTTAGGATTTTGTACCAGAAAAGAAGTCCTTCC
distal        GCTCAAGCGATCCGCCCCCCTCAGCCTCCCAAAGTGCTGGAATTACAGGCGTGGGCCACCCTGCTGGCCCCTAGTACATTCTTTTTAGGATTTTGTACCAGAAAAGAAGTCCTTCC

Patient 659
proximal      CCGGGGGTAGTGGCGGGCACCTTTAGTCCCAGCTACTCGGGAGGCTGAGGCAGGAGAATGGCGTTATCCCAGGGGGTGGAGCTTGCAGTGAGCAGAGATCACGCCACTGCACTCCA
deletion      CCGGGGGTAGTGGCGGGCACCTTTAGTCCCAGCTACTCGGGAGGCTGAGGCAGGAGAATCGCTTGAACCAAGGAGGTGGAGGTTGTGGTGAGCCAAGATCATGTCATTGCACTCCA
distal        ACATGGGGGAAACGGTGGTGCATGCATCCCAGCTACTCTGGAGGCTGAGGCAGGAGAATCGCTTGAACCAAGGAGGTGGAGGTTGTGGTGAGCCAAGATCATGTCATTGCACTCCA

Patient 1106
proximal      AAAAATTTAAAATTAGGCCAGGCACAGTTGCTGACGCCTGTAATCCCAGCACTTTGGGAAGCCAAGGCAGCAGATCACTTGAGGCCAGGAGTTCGAGACCAGCCTGGCCAACATGG
deletion      AAAAATTTAAAATTAGGCCAGGCACAGTTGCTGACGCCTGTAATCCCAGCACTTTGGGAGGCTGAGGTGGGAGGATTACTTGAGCCCAGGGGTTTGAGACCAGCCTGGACAATATA
distal        TTTTTAAGACTTACTTGCCGGGAGTGGTGGCTCATGCCTGTAATCCCAGCACTTTGGGAGGCTGAGGTGGGAGGATTACTTGAGCCCAGGAGTTTGAGACCAGCCTGGACAATATA

Patient D0801587
proximal      GCCCGCCACTATGCCTGGCTAAATTTTTGTATTTTTATTAGAGACGGGGTTTCACCGTGTTAGCCAGGATGGTCTTGATCTCCTGACCTCGTGATCTGCCCGCCTTGGCCTCCCAA
deletion      GCCCGCCACTATGCCTGGCTAAATTTTTGTATTTTTATTAGAGACGGGGTTTCACCGTGTTAGCCAGGATGGTCTCGATCTCCTGACCTTGTGATCCGCCCGCCTTGGCCTCCCAA
distal        ACCATCACTCCTGGCTAATTTTTTTTTTGTATTTTTAGTAGAGACGGGGTTTCACCGTGTTAGCCAGGATGGTCTCGATCTCCTGACCTTGTGATCCACCCGCCTTGGCCTCCCAA
```

Figure S4: Alignments of the deletion junction sequences of the 15 atypical *NF1* deletions against the reference sequence of the human genome (hg19). The reference sequences at the proximal (centromeric) and distal (telomeric) breakpoint flanking regions are given in black and blue, respectively. Microhomology at the breakpoints is indicated in red and is characterized by one or more perfectly matching nucleotides at the breakpoints. Microinsertions of nucleotides at the deletion breakpoints are indicated in green and sequence mismatches between the junction sequence and the reference sequence is marked in grey. SNPs are highlighted in turquoise.

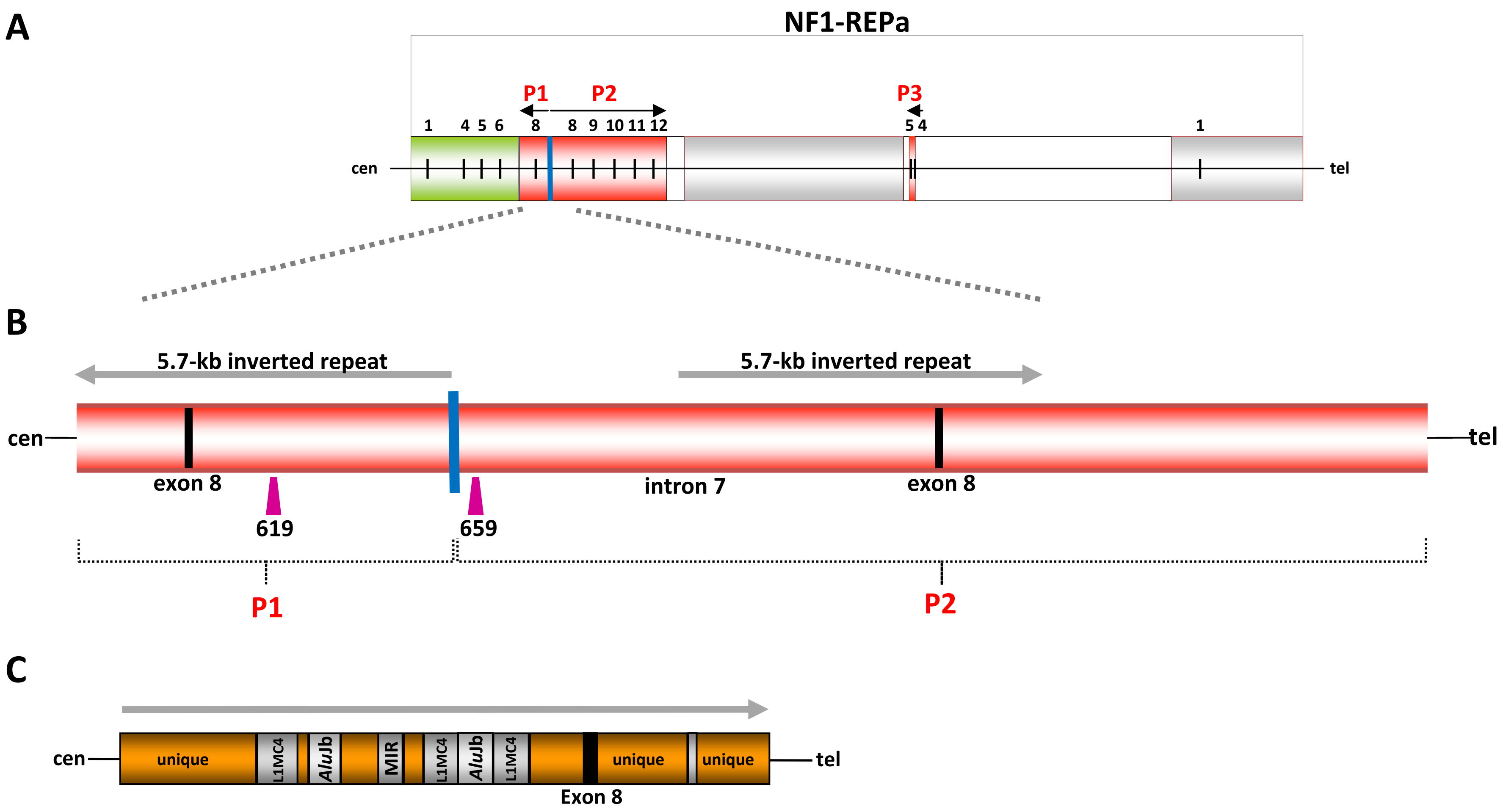

Figure S5: Location of the centromeric breakpoints of the atypical *NF1* deletions identified in patients 619 and 659 within NF1-REPa. **(A)** Sequence composition of NF1-REPa harbouring *LRRC37B-P* (red) and *SMURF2-P* (green), both pseudogenes. The numbers of the exons located within these pseudogenes are indicated. Additionally, NF1-REPa contains sequences that are highly homologous to chromosome 19p13.12 (marked in grey). The black arrows indicate the genomic orientation of the three *LRRC37B* pseudogene fragments (designated P1–P3). P1–P3 represent partial duplications of the *LRRC37B* gene located within NF1-REPc (not shown). **(B)** Inverted repeats of 5.7-kb were identified within *LRRC37B*-P1 and P2 (indicated by grey arrows). The centromeric deletion breakpoint in patient 619 (genomic position: 28,946,218; hg19) is located within the centromeric 5.7-kb inverted repeat. The centromeric deletion breakpoint in patient 659 (genomic position: 28,948,946; hg19) is located 48-bp telomeric to the 5.7-kb repeat. The relative positions of both breakpoints are indicated by lilac triangles. The 5.7-kb inverted repeats exhibit 99% sequence identity and are separated by 3,710-bp. We surmise that these repeats contributed to the occurrence of the large *NF1* deletions by forming a hairpin structure, thereby inducing a DNA double strand break. The sequence composition of the 5.7-kb repeat is shown in **(C)**.

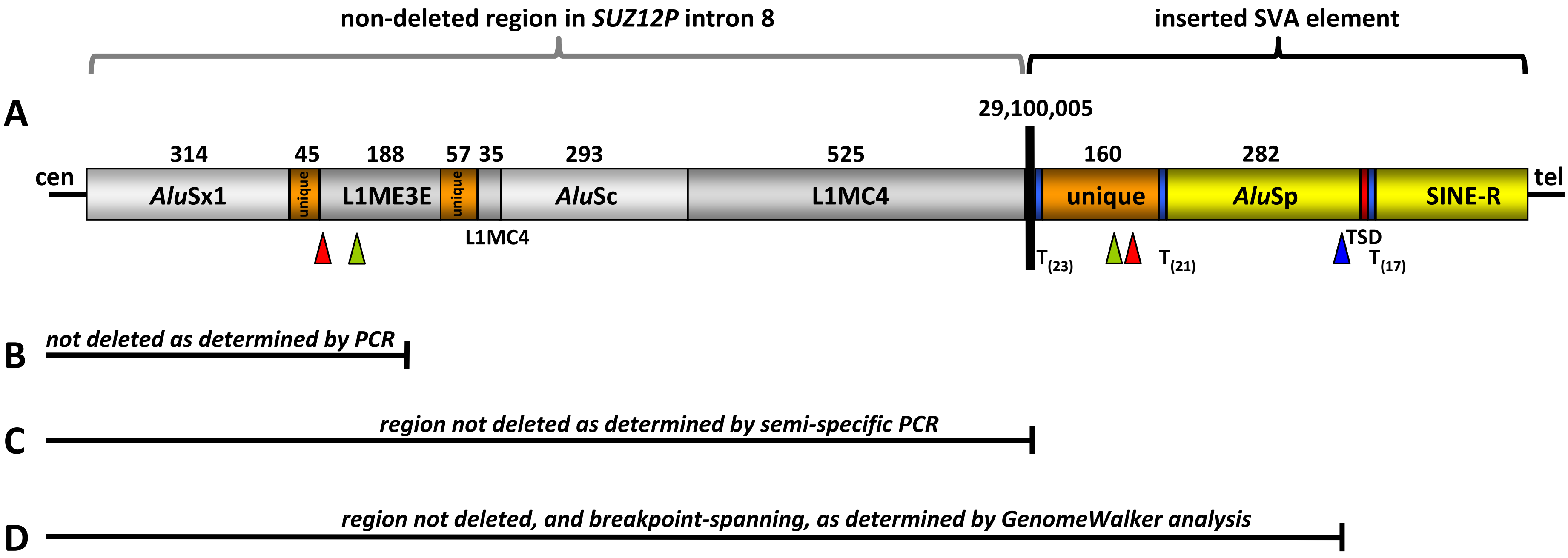

Figure S6: Identification of the centromeric deletion breakpoint in patient DA-77. **(A)** The breakpoint of the deletion is located within *SUZ12P* intron 8. The genomic region that is not deleted is marked by a grey bracket. The lengths of the repetitive and unique regions are indicated in base-pairs. **(B)** Relative extent of the region not deleted as determined by PCR using DNA isolated from somatic hybrid cells containing only the chromosome 17 with the deletion and not the normal chromosome 17 from the patient. **(C)** Semi-specific PCR indicated that the breakpoint was located within an L1MC4 element. At the breakpoint, a poly$T_{(23)}$ tract was observed that is not present at the corresponding position of the human genome reference sequence. **(D)** Identification of the SVA element insertion by means of GenomeWalker analysis using the restriction enzymes *Pvu*II (blue triangle), *Msl*I (green triangle) and *Swa*I (red triangle). Genomic position 29,100,005 demarcates the breakpoint of the deletion and the insertion site of the SVA element.

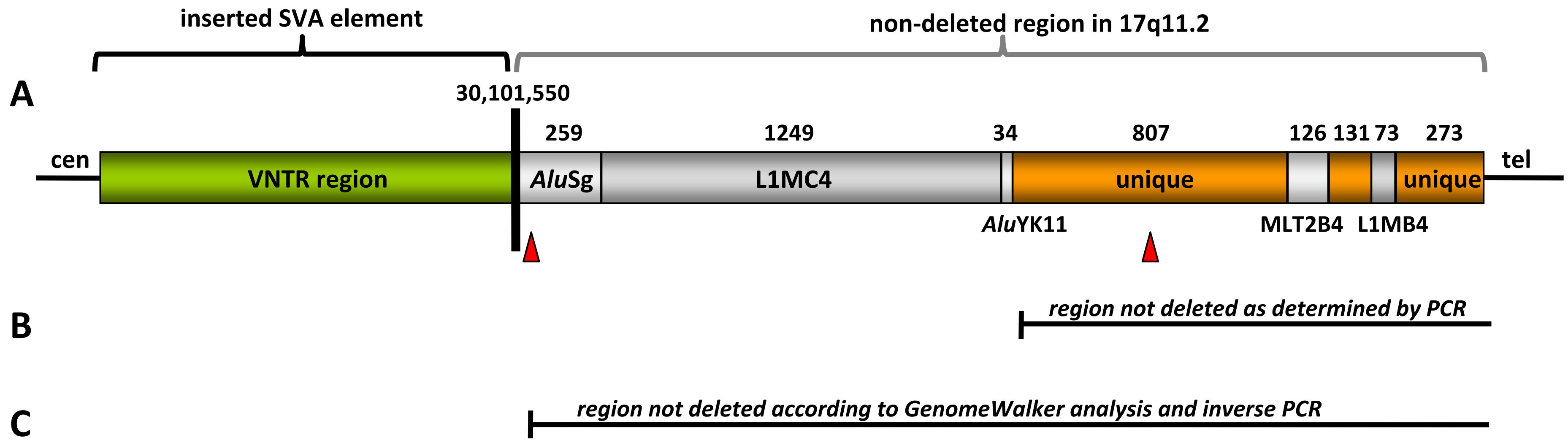

Figure S7: Analysis of the telomeric breakpoint region of the *NF1* deletion in patient DA-77. **(A)** The breakpoint is located within an intergenic region between the *RAB11FIP4* and *COPRS* genes at 17q11.2. The genomic region in 17q11.2 that is not deleted is marked by a grey bracket. The lengths of the repetitive and unique sequences located within the breakpoint-flanking regions are indicated in base-pairs. **(B)** Relative extent of the region not deleted, as determined by PCR using DNA isolated from somatic hybrid cells containing only the chromosome 17 with the deletion from the patient. **(C)** GenomeWalker analysis and inverse PCR using the restriction enzyme *Eco*53KI (restriction sites are marked by red triangles) indicated that the genomic region encompassing positions 30,101,577-30,103,521 is not deleted. Breakpoint-spanning PCR with primers depicted in Figure S8 revealed that the deletion breakpoint is located at position 30,101,550 within a truncated *Alu*Sg element of 259-bp. The corresponding full-length *Alu*Sg element in the reference sequence of the human genome spans 304-bp.

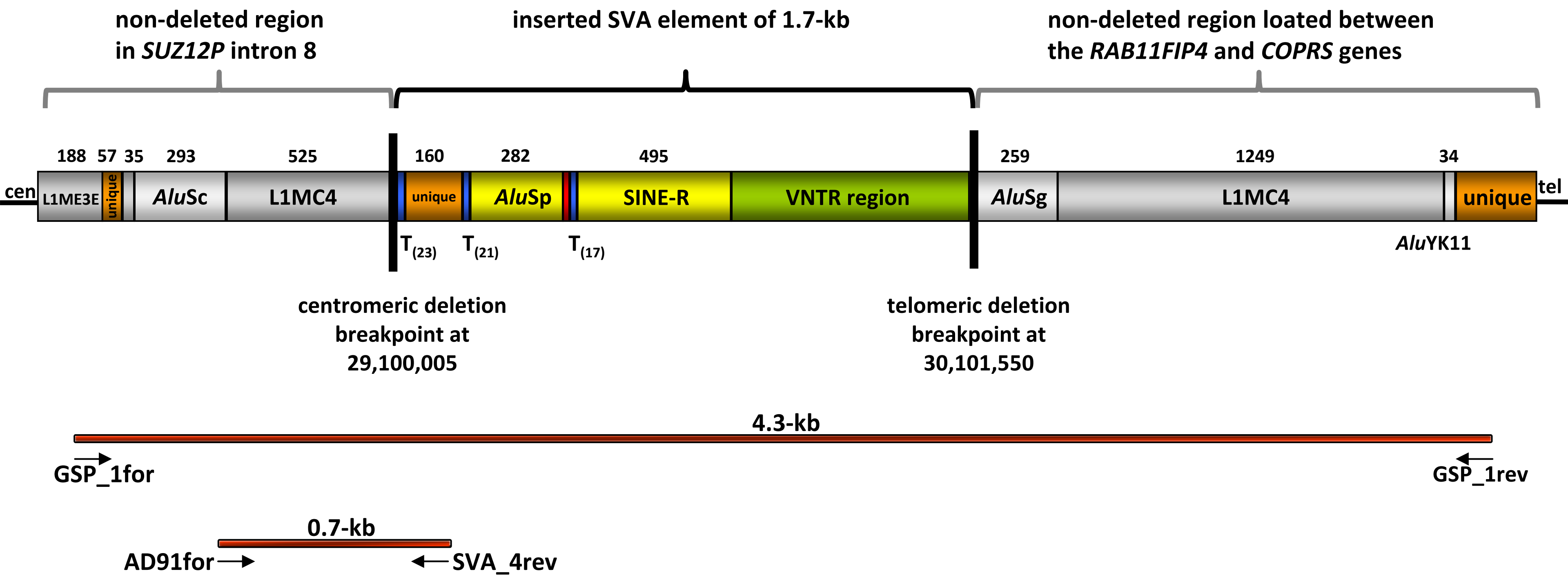

Figure S8: PCRs performed in order to amplify across the SVA element inserted at the breakpoints of the atypical *NF1* deletion identified in patient DA-77. The centromeric and the telomeric breakpoint regions within 17q11.2 and the inserted SVA element are indicated. Breakpoint-spanning PCRs were performed with primers GSP_1for and GSP_1rev (indicated by arrows). PCR with primers AD91for and SVA_4rev was also performed to characterize the insertion site of the SVA element. Sequence analysis of these PCR products revealed the structure of the inserted SVA element at the deletion breakpoints in patient DA-77 and indicated that the centromeric deletion breakpoint was located within intron 8 of *SUZ12P* whereas the telomeric breakpoint was located in an intergenic region between the *RAB11FIP4* and *COPRS* genes.

Figure S9: Sequence of the inserted SVA element identified at the deletion breakpoints of patient DA-77.

TTT**TTTTTTTTTTTTTTTTTT**TTTGATTGTAAAGATATTTATTTCTTAGA $T_{(23)}$, poly-adenylation
GACTTTCTAAGAAGGAAAAGGCATATAAGTAAATCTTATTAGACCTTCAC signal, unique
TGTAAAGGACATATCATATTTATTCATACACATGCTGGAATTATTGGTGC poly-adenylation signal
AGACATTTAAATACATTTTCTTTGAGAAAGTCCTTTTTTTTTTTTTTTTT $T_{(21)}$
TTTTGATGGAGTTTCCCTCTTGTTGCCCAGGCTGGAGTGCAATGGTGCAA *Alu*Sp
TCTCAGCTCACAACAACCTCTGCCTCCTGGGTTCAAGCAATTCTCCTGCC
TCAGCCTCCCAAGTAGCTGGGATTACAGGCATGCACCACCACGCCCAGCT
AATTTTTTTTATTTTTAGTAGAGACGGGGTTTCTCCGTGTTGGTCAGGCT
GGTCTTGAACTCCTGATCTCAGGTGATCTGCCCACCTTGGCCTGCCACAG
TGCTGGGATTACAGTCGTGAGCCACCACAGCTGGCCTGGGAAAGTCCATT TSD
CTTTTTTTTCTTTTTTTTTTTTTTTTTAAATTTATTTTTTTATTGATAAT $T_{(17)}$, poly-adenylation
TCTTGGGTGTTTCTCACAGAGGGGGATTTGGCAGGGTCATGGGACAATAG signal, SINE-R
TGGAGGGAAGGTCAGCAGATAAACAAGTGAACAAAGGTCTCTGGTTTTCC
TAGGCAGAGGACCCCGCGGCCTTCCGCAGTGTTTGTGTCCCTGATTACTT
GAGATTAGGGATTGGTGATGACTCTTAACGAGCATGCTGCCTTCAAGCAT
CTGTTTAACAAAGCACATCTTGCACTGCCCTTAATCCATTTAACCCTGAG
TGGACACAGCACATGTTTCAGAGAGCACAGGGTTGGGGGTAAGGTCACAG
ATCAACAGGATCCCAAGGCAGAGGAATTTTTCTTAGTGCAGAACAAAATG
AAAAGTCTCCCATGTCTACTTCTTTCTACACAGACACGGCAACCATCCGA
TTTCTCAATCTTTTCCCCACCTTTCCCGCCTTTCTATTCCACAA**A**GCCGC
CATTGTCATCCTGGCCCGTTCTCAATGAGCTGTTGGGCACACCTCCCAGA VNTR
CGGGGTGGTGGCCGGGCAGAGGGGCTCCTCACTTCCCAGTAGGGGCGGCC
GGGCAGAGGCGCCCCTCACCTCCCGGACGGGGCGGCTGGCCGGGCAGGGG
GGCTGACCCCCCCCACCTCCCTCCCGGACGGGGCGGCTG**C**CCGGGCGGGG
GGCTGACCCCCCAACCTCCCTCCCGGACGGGGCGGCTGGCCGGGCGGGGG
GCTGACCCCCCCACCTCCCTCCCGGACGGGGCGGCTGGCCGGGCAGAGGG
GCTCCTCACTTCCCAGTAGGGGCGGCCAGGCAGAGGCGCCCCTCACCTCC
CGGACGGGGCGGCTGGCCGGGCAGGGGGGCCGACCCCCCCCACCTCCCTC
CCGGACGGGGCGGCTGGCCGGGCGGGGGGCCGACCCCCCCACCTCCCTCC
CGGACGGGGCGGCTGGCCGGGCGGGGGGCCGACCCCCCCACCTCCCTCCC
GGACGGGGCGGCTGGCCGGGCAGAGGGGCTCCTCACTTCCCAGTAGGGGC
GGCCGGGCAGAGGCGCCCCTCACCTCCCAGACGGGGCGGCTGGCCGGGCG
GAGGGCTGAGCCCCCCATCTCCCTCCCGGACGGGGTGGCTGGCCGGGCTG
AGGGGCTCCTCACTTCCCAGTAGGGGCGGCCGGGCAGAGGCGCCCCTCAC
CTCCCGGACGGGGCGGCTGGCCGGGCGGGGGGCTGACCCCCCCACCTCCC
TCCCGGATGGCACGGCTGGCCGGGCGGGGG

Bold letters: nucleotides that differ from the source element H10_1
TSD: target site duplication
VNTR: variable number of tandem repeats

Figure S10: Sequence of the full-length SVA element H10_1, genomic position 101,596,732-101,600,770; hg19.

AAGAATGGACTTTTACTGTGACAGGCCTAGGGGGTCTGGAGGGCTCTGGT TSD, 5’TD
TCACACCTCAGGATGCCTGGAGCCCCTAGGTTTTCTGATTTCCTATCTCC
ATCCTCACTGGCAGGAAAGCTTCTGGAACTAGGAGAGGGTTGCTTAAGAG
GATGAGGGGTCAGGACCAGAGATGGAGGAGGAAAAGAAAGCTCACAGGTG
GCTGGGCGCAGTGGCTCACACCTATAATCCCAGCGCTTTGGGAGGCTGAG *Alu*Sc
GCGGGCGGATCATGAGGTCAAGAGATTGAGATGATCCTGGCCAACATGGT
GAAACCCCTTCTCTACTAAAAACAAACAAACAAAAATAGCTGGGCGTGGT
GGTGCACACCTGTAGTCCCAGCTACTCAGGAGGCTGAGGCTGAGGCAGGA
AAATCGCTTCAACCTGGGAGGCAGAGGTTGCAGTGAGCCGAGATTGCACC
ACTGCACTCCACCCAGACAACAGAGCAAGACTCCGTCTCAAAAAATAAAA
TAAAATAAAAGGAAAAGAAAAAAGAAATTTCAGGCAGAGGAGGTCGCGGC *MAST2* Exon1
GCCGGAGGCCCCAGAAGGGTCGAAGGCGCCGCGGGCTGGGGTCGGTGGCT
TAGGGAGCCCGTCCGGCCATGGTGGCCGCGGCTGGTGGTTGGCGCGGCTG
CGCTGCGGCCCGGGGCAGTGCGGAGCCAGGACAGTCGCGGCGCTGACGCC
CGCGGGCCCCAGCTGCAGATATGAAGCGGAGCCGCTGCCGCGACCGACCG
CAGCCGCCGCCGCCCGACCGCCGGGAGGATGGAGTTCAGCGGGCAGCGGA
GCTGTCTCAGTCTTTGCCGCCGCGCCGGCGAGCGCCGCCCGGGAGGCAGC
GGCTGGAGGAGCGGACGGGCCCCGCGGGGCCCGAGGGCAAGGAGCAGCCG *Alu*-like
CCTGCCTTGGCCTCCCAAAGTGCCGAGATTGCAGCCTCTGCCCGGCTGCC VNTR
ACCCCGTCTGGGAAGTGAGGAGTGTCTCTGCCTGGCCGCCCATCGTCTGG
GATGTGAGGAGCCCCTCTGCCTGGCTGCCCAGTCTGGAAAGTGAGGAGCG
TCTCCGCCCGGCCGCCATCCCATCTAGGAAGTGAGGAGCGCCTCTTCCCA
GCCGCCATCACATCTAGGAAGTGAGGAGCGTCTCTGCCCGGCCGCCCATC
GTCTGAGATGTGGGGAGCGCCTCTGCCCCTCCGCCCCATCTGGGATGTGA
GGAGCGCCTCTGCCCGGCCGAGACCCCGTCTGGGAGGTGAGGAGCGTCTC
TGCCCGGCCGCCCCGTCTGAGAAGTGAGGAGACCCTCTGCCTGGCAACCA
CCCCGTCTGAGAAGTGAGGAGCCCCTCCGCCCGGCAGCTGCCCCGTCTGA
GAAGTAAGGAGCCTCTCCGCCCGGCAGCCACCCCATCTGGGAAGTGAGGA
GCGTCTCCGCCCGGCAGCCACCCCGTCCGGGAGGGAGGTGGGGGGGGTC
AGCCCCCCGCCCGGCCAGCCGCCCCATCCGGGAGGGAGGTGGGGGGTCAG
CCCCCCGCCCGGCCAGCCGCCCCATCCGGGAGGGAGGTGGGGGGTCAGCC
CCCCCGCCCGGCCAGCCACCCCGTCCGGGAGGGAGGTGGGGGGGTCAGCC
CCCCGCCCGGCCAGCCGCCCCGTCCGGGAGGGAGGTGGGGGGGGGTCAGC
CCCCCCGCCCAGCCAGCCGCCCTGTCCGGGAGGTGAGGGGCGCCTCTGCC
CGGCCGCCCCTACTGGGAAGTGAGGAGCCCCTCTGCCCGGCCAGCCGCCC
CGTCCGGGAGGGAGGTGGGGGGTCGGCCCCCCGCCCGGCCAGCCGGCCC
GTCCGGGAGGGAGGTGGGGGGTTGGCCCCCCGCCCGGCCAGCCGCCCCG
TCCGGGAGGGAGGTGGGGGGTCGGCCCCCCGCCCGGCCAGCCGCCCCGT
CCGGGAGGGAGGTGGGGGGGGGTCTGCCCCCCTGCCCGGCCAGCCGCCC
CGTCCGGGAGGTGAGGGGCGCCTCTGCCCGGCCGCCCCTACTGGGAAGTG
AGGAGCCCCTCTGCCCGGCCAGCCGCCCCGTCCGGGAGGGAGGTGAGGGG
GTCGGCCCCCCGCCCGGCCAGCCGCCCCGTCCGGGAGGTGAGGGGCGCCT
CTGCCCGGCCGCCCCTACTGGGAAGTGAGGAGCCCCTCTGCCCGGCCAGC
CGCCCCGTCCGGGAGGGAGGTGGGGGGTCAGCCCCCCGCCCGGCCAGCC
GCCCCGTCCGGGAGGGAGGTGGGGGGGTCAGCCCCCCCGCCCAGCCAGC
CGCCCTGTCCGGGAGGGAGGTGGGGGTCAGCCCCCCGCCCGGCCAGCC
GTGCCATCCGGGAGGGAGGTGGGGGGTCAGCCCCCCGCCCGGCCAGCCG
CCCCGTCCGGGAGGTGAGGGGCGCCTCTGCCCGGCCGCCCCTACTGGGAA
GTGAGGAGCCCCTCAGCCCGGCCAGCCACCCCGTCCGGGAGGGAGATGGG
GGGCTCAGCCCTCCGCCCGGCCAGCCGCCCCGTCTGGGAGGTGAGGGGCG
CCTCTGCCCGGCCGCCCCTACTGGGAAGTGAGGAGCCCCTCTGCCCGGCC
AGCCGCCCCGTCCGGGAGGGAGGTGGGGGGTCGGCCCCCCGCCCGGCCA
GCCGCCCCGTCCGGGAGGGAGGTGGGGGGTCGGCCCCCCGCCCGGCCAG
CCGCCCCGTCCGGGAGGGAGGTGGGGGGGTCGGCCCCCCTGCCCGGCCA
GCCGCCCCGTCCGGGAGGTGAGGGGCGCCTCTGCCTGGCCGCCCCTACTG
GGAAGTGAGGAGCCCCTCTGCCCGGCCAGCCGCCCCGTCCGGGAGGGAGG
TGGGGGGGTCAGCCCCCCGCCCGGCCAGCCGCCCCGTCCGGGAGGGAGGT

```
TGGGGGGTCAGCCCCCCGCCCGGCCAGCCGCCCCGTCCGGGAGGGAGGTG
GGGGGGGTCAGCCCCCCTGCCCGGCCAGCCGCCCCGTCCGGGAGGTGAGG
GGCGCCTCTGCCCGGCCGCCCCTACTGGGAAGTGAGGAGCCCCTCTGCCC
GGCCACCACCCCGTCTGGGAGGTGTGCCCAACAGCTCATTGAGAACGGGC  SINE-R
CAGGATGACAATGGCGGCATTGTGGAATAGAAAGGCGGGAAAGGTGGGGA
AAAGATTGAGAAATCGGATGGTTGCCGTGTCTGTGTAGAAAGAAGTAGAC
ATGGGAGACTTTTCATTTTGTTCTGCACTAAGAAAAATTCCTCTGCCTTG
GGATCCTGTTGATCTGTGACCTTACCCCCAACCCTGTGCTCTCTGAAACA
TGTGCTGTGTCCACTCAGGGTTAAATGGATTAAGGGCAGTGCAAGATGTG
CTTTGTTAAACAGATGCTTGAAGGCAGCATGCTCGTTAAGAGTCATCACC
AATCCCTAATCTCAAGTAATCAGGGACACAAACACTGCGGAAGGCCGCGG
GGTCCTCTGCCTAGGAAAACCAGAGACCTTTGTTCACTTGTTTATCTGCT
GACCTTCCCTCCACTATTGTCCCATGACCCTGCCAAATCCCCCTCTGTGA
GAAACACCCAAGAATTATCAATAAAAAAATAAATTTAAAAAAAAAAAAAA  poly-adenylation signal,
AAAGAAAAAAAAGAATGGACTTTCCCAGGCCAGCTGTGGTGGCTCACGAC  A(17), TSD, AluSp
TGTAATCCCAGCACTGTGGCAGGCCAAGGTGGGCAGATCACCTGAGATCA
GGAGTTCAAGACCAGCCTGACCAACACGGAGAAACCCCGTCTCTACTAAA
AATAAAAAAAATTAGCTGGGCGTGGTGGTGCATGCCTGTAATCCCAGCTA
CTTGGGAGGCTGAGGCAGGAGAATTGCTTGAACCCAGGAGGCAGAGGTTG
TTGTGAGCTGAGATTGCACCATTGCACTCCAGCCTGGGCAACAAGAGGGA
AACTCCATCAAAAAAAAAAAAAAAAAGGACTTTCTCAAAGAAAATGTATT  A(17), unique
TAAATGTCTGCACCAATAATTCCAGCATGTGTATGAATAAATATGATATG  poly-adenylation signal
TCCTTTACAGTGAAGGTCTAATAAGATTTACTTATATGCCTTTTCCTTCT
TAGAAAGTCTCTAAGAAATAAAATATCTTTACAATCAAACTCCTGACCTG  poly-adenylation signal
TCCACATTCCAAATAATGCTGAGCATCTTTAGCGCCTCAAGACACTTTGG
AAGCCACTCAAGAATTTTTCTTCCTGAAAAAGATCTTCCTCTTTCCAGGA
CATACTGCTTGCCAGGGCTTAAGGATACTGGAGAATAATGCTCACCCTGT
TCACAAGACGGAAGCTTCCCTTTCTGTCATCAATGGTCTTACCTAAAAAT
TCCCAACTCTCCTCTCTCCAACCTCCACTGCCTTCACTACCCAGAAAGAG
TGTCATGACTATGGAGAATCTCACATCCGTTCTCAGAAAAGTAAACAGAG
GATGTTCAGTTCACCCAGCACTTATTTGGTACTGGGTTACAAAATGCTCA
CAAATGTCATTTGACTTCCACCATGAGACAGGTGGAACCCTGTGAGATAG
GGTGTGTTCTGGCCATTACAAAAACAAAGAAATGGAGATTCTCCTTAGAG
AAGTCAAGGCAATTCACCCAGCTTGGTAAACGGCAGAGCTGTCATGGGGA
```

5’-TD: 5’ transduction sequence

TSD: target site duplication

VNTR: variable number of tandem repeats

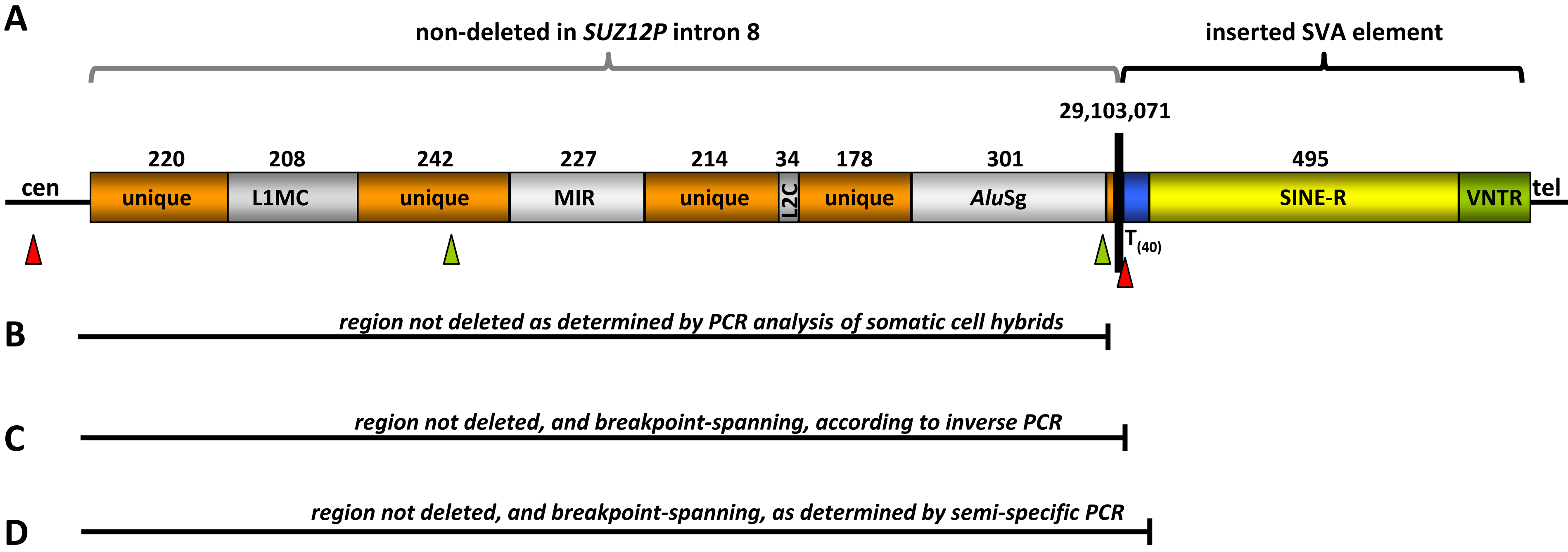

Figure S11: Identification of the centromeric deletion breakpoint in patient ASB4-55. **(A)** The breakpoint of the deletion is located within *SUZ12P* intron 8. The genomic region that is not deleted is indicated by a grey bracket. The lengths of the repetitive elements and unique sequences located within breakpoint-flanking regions are indicated in base-pairs. **(B)** Relative extent of the non-deleted regions as determined by PCR using DNA isolated from somatic hybrid cells containing only the chromosome 17 with the deletion and not the normal chromosome 17 from the patient. **(C)** Inverse PCR after restriction of genomic DNA with *Pci*I (red triangles) and *Hinc*II (green triangles) indicated that the deletion breakpoint lies immediately adjacent to a polyT$_{(40)}$ tract that is not included in the reference sequence of the human genome (hg19). **(D)** Semi-specific PCR confirmed the presence of the polyT tract at the deletion breakpoint. Breakpoint-spanning PCR as indicated in Figure S13 revealed that the breakpoint of the deletion and the insertion of the SVA element occurred at genomic position 29,103,071.

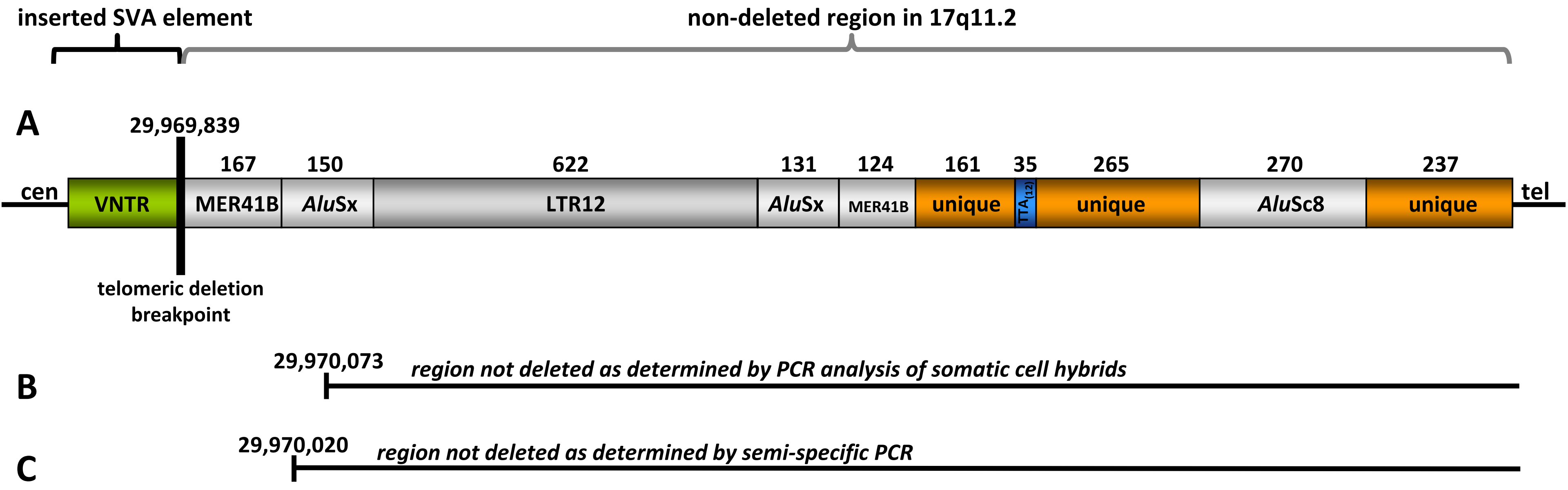

Figure S12: Analysis of the telomeric breakpoint-flanking region in patient ASB4-55. **(A)** The telomeric breakpoint region is located between the *RAB11FIP4* and *COPRS* genes. The lengths of the repetitive elements and unique sequences located close to the deletion breakpoint are indicated in base-pairs. **(B and C)** Array CGH analysis suggested that the breakpoint should be located between nucleotide positions 29,968,972 and 29,971,033. The breakpoint region was further narrowed down by PCR using DNA isolated from somatic cell hybrids containing only the chromosome 17 harbouring the deletion from the patient as well as semi-specific PCR. These experiments indicated that the genomic region telomeric to position 29,970,020 was not deleted. Breakpoint-spanning PCR with primers indicated in Figure S13 revealed that the breakpoint was located within an MER41B element. In the reference sequence of the human genome (hg19), the corresponding MER41B element encompasses 300-bp. In patient ASB4-55, however, the MER41B element is truncated, spanning only 167-bp and is located immediately adjacent to the VNTR region of the inserted SVA element. Genomic position 29,969,839 demarcates the breakpoint of the deletion and the insertion of the SVA element.

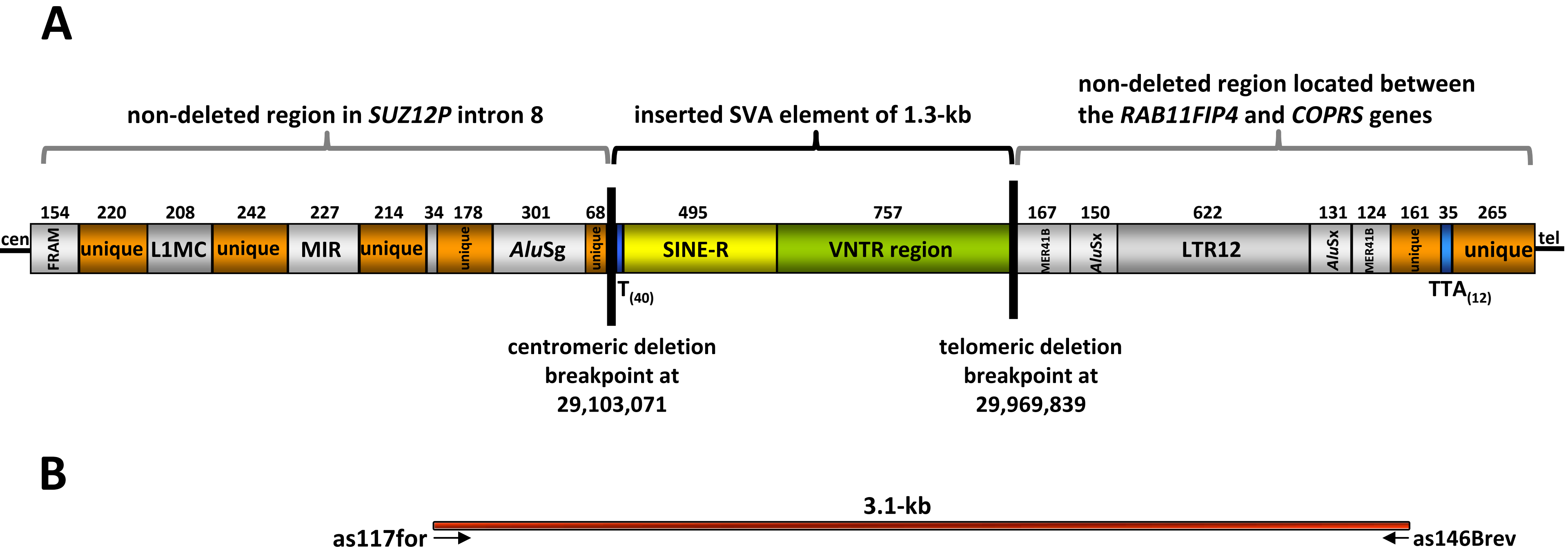

Figure S13: Structure of the deletion breakpoint region in patient ASB4-55. **(A)** Schematic representation of the 17q11.2 region harbouring the SVA insertion-associated *NF1* deletion in this patient. Indicated are the centromeric and telomeric regions flanking the deletion breakpoints within 17q11.2 and the inserted SVA element. The lengths of the repetitive and unique sequences located within the breakpoint-flanking regions are indicated in base-pairs. **(B)** PCR performed in order to amplify across the SVA element inserted at the breakpoints of the atypical *NF1* deletion identified in patient ASB4-55. Breakpoint-spanning PCR was performed with primers as117for and as146Brev. Sequence analysis of the corresponding PCR product indicated the structure of the SVA element as well as its insertion sites.

Figure S14: Sequence of the full-length SVA element H6_1084, genomic position: 123,168,910-123,171,600; hg19.

AAAAGAATACAAAATAGGTGGGGAAAGGGCAAGAAGGCAGGGGAATCACCATGTTTGGGT TSD
GGACCTAGTTTCTAATGGCTTGCATTTACATATCAAAGGTTGCCAGCCTGGCTCTAAGAG 5'TD
CCGGGGCTATACAAGAAACTTTTCCGGCTCTCCCTCTCCCTCTGTCTCCCTCTCCCCACG Hexamer
GTCTCCCTCTCATGCGGAGCCGAAGCTGGACTGTACTGCTGCCATCTCGGCTCACTGCAA *Alu*-like
CCTCCCTGCCTGATTCTCCTGCCTCAGCCTGCCCAGTGCCTGCAATGGCGCCGCCACGCC
TGACTGGTTTTGGTGGAGACGGGGTTTCGCTGTGTTGGCCGGGCCGGTCTCCAGCCCCTA
ACCGCGAGTGATCCCGCCAACCTCAGCCTCCCGAGGTGCCGGGATTGCAGACGGAGTCTC
GTTCACTCAGTGCTCAATGGTGCCCAGGCTGGAGTGCAGTGGCGTGATCTCGGCTCACTA
CAACCTACACCTCCCAGCCGCCTGCCTTGGCCTCCCAAAGTGCCGAGATTGCAGCCTCTG VNTR
CCCGGCCGCCACCCCGTCTGGGAAGTGAGGAGCCTCTCTGCCTGGCCGCCCATCGTCTGG
GATGTGAGGAGCCCCTCTGCCTGGCTGCCCAGTCTGGAAAGTGAGGAGCGTCTCCGCCCG
GCCGCCATCCCATCTAGGAAGTGAGGAGCGCCTCTTCCCAGCCGCCATCACATCTAGGAA
GTGAGGAGCGTCTCTGCCCGGCCGCCCATCGTCTGAGATGTGGGGAGCGCCTCTGCCCCG
CCGCCCCATCTGGGATGTGAGGAGTGCCTCTGCCCGGCTGAGACCCCGTCTGGGAGGTGA
GGAGCGTCTCTGCCCGGCCGCCCCGTCTGAGAAGTGAGGAGACCCTCTGCCTGGCAACCA
CCCCGTCTGAGAAGTGAGGAGCCCCTCCGCCCGGCAGCTGCCCCGTCTGAGAAGTGAGGA
GCCTCTCCGCCCGGCAGCCACCCCATCTGGGAAGTGAGGAGCATCTCCGCCCAGCAGCCA
CCCCGTCCGGGAGGGAGGTGGGAGGGGGTCAACCCCCCGCCCGGCCAGCCGCCCCATCTG
GGAGGGAGGTGGGGGGTCAGCCCCCCCGACCGGCCAGCCGTGCCATCCGGGAGGGAGGTG
GGGGGGTCAGCCCCCCGCCCGGCCAGCCGCCCCGTCCGGGAGGTGAGGGGTGCCTCTGCC
CGGCCGCCCCTACTGGGAAGTGAGGAGCCCCTCAGCCCGGCCAGCCACCCCGTCCGGGAG
GGAGATGGGGGGGTCAGCCCCCCCACCCGGCCAGCCGCCCCGTCCGGGAGGGAGGTGGGG
GGGTAAGCCCCCCGCCTGGCCAGCCGCCCCGTCAGGAAGGGAGGTGGGGGGGTCAGCCCT
CCGCCCGGCCAGCCGCCCCGTCCGGGAGGTGAGGGGCGCCTCTGCCCGGCCGCCCCTACT
GGGAAGTGAGGAGCCCCTCTGCCCGGCCAGCCGCCCCGTCCGGGAGGGAGGTGGGGGGGG
TCAGCCCCCCGCCCGGCCGGCCGCCCTGTCCGGGAGGGAGGTGGGGGGGGTCAGCCCCCC
GCCTGGCCAGCCGCCCTGTCCGGGAGGGAGGTGGGGGGGGGTCAGCCCTCCGCCCGGCCA
GCCGCCCCGTCTGGGAGGTGAGGGGCGCCGCTGCCCGGCCGCCCCTACTGGGAAGTGAGG
AGCCCCTCTGCCCGGCCAGCCGCCCCGTCCGGGAGGGAGGTGGGGGGGGTCAGCCCCCCG
CCCGGCCGGCCGCCCTGTCCGGGAGGGAGGTGGGGGGGTCAGCCCTCCGCCCGGCCAGCC
GCCCCGTCCGGGAGGTGAGGGGCGCCTCTGCCCGGCCGCCCCTACTGGGAAGTGAGGAGC
CCCTCTGCCCGGCCAGCCGCCCCGTCCCGGAGGGAGGTGGGGGGGTCAGCCCCCCTGCC
CGGCCAGCCGCCCCGTCCGGGAGGTGAGGGGCGCCTCTGCCCAGCCGCCCCTACTGGGAA
GTGAGGAGCCCCTCTGCCCGGCCAGCCGCCCCGTCCGGGAGGGAGGTGGGGGGGTCAGC
CCCCCCGCCCGGCCAGCCGCCCCGTCCGGGAGGTGAGGGGCGCCTCTGCCCGGCCGCCCC
TACTGGGAAGTGAGGAGCCCCTCTGCCCGGCCACCACCCCGTCTGGGAGGTGTGCCCAAC
AGCTCATTGAGAACGGGCCAGGATGACAATGGCGGCTTTGTGGAATAGAAAGGCAGGAAA SINE-R
GGTGGGGAAAAGATTGAGAAATCGGATGGTTGCCGTGTCTGTGTAGAAAGAAGTAGACAT
GGGAGACTTTTCATTTTGTTCTGCACTAAGAAAAATTCCTCTGCCTTGGGATCCTGTTGA
TCTGTGACCTTACCCCCAACCCTGTGCTCTCTGAAACATGTGCTGTGTCCACTCAGGGTT
AAATGGATTAAGGGCGGTGCAAGATGTGCTTTGTTAAACAGATGCTTGAAGGCAGCATGC
TCGTTAAGAATCATCACCAATCCCTAATCTCAAGTAATCAGGGACACAAACACTGCGGAA
GGCCGCAGGGTCCTCTGCCTAGGAAAACCAGAGACCTTTGTTCACTTGTTTATCTGCTGA
CCTTCCCTCCACTATTGTCCCATGACCCTGCCAAATCCCCCTCTGTGAGAAACACCCAAG
AATTATCAATAAAAAAATAAATTTAAAAAAAAAAAAAAAGAATACAAAATA poly-adenylation signal, $A_{(11)}$, TSD

TSD: target site duplication
VNTR: variable number of tandem repeats

Figure S15: Sequence of the inserted SVA element in *SUZ12P* intron 8 of patient ASB4-55.

TTTTTTTTTTTTTTTTTTTTTTTTTTTTTTTTTTTTTTTTAAATTTATTT T$_{(40)}$, poly-adenylation signal
TTTTATTGATAATTCTTGGGTGTTTCTCACAGAGGGGGATTTGGCAGGGT SINE-R
CATGGGACAATAGTGGAGGGAAGGTCAGCAGATAAACAAGTGAACAAAGG
TCTCTGGTTTTCCTAGGCAGAGGACCCTGCGGCCTTCCGCAGTGTTTGTG
TCCCTGATTACTTGAGATTAGGGATTGGTGATGA**C**TCTTAACGAGCATGC
TGCCTTCAAGCATCTGTTTAACAAAGCACATCTTGCACCGCCCTTAATCC
ATTTAACCCTGAGTGGACACAGCACATGTTTCAGAGAGCACAGGGTTGGG
GGTAAGGTCACAGATCAACAGGATCCCAAGGCAGAGGAATTTTTCTTAGT
GCAGAACAAAATGAAAAGTCTCCCATGTCTACTTCTTTCTACACAGACAC
GGCAACCATCCGATTTCTCAATCTTTTCCCCACCTTTCCTGCCTTTCTAT
TCCACAAAGCCGCCATTGTCATCCTGGCCCGTTCTCAATGAGCTGTTGGG VNTR
CACACCTCCCAGACGGGGTGGTGGCCGGGCAGAGGGGCTCCTCACTTCCC
AGTAGGGGCGGCCGGGCAGAGGCGCCCCTCACCTCCCGGACGGGGCGGCT
GGCCGGGCGGGGGGGCTGACCCCCCCCACCTCCCTCCCGGACGGGGCGGC
TGGCCGGGCAGAGGGGCTGACACCCCCACCTCCCTCCCGGACGGGGCGGC
TGGCCGGGCAGAGGGGCTCCTCACTTCCCAGTAGGGGCGGCCGGGCAGAG
GCGCCCCTCACCTCCCGGACGGGGCGGCTGGCCGGGCAGGGGGGCTGACC
CCCCCCACCTCCCTCC**G**GGACGGGGCGGCTGGCCGGGCAGAGGGGCTCCT
CACTTCCCAGTAGGGGCGGC**C**GGGCAGAGGCGCCCCTCACCTCCCGGACG
GGGCGGCTGGCCGGGCAGGGGGGCTGACC**T**CCCCCACCTCCCTCCGGGAC
GGGGCGGCTGGCCGGGCAGAGGGGCTCCTCACTTCCCAGTAGGGGCGGCC
GGGCAGAGGCGCCCCTCACCTCCC**A**GACGGGGCGGCTGGCCGGGCGGAGG
GCTGACCCCCCACCTCCCTCCCGGACAGGGCGGCCGGCCGGGCGGGGGGC
TGACCCCCCCCACCTCCCTCCCGGACGGGGCGGCTGGCCGGGCAGAGGGG
CTCCTCACTTCCCAGTAGGGGCGGC**G**GGGCAGCGGCGCCCCTCACCTCCC
AGACGGGGCGGCTGGCCGGGCGGAGGGCTGACCCCCCCCCACCTCCCTCC
CGG

Bold letters: nucleotides that differ from those of the source element H6_1084.
Within the variable number of tandem repeats (VNTR) region indicated in green, a duplication of 178-bp (underlined sequence) was identified. The source element H6_1084 does not include this duplication which is specific to the SVA copy identified in patient ASB4-55.

Figure S16: Alignment of the reference sequence of *SUZ12P* intron 8 (hg19) against the corresponding region in patient DA-77. The polyT tract of the SVA element inserted into *SUZ12P* intron 8 in this patient is marked in red. The LINE 1 endonuclease (L1 EN), most likely involved in the insertion of this SVA element, is known to exhibit substrate specificity and cleaves at specific L1 EN consensus cleavage sites such as 5'-TTTT/A-3' and 5'-CTTT/A-3' (Morrish et al., 2002). The L1 EN cleavage site 5'-TTTT/A-3' in the reference sequence hg19 is highlighted in yellow and the position of cleavage is indicated by an arrow. The SNP rs8071236 (T/C) is located within this sequence motif as highlighted in blue. The chromosome 17 sequence of patient DA-77 harbouring the SVA insertion exhibited the C-allele of this SNP. Hence, the corresponding L1 EN cleavage site was 5'-CTTT/A-3'.

```
                                                 ↓
Reference    5'-TTTTGGCAGGGATTCTAATGAGTGATGTGTTTTTTACGTACTACATCACA-3'
DA-77        5'-TTTTGGCAGGGATTCTAATGAGTGATGTGTTCTTTTTTTTTTTTTTTTTT-3'
```

Figure S17: Alignment of the reference sequence of *SUZ12P* intron 8 (hg19) against the corresponding region in patient ASB4-55. The polyT tract of the SVA element inserted into *SUZ12P* intron 8 in this patient is marked in red. The LINE 1 endonuclease (L1 EN), most likely involved in the insertion of this SVA element, is known to exhibit substrate specificity and cleaves at specific L1 EN consensus cleavage sites such as 5'-CTTT/A-3' (Morrish et al., 2002). The L1 EN cleavage site 5'-CTTT/A-3' in the reference sequence hg19 is highlighted in yellow and the position of cleavage is indicated by an arrow.

```
                                             ↓
Reference    5'-AGGTTTTGAGACCTCAGGCATATTATACTTTACATGTTTAGAGTTATATC-3'
ASB4-55      5'-AGGTTTTGAGACCTCAGGCATATTATACTTTTTTTTTTTTTTTTTTTTTT-3'
```

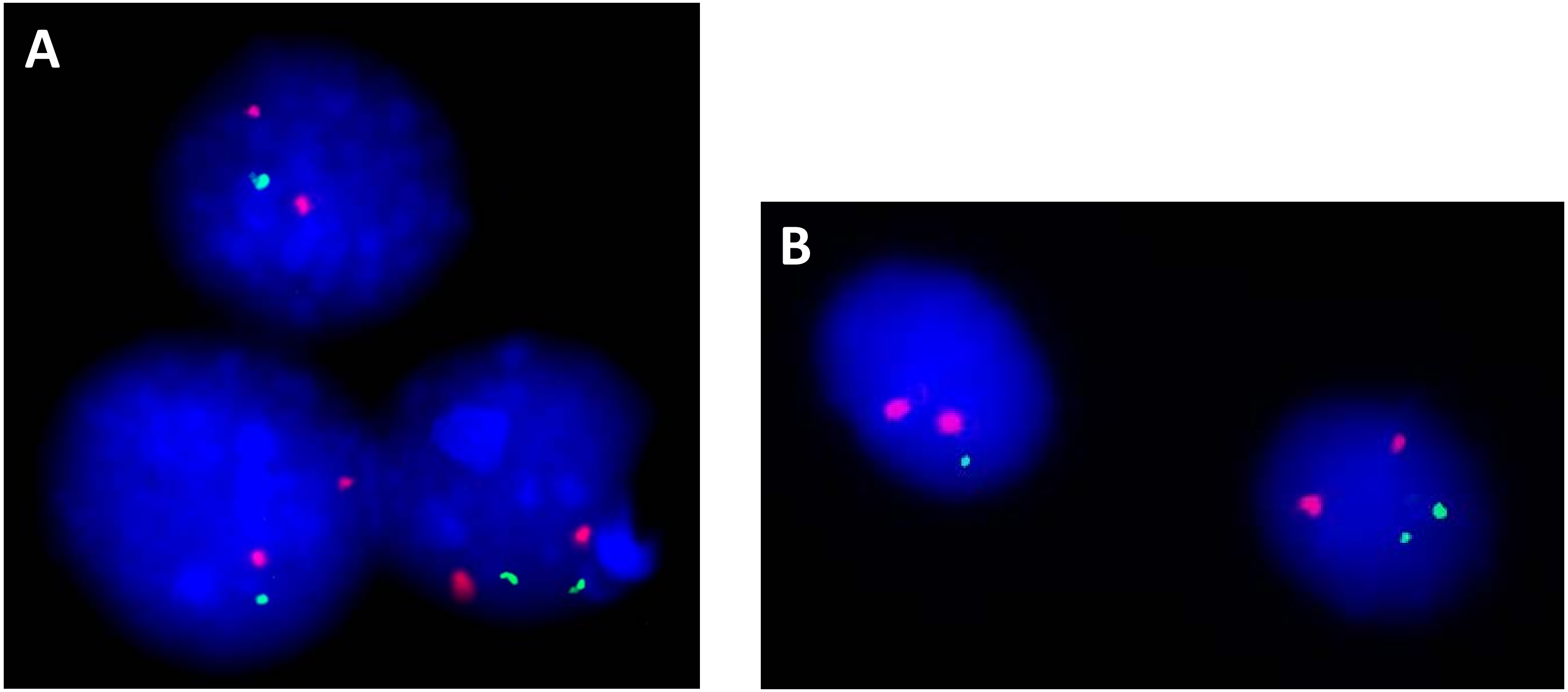

Figure S18: Representative FISH images indicating the somatic mosaicism of cells harbouring the *NF1* deletion and normal cells in the blood of the grandmother of patient DA-77 (A) and patient ASB4-55 (B). In each case, at least 200 interphase nuclei from cultured blood were investigated. Dual-colour FISH was performed with BAC RP11-142O6 which spans the proximal part of the *NF1* gene and the alpha-satellite enumeration probe SE17/D17Z1 (Kreatech, Amsterdam, Netherlands) which was used as a control. The *NF1* probe is visible in green whereas the control probe is visible in red.

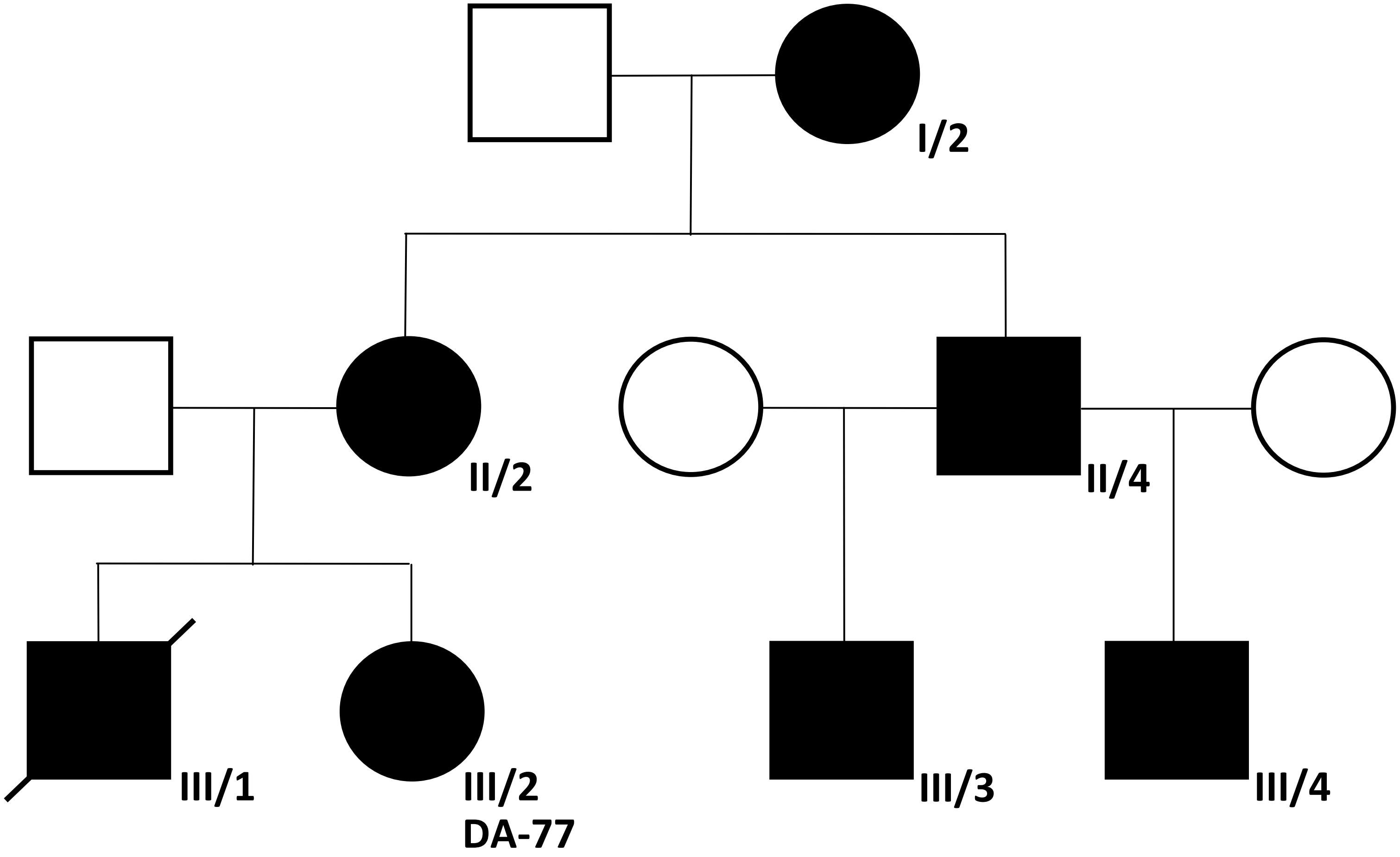

Figure S19: Segregation of the 1-Mb-spanning atypical *NF1* deletion in the family of patient DA-77 (III/2). The SVA insertion-associated *NF1* deletion was originally identified in patient III/2 and had occurred in the grandmother (I/2). It must have resulted from a postzygotic rearrangement since the grandmother (I/2) exhibited somatic mosaicism with normal cells as determined by FISH. The grandmother then passed on the SVA insertion-associated atypical *NF1* deletion to her offspring. The SVA insertion associated-deletion was verified in her grandchildren by PCR and sequence analysis of the corresponding PCR products (patients III/1, III/2, III/3 and III/4).

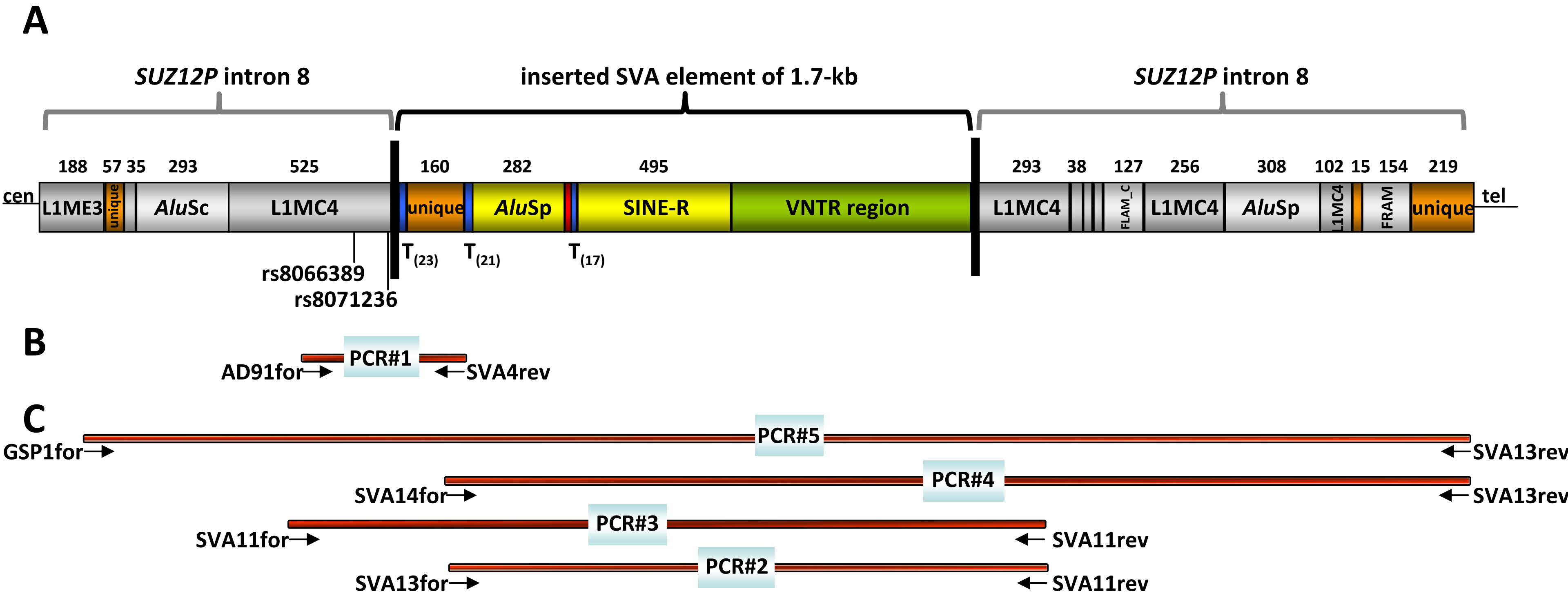

Figure S20: PCR analysis performed to investigate (i) whether the SVA insertion into *SUZ12P* intron 8 had occurred prior to the *NF1* deletion in the grandmother of patient DA-77 and (ii) whether the SVA insertion might represent a frequent insertion/deletion polymorphism at this position on chromosome 17 in healthy controls. **(A)** Structure of a hypothetical normal chromosome 17 lacking the large *NF1* deletion but possessing the insertion of the 1.7-kb SVA element within *SUZ12P* intron 8. **(B)** To investigate whether the insertion of the SVA element in *SUZ12P* intron 8 would be a frequent insertion/deletion polymorphism, 50 African and 50 white European DNA samples were analysed by PCR#1. However, PCR#1 was not positive in any of these DNA samples and hence it is unlikely that the SVA insertion would represent a frequent polymorphism. By contrast, PCR#1 was positive in patient DA-77 and her family members III/1, III/3, III/4 and I/2 who harboured the large *NF1* deletion and the SVA insertion at the deletion breakpoints. **(C)** To analyse whether the SVA insertion had preceded the occurrence of the large *NF1* deletion in the grandmother of patient DA-77, we investigated the potential presence of cells with the SVA insertion but lacking the large *NF1* deletion by PCR. The grandmother (I/2) exhibited somatic mosaicism in blood with 75% of cells harbouring the deletion whilst 25% of cells were normal as determined by FISH. However, PCR#2 and PCR#4 performed using blood-derived DNA from the grandmother were negative for the anticipated PCR products of 1.8-kb (PCR#2) and 3.3-kb (PCR#4) under the scenario of the SVA insertion being present whilst the large *NF1* deletion was absent. We therefore concluded that the grandmother did not possess a chromosome 17 which harbours the inserted SVA element without the large *NF1* deletion. This conclusion was further confirmed by PCRs #3 and #5 performed using genomic DNA from the grandmother. If the SVA element had been inserted into a chromosome 17 lacking the large *NF1* deletion, a PCR product of 4.3-kb would have been anticipated for PCR#5 and a 2.3-kb product for PCR#3. However, only shorter PCR products of 533-bp (PCR#3) and 2.6-kb (PCR#5) were obtained, which were derived from a normal chromosome 17 lacking the SVA element insertion. It might also have been possible that a full-length SVA element had inserted into chromosome 17. Therefore, PCRs #2-5 were also performed with elongation times ranging from 3–5 minutes in order to amplify PCR products up to 6.6-kb. However, under these conditions, only short PCR products of 533-bp (PCR#3) and 2.6-kb (PCR#5) were obtained, derived from a normal chromosome 17 lacking the SVA insertion. Sequence analysis of PCR#3 spanning 533-bp indicated the presence of two heterozygous SNPs (rs8066389 and rs8071236) thereby confirming the presence of two normal chromosomes 17 neither of which possessed an SVA insertion at the corresponding position in *SUZ12P* intron 8. We conclude that the large *NF1* deletion occurred concurrently with the SVA insertion.

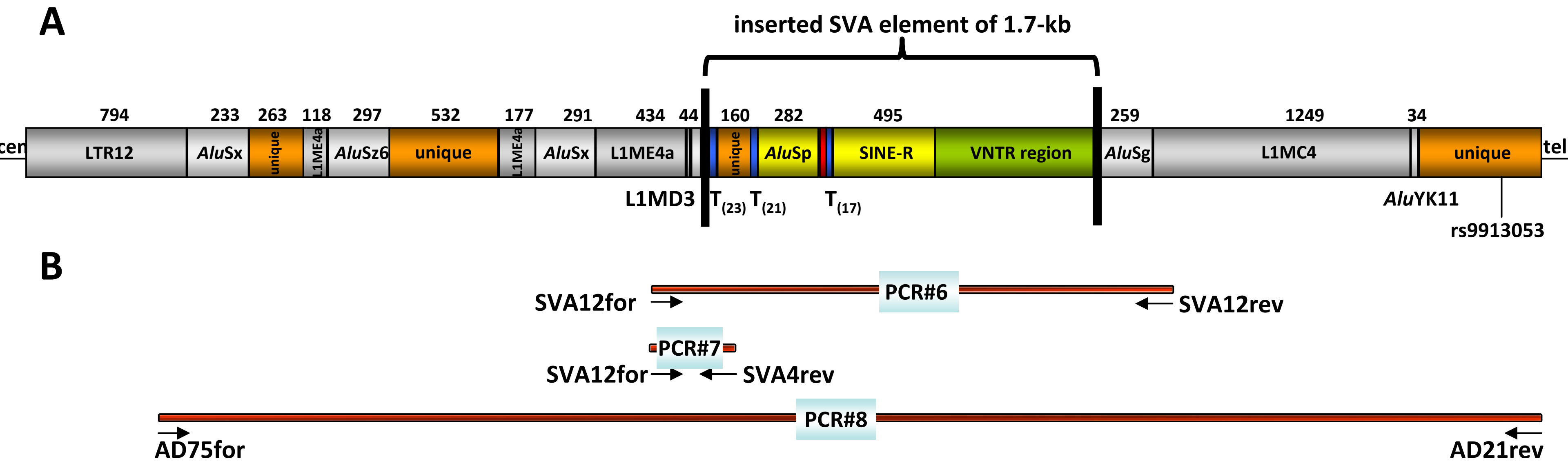

Figure S21: PCR analysis performed to investigate whether the SVA element identified at the deletion breakpoint had inserted into chromosome 17 within the intergenic region between *RAB11FIP4* and *COPRS* prior to the *NF1* deletion occurring in the grandmother of patient DA-77. The grandmother exhibited somatic mosaicism for the deletion which was present in 75% of her blood cells as determined by FISH. **(A)** Structure of a hypothetical normal chromosome 17 lacking the large *NF1* deletion but possessing the SVA insertion within the region between *RAB11FIP4* and *COPRS*. **(B)** In order to determine whether the insertion of the SVA element had occurred prior to the large *NF1* deletion in the grandmother of patient DA-77, we performed PCRs #6–8 using blood-derived DNA from the grandmother. PCR#7 was negative for the PCR product of 416-bp anticipated under the scenario of the SVA insertion being present whilst the large *NF1* deletion was absent. We concluded that the grandmother did not possess a chromosome 17 harbouring the inserted SVA element in the absence of the large *NF1* deletion. This conclusion was further confirmed by PCR#6. If the SVA element had been inserted into a chromosome 17 lacking the large deletion, a PCR product of 2.3-kb would have been expected for PCR#6. However, only a shorter PCR product of 588-bp was obtained which was derived from a normal chromosome 17 lacking the SVA insertion. It might also have been possible that a full-length SVA element had inserted into chromosome 17. Therefore, PCR#6 was also performed with an elongation time of 4 minutes in order to amplify a PCR product of 4-kb corresponding to the size of a full-length SVA element. However, under these conditions, only the short PCR product of 588-bp was obtained which was derived from a normal chromosome 17 without the insertion. Sequence analysis of the 4,684-bp PCR product of PCR#8 obtained with primers AD75for/AD21rev indicated the presence of one heterozygous SNP (rs9913053) thereby confirming the presence of two normal chromosomes 17 neither of which possessed an SVA insertion at the corresponding position within the intergenic region between *RAB11FIP4* and *COPRS*. We conclude that the large *NF1* deletion must have occurred concurrently with the SVA insertion in the grandmother of patient DA-77.

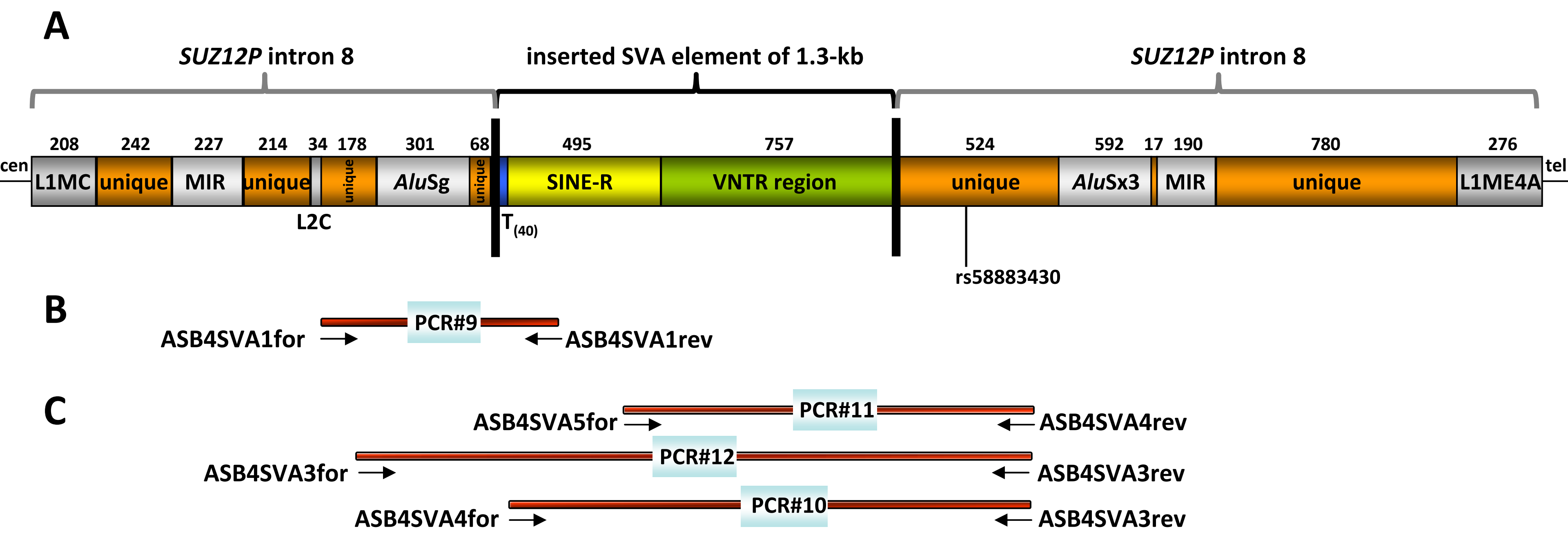

Figure S22: PCR analysis performed to investigate (i) whether the SVA element identified at the deletion breakpoint within *SUZ12P* intron 8 of patient ASB4-55 had inserted into chromosome 17 prior to the occurrence of the *NF1* deletion, and (ii) whether the SVA element might represent a frequent insertion/deletion polymorphism at this position on chromosome 17 in healthy controls. **(A)** Structure of a hypothetical chromosome 17 lacking the large *NF1* deletion but exhibiting the insertion of the 1.3-kb SVA element within *SUZ12P* intron 8. **(B)** To investigate whether the SVA insertion within *SUZ12P* intron 8 might represent a frequent insertion/deletion polymorphism, 50 African and 50 white European DNA samples were analysed by PCR#9. However, PCR#9 was not positive in any of these DNA samples and hence it is unlikely that the insertion represents a frequent polymorphism at this location in the human genome. By contrast, PCR#9 yielded a 800-bp spanning PCR product in patient ASB4-55 harbouring the large *NF1* deletion and the SVA insertion at the breakpoints. **(C)** In order to determine whether the insertion of the SVA element occurred prior to the large *NF1* deletion, we investigated genomic DNA from patient ASB4-55 by PCR. The patient exhibited somatic mosaicism for the deletion which was present in 93% of her blood cells as determined by FISH. PCRs #10 and #11 performed using blood-derived DNA from the patient as template were negative for the PCR product of 1.5-kb (PCR#10) and 1.3-kb (PCR#11) anticipated under the scenario of the SVA insertion being present whilst the large *NF1* deletion was absent. We therefore concluded that patient ASB4-55 did not possess a chromosome 17 lacking the large *NF1* deletion but harbouring the inserted SVA element within *SUZ12P* intron 8. This conclusion was further confirmed by PCR#12 performed using genomic DNA from the patient. If patient ASB4-55 were to possess a chromosome 17 with the SVA insertion but lacking the large *NF1* deletion, a PCR product of 2.1-kb would have been anticipated for PCR#12. However, PCR#12 yielded only a shorter PCR product of 854-bp which was derived from a normal chromosome 17 lacking the SVA insertion. It might also have been possible that a full-length SVA element had inserted into chromosome 17. Therefore, PCRs #10–12 were performed with elongation times of 3 minutes in order to amplify PCR products up to 4-kb corresponding to the size of a full-length SVA element. However, under these conditions, only shorter PCR products were obtained which were derived from a normal chromosome 17 lacking the SVA insertion. Sequence analysis of the PCR#12 product spanning 854-bp was indicative of one heterozygous SNP (rs58883430), thereby confirming the presence of two normal chromosomes 17 neither of which possessed an SVA insertion at the corresponding position in *SUZ12P* intron 8. We conclude that the large *NF1* deletion must have occurred concurrently with the SVA element insertion.

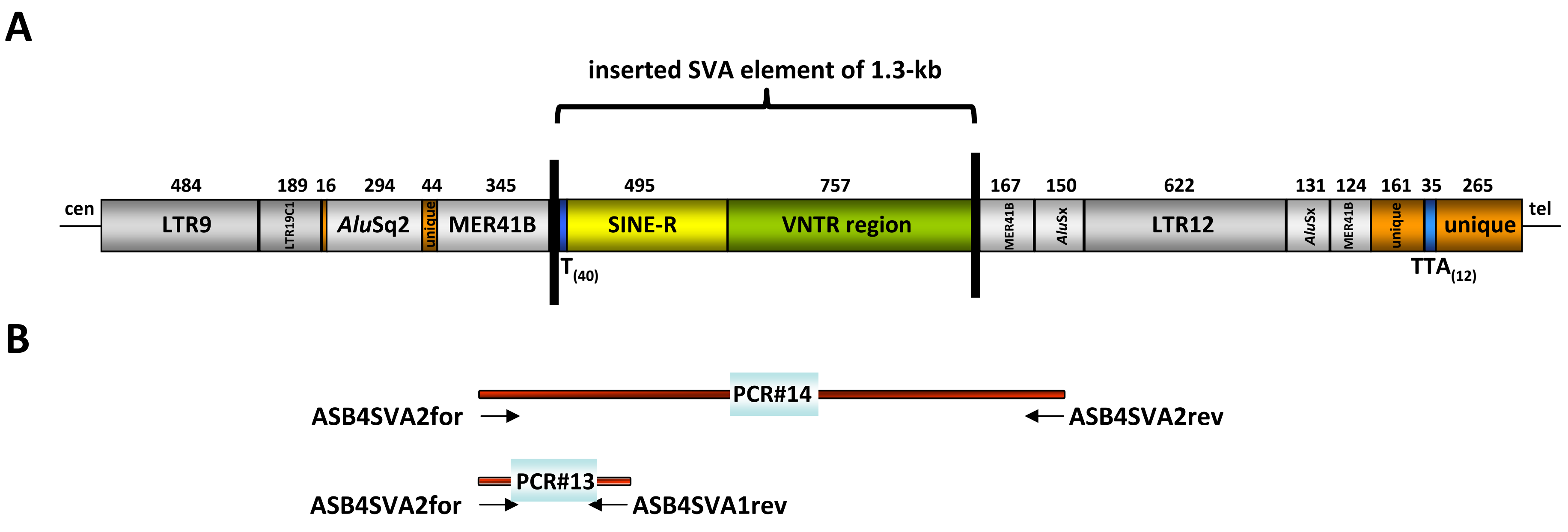

Figure S23: PCR analysis performed to investigate whether the SVA element identified at the deletion breakpoint of patient ASB4-55 had inserted into chromosome 17 within the intergenic region between *RAB11FIP4* and *COPRS* prior to the occurrence of the *NF1* deletion. **(A)** Structure of a hypothetical normal chromosome 17 lacking the large *NF1* deletion but possessing the 1.3-kb SVA element insertion within the intergenic region between *RAB11FIP4* and *COPRS*. **(B)** In order to ascertain whether the insertion of the SVA element occurred prior to the large *NF1* deletion, we investigated genomic DNA from patient ASB4-55. However, PCR#13 performed using blood-derived DNA of the patient as template was negative for the PCR product of 457-bp anticipated under the scenario of the SVA insertion being present whilst the large *NF1* deletion was absent. We concluded that patient ASB4-55 did not possess a normal chromosome 17 (lacking the *NF1* deletion) that nevertheless harboured the inserted SVA element within the intergenic region between *RAB11FIP4* and *COPRS*. This conclusion was further confirmed by PCR#14 performed using genomic DNA from the patient. If the SVA element had been inserted into a chromosome 17 lacking the large deletion, a PCR product of 1.7-kb would have been expected for PCR#14. However, only a shorter PCR product of 449-bp was obtained which was derived from a normal chromosome 17 without the SVA element insertion. It might also have been possible that a full-length SVA element had inserted into chromosome 17. Therefore, PCR#14 was also performed with an elongation time of 3 minutes in order to amplify PCR products up to 4-kb corresponding to the size of a full-length SVA element. However, under these conditions, only a short PCR product of 449-bp, derived from a normal chromosome 17 lacking the insertion, was obtained. We conclude that the large *NF1* deletion must have occurred concurrently with the SVA element insertion.

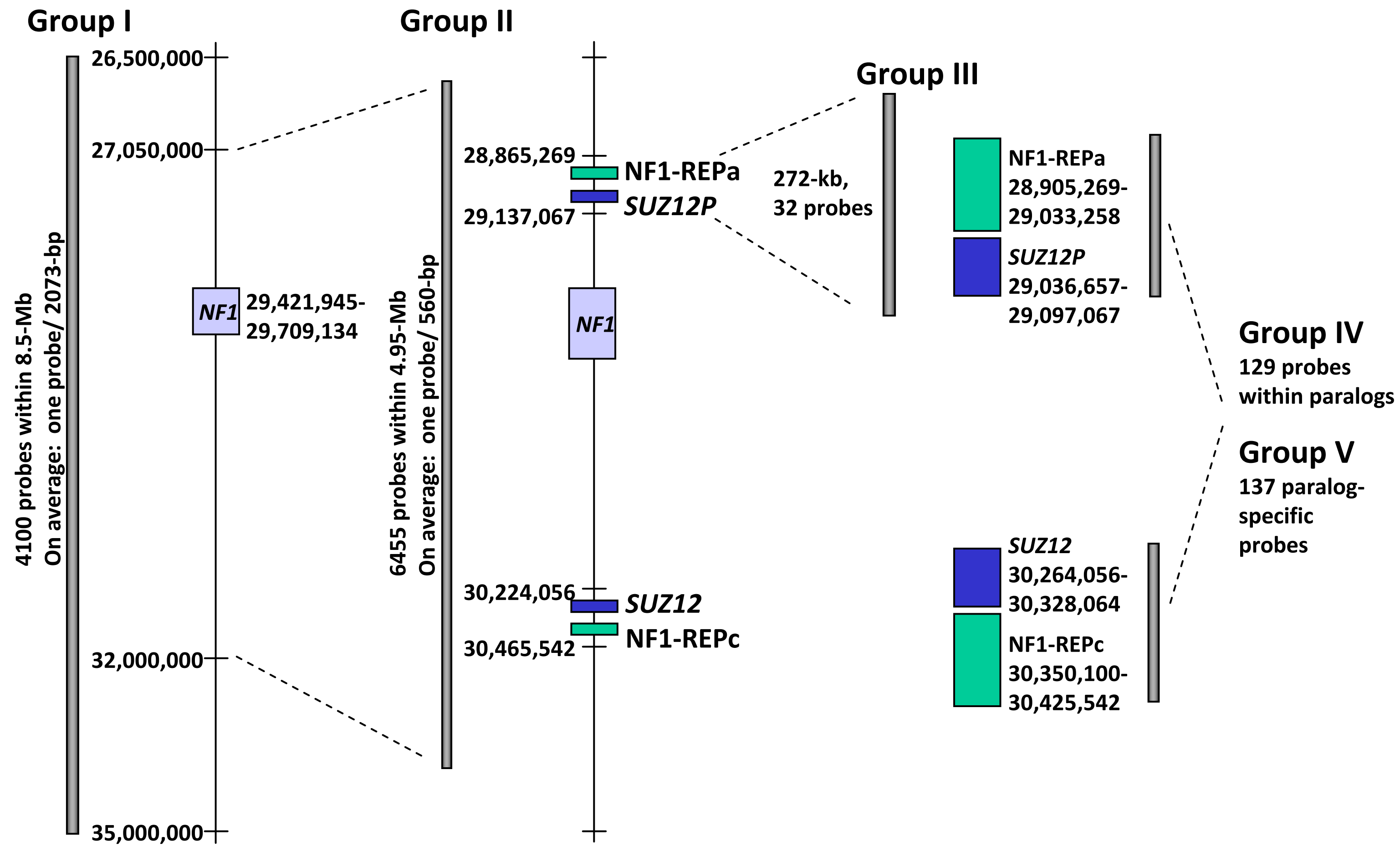

Figure S24: Design and analysis of the 8 x 15K custom array (Agilent Technologies, Santa Clara, CA, USA), which contained 15,744 oligonucleotide probes including 4,891 control probes as well as 10,853 test probes assigned to five different groups. The probes in groups I-III were selected from the Agilent eArray library, which provides validated catalogue probes, avoiding all common repeats and other redundant sequences. Since the coverage of the paralogous NF1-REPs and the *SUZ12* sequences with regard to these catalogue probes is low, we designed additional customized probes (129 probes in group IV and 137 probes in group V) located within these segmental duplications. For this purpose, we used the genomic tiling approach of the eArray tool (Agilent eArray library, https://earray.chem.agilent.com/earray/). The 129 customized probes in group IV were located within regions of absolute sequence identity between the paralogs whereas the 137 probes in group V were designed so as to contain several paralogous sequence variants in order to potentiate paralog-specific hybridization. Restriction enzymatic digestion of 0.5 µg genomic DNA from the patient as well as from a sex-matched control was performed with a mixture of *Alu*I and *Rsa*I at 37°C for two hrs. Sample labelling of the restriction-digested genomic DNA samples with Cy5-dUTP or Cy3-dUTP, respectively, was performed using the Genomic DNA Enzymatic Labeling Kit (Agilent Technologies). Sample hybridization and washing of the microarrays was carried out by means of the Oligo aCGH-on-chip Hybridization and Wash Buffer Kits (Agilent Technologies). Fluorescent intensities were detected with Scan Control A.8.4.1 Software on the Agilent DNA Microarray Scanner and extracted from the images using Feature Extraction 10.7.3.1 Software (Agilent Technologies) and the design file 033151_D_F_20110323.xml. The software tools Feature Extraction 10.7.3.1 and Genomic Workbench Lite 6.0.130.24 (CGH module) were used for quality control, annotation, statistical data analysis and visualization. The quality of the individual microarrays used in the experiments was validated against the quality metrics (QCmetrics) of this software (Feature Extraction 10.7.3.1). The microarray data were normalized to compensate for varying global signal intensities and to adjust them for downstream analyses. The identification of aberrant regions was performed with the analysis software Genomic Workbench Lite using the aberration algorithm ADM-2 in combination with a centralization algorithm. The analysis of the microarrays was performed by IMGM Laboratories (Martinsried, Germany).

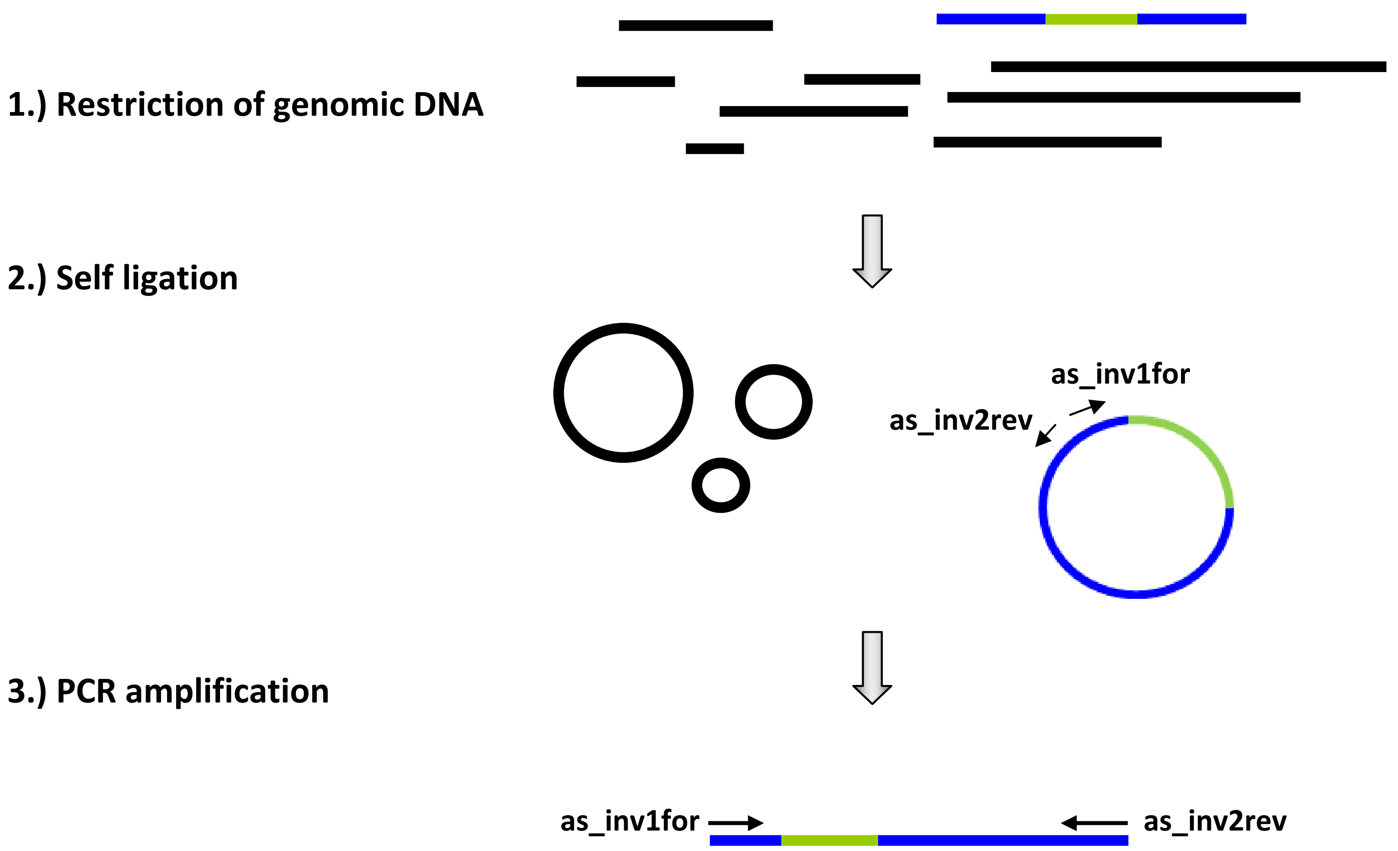

Figure S25: Principle of the inverse PCR technique exemplified in the context of the characterization of the centromeric breakpoint of the *NF1* deletion in patient ASB4-55. In step 1, 10 µg genomic DNA derived from blood of the patient was restriction-digested with *Pci*I (New England Biolabs, Ipswich, USA). Among the resulting DNA fragments is the target fragment harbouring the deletion junction with the unknown inserted sequence (green) immediately flanked by non-deleted sequences (blue). The restriction fragments were purified with the QIAquick Nucleotide Removal Kit (Qiagen, Hilden, Germany) and eluted in 50 µl water. In step 2, self-ligation of the DNA fragments was set up in a final volume of 1 ml including 50 units T4 DNA ligase (Promega, Mannheim, Germany) and incubated overnight at 16°C. Subsequently, the self-ligation reaction mixture was purified and concentrated with the QIAquick Nucleotide Removal Kit and used as a template for PCR with inversely oriented primers (as_inv2rev and as_inv1for) located in regions flanking the telomeric deletion breakpoint (step 3). These primers were located within unique, non-repetitive sequences in the vicinity of non-deleted regions. The resulting PCR products were cloned using the StrataClone PCR Cloning Kit (Agilent Technologies, Santa Clara, USA) and sequenced from both ends using M13 primers. All enzymes and primers used to analyse the deletion breakpoint-flanking regions in patients DA-77 and ASB4-55 by inverse PCR are listed in Tables S28 and S29.

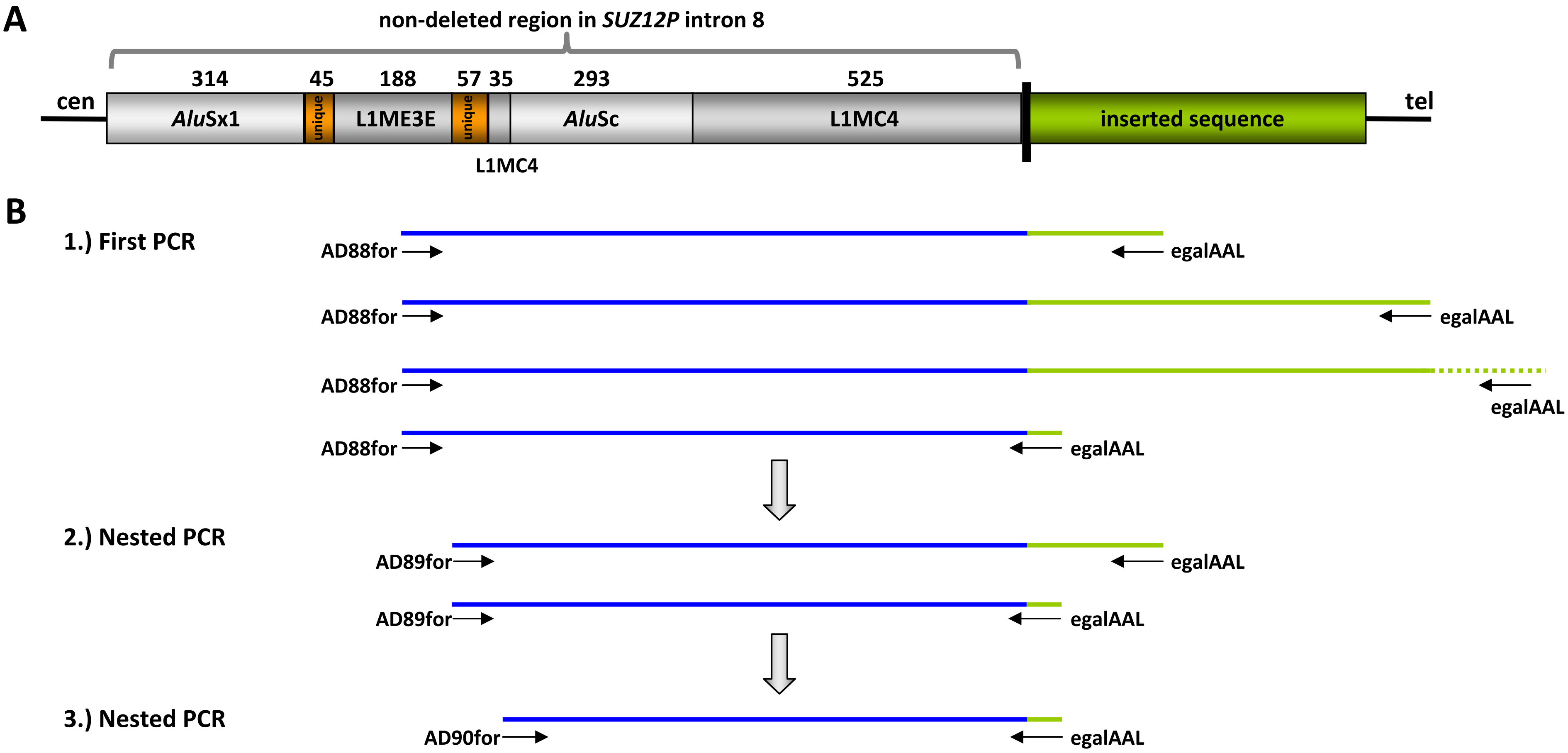

Figure S26: Semi-specific PCR performed to narrow down the breakpoint regions and to identify the unknown sequences inserted at the deletion breakpoints. The principle of the assay is explained using the identification of the centromeric deletion breakpoint in patient DA-77 as an example. **(A)** Schema of the centromeric deletion breakpoint region in patient DA-77. **(B)** The first PCR was performed with the region-specific forward primer (AD88for) located within the non-deleted reference sequence (blue) in combination with a non-specific return PCR primer (egalAAL) which is expected to bind within the inserted, unknown sequence (green). In this first step, many different PCR fragments were amplified which were distinguishable by their lengths as schematically indicated. An aliquot of 4 µl (100 ng) of the resulting heterogeneous PCR products was used as a template for a nested PCR (step 2) using a region-specific primer AD89for together with the non-specific return primer egalAAL. In step 3 of the assay, a further nested PCR was performed using 4 µl of the second PCR as a template and the primers AD90for and egalAAL. Subsequently, the PCR products resulting from step 3 were subject to direct sequence analysis or were cloned and sequenced from both ends using vector-based M13 primers. All PCRs were performed using the Expand Long Template PCR system (Roche, Mannheim, Germany). All primers used to analyse the deletion breakpoint-flanking regions in patients DA-77 and ASB4-55 by semispecific PCR are listed in Tables S30 and S31.

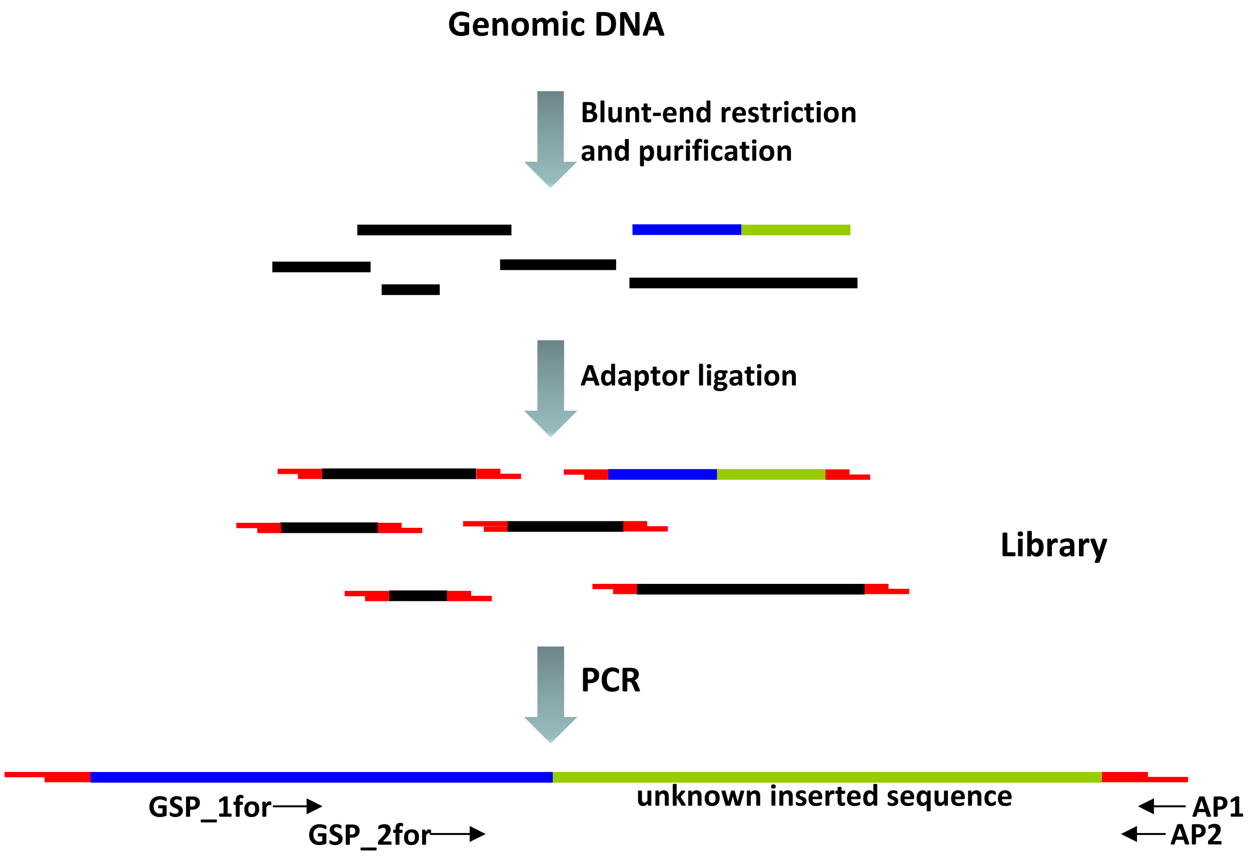

Figure S27: Principle of the GenomeWalker assay used to identify unknown sequences inserted at the deletion breakpoints of patients DA-77 and ASB4-55. In the first step, 2.5 µg genomic DNA was digested with a blunt-end restriction enzyme (New England Biolabs, Ipswich, USA) for two hours at the enzyme-specific temperature. Among the restricted DNA fragments was the target fragment that encompasses the deletion breakpoint region (blue) and the adjacent unknown sequence (green). After inactivation of the enzyme, the fragmented DNA was purified by means of the Nucleotide Removal Kit (Qiagen, Hilden, Germany) and resolved in a final volume of 30 µl TE-buffer. An aliquot (4 µl) of the purified DNA fragments was then added to the ligation-reaction which also included 1.9 µl oligonucleotide adaptors (indicated in red), 1.6 µl T4 DNA ligase buffer and 0.5 µl T4 DNA ligase. The ligation was performed at 16°C overnight. The next day, the reaction was inactivated at 70°C for 5 minutes, 72 µl TE-buffer were added and this library of adaptor-ligated restriction fragments was then used as a template for two subsequent PCR steps. The first-step PCR was performed with a region-specific primer (GSP_1for) located within the non-deleted sequence close to the deletion breakpoint (blue) and the adaptor primer (AP1) which hybridized to the adaptor. Subsequently, an aliquot of this first PCR was used as template for the second PCR using a nested region-specific primer (GSP_2for) and the adaptor primer AP2. Both PCRs were performed with the Advantage® 2 PCR Kit (Clontech, Saint-Germain-en-Laye, France), according to the manufacturer's instructions. The PCR products were then gel-purified (S.N.A.P.™ UV-Free Gel Purification Kit, Invitrogen, CA, USA) and cloned (StrataClone PCR Cloning Kit, Agilent Technologies, Santa Clara, CA, USA) prior to sequence analysis. All enzymes and primers used for these assays are listed in Tables S32 and S33.

References cited in Tables S1-S33 and Figures S1-S27 of the additional data

Cer RZ, Bruce KH, Mudunuri US, Yi M, Volfovsky N, Luke BT, Bacolla A, Collins JR, Stephens RM: **Non-B DB, a database of predicted non-B DNA-forming motifs in mammalian genomes**. *Nucleic Acids Res* 2011, **39**:D383-391.

Cer RZ, Donohue DE, Mudunuri US, Temiz NA, Loss MA, Starner NJ, Halusa GN, Volfovsky N, Yi M, Luke BT, Bacolla A, Collins JR, Stephens RM: **Non-B DB v2.0, a database of predicted non-B DNA-forming motifs and its associated tools**. *Nucleic Acids Res* 2013, **41**:D94-100.

Cnossen MH, van der Est MN, Breuning MH, van Asperen CJ, Breslau-Siderius EJ, van der Ploeg AT, de Goede-Bolder A, van den Ouweland AM, Halley DJ, Niermeijer MF: **Deletions spanning the neurofibromatosis type 1 gene, implications for genotype-phenotype correlations in neurofibromatosis type 1?** *Hum Mutat* 1997, **9**:458-464.

Dorschner MO, Sybert VP, Weaver M, Pletcher BA, Stephens K: ***NF1* microdeletion breakpoints are clustered at flanking repetitive sequences**. *Hum Mol Genet* 2000, **9**:35-46.

Jenne DE, Tinschert S, Stegmann E, Reimann H, Nürnberg P, Horn D, Naumann I, Buske A, Thiel G: **A common set of at least 11 functional genes is lost in the majority of NF1 patients with gross deletions**. *Genomics* 2000, **66**:93-97.

Kayes LM, Burke W, Riccardi VM, Benett R, Ehrlich P, Rubinstein A, Stephens K: **Deletions spanning the neurofibromatosis I gene, identification and phenotype of five patients.** *Am J Hum Genet* 1994, **54:**424-436.

Kayes LM, Riccardi VM, Burke W, Bennett RL, Stephens K: **Large *de novo* DNA deletion in a patient with sporadic neurofibromatosis 1, mental retardation, and dysmorphism**. *J Med Genet* 1992, **29**:686-690.

Kehrer-Sawatzki H, Kluwe L, Fünsterer C, Mautner VF: **Extensively high load of internal tumors determined by whole body MRI scanning in a patient with neurofibromatosis type 1 and a non-LCR-mediated 2-Mb deletion in 17q11.2**. *Hum Genet* 2005, **116**:466-475.

Kehrer-Sawatzki H, Schmid E, Fünsterer C, Kluwe L, Mautner VF: **Absence of cutaneous neurofibromas in an NF1 patient with an atypical deletion partially overlapping the common 1.4 Mb microdeleted region.** *Am J Med Genet A* 2008, **146A:**691-699.

Kehrer-Sawatzki H, Tinschert S, Jenne DE: **Heterogeneity of breakpoints in non-LCR-mediated large constitutional deletions of the 17q11.2 *NF1* tumor suppressor region.** *J Med Genet* 2003, **40:**E116.

Lee J, Ha J, Son SY, Han K: **Human genomic deletions generated by SVA-associated events.** *Comp Funct Genomics* 2012, **2012:** 807270.

Maertens O, De Schepper S, Vandesompele J, Brems H, Heyns I, Janssens S, Speleman F, Legius E, Messiaen L: **Molecular dissection of isolated disease features in mosaic neurofibromatosis type 1.** *Am J Hum Genet* 2007, **81:**243-251.

Mantripragada KK, Thuresson AC, Piotrowski A, Díaz de Ståhl T, Menzel U, Grigelionis G, Ferner RE, Griffiths S, Bolund L, Mautner V, Nordling M, Legius E, Vetrie D, Dahl N, Messiaen L, Upadhyaya M, Bruder CE, Dumanski JP: **Identification of novel deletion breakpoints bordered by segmental duplications in the *NF1* locus using high resolution array-CGH.** *J Med Genet* 2006, **43:**28-38.

Morrish TA, Gilbert N, Myers JS, Vincent BJ, Stamato TD, Taccioli GE, Batzer MA, Moran JV: **DNA repair mediated by endonuclease-independent LINE-1 retrotransposition.** *Nat Genet* 2002, **31:**159-165.

Pasmant E, de Saint-Trivier A, Laurendeau I, Dieux-Coeslier A, Parfait B, Vidaud M, Vidaud D, Bièche I: **Characterization of a 7.6-Mb germline deletion encompassing the *NF1* locus and about a hundred genes in an *NF1* contiguous gene syndrome patient.** *Eur J Hum Genet* 2008, **16:**1459-1466.

Pasmant E, Sabbagh A, Masliah-Planchon J, Haddad V, Hamel MJ, Laurendeau I, Soulier J, Parfait B, Wolkenstein P, Bièche I, Vidaud M, Vidaud D: **Detection and characterization of *NF1* microdeletions by custom high resolution array CGH.** *J Mol Diagn* 2009, **11:**524-529.

Pasmant E, Sabbagh A, Spurlock G, Laurendeau I, Grillo E, Hamel MJ, Martin L, Barbarot S, Leheup B, Rodriguez D, Lacombe D, Dollfus H, Pasquier L, Isidor B, Ferkal S, Soulier J, Sanson M, Dieux-Coeslier A, Bièche I, Parfait B, Vidaud M, Wolkenstein P, Upadhyaya M, Vidaud D; members of the NF France Network: ***NF1* microdeletions in neurofibromatosis type 1, from genotype to phenotype.** *Hum Mutat* 2010, **31:**E1506-1518.

Riva P, Corrado L, Natacci F, Castorina P, Wu BL, Schneider GH, Clementi M, Tenconi R, Korf BR, Larizza L: ***NF1* microdeletion syndrome, refined FISH characterization of sporadic and familial deletions with locus-specific probes.** *Am J Hum Genet* 2000, **66:**100-109.

Upadhyaya M, Roberts SH, Maynard J, Sorour E, Thompson PW, Vaughan M, Wilkie AO, Hughes HE: **A cytogenetic deletion, del(17)(q11.22q21.1), in a patient with sporadic neurofibromatosis type 1 (NF1) associated with dysmorphism and developmental delay.** *J Med Genet* 1996, **33:**148-152.

Venturin M, Gervasini C, Orzan F, Bentivegna A, Corrado L, Colapietro P, Friso A, Tenconi R, Upadhyaya M, Larizza L, Riva P: **Evidence for non-homologous end joining and nonallelic homologous recombination in atypical *NF1* microdeletions.** *Hum Genet* 2004a, **115:**69-80.

Venturin M, Guarnieri P, Natacci F, Stabile M, Tenconi R, Clementi M, Hernandez C, Thompson P, Upadhyaya M, Larizza L, Riva P: **Mental retardation and cardiovascular malformations in *NF1* microdeleted patients point to candidate genes in 17q11. 2.** *J Med Genet* 2004b**, 41:**35-41.

Vogt J, Mussotter T, Bengesser K, Claes K, Högel J, Chuzhanova N, Fu C, van den Ende J, Mautner VF, Cooper DN, Messiaen L, Kehrer-Sawatzki H: **Identification of recurrent type-2 *NF1* microdeletions reveals a mitotic nonallelic homologous recombination hotspot underlying a human genomic disorder.** *Hum Mutat* 2012, **33:**1599-1609.

Wang AJ, Quigley GJ, Kolpak FJ, van der Marel G, van Boom JH, Rich A: **Left-handed double helical DNA, variations in the backbone conformation.** *Science* 1981, **211:**171-176.
